# Chronic back pain sub-grouped via psychosocial, brain and physical factors using machine learning

**DOI:** 10.1038/s41598-022-19542-5

**Published:** 2022-09-07

**Authors:** Scott D. Tagliaferri, Tim Wilkin, Maia Angelova, Bernadette M. Fitzgibbon, Patrick J. Owen, Clint T. Miller, Daniel L. Belavy

**Affiliations:** 1 Institute for Physical Activity and Nutrition, School of Exercise and Nutrition Sciences, Deakin University, 221 Burwood Highway, Geelong, Burwood, VIC 3125 Australia; 2 Data to Intelligence Research Centre, School of Information Technology, Deakin University, Geelong, VIC Australia; 3 Department of Psychiatry, Faculty of Medicine, Nursing and Health Sciences, Monash University, Melbourne, VIC Australia; 4 Monarch Research Group, Monarch Mental Health Group, Sydney, NSW Australia; 5 Division of Physiotherapy, Department of Applied Health Sciences, Hochschule Für Gesundheit (University of Applied Sciences), Gesundheitscampus 6-8, 44801 Bochum, Germany

**Keywords:** Diagnosis, Musculoskeletal system

## Abstract

Chronic back pain (CBP) is heterogenous and identifying sub-groups could improve clinical decision making. Machine learning can build upon prior sub-grouping approaches by using a data-driven approach to overcome clinician subjectivity, however, only binary classification of pain versus no-pain has been attempted to date. In our cross-sectional study, age- and sex-matched participants with CBP (n = 4156) and pain-free controls (n = 14,927) from the UkBioBank were included. We included variables of body mass index, depression, loneliness/social isolation, grip strength, brain grey matter volumes and functional connectivity. We used fuzzy c-means clustering to derive CBP sub-groups and Support Vector Machine (SVM), Naïve Bayes, k-Nearest Neighbour (kNN) and Random Forest classifiers to determine classification accuracy. We showed that two variables (loneliness/social isolation and depression) and five clusters were optimal for creating sub-groups of CBP individuals. Classification accuracy was greater than 95% for when CBP sub-groups were assessed only, while misclassification in CBP sub-groups increased to 35–53% across classifiers when pain-free controls were added. We showed that individuals with CBP could sub-grouped and accurately classified. Future research should optimise variables by including specific spinal, psychosocial and nervous system measures associated with CBP to create more robust sub-groups that are discernible from pain-free controls.

## Introduction

Back pain generates a substantial global burden as the leading cause of disability^1^. Chronic pain defined as pain lasting greater than 12 weeks, contribute to the majority of financial^2^, physical and mental burden in back pain cases^3^. As high as 90% of back pain cases are classed as non-specific, meaning that there is no clear pathoanatomical cause for the experienced pain^4^. Heterogenous clinical presentations in spinal tissue, pain processing mechanisms and psychological state have been shown in those with chronic back pain (CBP)^5^; and provides a strong rationale for the development and implementation of an effective sub-classification approach in clinical practice to improve treatment outcomes for clients with chronic low back pain.

Previous attempts at back pain sub-classification have had minimal impact on the disease burden.^6^; A limitation of prior approaches is that they have investigated specific pain mechanisms^7^ or spinal tissues^8–10^ in isolation. This is in contrast to the current understanding of CBP, which is known to be influenced by an interplay between nervous system, psychosocial and physical factors^11^. Using measures of the central nervous system, psychosocial and physical health together, may allow for more appropriate sub-grouping of back pain individuals^11^. Machine learning (ML) can analyse data and recognise patterns, which is of particular interest in heterogenous pain presentations^12^. However, a systematic review of artificial intelligence in low back pain research has showed that only the binary classification (e.g. low back pain versus healthy controls) has been completed to date, and there has been no published attempts to sub-classify low back pain clients based on clinical presentation^13^. Therefore, the next step to implementing such tools in back pain research is to determine if sub-classification in back pain is possible using data science approaches.

The UKBioBank^14^, which recruited 500,000 individuals across the United Kingdom to track health-related outcomes such as brain function and structure, psychosocial and physical health, presents an opportunity to develop approaches to sub-classification of CBP using data science tools. We have previously analysed differences in brain structure, psychosocial, and physical health in those with acute or chronic and localised or widespread back pain in the UKBioBank^15^; however, exploring individual differences through data-driven classification could lead to an improved management of the condition. Therefore, the aim of this study was to develop a ML approach for CBP sub-grouping, based on clustering and classification methods using central nervous system, psychosocial and physical factors from the UKBioBank. A secondary aim was to determine the robustness of these sub-groups by adding pain-free individuals into classification methods and to test clustering within each domain (e.g. psychosocial, physical and central nervous system variables alone).

## Results

### Demographics, false discovery rate, feature weighting and cluster validation

**Figure 1:**
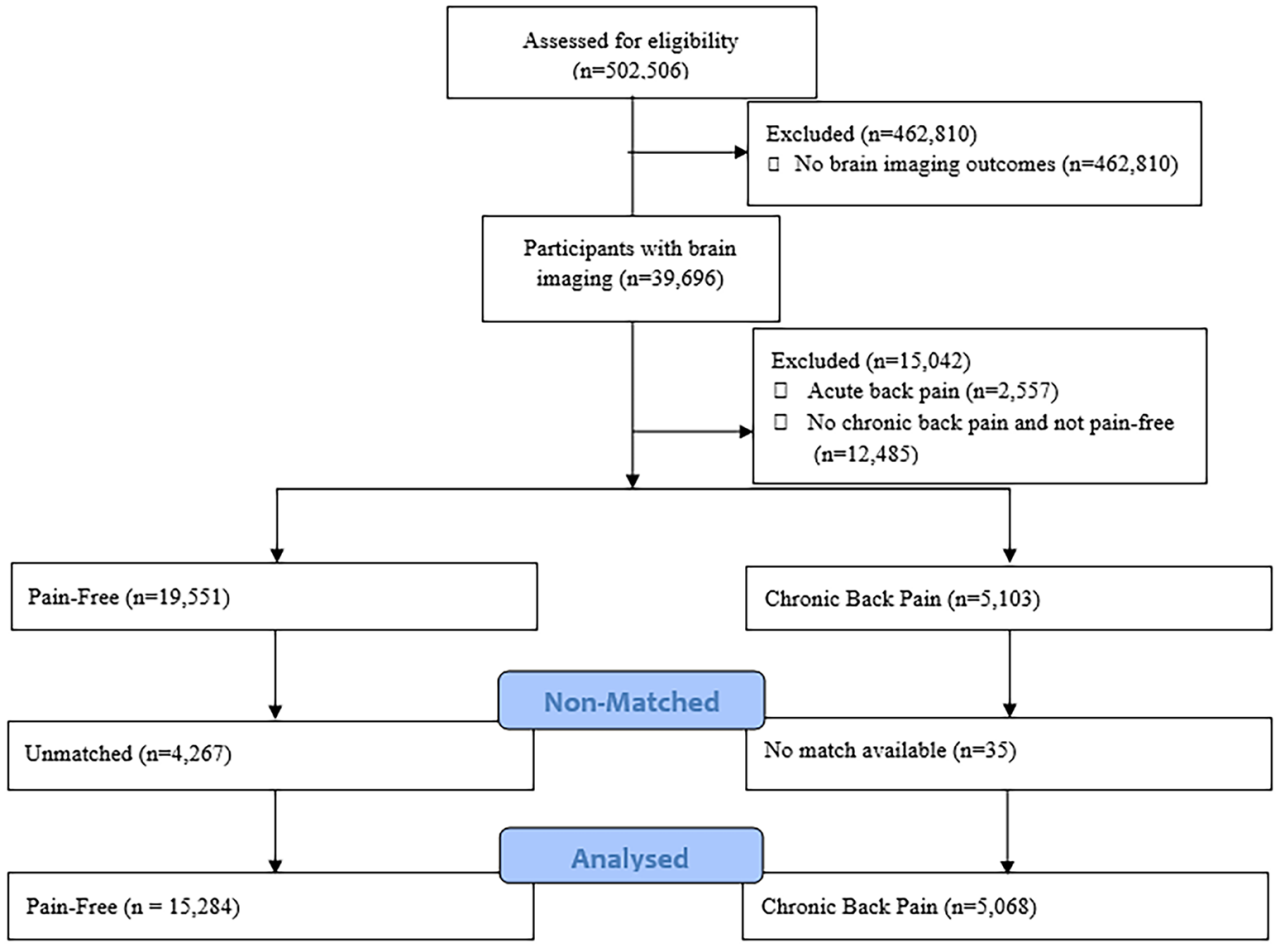
Flow diagram of participant selection for this UKBioBank sub-study.

The results of demographic data are presented in Table 1 and the participant flow in Fig. 1. The following steps were used to determine important variables to use at each point in the analyses:

- *Step 1:* Of the 1502 variables used in this study, 100 were significantly different between CBP and pain-free controls from t-tests after adjustment of *p* values using the false discovery rate method (Supplementary Table 2). Assessment for multicollinearity showed that no variables were highly correlated (Supplementary Table 3). These 100 variables were analysed in subsequent feature weighting methods to determine the most important variables for differentiating CBP and pain-free controls.
- *Step 2:* Following feature weighting tests, ten variables showed potential importance for differentiating CBP and pain-free individuals and were used in subsequent analyses (Supplementary Figures 1 and 2). These variables included depressive symptoms, symptoms of loneliness and social isolation, body mass index, grip strength, frontoparietal to default mode network connectivity (FPN-DMN; edge 1014), right frontoparietal to sensorimotor network connectivity (FPN-SMN; edge 222) and visual to default mode network connectivity (VN-DMN; edge 44), primary motor, primary somatosensory and fronto-orbital cortex grey matter volumes.
- *Step 3:* Using Laplacian scores to rank features based on variability (distances in the data space) for CBP only, the order of importance of features in a CBP-only space was symptoms of loneliness/social isolation, depressive symptoms, FPN-DMN connectivity (edge 1014), grip strength, body mass index, FPN-SMN connectivity (edge 222) and VN-DMN connectivity (edge 44), fronto-orbital cortex, primary motor and somatosensory grey matter volumes (Supplementary Figure 3).
- *Step 4:* For cluster validity techniques, adding dimensions in the order of importance in Step 3 demonstrated that clustering in three or more dimensions added no new information; hence, two dimensions (loneliness/social isolation and depressive symptoms) were used for the main clustering analysis (Supplementary Table 4). Results from clustering validity methods indicated the five clusters were optimal for the two-dimensional space (Supplementary Table 4).

**Table 1:** Demographics.

	Pain-free controls (n = 15,284)	Chronic back pain (n = 5068)
	Mean (SD)	Mean (SD)
Age	63.5 (7.6)	63.5 (7.7)
Sex (male/female—n)	6772/8493	2258/2834
Height (cm)	169.8 (9.3)	169.8 (9.6)
Weight (kg)	74.1 (14.3)	78.2 (16.1)
**Qualifications n (%)**		
College/University	8038 (53)	2138 (42)
A/AS	1925 (13)	580 (11)
O/GCSE	2586 (17)	997 (20)
CSE	464 (3)	264 (5)
NVQ or HND or HNC	755 (5)	351 (7)
Other qualification	728 (5)	324 (6)
None of the above/did not answer	788 (5)	442 (9)
**Employment status n (%)**		
Paid employment or self-employed	6126 (40)	1920 (38)
Retired	8655 (57)	2889 (57)
Looking after home or family	214 (1)	95 (2)
Unable to work from sickness or disability	22 (0.1)	88 (2)
Unemployed	60 (0.4)	33 (1)
Unpaid or voluntary work	115 (0.8)	38 (1)
Student	19 (0.1)	5 (0.1)
None of the above/did not answer	73 (0.5)	28 (0.6)

Data are presented as mean (SD) unless specified.

### Clusters of individuals with chronic back pain

**Figure 2:**
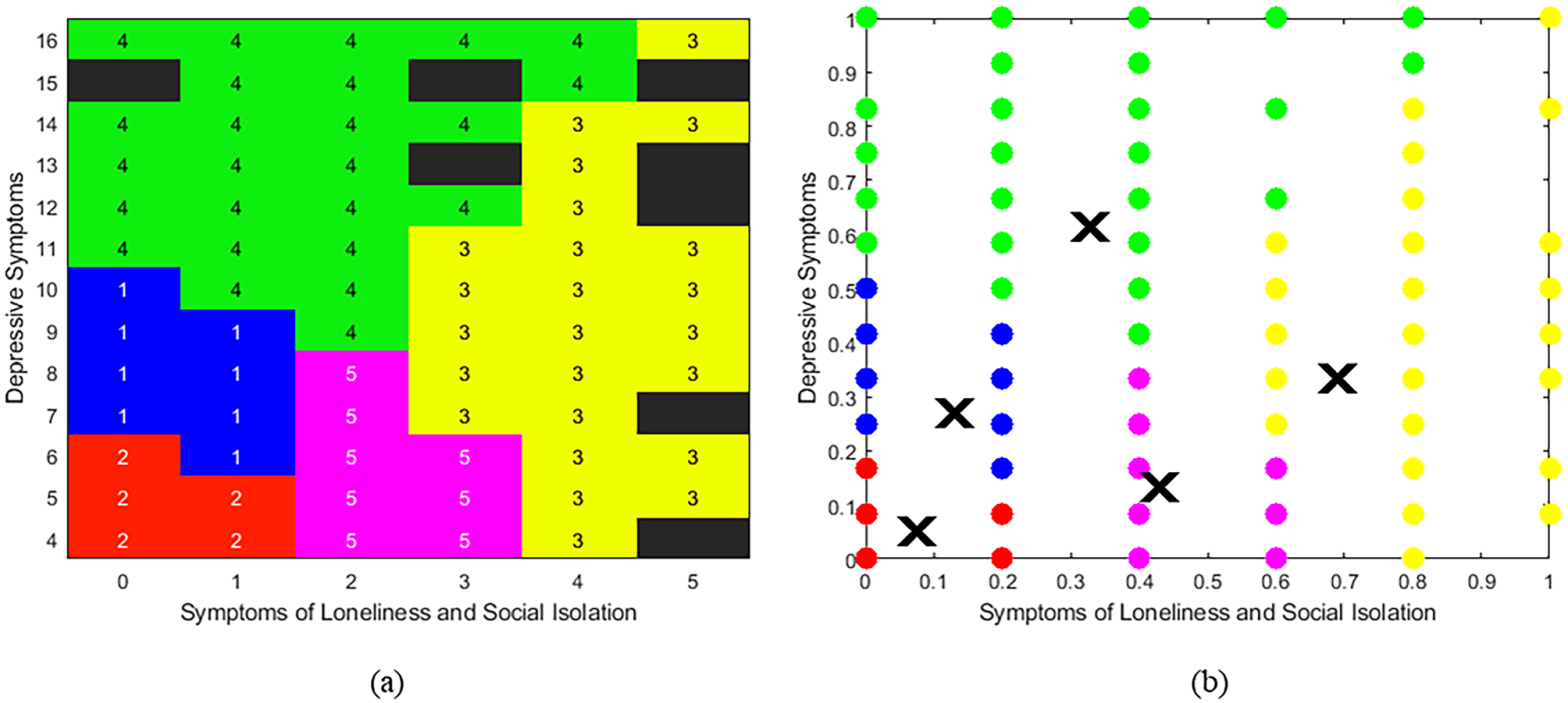
(**a**) Heat Map and (**b**) Scatter plot of the distribution of sub-groups of individuals with CBP based on symptoms of depression and loneliness/social isolation. Data are at discrete intervals due to outcomes being questionnaire based. Data is presented on (**a**) normal item range and (**b**) normalised scale of 0–1. Higher scores indicate greater symptoms of depression or loneliness/social isolation. Based on the centroids of fuzzy c-means clustering, classes and colours are (1; blue) low social isolation and loneliness and moderate depressive symptoms (n = 776; 18.7%), (2; red) low loneliness/social isolation and depressive symptoms (n = 2296; 55.3%), (3; yellow) high loneliness/social isolation and moderate depressive symptoms (n = 185; 4.5%), (4; green) moderate loneliness/social isolation and high depressive symptoms (n = 297; 7.2%) and (5; pink) moderate loneliness/social isolation and low depressive symptoms (n = 602; 14.5%). Black squares on the heat map indicate no class was available at those values. The X value on the scatter plot indicates the centroid of that cluster.

*Step 5:* Given the prior steps, five clusters were derived using symptoms of loneliness/social isolation and depression. Based on the centroids from fuzzy c-means clustering, five sub-groups consisted of: (1) low loneliness/social isolation and moderate depressive symptoms (n = 776; 18.7%); (2) low loneliness/social isolation and depressive symptoms (n = 2296; 55.3%); (3) high loneliness/social isolation and moderate depressive symptoms (n = 185; 4.5%); (4) moderate loneliness/social isolation and high depressive symptoms (n = 297; 7.2%); and (5) moderate loneliness/social isolation and low depressive symptoms (n = 603; 14.5%) (Fig. 2). Post-hoc evaluation showed a Silhouette Index of 0.589, indicating moderate similarity of a data point, on average, to its cluster (Supplementary Figure 4). In addition, within-cluster distances on a normalised 0–1 scale ranged from 0.11 to 0.21, while between cluster distances ranged from 0.23 (Clusters 1 and 2) to 0.68 (Clusters 2 and 3) (Supplementary Table 5). Cluster discrimination values ranged from -1.08 to − 0.22 (Supplementary Table 5).

### Sub-group classification

**Figure 3:**
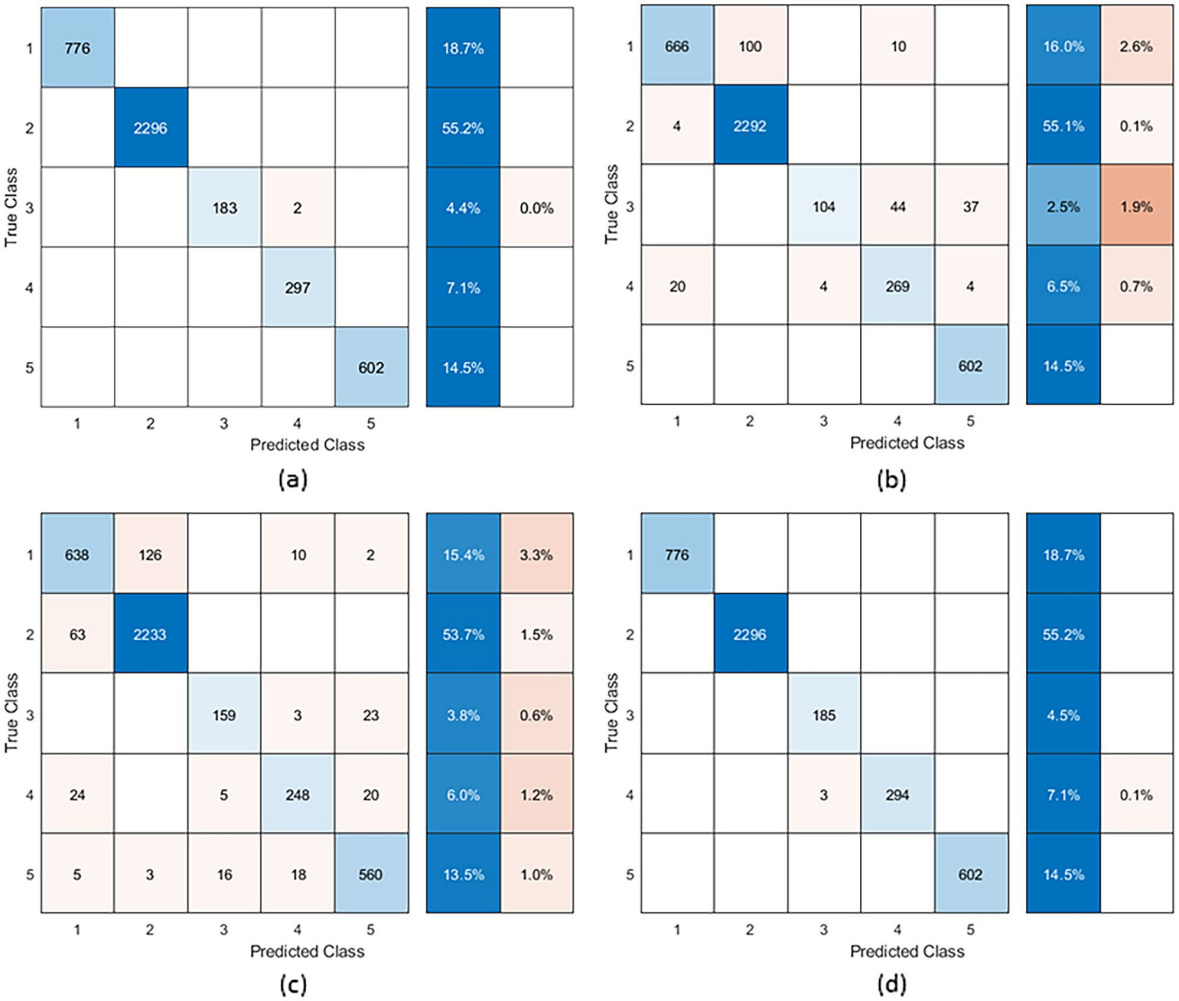
Confusion matrix of classifiers on chronic back pain classes (no pain-free controls included) with (**a**) Support Vector Machine, (**b**) Naïve Bayes, (**c**) k-Nearest Neighbour and (**d**) Random Forest classifiers. The x-axis is the predicted class while the y-axis is the true class. Blue squares indicate the number in the class that was accurately classified, while the oranges squares show the number of misclassifications. The boxes on the right of the matrix show the percentage of classification (blue) and misclassification (orange) for the class. Classes are (1) low social isolation and loneliness and moderate depressive symptoms (n = 776; 18.7%), (2) low loneliness/social isolation and depressive symptoms (n = 2296; 55.3%), (3) high loneliness/social isolation and moderate depressive symptoms (n = 185; 4.5%), (4) moderate loneliness/social isolation and high depressive symptoms (n = 297; 7.2%) and (5) moderate loneliness/social isolation and low depressive symptoms (n = 602; 14.5%).

**Figure 4:**
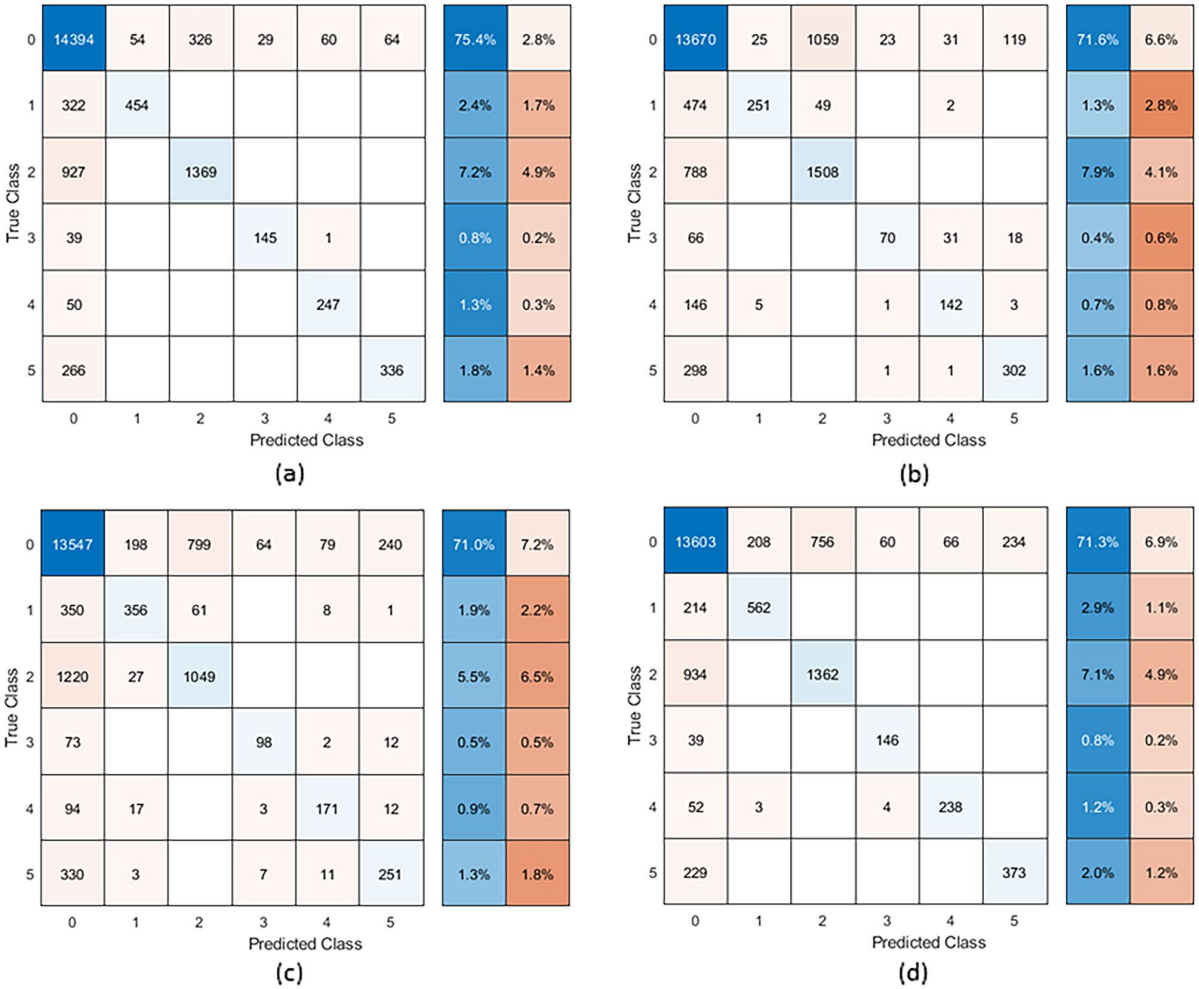
Confusion matrix of classifiers on chronic back pain and pain-free classes with (**a**) Support Vector Machine, (**b**) Naïve Bayes, (**c**) k-Nearest Neighbour and (**d**) Random Forest classifiers. The x-axis is the predicted class while the y-axis is the true class. Blue squares indicate the number in the class that was accurately classified, while the oranges squares show the number of misclassifications. The boxes on the right of the matrix show the percentage of classification (blue) and misclassification (orange) for the class. Classes are (0) pain-free individuals (n = 14,927; 78.2%), (1) low social isolation/loneliness and moderate depressive symptoms (n = 776; 4.1%), (2) low loneliness/social isolation and depressive symptoms (n = 2296; 12.0%), (3) high loneliness/social isolation and moderate depressive symptoms (n = 185; 1.0%), (4) moderate loneliness/social isolation and high depressive symptoms (n = 297; 1.6%) and (5) moderate loneliness/social isolation and low depressive symptoms (n = 602; 3.2%).

*Step 6:* Classifiers were first fit to CBP sub-groups using the seven variables determined important in feature weighing methods. The results showed misclassification error rates of < 1% for SVM (Fig. 3a), 5.3% for Naïve Bayes (Fig. 3b), 7.7% for kNN (Fig. 3c) and < 0.1% for RF classifiers (Fig. 3d), which would indicate accurate classification of CBP sub-groups. The range of area under the curve (AUC) for each class was 1.00–1.00, 0.99–1.00, 0.92–0.99 and 1.00–1.00 for SVM, Naïve Bayes, kNN and RF classifiers respectively (Supplementary Figure 5). Calibration curves are reported in Supplementary Figures 6–9 and calibration metrics are reported in Supplementary Table 6. Adding pain-free individuals into the dataset increased misclassification to 11.2% for SVM (Fig. 4a), 16.5% for Naïve Bayes (Fig. 4b), 18.9% for kNN (Fig. 4c) and 14.7% for RF classifiers (Fig. 4d). However, this misclassification was mostly impacted for the CBP sub-groups. For example, the misclassification error of CBP sub-groups from these classifiers was 38% for SVM, 45% for Naïve Bayes, 53% for kNN and 35% for Random Forest classifiers (Fig. 4). The range of area under the curve (AUC) for each class was 0.88–0.99, 0.86–0.99, 0.71–0.99 and 0.84–0.99 for SVM, Naïve Bayes, kNN and RF classifiers respectively (Supplementary Figure 10). Calibration curves are reported in Supplementary Figures 11–14 and calibration metrics are reported in Supplementary Table 6.

### Results of clustering and classification in each sub-domain

Step 7: Given the main clustering analysis was derived from available psychosocial factors, no further clustering analyses were completed in this sub-domain.

For physical measures of body mass index and grip strength, cluster validity methods showed that three sub-groups were optimal for this two-dimensional space (Supplementary Table 7). These three sub-groups consisted of: (1) higher body mass index and lower grip strength (n = 870; 20.9%); (2) lower body mass index and grip strength (n = 1933; 46.5%); and (3) lower body mass index and higher grip strength (n = 1353; 32.6%) (Supplementary Figure 15). There was moderate similarity of each data points to its cluster with a Silhouette value of 0.581 (Supplementary Figure 16). Within-cluster distances ranged from 0.09 to 0.12, while between cluster distances ranged from 0.23 to 0.27, while discrimination values ranged from − 0.33 to − 0.27 (SSupplementary Table 8). The results showed misclassification error rates of 1.1% for SVM, 5.5% for Naïve Bayes, 15.6% for kNN and 1.3% for RF classifiers, which indicated accurate classification of CBP sub-groups (Supplementary Figure 17). Adding pain-free individuals into the dataset increased misclassification to 10.8% for SVM, 15.6% for Naïve Bayes, 19.7% for kNN and 15.1% for RF (Supplementary Figure 18). AUC metrics were similar to the main analyses (Supplementary Figures 19 and 20). Calibration curves are reported in Supplementary Figures 21–28 and calibration metrics are reported in Supplementary Table 6.

For brain function, FPN-DMN connectivity (edge 1014), FPN-SMN connectivity (edge 222) and VN-DMN connectivity (edge 44) were ranked, in order of importance, using Laplacian scores (Supplementary Figure 29). Cluster validity methods indicated three clusters in two dimensions were optimal (Supplementary Table 9). Based on the centroids of fuzzy c-means clustering, sub-groups were: (1) higher FPN-DMN connectivity (n = 1,347; 32.4%); (2) higher FPN-SMN connectivity (n = 1335; 32.1%); and (3) lower FPN-DMN and FPN-SMN connectivity (n = 1,474; 35.5%) (Supplementary Figure 30). For the similarity of clusters, the Silhouette value was 0.497 (Supplementary Figure 31). The within-cluster distances ranged from 0.11 to 0.12, the between-cluster distances ranged from 0.23 to 0.25 and the discrimination values ranges from − 0.27 to − 0.24 (Supplementary Table 10). The results showed misclassification error rates of < 1% for SVM, 4.9% for Naïve Bayes, 11.1% for kNN and 2.1% for RF classifiers, which indicated accurate classification of CBP sub-groups (Supplementary Figure 32). Adding pain-free individuals into the dataset increased misclassification to 11.3% for SVM, 15.2% for Naïve Bayes, 20.2% for kNN and 15.6% for RF (Supplementary Figure 33). AUC metrics were similar to the main analyses (Supplementary Figures 34 and 35). Calibration curves are reported in Supplementary Figures 36–43 and calibration metrics are reported in Supplementary Table 6.

For brain structure, Laplacian scores to rank features are in Supplementary Figure 44. Two variables and two clusters were optimal for clustering (Supplementary Table 11). Results of clustered showed sub-groups of: (1) higher fronto-orbital and primary motor cortex sizes (n = 1917; 46.1%); and (2) lower fronto-orbital and primary motor cortex sizes (n = 2239; 53.9%) (Supplementary Figure 45). The similarity of data points to its cluster, based on the Silhouette value, was 0.589 (Supplementary Figure 46). The within-cluster distance ranged from 0.07 to 0.08, the between-cluster distances was 0.18 and the discrimination values was − 0.20 (Supplementary Table 12). The results showed misclassification error rates of < 1% for SVM, 5.6% for Naïve Bayes, 16.4% for kNN and 1.7% for RF classifiers, which indicated accurate classification of CBP sub-groups (Supplementary Figure 47). Adding pain-free individuals into the dataset increased misclassification to 10.7% for SVM, 17.4% for Naïve Bayes, 19.7% for kNN and 15.7% for RF (Supplementary Figure 48). AUC metrics were similar to the main analyses (Supplementary Figures 49 and 50). Calibration curves are reported in Supplementary Figures 51–58 and calibration metrics are reported in Supplementary Table 6.

### Between-group differences in variables across derived sub-groups

*Step 7:* For the five psychosocial sub-groups, results of a one-way ANOVA comparing CBP sub-groups to pain-free individuals are presented in Table 2. For depressive symptoms, CBP sub-groups one (mean difference [95%CI]: 2.3 [2.2, 2.5]pts, *p* < 0.001, ES = large), three (3.2 [2.9, 3.5]pts, *p* < 0.001, ES = large), four (5.2 [5.1, 5.4]pts, *p* < 0.001, ES = large) and five (0.7 [0.6, 0.9]pts, *p* < 0.001, ES = moderate) had higher levels of depressive symptoms than pain-free control, while sub-group two had lower levels of depressive symptoms (− 0.2 [− 0.3, − 0.1]pts, *p* < 0.001, ES = small). For loneliness/social isolation, sub-group two (− 0.34 [− 0.39, − 0.29]pts, *p* < 0.001, ES = small) had lower symptoms, while sub-groups three (2.73 [2.56, 2.90] pts, *p* < 0.001, ES = large), four (0.90 [0.76, 1.03]pts, *p* < 0.001, ES = moderate) and five (1.44 [1.34, 1.53]pts, *p* < 0.001, ES = large) had greater symptoms. No difference in loneliness/social isolation symptoms were observed between sub-group one and pain-free controls.

**Table 2:** Results of the Analysis of Covariance (ANCOVA) for sub-groups derived from psychosocial variables.

	Pain-free controls^a^	CBP sub-group 1^b^		CBP sub-group 2^c^		CBP sub-group 3^d^		CBP sub-group 4^e^		CBP sub-group 5^f^	
	Mean (SE)	Mean (SE) Mean diff (95 CI) ES (95 CI)	*p* value	Mean (SE) Mean diff (95 CI) ES (95 CI)	*p* value	Mean (SE) Mean diff (95 CI) ES (95 CI)	*p* value	Mean (SE) Mean diff (95 CI) ES (95 CI)	*p* value	Mean (SE) Mean diff (95 CI) ES (95 CI)	*p* value
Depressive symptoms (4–16)	4.9 (0.01)	7.2 (0.05)		4.6 (0.03)		8.0 (0.10)		11.3 (0.08)		5.6 (0.05)	
Versus PF		2.3 (2.2, 2.5) 1.87 (1.79, 1.94)	**< 0.001**	− 0.2 (− 0.3, − 0.1) − 0.24 (− 0.28, − 0.20)	**< 0.001**	3.2 (2.9, 3.5) 2.52 (2.39, 2.68)	**< 0.001**	6.4 (6.2, 6.6) 5.22 (5.10, 5.35)	**< 0.001**	0.7 (0.6, 0.9) 0.57 (0.49, 0.65)	**< 0.001**
Loneliness/isolation (0–5)	0.72 (0.01)	0.65 (0.03)		0.37 (0.02)		3.4 (0.06)		1.6 (0.05)		2.2 (0.03)	
Versus PF		− 0.06 (− 0.14, 0.02) − 0.06 (− 0.13, 0.01)	0.300	− 0.34 (− 0.39, − 0.29) − 0.29 (− 0.34, − 0.25)	**< 0.001**	2.73 (2.56, 2.90) 2.20 (2.05, 2.35)	**< 0.001**	0.90 (0.76, 1.03) 0.72 (0.61, 0.84)	**< 0.001**	1.44 (1.34, 1.53) 1.23 (1.14, 1.31)	**< 0.001**

Data are presented as adjusted mean (standard error). Adjusted mean differences and 95% confidence interval (95 CI) are reported. Effect sizes and 95% confidence intervals were calculated using standard formulae. All data were adjusted for age and sex. *p* value s were adjusted for multiple comparisons using Tukey HSD method.

*PF pain-free, 95 CI* 95% confidence interval, *ES *effect size.

Significant values are in [bold].

^a^Pain-free individuals (n = 14,927; 78.2%).

^b^CBP sub-group 1: low social isolation/loneliness and moderate depressive symptoms (n = 776; 4.1%).

^c^CBP sub-group 2: low loneliness/social isolation and depressive symptoms (n = 2296; 12.0%).

^d^CBP sub-group 3: high loneliness/social isolation and moderate depressive symptoms (n = 185; 1.0%).

^e^CBP sub-group 4: moderate loneliness/social isolation and high depressive symptoms (n = 297; 1.6%).

^f^CBP sub-group 5: moderate loneliness/social isolation and low depressive symptoms (n = 603; 3.2%).

For the three sub-groups derived from physical variables, results from ANCOVA are presented in Table 3. Sub-groups one (8.5 [8.1, 8.8]kg/m^2^, *p* < 0.001, ES = large) and three (0.7 [0.4, 1.0]kg/m^2^, *p* < 0.001, ES = very small) had a higher body mass index than pain-free controls, while sub-group two had a lower value (− 1.1 [− 1.3, − 0.8]kg/m^2^, *p* < 0.001, ES = small). Sub-groups one (− 2.9 [− 3.5, − 2.3]kg, *p* < 0.001, ES = small) and two (− 3.0 [− 3.4, − 2.6]kg, *p* < 0.001, ES = small) had lower grip strength than pain-free controls. Compared to pain-free controls, sub-group three had higher grip strength (4.8 [4.3, 5.3]kg, *p* < 0.001, ES = small).

**Table 3:** Results of the Analysis of Covariance (ANCOVA) sub-groups derived from physical variables of body mass index and grip strength.

	Pain-free controls^a^	CBP sub-group 1^b^		CBP sub-group 2^c^		CBP sub-group 3^d^	
	Mean (SE)	Mean (SE) Mean diff (95 CI) ES (95 CI)	*p* value	Mean (SE) Mean diff (95 CI) ES (95 CI)	*p* value	Mean (SE) Mean diff (95 CI) ES (95 CI)	*p* value
Body mass index (kg/m^2^)	25.9 (0.03)	34.4 (0.1)		24.8 (0.1)		26.6 (0.1)	
Versus PF		8.5 (8.1, 8.8) 2.34 (2.27, 2.41)	**< 0.001**	− 1.1 (− 1.3, − 0.8) − 0.29 (− 0.34, − 0.25)	**< 0.001**	0.7 (0.4, 1.0) 0.19 (0.14, 0.25)	**< 0.001**
Grip strength (kg)	31.9 (0.1)	29.0 (0.2)		28.9 (0.2)		36.8 (0.2)	
Versus PF		− 2.9 (− 3.5, − 2.3) − 0.24 (− 0.31, − 0.17)	**< 0.001**	− 3.0 (− 3.4, − 2.6) − 0.25 (− 0.30, − 0.21)	**< 0.001**	4.8 (4.3, 5.3) 0.41 (0.36, 0.47)	**< 0.001**

Data are presented as adjusted mean (standard error). Adjusted mean differences and 95% confidence interval (95 CI) are reported. Effect sizes and 95% confidence intervals were calculated using standard formulae. All data were adjusted for age and sex. *p* value s were adjusted for multiple comparisons using Tukey HSD method.

*PF* pain-free, *95 CI* 95% confidence interval, *ES* effect size.

Significant values are in [bold].

^a^Pain-free individuals (n = 14,927; 78.2%).

^b^CBP sub-group 1: higher body mass index and low grip strength (n = 870; 4.6%).

^c^CBP sub-group 2: lower body mass index and grip strength (n = 1933; 10.1%);

^d^CBP sub-group 3: lower body mass index and higher grip strength (n = 1353; 7.1%).

Between-group differences for sub-groups derived from brain variables are reported in Table 4. Results showed that for FPN-DMN connectivity (edge 1,014), sub-group one had higher connectivity (0.87 [0.81, 0.93]z, *p* < 0.001, ES = moderate), while sub-groups two (− 0.43 [− 0.49, − 0.37]z, *p* < 0.001, ES = small) and three (− 0.53 [− 0.59, − 0.47]z, *p* < 0.001, ES = small) had lower connectivity compared to pain-free controls. For FPN-SMN connectivity, sub-group one (− 0.13 [− 0.19, − 0.08]z, *p* < 0.001, ES = very small) and three (− 0.74 [− 0.79, − 0.68]z, *p* < 0.001, ES = moderate) had lower connectivity, while sub-group two (0.74 [0.68, 0.80]z, *p* < 0.001, ES = moderate) had higher connectivity compared to pain-free controls. While not used for eventual clustering, for VN-DMN connectivity, sub-groups one (0.09 [0.03, 0.16]z, *p* < 0.001, ES = very small) and sub-group three (0.09 [0.03, 0.15]z, *p* < 0.001, ES = very small) had higher connectivity than pain-free controls, while no statistically significant difference was observed for sub-group two (0.02 [− 0.05, 0.08]z, p = 0.927, ES = very small).

**Table 4:** Results of the Analysis of Covariance (ANCOVA) sub-groups derived from brain function variables.

	Pain-free controls^a^	CBP sub-group 1^b^		CBP sub-group 2^c^		CBP sub-group 3^d^	
	Mean (SE)	Mean (SE) Mean diff (95 CI) ES (95 CI)	*p* value	Mean (SE) Mean diff (95 CI) ES (95 CI)	*p* value	Mean (SE) Mean diff (95 CI) ES (95 CI)	*p* value
FPN-DMN connectivity (z)	1.33 (0.01)	2.20 (0.02)		0.91 (0.02)		0.81 (0.02)	
Versus PF		0.87 (0.81, 0.93) 0.73 (0.66, 0.80)	**< 0.001**	− 0.43 (− 0.49, − 0.37) − 0.35 (− 0.40, − 0.31)	**< 0.001**	− 0.53 (− 0.59, − 0.47) − 0.44 (− 0.49, − 0.38)	**< 0.001**
FPN-SMN connectivity (z)	0.70 (0.01)	0.56 (0.02)		1.44 (0.02)		− 0.04 (0.02)	
Versus PF		− 0.13 (− 0.19, − 0.08) − 0.12 (− 0.19, − 0.05)	**< 0.001**	0.74 (0.68, 0.80) 0.62 (0.58, 0.67)	**< 0.001**	− 0.74 (− 0.79, − 0.68) − 0.62 (− 0.68, − 0.57)	**< 0.001**
VN-DMN connectivity (z)	0.40 (0.01)	0.49 (0.02)		0.41 (0.02)		0.49 (0.02)	
Versus PF		0.09 (0.03, 0.16) 0.08 (0.02, 0.13)	**< 0.001**	0.02 (− 0.05, 0.08) 0.01 (− 0.15, 0.06)	0.927	0.09 (0.03, 0.15) 0.08 (0.02, 0.13)	**< 0.001**

Data are presented as adjusted mean (standard error). Adjusted mean differences and 95% confidence interval (95 CI) are reported. Effect sizes and 95% confidence intervals were calculated using standard formulae. All data were adjusted for age and sex. *p* value s were adjusted for multiple comparisons using Tukey HSD method.

*PF* pain-free, *95 CI* 95% confidence interval, *ES* effect size, *FPN* frontoparietal network, *DMN* default mode network, *SMN* sensorimotor network, *VN*  visual network.

Significant values are in [bold].

^a^Pain-free individuals (n = 14,927; 78.2%).

^b^CBP sub-group 1: high FPN-DMN connectivity (n = 1347; 7.1%).

^c^CBP sub-group 2: high FPN-SMN connectivity (n = 1335; 7.0%);

^d^CBP sub-group 3: low FPN-DMN and FPN-SMN network connectivity (n = 1474; 7.7%).

Differences between-groups derived from brain structure are presented in Table 5. Fronto-orbital cortex volume was higher in sub-group one (765.3 [692.0, 838.7]mm^3^, *p* < 0.001, ES = moderate) and lower in sub-group two (− 799.4 [− 867.7, − 731.2]mm^3^, *p* < 0.001, ES = moderate) compared to pain-free controls. Primary motor cortex volume was also higher in sub-group one (1,486.1 [1,322.3, 1,649.8]mm^3^, *p* < 0.001, ES = moderate) and lower (− 3188.0 [− 3400.5, − 2975.6]mm^3^, *p* < 0.001, ES = moderate) in sub-group two compared to pain-free controls. Lastly, even though it was not used for deriving sub-groups, primary somatosensory volume was higher in sub-group (743.1 [601.0, 885.2]mm^3^, *p* < 0.001, ES = small) one and lower in sub-group two (− 1154.3 [− 1286.5, − 1022.0]mm^3^, *p* < 0.001, ES = small) compared to pain-free controls.

**Table 5:** Results of the Analysis of Covariance (ANCOVA) sub-groups derived from brain structure variables.

	Pain-free controls^a^	CBP sub-group 1^b^		CBP sub-group 2^c^	
	Mean (SE)	Mean (SE) Mean diff (95 CI) ES (95 CI)	*p* value	Mean (SE) Mean diff (95 CI) ES (95 CI)	*p* value
Fronto-orbital cortex volume (mm^3^)	12,635.6 (10.4)	13,400.9 (29.5)		11,836.2 (27.2)	
Versus PF		765.3 (692.0, 838.7) 0.60 (0.55, 0.65)	**< 0.001**	− 799.4 (− 867.7, − 731.2) − 0.63 (− 0.67, − 0.58)	**< 0.001**
Primary motor cortex volume (mm^3^)	27,515.4 (23.3)	29,001.5 (65.9)		25,813.4 (60.7)	
Versus PF		1486.1 (1322.3, 1649.8) 0.52 (0.47, 0.57)	**< 0.001**	− 3188.0 (− 3400.5, − 2975.6) − 0.60 (− 0.64, − 0.55)	**< 0.001**
Primary somatosensory cortex volume (mm^3^)	21,706.0 (20.2)	22,449.1 (57.2)		20,551.8 (52.7)	
Versus PF		743.1 (601.0, 885.2) 0.30 (0.25, 0.35)	**< 0.001**	− 1154.3 (− 1286.5, − 1022.0) − 0.47 (− 0.51, − 0.42)	**< 0.001**

Data are presented as adjusted mean (standard error). Adjusted mean differences and 95% confidence interval (95 CI) are reported. Effect sizes and 95% confidence intervals were calculated using standard formulae. All data were adjusted for age and sex. *p* value s were adjusted for multiple comparisons using Tukey HSD method.

*PF* pain-free, *95 CI* 95% confidence interval, *ES* effect size.

Significant values are in [bold].

^a^Pain-free individuals (n = 14,927; 78.2%).

^b^CBP sub-group 1: high fronto-orbital and primary motor cortex volumes (n = 1917; 10.1%);

^c^CBP sub-group 2: low fronto-orbital and primary motor cortex volumes (n = 2239; 11.7%).

### Clustering and classification validation

*Step 8:* Feature ranking and clustering was completed in training set then applied to the test data set. Similar feature weight and clustering performances were observed across training and test data sets (Supplementary Figures 59–61; Supplementary Table 12). The Silhouette value for the training and test sets were 0.65 and 0.63, respectively (Supplementary Figures 62 and 63). The within-cluster distances ranged from 0.054 to 0.217 in the training data and 0.054 to 0.243 in the test data (Supplementary Tables 14 and 15). The between cluster distances ranged from 0.200 to 0.699 in the training data and 0.200 to 0.716 in the test data (Supplementary Tables 14 and 15). The discrimination values ranged from − 0.29 to − 1.16 in the training data clusters and − 0.29 to − 1.14 in the test set clusters (Supplementary Tables 14 and 15).

For classification, the classification accuracy (range of AUC) for the validation set for SVM, Naïve Bayes, kNN and RF classifiers were > 99% (1.00–1.00), 63.6% (0.58–0.99), 92.3% (0.95–0.99) and > 99% (1.00–1.00), respectively (Supplementary Table 16; Supplementary Figures 64 and 65). For the test data the results SVM, Naïve Bayes, kNN and RF classifiers were 97.9% (0.99–1.00), 62.9% (0.56–0.99), 89.9% (0.94–0.98) and 98.4% (0.99–1.00) (Supplementary Table 16; Supplementary Figures 66 and 67). Outside of Naïve Bayes classification, this shows similar clustering a classification results to the main analyses (Supplementary Table 16; Supplementary Figures 64 and 66). Calibration curves are reported in Supplementary Figures 68–71 and calibration metrics are reported in Supplementary Table 6.

The results of prior probably adjusted classification models are in Supplementary Table 17. For classifiers testing CBP individuals, no major differences were observed between original and prior probability adjusted models. For models on CBP versus pain-free individuals, SVM and Naïve Bayes overall classification accuracy remained stable across all analyses. However, when looking at different classes, there was greater misclassification of pain-free controls and increased accuracy of classification for CBP sub-groups to maintain this result. Classification accuracy dropped for kNN and RF classifiers for CBP versus pain-free controls, particularly due to the increased misclassification of pain-free controls.

## Discussion

Our primary results showed that there are sub-groups of individuals with CBP based on psychosocial factors, yet not brain and the limited physical variables examined. These groups consisted of: (1) low loneliness/social isolation and moderate depressive symptoms; (2) low loneliness/social isolation and depressive symptoms; (3) high loneliness/social isolation and moderate depressive symptoms; (4) moderate loneliness/social isolation and high depressive symptoms; and (5) moderate loneliness/social isolation and low depressive symptoms. Furthermore, these groups could be accurately classified using ML approaches when people with CBP were evaluated alone. When pain-free controls were examined as well, the error rate in the models increased, particularly in CBP sub-groups. We interpret the latter finding to indicate that these variables alone are insufficient for predicting who has CBP yet are likely necessary in determining sub-group classification of patients with known CBP.

We have previously showed using UKBioBank data that differences in brain structure, psychosocial, and physical health exist depending on if back pain is acute or chronic and localised or widespread^15^. However, identifying sub-groups of individuals with back pain has been a goal of clinicians and researchers to improve diagnosis and treatment^16^, with the aim of improved patient outcomes and reduced financial costs at both the individual and societal level. To build on prior ML research that only used binary classification of back pain versus no-back pain^13^, our results showed that individuals with CBP could be sub-grouped based on psychosocial factors of symptoms of depression and loneliness/social isolation and attempt classification on these new classes. These results are supported by Backyrd et al.^17^, who included psychosocial measures only and found four sub-groups of individuals with chronic pain based on hierarchical clustering methods characterised by low and high levels of psychological and social distress. Given back pain is a biopsychosocial issue^11^, we attempted to include additional brain-related and physical variables in our analyses to derive sub-groups. Even though these differed between CBP and pain-free controls, they were not important for deriving subgroups based on feature weighting and cluster validity methods for the main analyses. However, as shown by our secondary analyses, physical and brain measures may still be important for deriving sub-groups of CBP. Importantly, even though psychosocial variables are often treated as continuous in analyses, they have results at discrete intervals on a measurement scale (e.g. a numeric rating scale of 0–10)^18^. Given feature weighting and cluster validity methods are based on distances within- and between-groups^19,20^, this may mean psychosocial factors are the most important solely due to method of measurement and the potential for greater distances on normalised data scales within- and between-groups. Therefore, future research should: (1) derive sub-groups based on homogenous outcomes across multidimensional domains linked to CBP, and (2) develop methods to account for differences in measurement scales in feature weighting and cluster validity methods.

The findings from the current study showed that individuals with CBP can be accurately into all the subgroups derived. However, the addition of pain-free controls increased the misclassification of CBP individuals to greater than 35–53% for CBP groups in the main analyses. Prior research has showed sub-groups in chronic pain based on psychosocial distress^17^, while research in low back pain had showed clusters based on sensory testing^21^ and spinal tissue profiles^9^. However, only one of these studies included pain-free controls for comparison^21^. For example, prior work in acute low back pain showed that sub-groups may exist based on pressure-pain thresholds and conditioned pain modulation, with differences observed in ANCOVA compared to pain-free controls in all but one cluster (n = 31/125; 25%)^21^. In a study without pain-free controls, which assessed sub-groups of low back pain based on MRI morphological findings, a cluster containing 51.6% of 3155 vertebral motion-segments consisted of no or minimal (< 5%) MRI morphological findings^9^. Even though no ML techniques were used in either study^9,21^, it seems the inclusion of a pain-free control arm is paramount for developing meaningful back pain sub-groups. However, in our study, pain-free and back pain status was self-report and therefore it is unclear if pain-free individuals had prior pain. Based on our findings, future research should include a pain-free control arm to identify variables which produce less overlap to create more meaningful CBP sub-groups.

When examining sub-groups based on physical variables of body mass index and grip strength, we showed that three sub-groups may exist. A systematic review showed no statistically significant relationship on developing chronic low back pain in those with a body mass index above ≥ 25 kg/m^2^^22^. However, a prior longitudinal study showed that those with obesity (body mass index ≥ 30 kg/m^2^) were 30% more likely to develop low back pain compared to those with a body mass index < 25 kg/m^2^^23^. Therefore, the importance of body mass index may be in those with CBP who are obese (e.g. physical sub-group one). For grip strength, muscular strength is not considered to be a risk factor for back pain^24^, however, may be considered a consequence of deconditioning^25^. In our analyses we showed physical sub-groups two and three have lower grip strength compared to pain-free controls. Therefore, treatment orientated at restoring muscular conditioning may be reserved for sub-groups two and three. Notably, we were unable to assess other key physical parameters, such as intervertebral disc health^26^, as these measures were not available in the UKBioBank dataset. Clusters of CBP based on body mass index and grip strength exist, however, follow-up with other key physical parameters, such as spine imaging, is required.

For sub-groups based on resting state brain function, we showed that clusters may exist for connectivity between frontoparietal, default mode and sensorimotor networks in those with CBP. These function of these brain networks, while linked, differ; the frontoparietal network is linked to cognitive control of behaviour in a goal-driven manner^27^; the default mode network is involved in memory retrieval and internal cognitions, while deactivating during attention demanding external tasks^28^, and; the sensorimotor network is involved in sensory processing, and could be involved in encoding the location and intensity of pain^29^. Previous studies have examined connectivity differences in the regions between individuals with chronic pain and pain-free controls^30–33^, yet none have attempted sub-grouping. In our analyses, we showed that sub-group one had greater FPN-DMN connectivity compared to pain-free controls and could indicate impairments in the interaction between memory, internal cognitions, goal-driven behaviour and pain. This may explain why some individuals with CBP have impaired movements in the presence of fear and catastrophising^34^. Sub-group two had higher connectivity in the FPN-SN, while sub-group three had lower connectivity compared to pain-free controls. This could indicate impairments in connectivity between goal-driven behaviours^27^ and sensory processing of pain^29^. This may be important for sub-group two given increases in sensorimotor network activity have been shown to have a moderate strength correlation with higher levels of back pain^30^. However, for both FPN-DMN and FPN-SN connectivity, we were unable to explore the association between clinical outcomes (pain, fear and catastrophising) in our study due to outcome being unavailable in the UKBioBank. Overall, these results indicate that sub-groups of CBP may have impairments in frontoparietal, default mode and sensorimotor networks. Regardless, more research is required to determine the clinical meaningfulness of these findings.

For sub-groups based on brain structure, we derived two clusters based on the fronto-orbital and primary motor cortex grey matter volumes. The fronto-orbital cortex is linked to cognitive/emotional processing of pain, while the primary motor cortex to motor aspects^35^. In addition to this, the primary somatosensory cortex grey matter volume differentiated CBP individuals and pain-free controls and was a candidate for clustering. The primary somatosensory cortex has been previously linked to somatosensory aspects of pain encoding^35^. When assessing the derived sub-groups, sub-group one had higher values in all three brain grey matter volumes, while sub-group two had lower values. For sub-group two, this could indicate impairments in motor, sensory and cognitive/emotional areas related to pain^35^. Furthermore, given that all brain grey matter volumes were lower, this may indicate impairments with central processing of pain in this sub-group^36^. However, given we did not assess if the pain was localised or widespread in this study, this should be considered in future research to further identify important brain markers across back pain individuals. Differences in brain grey matter volumes also need to be related to clinical outcomes to determine if they are clinically relevant for treatment. Our study showed that clusters may exist in brain structure in individuals with CBP and should be further explored to determine clinically relevant CBP sub-groups.

It is appropriate to consider the potential treatment implications of the sub-groups identified. A biopsychosocial approach is promoted for the treatment of back pain^11^ and our study indicates that clinicians could consider differing psychosocial, physical and brain outcomes in individuals they treat for back pain. An approach to integrate this into clinical practice could be to individualise assessment based on findings from subjective assessment^11^ or use physical/psychosocial screening tools such as the STarTBack tool^37^ and Orebro Musculoskeletal Questionnaire to identify psychosocial aspects of pain through question responses^38^. However, even though we could not assess this in our study, prior research has showed that other sensory testing^21^ and spinal tissue^9^ factors could also be used to sub-group back pain individuals. Even though some back pain sub-groups may not be clearly separated from pain-free controls, until it is clear which variables best segregate these groups, they should continue to be considered clinically to truly adopt a biopsychosocial approach to treatment. Using such an approach, of targeting psychosocial factors with treatment, may improve patient centred care by identifying and targeting more individualised factors in the treatment of back pain conditions.

The current study had multiple strengths. Firstly, this was the first study to develop and classify sub-groups of CBP based on psychosocial, physical and nervous system measures using data-analytic approaches^13^. Secondly, we had 19,083 participants that could be used to help identify sub-groups of individuals with CBP. Furthermore, we followed up clustering methods with post-hoc tests and relevant adjustments to ensure more robust sub-groups that consider covariates of important variables. For limitations, firstly, there was a lack of spinal tissue measures, which could be important for sub-grouping some individuals with CBP^8–10^. Even though spinal tissues outcomes do not always relate to pain, they are a source of nociception^39^ and need consideration in future studies for a more informative approach to sub-classification^40^. Secondly, clinical outcomes, such as pain intensity, disability and pain duration, were not available in the UKBioBank. It would be beneficial to relate the sub-groups detected to these clinical characteristics. However, validation of our classification models with the inclusion of pain-free controls suggests future research should determine which variables best discriminate cases from controls for more robust subgroups, regardless of additional pain-related variables. Furthermore, limited psychological variables that can modulate pain and disability were available in the UKBioBank. Future research using classification methods should consider additional psychological variables such as anxiety and pain catastrophizing. Finally, the cross-sectional nature of this study precludes conclusions regarding causality. Future research should use prospective data to determine how sub-groups transition through the stages of back pain.

Based on our findings and limitations, future studies should consider which variables they use in design and implementation of attempting accurate sub-classification of back pain individuals with ML approaches. In particular, pain processing mechanisms^7,21^ and changes in spinal tissues^8–10^ could be used in addition to psychosocial factors to identify relevant back pain sub-groups. This has the potential to reduce overlap and misclassification in the data; hence, creating more robust sub-groups. Furthermore, studies should investigate combining different outcomes, while also completing sub-domain analyses separately, to better understand how to combine variables in data-analytic method. In addition, future studies should consider relating clinical outcomes (e.g. pain intensity and disability) to new sub-groups. Understanding the relationship between sub-groups and important clinical measures may allow for improved knowledge on how to target outcomes for individuals with back pain.

This study was the first to use ML algorithms to attempt to divide individuals with CBP into sub-groups based on psychosocial, brain and physical factors. For the main analysis, optimal sub-grouping was determined to require only two psychosocial variables. Secondary analyses indicate that sub-groups based on physical, brain structure and function can also be derived in sub-domains analyses. Misclassification error may indicate that variables used in this study were not optimal to differentiate these sub-groups from pain-free controls. Future research should optimise variables via including spinal tissue (e.g. intervertebral disc height), sensory testing (e.g. pressure-pain thresholds) and additional psychosocial variables (e.g. self-efficacy) to create more robust CBP sub-groups that discriminate them from pain-free controls to optimise targeted treatments for the condition.

## Methods

This was a cross-sectional study of the UKBioBank imaging visit (Project ID: 55843). Following the first wave of recruitment between 2006 and 2010, a subset of participants was invited for initial imaging analyses between 2014 and 2018. Participants provided written informed consent prior to data collection. For this sub-study, we used a sample of individuals with chronic back pain and those who were pain-free. The UKBioBank was ethically approved by the NHS National Research Ethics Service (dated 17th June 2011, ref. 11/NW/0382) and all experiments were performed in line with relevant guidelines and regulations. The current project was approved by Deakin University Human Research Ethics Committee (project ID: 2020–323).

### Inclusion and exclusion criteria

The UKBioBank initially recruited adults from the United Kingdom between the ages of 40–69 who were able, and consented to, attending an assessment centre for testing. For inclusion, self-report questions available in the UkBioBank (https://biobank.ndph.ox.ac.uk/showcase/label.cgi?id=100048) were used to determine pain status. Information on the specific questions can be found here: https://biobank.ctsu.ox.ac.uk/crystal/crystal/docs/TouchscreenQuestionsMainFinal.pdf (search SY5). The following groups were included for this sub-study: (a) pain-free, in which participants reported ‘none of the above’ to pain experienced over the month prior; and (b) CBP: participants reporting ‘yes’ to back pain lasting greater than three months. Participants without full neuroimaging datasets were excluded from the sample. For magnetic resonance imaging, individuals with metal implants, such as a pacemaker, were excluded.

### Data collection

We selected and extracted key variables available in the UKBioBank which have previously shown to be related to CBP. For brain imaging, meta-analyses and systematic reviews show both of structural^41^ and functional magnetic resonance imaging (MRI)^42^ measures may be important for individuals with CBP. Psychosocial factors including mental and social health^43^, and physical factors of body mass index^22^ and muscular strength^44^ have also been shown to be associated with CBP.

#### Neuroimaging

A 3.0-T MRI imaging machine (Siemens Skyra, Siemens Healthcare, GER) with a 32-channel receiver head coil was used to scan individuals. FSL packages (version 5.0; FMRIB software library, Oxford, England; http://fsl.fmrib.ox.ac.uk/fsl) was used for image pre-processing. Protocols for detailed imaging methods are available on the UKBioBank website (http://biobank.ctsu.ox.ac.uk/crystal/refer.cgi?id=2367; http://biobank.ctsu.ox.ac.uk/crystal/refer.cgi?id=1977). For analyses here, we used volumetric and functional brain analyses.

For functional brain analyses, we used partial correlation matrices in the 100-dimension from independent component analysis to assess 1,485 direct connections between sub-regions of functional brain nodes at varying anatomical labels. For important connections in our analyses, we used the Automated Anatomical Labelling (AAL) atlas through MRICron (https://www.nitrc.org/projects/mricron) to define the location of the peak activations of each node and determine the anatomical location of the connectives^45^. To further label the anatomical locations of each node to a corresponding functional network (such as the default mode network), we used 7-network parcellation^46^. Brain coordinates, anatomical locations and corresponding functional networks of connectivity edges used in data-analytic approaches are in Supplementary Table 1.

For structural brain analyses, we used regional grey matter volumes linked to pain processing^47^. The amygdala, thalamus, insula, medial frontal cortex, anterior cingulate cortex, caudate, putamen, hippocampus, precentral gyrus (primary motor cortex), supplementary motor cortex, post-central gyrus (primary somatosensory cortex) and parietal operculum (secondary somatosensory cortex) were used to determine how these brain volumes differed between groups^47^. Data from left- and right-hand sides were pooled for the analyses.

#### Mental and social health

We used measures of current depression and loneliness/social isolation symptoms as these were available psychosocial measures in the UKBioBank dataset linked to CBP^43^. Both measures were collected via questionnaires on a touch screen. Four questions were used to determine current symptoms of depression. From the validated Patient Health Questionnaire, questions assessed frequency of depressed mood, disinterest/unenthusiasm, tenseness/restlessness and tiredness/lethargy over the prior 2 weeks^48^. For each question, a four-point scale, ranging from not at all, several days, more than half the time to almost every day was set. The total score was summed from four to 16, with higher scores indicating a greater level of current depressive symptoms^49^. As reported in previous UKBioBank studies, symptoms of social isolation/loneliness were also assessed in the current study^15,50^. Social isolation consisted of three questions: (1) “Including yourself, how many people are living together in your household?” (2) “How often do you visit friends or family or have them visit you?” and (3) “Which of the following (leisure/social activities) do you engage in once a week or more often? You may select more than one.” Loneliness was assessed with two questions: (1) “Do you often feel lonely?” and (2) “How often are you able to confide in someone close to you?” The scale was scored from 0 to 5, where individuals with higher scores were deemed to have greater symptoms of social isolation and loneliness^50^.

#### Muscular strength

A Jamar J00105 Hydraulic Hand Dynamometer (Lafayette Instruments, IN, USA) measured grip strength for both right- and left-hand sides. The participant was instructed to have their arm by their side with the elbow at 90 degrees, and the wrist in a neutral position (thumb to the ceiling). The participant squeezed the dynamometer for three seconds with encouragement to produce as much force as possible. For both right- and left-hand sides, the maximum grip strength was measured in kilograms of force. The highest grip strength achieved on either the left- or right-hand side was used for analysis.

#### Anthropometrics

Body mass index was used as a measure of adiposity. Height in centimetres was measured using Seca 202 stadiometer (Seca GMBH & Co, Hamburg, GER). Weight in kilograms was measured using a Tanita BC-418 (Tanita, Tokyo, JAP). Body mass index was then calculated as kg/m^2^.

### Sampling, statistical and data analytic methods

#### Sampling and variable selection

*Step 1:* Initial data preparation was completed in StataMP version 15 (StataCorp, Texas, USA) to allow for a reduced working file size for subsequent analyses. Individuals who did not complete the first brain imaging visit (instance 2) were removed from the dataset. Following this, the sample was matched one-to-three back pain-to-pain-free control on age and sex using a custom code in software package R version 3.5.1 (www.r-project.org), while preferentially matching for cases without missing data if possible. We selected three controls for matching, given little is gained by including more^51^. Following matching, 20,352 individuals remained (pain-free controls, n = 15,284; CBP, n = 5068), with 19,083 having full datasets for ML analysis (pain-free controls, n = 14,927; CBP, n = 4156) (Fig. 1). Further, statistical analyses were conducted using StataSE version 16 (StataCorp, Texas, USA). Independent t-tests were used to determine between-group differences and the order of importance of variables in the dataset with all variables treated as continuous. Given 1502 variables existed, with an initial *p* value set at 0.05, adjustment for false positives across each t-test was completed using the Benjamini–Hochberg false discovery rate method^52^. Variables which remained significant following *p* value adjustment were retained and multicollinearity was assessed with a correlation matrix using a threshold of r > 0.8^53^. Variables which passed both false discovery rate and multicollinearity were used in subsequent data-analytic methods.

#### Data-analytic methods

We conducted the data-analytic methods using software package MATLAB version R2020a (MathWorks, Massachusetts, USA) to determine sub-groups of CBP. Further details on the data-analytic methods used below are available in the Supplementary Methods 1.

*Step 2:* Feature weighting was completed to determine variables which best distinguish CBP from pain-free controls to create the most robust CBP sub-groups. Treating variables as continuous, data was first normalised on a 0–1 scale to allow comparability of different variables and different scales of values. Variable weighting methods of (a) minimum redundancy maximum relevance (MRMR) and (b) a random forest (RF) predictor was compared to determine important features. Following this, pain-free controls were removed from the dataset, to explore clustering of CBP individuals.

*Step 3:* Laplacian scores were used to rank remaining variables in the CBP space in order of importance. The dataspace for clustering was initially explored with two variables and were added into the dataspace in order of importance to determine where the breakpoint of random noise being added to the models.

*Step 4:* To determine the optimal number of variables and clusters in each dataspace, the noise breakpoint was explored using Calinski-Harabasz, Davies-Bouldin and Silhouette cluster evaluation methods with k-means linkage^54^, as well as fuzzy c-means clustering using entropy values^55–57^.

*Step 5:* Following this, new sub-groups of CBP were labelled based on the membership values to each cluster from fuzzy c-means clustering. To evaluate the post-hoc similarity of each data point, on average, to its derived clusters, we used Silhouette Index values to determine the goodness of fit (overall tightness and separation). In addition, we evaluated the average within- and between-cluster distances using the centroids of each cluster. From the average within- and between-cluster distances, we also calculated the discrimination value between clusters as reported here^58^. Given the potential for differences in effect sizes/variance in outcomes across domains due to the way they are measured, even when they are all scaled to 0–1, we also explored clustering for variables which passed prior steps in each sub-domain of psychosocial, physical, brain function and brain structure separately^59^.

*Step 6:* Retaining all variables which passed FDR and feature weighting, Support Vector Machine (SVM), Naïve Bayes, k-Nearest Neighbour (kNN) and RF one-vs-one multi-class classifiers were fit to the new CBP sub-groups to determine how accurately they could be classified. To cross-validate the model, a portion of the training set (validation set) was used through tenfold cross-validation (9 parts of the data set were used for training and one part for validation, repeated each time reserving a different tenth of the data for validation). Pain-free controls were readded into the dataset and classifiers were trained and validated again to determine if classification accuracy changed. The reason for completing classification with the addition of pain-free controls was to determine if and what sub-groups were separable from pain-free controls. There is contention surrounding the most important pain-related features, therefore, determining which features separate CBP from pain-free controls is important to guide future research to find specific treatable phenotypes of the condition^59^. Furthermore, we attempted classification on the sub-groups derived from the main and sub-domain clustering analyses. Area under the received operating curve was calculated for every class following each classifier. Reliability diagrams (calibration curves), histograms of predicted probabilities and calibration metrics (Brier score, logistic loss and expected calibration error) were also determined for each model and class in binary one-vs-rest approach^60^.

#### Between sub-group and pain-free control statistical analyses

*Step 7:* To further determine where statistical significance existed between each derived sub-group and pain-free controls, an analysis of covariance (ANCOVA) was used on remaining variables using StataSE version 16 (StataCorp, Texas, USA). The ANCOVA models were adjusted for age and sex (to account for any remaining variability following matching). *p* values were adjusted for multiple comparisons using the Tukey HSD method. An alpha level was set to 0.05. Standard formula were used to calculate effect sizes (Cohen’s d; ES) and relevant confidence intervals between CBP sub-groups and pain-free controls^61^. Effect sizes were considered small from 0.2–0.49, moderate from 0.5–0.79 and large from 0.8 and above^62^.

#### Clustering and classification validation

*Step 8:* To further validate the result from our clustering and classification, the processing pipeline was completed in CBP individuals again by splitting the data 50/50 into training and test sets. Firstly, we analysed Laplacian scores in the training set to rank features in order of explaining the most variance. Following this cluster validity methods were repeated in the training set by adding variables in order of Laplacian scores to determine the optimal number of clusters and variables. After this, fuzzy c-means clustering was applied to the training set only. Fuzzy c-means clustering was then applied to the test set ‘out-of-sample’ to determine if clustering performance was similar to the training set. Cluster centroids, within- and between-cluster distances, Silhouette index and discrimination values were compared between clustering in the training and test sets. The classifiers used in Step 6 were then repeated with only the training data being used to train the model. To cross-validate the model, a portion of the training set (validation set) was used through tenfold cross-validation. Following this, the trained classification model was applied to the test set and the accuracy and areas under the receiver operating characteristics curve compared to that of the training validation set. Furthermore, to explore the effects of class imbalance on all of our classification models, we balanced the models class prior probabilities uniformly to determine if this altered the classification results^63^.

### Ethics approval

The UKBioBank was approved ethically by the NHS National Research Ethics Service (dated 17th June 2011, ref. 11/NW/0382), with ethical exemption granted by the Deakin University Human Research Ethics Committee (project ID: 2020-323) for this project. All experiments were performed in line with relevant guidelines and regulations.

## Supplementary Information

Supplementary Information.

## Data Availability

Publicly available UKBioBank data was analysed in our study. Researchers can access datasets through open registration (see: https://www.ukbiobank.ac.uk/register-apply/).
