# Supplementary material for: Chronic back pain sub-grouped via psychosocial, brain and physical factors using machine learning
Source: Sci Rep. 2022 Sep 7;12:15194. doi: 10.1038/s41598-022-19542-5 (PMC9452567; doi:10.1038/s41598-022-19542-5)
Supplement: Supplementary file 1 — Supplementary Information. [file 41598_2022_19542_MOESM1_ESM.docx]

**SUPPLEMENTARY METHODS 1**

*Laplacian Scores*

The Laplacian function is a feature ranking method used in unsupervised learning.^1^ The Laplacian scores are computed with the following steps: (1) for each datapoint, determine local neighbours using the nearest neighbour method and find pairwise distances between neighbourhoods, (2) using kernel transformation to convert distances to similarity matrix, (3) centre the feature by removing its mean and (4) compute the scores for each feature, with higher values indicating greater feature importance. Further details are available here: <https://au.mathworks.com/help/stats/fsulaplacian.html>. The following equation provides the Laplacian score:


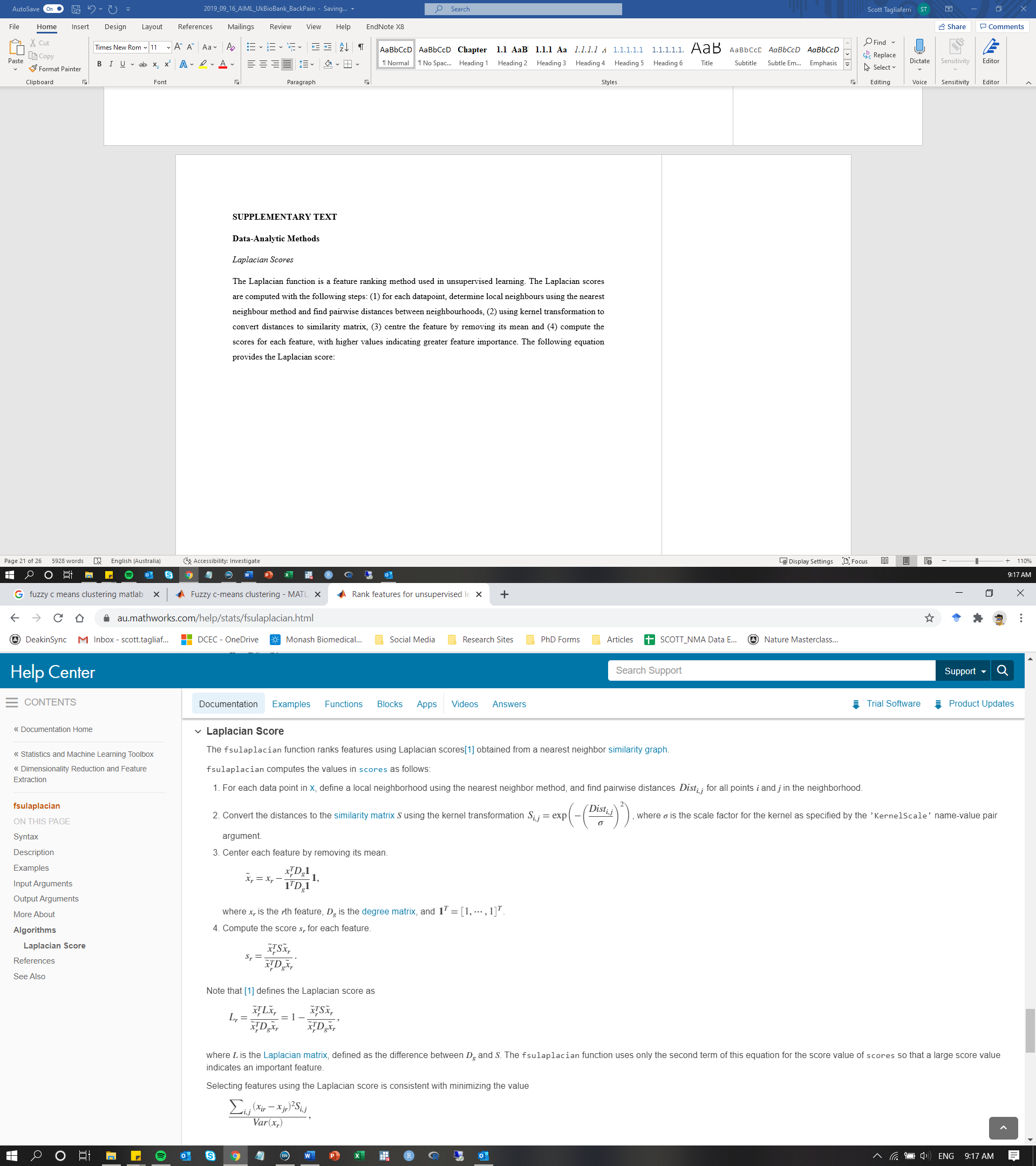

*Calinski-Harabasz cluster validity*

The Calinski-Harabasz^2^ criterion determines the variance ratio to determine the optimal number of clusters. It is a method of determine the between-cluster variance and within-cluster variances based on the numbers of clusters and observations, to determine the most separable clusters. Ideally, large between-cluster and small within-cluster variances are optimal. More details are available here: <https://au.mathworks.com/help/stats/clustering.evaluation.calinskiharabaszevaluation-class.html>. The following equation is used to calculate the Calinski-Harabasz index:


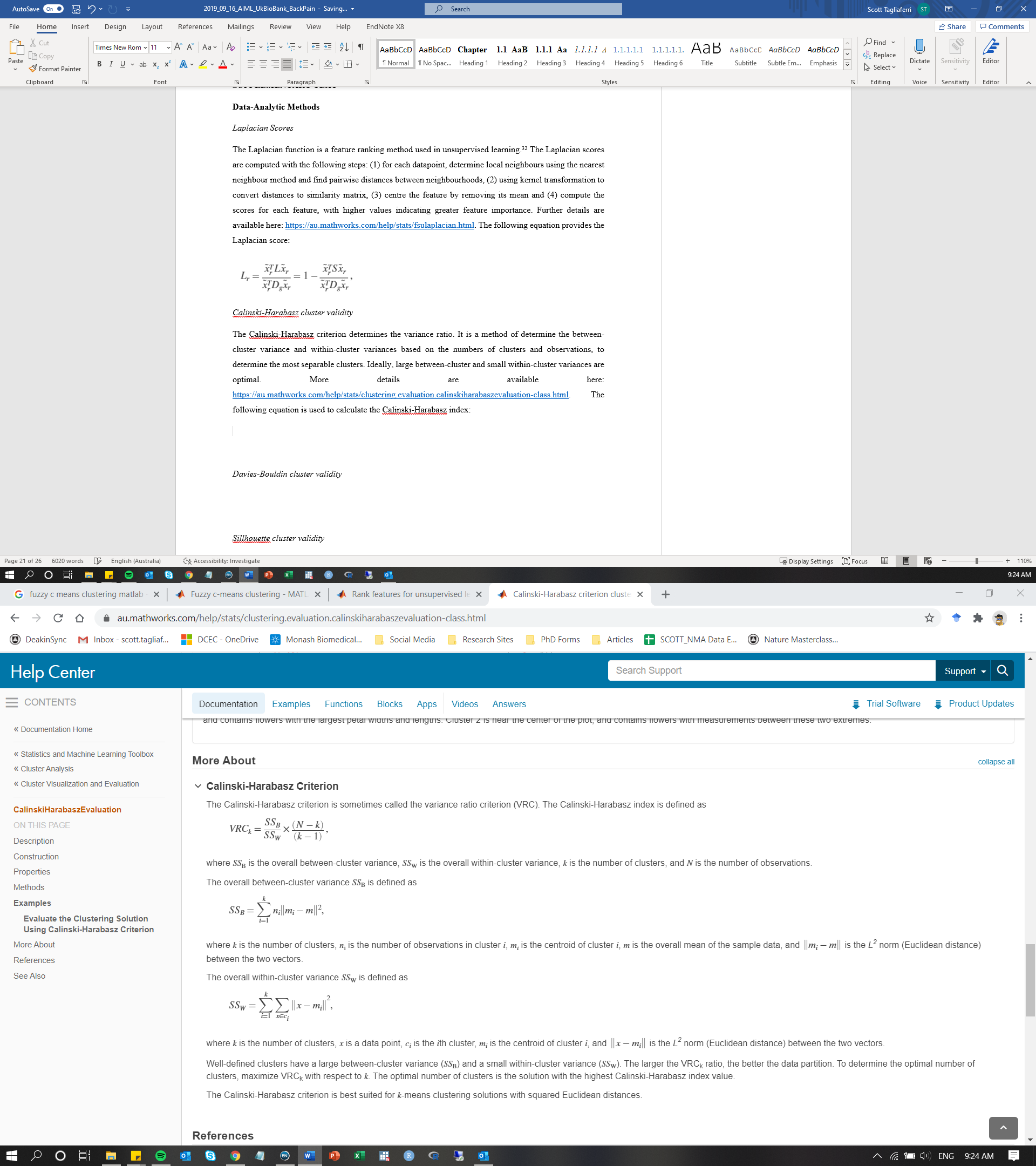


*Davies-Bouldin cluster validity*

The Davies-Bouldin^3^ method is like that of the Calinski-Harabasz, but determined the index based on within-cluster and between-cluster distances to determine the optimal number of clusters. More information can be found here: <https://au.mathworks.com/help/stats/clustering.evaluation.daviesbouldinevaluation-class.html>. The following equation is used to determine the index:


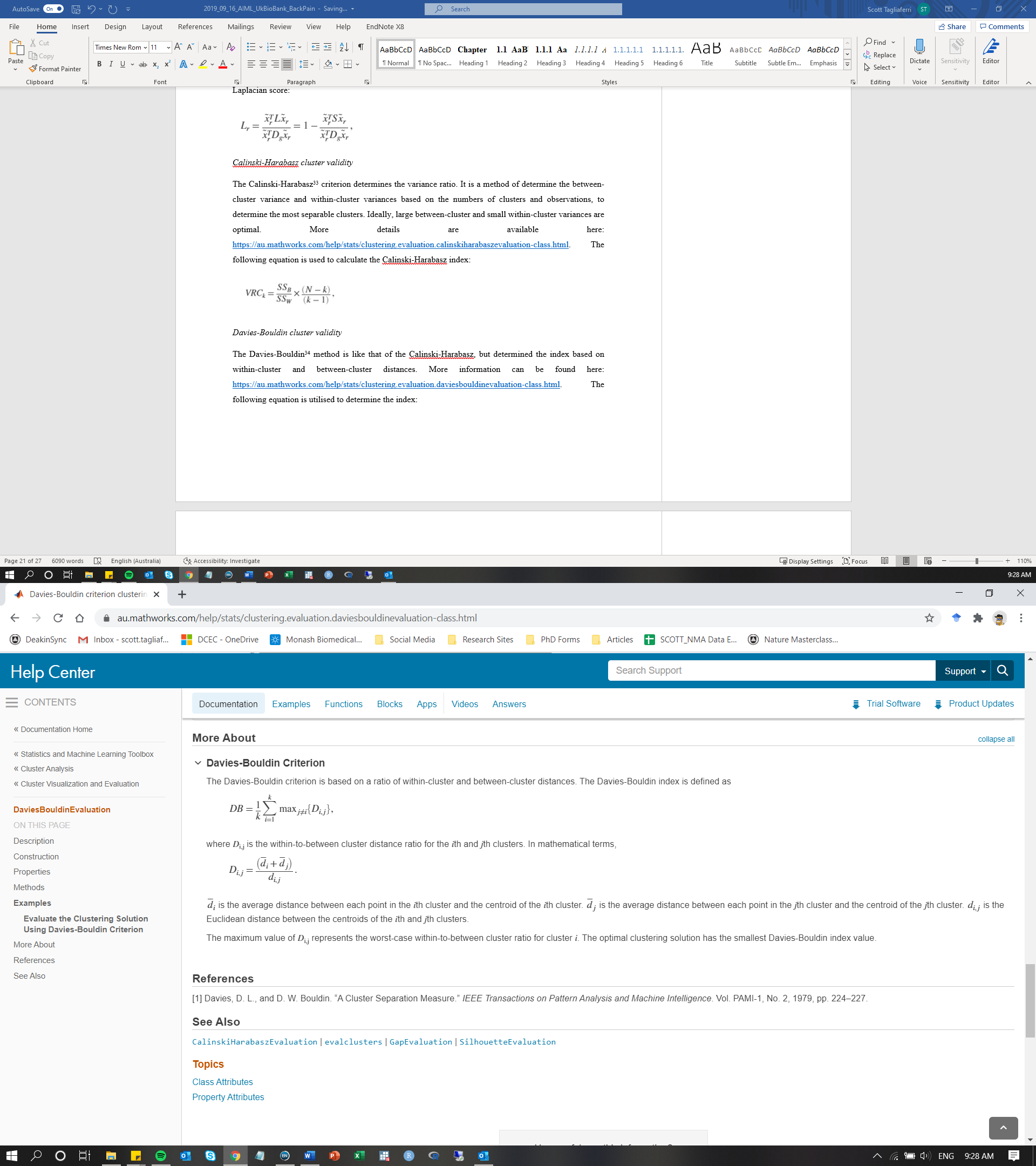


*Silhouette cluster validity* and evaluation

The Silhouette value^4^ determines the optimal cluster number by measuring how similar datapoints are to its own and other clusters. Values range from -1 to 1 with higher values indicating that the datapoint is highly like its cluster. More information is available here: <https://au.mathworks.com/help/stats/clustering.evaluation.silhouetteevaluation-class.html>. The following equation is used to calculate the Silhouette values:


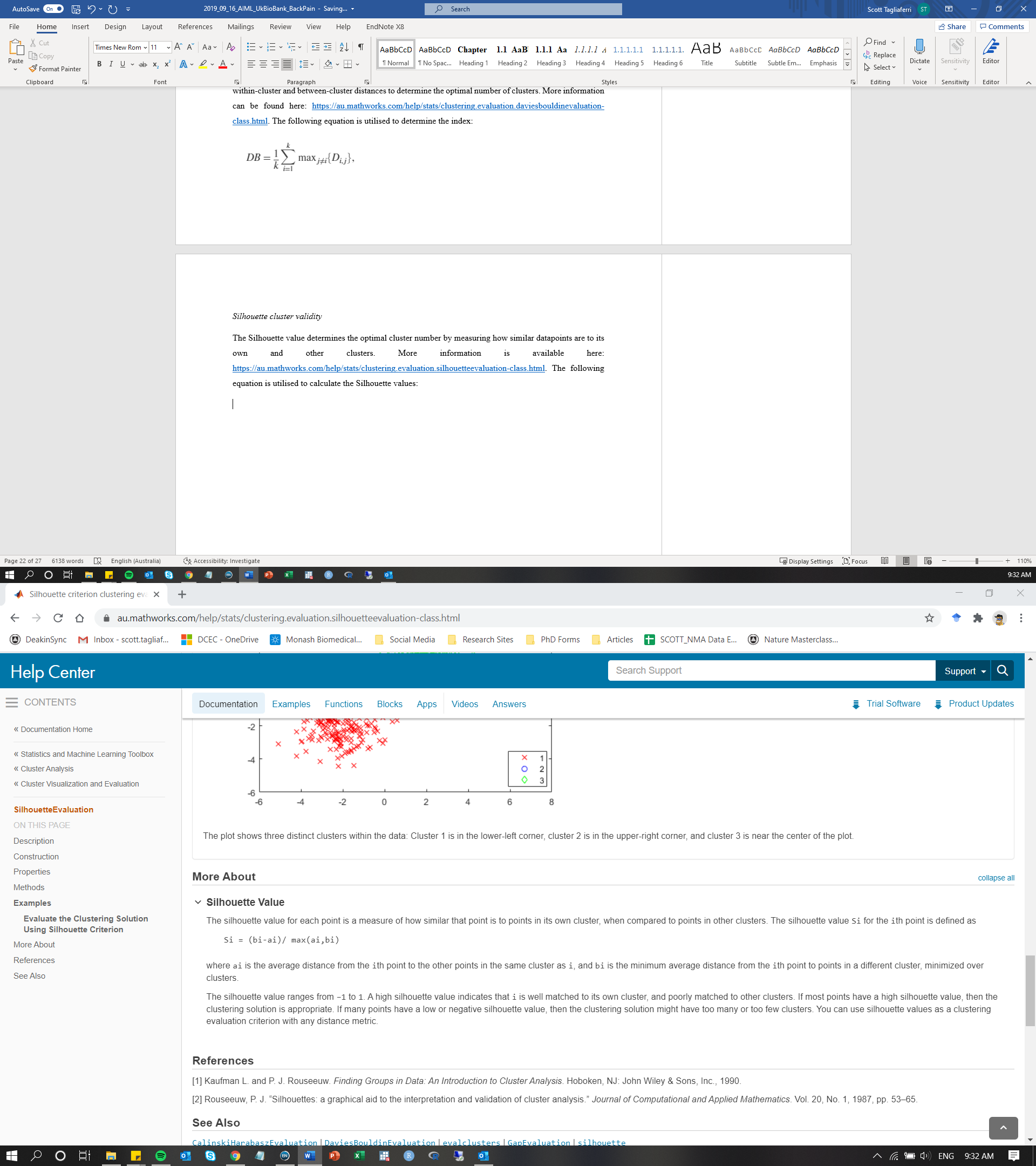


*Discrimination Values*

Discrimination values are used to determine the discriminability and density between clusters.^5^ The discrimination value is calculated as:


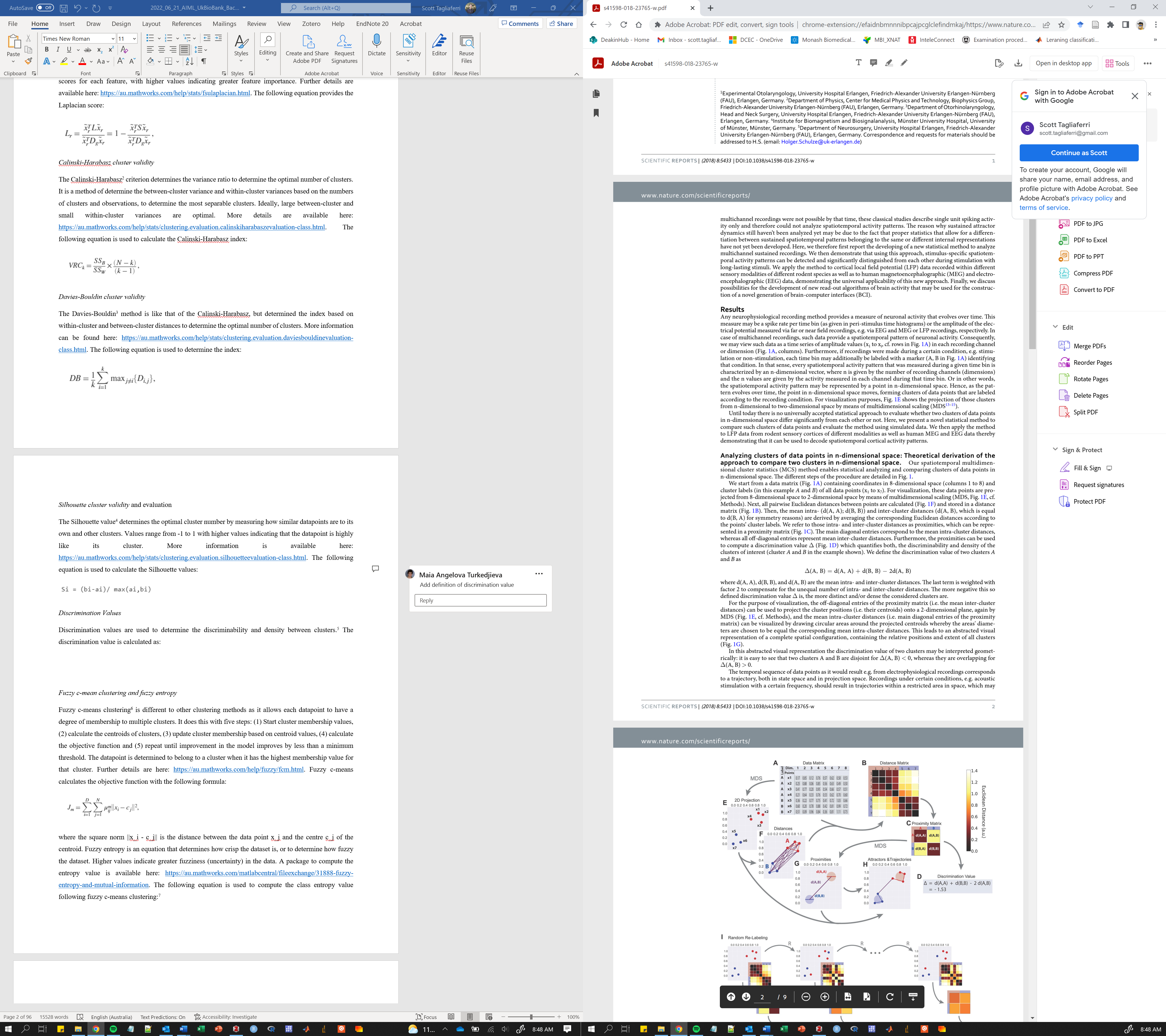


Where the d(A, A) and d(B, B) are the mean intra-cluster distances and d(A, B) is the inter-cluster distance. The inter-cluster distance metric is weighted due to the uneven number of values between intra- and inter-cluster distances. The more negative the values, the most distinct the clusters are.

*Fuzzy c-mean clustering and fuzzy entropy*

Fuzzy c-means clustering^6^ is different to other clustering methods as it allows each datapoint to have a degree of membership to multiple clusters. It does this with five steps: (1) Start cluster membership values, (2) calculate the centroids of clusters, (3) update cluster membership based on centroid values, (4) calculate the objective function and (5) repeat until improvement in the model improves by less than a minimum threshold. The datapoint is determined to belong to a cluster when it has the highest membership value for that cluster. Further details are here: <https://au.mathworks.com/help/fuzzy/fcm.html>. Fuzzy c-means calculates the objective function with the following formula:


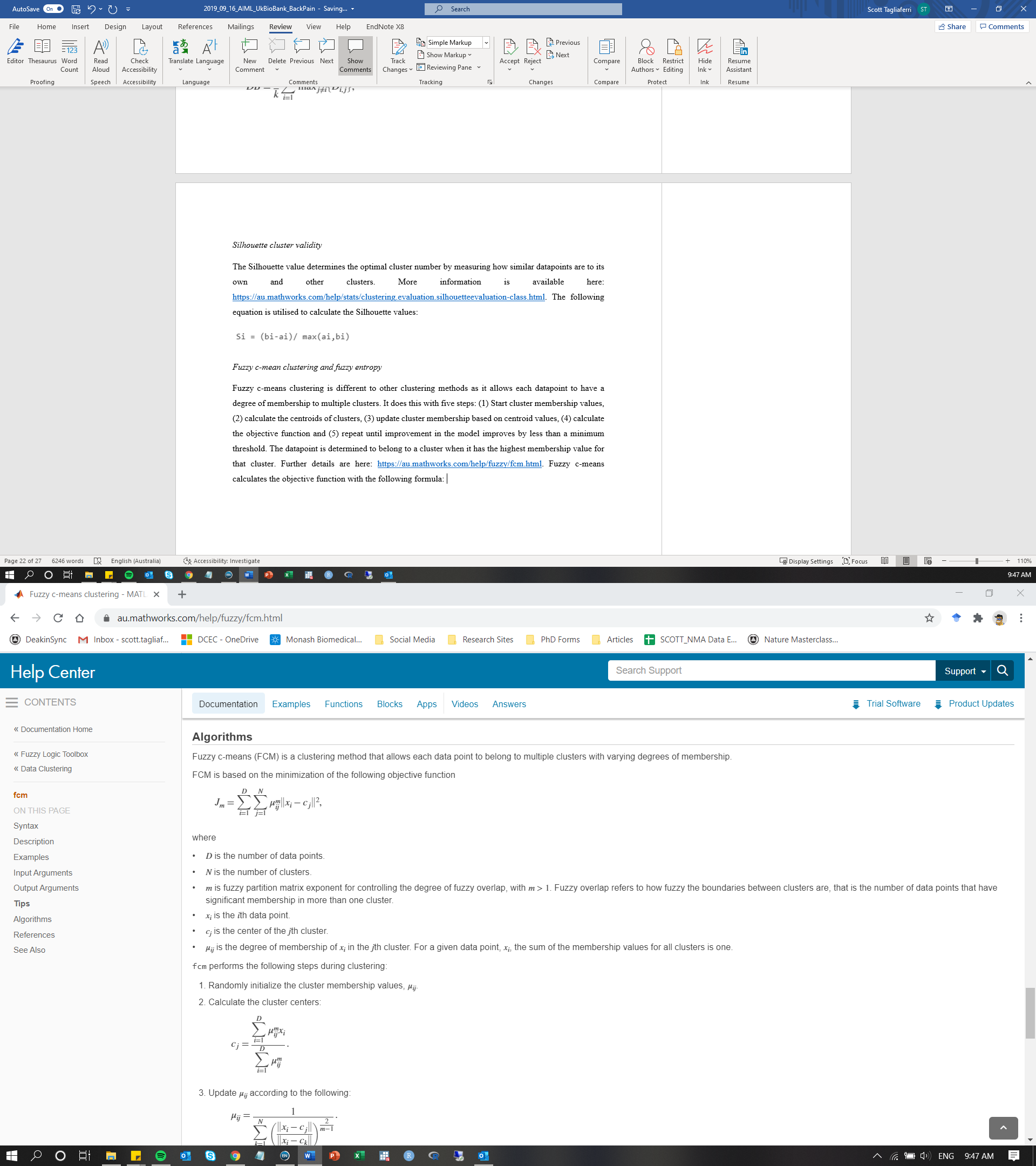


where the square norm ||x_i - c_j|| is the distance between the data point x_j and the centre c_j of the centroid. Fuzzy entropy is an equation that determines how crisp the dataset is, or to determine how fuzzy the dataset. Higher values indicate greater fuzziness (uncertainty) in the data. A package to compute the entropy value is available here: <https://au.mathworks.com/matlabcentral/fileexchange/31888-fuzzy-entropy-and-mutual-information>. The following equation is used to compute the class entropy value following fuzzy c-means clustering:^7^

H_C = -∑(Pr_C*log(Pr_C));

*Classifiers*

We used error-correcting output codes with SVM, Naïve Bayes, KNN and Random Forest templates for multiclass classification. Below provides an overview of how each classifier works:

Support Vector Machine: SVM classifiers are a method of fitting hyperplanes throughout the data where the model determines datapoints are separable, to determine which are most related to others. Observations are labelled based on which side of the hyperplane cut in the data they reside.^8^

Naïve Bayes: Naïve Bayes method applies density estimation to the data. It assigns observations to the most probable class by estimating the posterior probability of each observation to a class. The observation is assigned to the class yielding the maximum posterior probability.^9^

K-Nearest Neighbour: K-Nearest Neighbour algorithms determine observations in the data that is similar through measuring distance metrics of each observation in the data space to identify observations of nearest neighbours. Classes are determined based on the similarity measures between each observation based on features in the data space.^10^

Random Forest: Random Forest classifiers build a set of decision trees based on random vectors within the data, or for example, splits the data to inform a decision regarding the next set of trees. After a certain number of trees are created, each casts of a vote to determine the most popular class of those set of trees from the input data.^11^

*Calibration metrics*

Reliability diagram: The reliability diagram is a plot of the fraction of positive class (y-axis) relative to the mean predicted probabilities (x-axis) for each bin used. For a perfectly calibrated model, it is expected that if the mean predicted probability is 0.7 in one bin, then the fraction of positive classes in that bin should be 0.7. Therefore, a perfectly calibrated model would be represented by a straight line with a 45‑degree angle.^12^

Brier score: The Brier score is the mean squared error between the actual outcome and predicted probabilities.^12^ The calculation is:


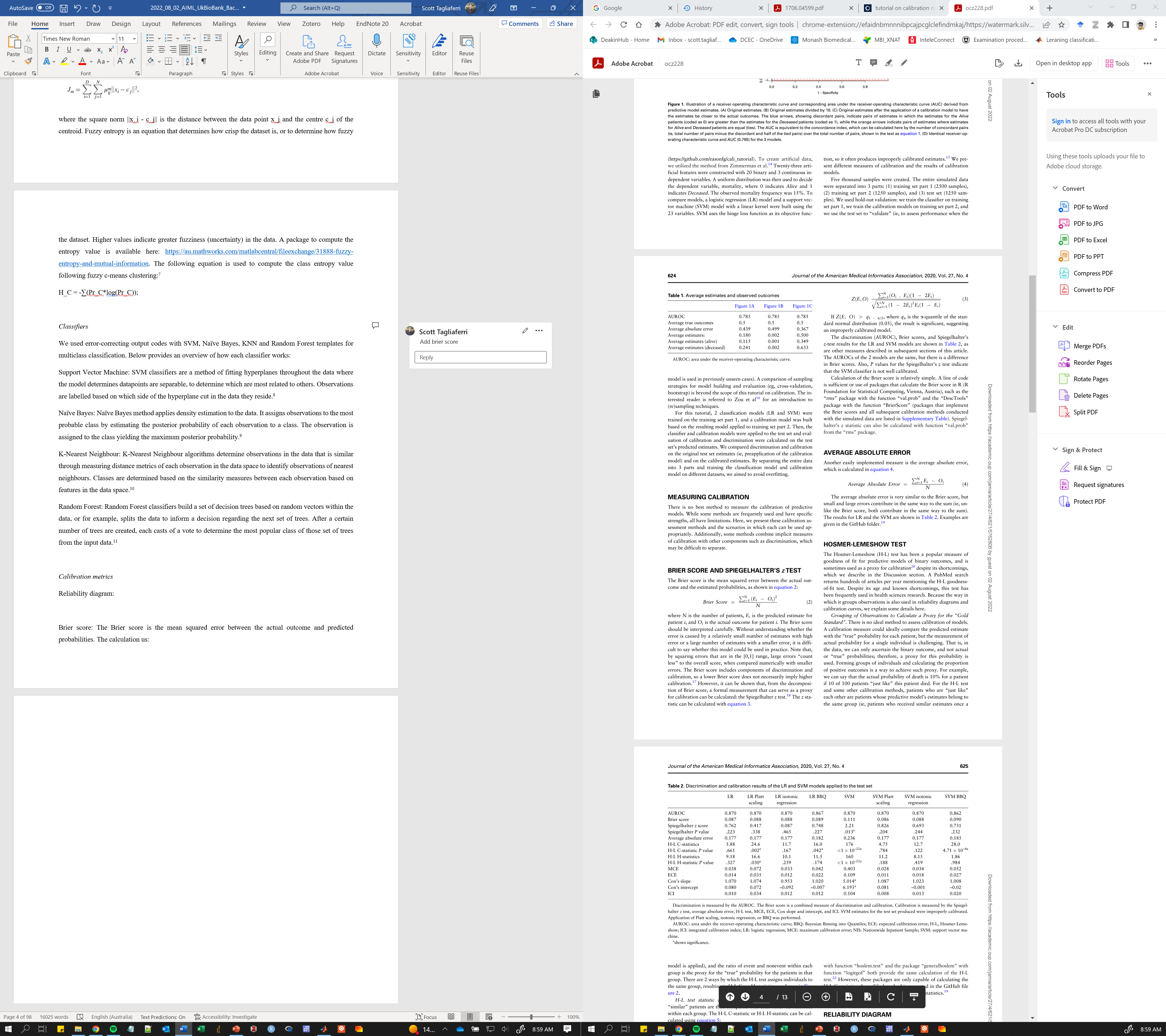


Log Loss: The log loss is the negative average of the log of correct predicted probabilities.^13^ It is calculated as follows for binary classifiers:


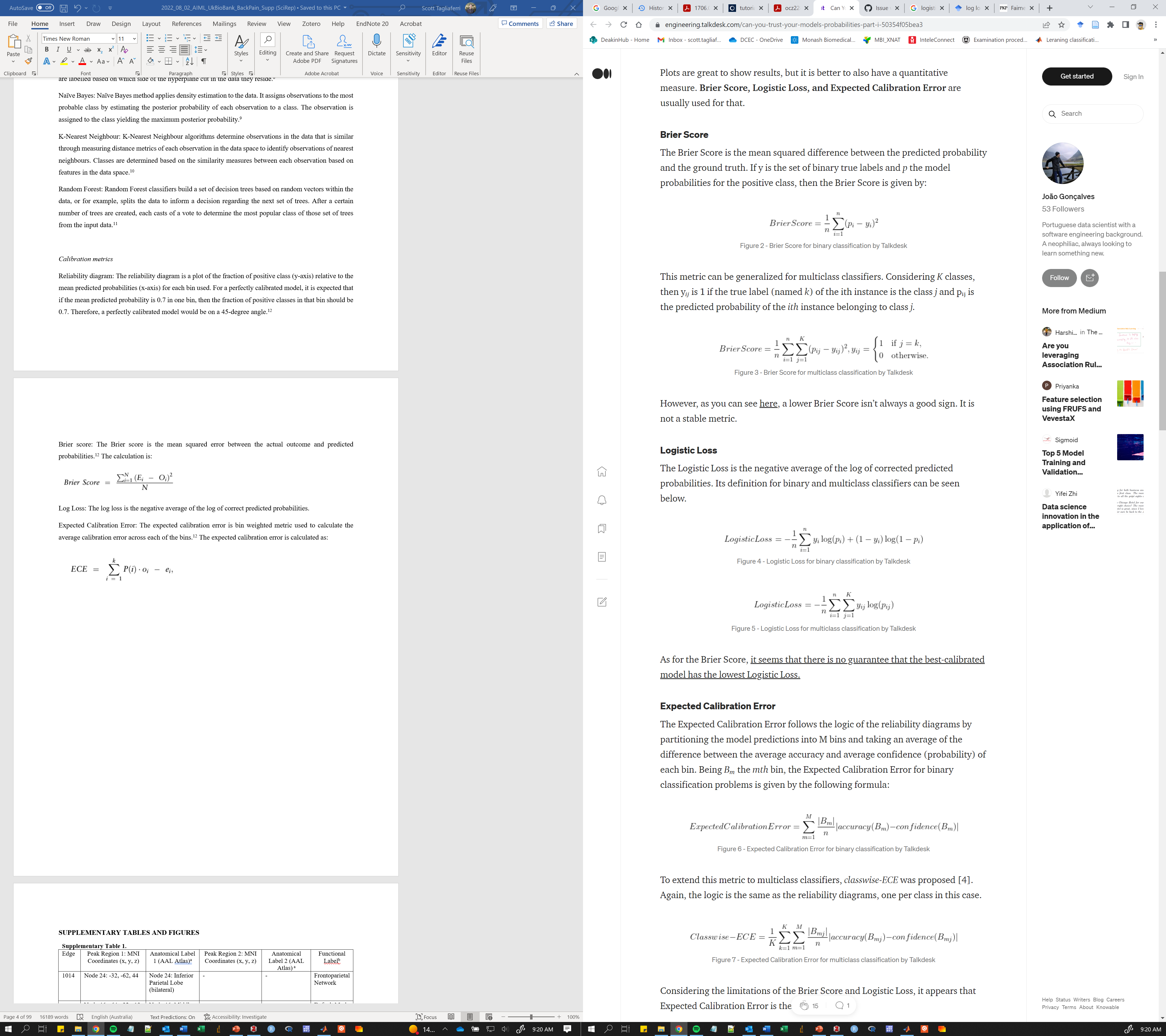


Expected Calibration Error: The expected calibration error is bin weighted metric used to calculate the average calibration error across each of the bins.^12^ The expected calibration error is calculated as:


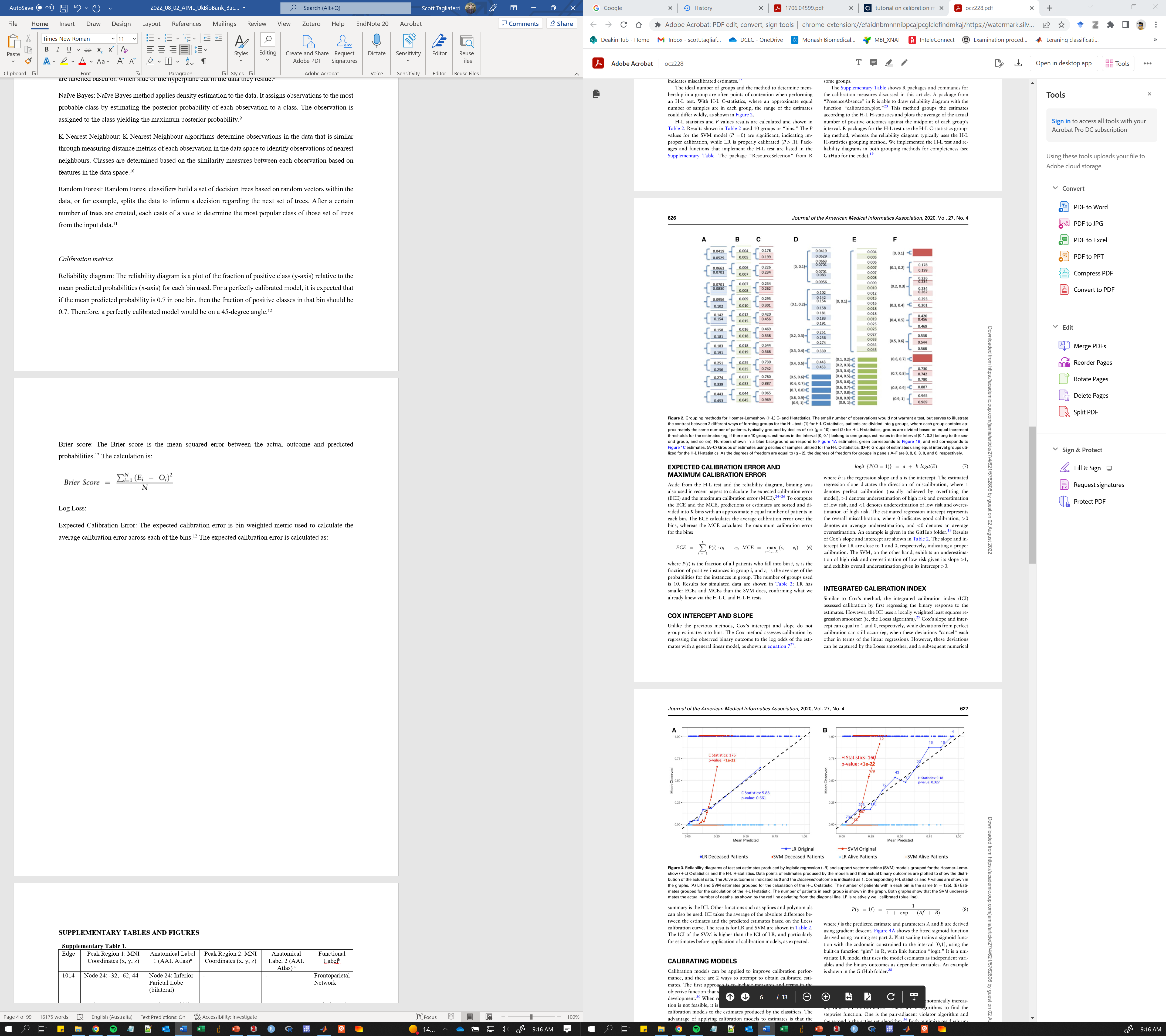


**SUPPLEMENTARY TABLES AND FIGURES**

| **Supplementary Table 1.** | | |  |  |  |
| --- | --- | --- | --- | --- | --- |
| Edge | Peak Region 1: MNI Coordinates (x, y, z) | Anatomical Label 1 (AAL Atlas)^a^ | Peak Region 2: MNI Coordinates (x, y, z) | Anatomical Label 2 (AAL Atlas) ^a^ | Functional Label^b^ |
| 1014 | Node 24: -32, -62, 44 | Node 24: Inferior Parietal Lobe (bilateral) | - | - | Frontoparietal Network |
|  | Node 46: -64, -32, -13 | Node 46: Middle Temporal Gyrus (bilateral) | - | - | Default Mode Network |
| 222 | Node 12: 49, -57, 46 | Node 12: Inferior Parietal Lobe (right only) | Node 12: 39, 53, 2 | Node 12: Middle Frontal Cortex (right only) | Frontoparietal Network |
|  | Node 22: 64, -21, 5 | Node 22: Superior Temporal Gyrus (bilateral) | - | - | Sensorimotor Network |
| 44 | Node 8: 2, -83, 0 | Node 8: Calcarine Cortex (bilateral) | - | - | Visual Network |
|  | Node 10: 14, -53, 15 | Node 10: Precuneus (bilateral) | Node 10: -37, -82, 30 | Node 10: Middle Occipital Gyrus (bilateral) | Default Mode Network |
| Nodes indicated as bilateral also had x coordinates on the opposite side. For example, -32, -62, 44 for Node 24.  ^A^Anatomical locations of peak activations were attained using MNI coordinates of the group-averaged results available from the UKBioBank through the AAL atlas.  ^b^The functional labels were derived from 7-network parcellations available from Yeo 2011 ([10.1152/jn.00338.2011](https://dx.doi.org/10.1152%2Fjn.00338.2011)) | | | | | |

| **Supplementary Table 2.** P-values for t-tests for all 1502 variables. The false discovery rate cut-off was a p=0.002838. Bold variables indicate passing of false discovery rate. vXXX variables indicate a node from fMRI | |
| --- | --- |
| **Variable** | **P-Value** |
| **depress** | **0.00E+00** |
| **bmi** | **1.01E-103** |
| **lonely_social** | **5.40E-44** |
| **s1_grey_vol** | **1.03E-12** |
| **insula_grey_vol** | **2.24E-08** |
| **amygdala_grey_vol** | **3.20E-08** |
| **m1_grey_vol** | **8.09E-08** |
| **mfc_grey_vol** | **1.74E-07** |
| **v849** | **2.21E-07** |
| **v380** | **3.32E-07** |
| **v135** | **3.82E-07** |
| **v1183** | **6.29E-07** |
| **v20** | **1.42E-06** |
| **v1143** | **3.75E-06** |
| **v145** | **4.64E-06** |
| **hippo_grey_vol** | **4.76E-06** |
| **v1357** | **5.16E-06** |
| **s2_grey_vol** | **5.27E-06** |
| **v1148** | **1.26E-05** |
| **v222** | **1.35E-05** |
| **v930** | **1.76E-05** |
| **v142** | **2.74E-05** |
| **v1140** | **4.08E-05** |
| **v262** | **4.12E-05** |
| **frontorbitalcortex_grey_vol** | **4.77E-05** |
| **v93** | **5.17E-05** |
| **v838** | **6.38E-05** |
| **v44** | **6.71E-05** |
| **acc_grey_vol** | **6.87E-05** |
| **v789** | **7.02E-05** |
| **v1253** | **9.02E-05** |
| **v1091** | **9.49E-05** |
| **v102** | **0.000118** |
| **v458** | **0.000172** |
| **v404** | **0.000179** |
| **v464** | **0.00019** |
| **v1165** | **0.0002** |
| **v312** | **0.000246** |
| **v732** | **0.000257** |
| **v581** | **0.000276** |
| **v201** | **0.00029** |
| **v319** | **0.000359** |
| **v164** | **0.000369** |
| **v1286** | **0.000397** |
| **v967** | **0.000414** |
| **v740** | **0.000444** |
| **v1137** | **0.000447** |
| **v369** | **0.000498** |
| **v1207** | **0.000504** |
| **v373** | **0.000527** |
| **v745** | **0.000596** |
| **v547** | **0.000623** |
| **v171** | **0.000679** |
| **v144** | **0.000698** |
| **v344** | **0.000735** |
| **v629** | **0.000749** |
| **v524** | **0.00075** |
| **v337** | **0.000775** |
| **v141** | **0.000858** |
| **v1408** | **0.00087** |
| **v614** | **0.000897** |
| **v1172** | **0.001027** |
| **v47** | **0.001088** |
| **v788** | **0.001127** |
| **v329** | **0.001138** |
| **v961** | **0.001171** |
| **v608** | **0.001186** |
| **v820** | **0.001223** |
| **sma_grey_vol** | **0.001235** |
| **v1076** | **0.00125** |
| **v953** | **0.001262** |
| **v1321** | **0.001315** |
| **v1133** | **0.001362** |
| **v804** | **0.001406** |
| **v861** | **0.001427** |
| **v420** | **0.001437** |
| **v162** | **0.001503** |
| **v639** | **0.001522** |
| **v630** | **0.001612** |
| **v940** | **0.001619** |
| **v1141** | **0.00178** |
| **v1096** | **0.00194** |
| **v1174** | **0.00197** |
| **v291** | **0.002062** |
| **v1083** | **0.002073** |
| **v554** | **0.002144** |
| **v1102** | **0.002186** |
| **v758** | **0.00221** |
| **v23** | **0.002291** |
| **v1294** | **0.002294** |
| **v1014** | **0.002339** |
| **v1288** | **0.002422** |
| **v1193** | **0.002424** |
| **v143** | **0.002502** |
| **v648** | **0.002646** |
| **v57** | **0.002679** |
| **v567** | **0.002711** |
| **v783** | **0.002736** |
| **grip_str_high** | **0.002767** |
| **v546** | **0.002838** |
| v570 | 0.003369 |
| caudate_grey_vol | 0.003384 |
| v260 | 0.003397 |
| v507 | 0.0034 |
| v18 | 0.003511 |
| v607 | 0.003556 |
| v255 | 0.003578 |
| v231 | 0.003749 |
| v385 | 0.003902 |
| v215 | 0.004237 |
| v706 | 0.004247 |
| v469 | 0.004365 |
| v218 | 0.004638 |
| v265 | 0.004779 |
| v119 | 0.004785 |
| v252 | 0.004809 |
| v807 | 0.004882 |
| v1079 | 0.004906 |
| v954 | 0.005099 |
| v582 | 0.005106 |
| v918 | 0.005134 |
| v599 | 0.005222 |
| v959 | 0.005226 |
| v942 | 0.005236 |
| v1349 | 0.005248 |
| v1390 | 0.005354 |
| v1162 | 0.005411 |
| v1156 | 0.005507 |
| v859 | 0.005576 |
| v378 | 0.005816 |
| v1026 | 0.006028 |
| v956 | 0.006028 |
| v1089 | 0.006099 |
| v209 | 0.006221 |
| v327 | 0.006236 |
| v744 | 0.00635 |
| v1167 | 0.006593 |
| v1395 | 0.006693 |
| v1124 | 0.006802 |
| v1375 | 0.007003 |
| v1436 | 0.008204 |
| v131 | 0.008306 |
| v1088 | 0.008355 |
| v619 | 0.00838 |
| v245 | 0.008495 |
| v1335 | 0.008738 |
| v488 | 0.008856 |
| v1130 | 0.008863 |
| v129 | 0.008879 |
| v523 | 0.008951 |
| v902 | 0.009031 |
| v1446 | 0.009206 |
| v239 | 0.009303 |
| v388 | 0.009341 |
| v782 | 0.009571 |
| v391 | 0.009604 |
| v632 | 0.009657 |
| v211 | 0.009676 |
| v400 | 0.009694 |
| v962 | 0.009965 |
| v659 | 0.010191 |
| v389 | 0.0102 |
| v903 | 0.010539 |
| v285 | 0.01056 |
| v72 | 0.010633 |
| v212 | 0.01132 |
| v923 | 0.011341 |
| v1000 | 0.011486 |
| v22 | 0.011586 |
| v168 | 0.012079 |
| v233 | 0.01245 |
| v549 | 0.013217 |
| v79 | 0.013239 |
| v604 | 0.013437 |
| v176 | 0.014081 |
| v1163 | 0.014089 |
| v289 | 0.014389 |
| v994 | 0.014513 |
| v971 | 0.01459 |
| v919 | 0.014737 |
| v858 | 0.014793 |
| v913 | 0.014817 |
| v270 | 0.01487 |
| v714 | 0.015058 |
| v409 | 0.015095 |
| v1013 | 0.01526 |
| v158 | 0.015751 |
| v296 | 0.015918 |
| v646 | 0.015946 |
| v246 | 0.016123 |
| v13 | 0.016771 |
| v836 | 0.016915 |
| v1202 | 0.017057 |
| v1212 | 0.017176 |
| v249 | 0.017188 |
| v658 | 0.017222 |
| v345 | 0.017352 |
| v362 | 0.01754 |
| v384 | 0.017648 |
| v330 | 0.01861 |
| v424 | 0.018951 |
| v647 | 0.019284 |
| v403 | 0.01971 |
| v1050 | 0.020226 |
| v356 | 0.020567 |
| v572 | 0.02067 |
| v101 | 0.021304 |
| v1338 | 0.021537 |
| v772 | 0.021611 |
| v720 | 0.021711 |
| v1189 | 0.021829 |
| v1378 | 0.02207 |
| v1077 | 0.022193 |
| v845 | 0.022247 |
| v891 | 0.023013 |
| v376 | 0.023052 |
| v197 | 0.023067 |
| v579 | 0.023129 |
| v616 | 0.023519 |
| v253 | 0.023576 |
| v541 | 0.023683 |
| v830 | 0.023781 |
| v973 | 0.023799 |
| v203 | 0.023993 |
| v1037 | 0.024011 |
| v1007 | 0.024071 |
| v117 | 0.02425 |
| v1423 | 0.024494 |
| v126 | 0.024625 |
| v387 | 0.024665 |
| v695 | 0.024832 |
| v943 | 0.025166 |
| v1341 | 0.025833 |
| v1034 | 0.026007 |
| v952 | 0.026142 |
| v38 | 0.02654 |
| v287 | 0.02677 |
| v612 | 0.027025 |
| v1441 | 0.027394 |
| v771 | 0.027462 |
| v104 | 0.027541 |
| v1159 | 0.027926 |
| v1315 | 0.028389 |
| v170 | 0.028469 |
| v722 | 0.028475 |
| v536 | 0.028573 |
| v516 | 0.028954 |
| v1138 | 0.028967 |
| v927 | 0.029293 |
| v1067 | 0.029352 |
| v680 | 0.029547 |
| v1463 | 0.030214 |
| v353 | 0.030956 |
| v650 | 0.031181 |
| v1319 | 0.031495 |
| v550 | 0.031803 |
| v1090 | 0.032037 |
| v1388 | 0.032042 |
| v1169 | 0.032893 |
| v1460 | 0.032909 |
| v335 | 0.033051 |
| v1331 | 0.033371 |
| v932 | 0.033473 |
| v552 | 0.034257 |
| v454 | 0.034533 |
| v1449 | 0.034991 |
| v1414 | 0.035562 |
| v1125 | 0.036138 |
| v534 | 0.036369 |
| v1317 | 0.036605 |
| v1144 | 0.03683 |
| v1078 | 0.036867 |
| v263 | 0.037502 |
| v506 | 0.03752 |
| v242 | 0.03771 |
| v199 | 0.038334 |
| v1190 | 0.038714 |
| v281 | 0.039706 |
| v1435 | 0.040217 |
| v1017 | 0.040837 |
| v300 | 0.04092 |
| v1412 | 0.040989 |
| v95 | 0.041491 |
| v52 | 0.041628 |
| v763 | 0.041934 |
| v479 | 0.042039 |
| v654 | 0.042257 |
| v1080 | 0.042438 |
| v868 | 0.042926 |
| v748 | 0.043151 |
| v470 | 0.044252 |
| v165 | 0.0452 |
| v109 | 0.045382 |
| v167 | 0.04545 |
| v700 | 0.045573 |
| v1448 | 0.046147 |
| v1403 | 0.046459 |
| v512 | 0.046662 |
| v1208 | 0.046809 |
| v896 | 0.046901 |
| v661 | 0.047057 |
| v1158 | 0.047288 |
| v1373 | 0.047642 |
| v338 | 0.048186 |
| v734 | 0.048431 |
| v467 | 0.04982 |
| v649 | 0.049897 |
| v412 | 0.049959 |
| v1385 | 0.050301 |
| v965 | 0.051262 |
| v825 | 0.051581 |
| v399 | 0.051662 |
| v205 | 0.051757 |
| v557 | 0.052461 |
| v1192 | 0.052508 |
| v148 | 0.054517 |
| v800 | 0.055413 |
| v756 | 0.055541 |
| v556 | 0.055758 |
| v894 | 0.055845 |
| v931 | 0.055916 |
| v742 | 0.056166 |
| v108 | 0.056203 |
| v1376 | 0.056354 |
| v515 | 0.056863 |
| v37 | 0.057714 |
| v842 | 0.058597 |
| v1248 | 0.059082 |
| v251 | 0.0593 |
| v445 | 0.059405 |
| v1152 | 0.059928 |
| v1166 | 0.060144 |
| v244 | 0.06136 |
| v1093 | 0.061614 |
| v1020 | 0.062548 |
| v668 | 0.063116 |
| v1396 | 0.063867 |
| v368 | 0.063946 |
| v848 | 0.064407 |
| v576 | 0.064495 |
| v908 | 0.064789 |
| v885 | 0.064895 |
| v1134 | 0.06559 |
| v248 | 0.065623 |
| v227 | 0.065973 |
| v1022 | 0.065985 |
| v89 | 0.066471 |
| v543 | 0.066631 |
| v664 | 0.066652 |
| v1021 | 0.066883 |
| v1198 | 0.067294 |
| v739 | 0.067534 |
| v288 | 0.068288 |
| v1216 | 0.069791 |
| v1275 | 0.070704 |
| v234 | 0.071168 |
| v1305 | 0.071293 |
| v881 | 0.071913 |
| v446 | 0.072557 |
| v419 | 0.074313 |
| v795 | 0.074993 |
| v715 | 0.075198 |
| v114 | 0.075517 |
| v1111 | 0.075705 |
| v92 | 0.076339 |
| v986 | 0.076434 |
| v1 | 0.077269 |
| v1277 | 0.078328 |
| v396 | 0.078808 |
| v1119 | 0.078847 |
| v1271 | 0.079228 |
| v301 | 0.079437 |
| v1059 | 0.080856 |
| v764 | 0.080884 |
| v175 | 0.080998 |
| v634 | 0.081904 |
| v950 | 0.082149 |
| v317 | 0.082755 |
| v282 | 0.083067 |
| v542 | 0.08341 |
| v1018 | 0.083427 |
| v1364 | 0.083934 |
| v809 | 0.084591 |
| v562 | 0.085528 |
| v1346 | 0.085803 |
| v991 | 0.085864 |
| v381 | 0.086298 |
| v818 | 0.086339 |
| v27 | 0.086942 |
| v808 | 0.086997 |
| v1155 | 0.087126 |
| v313 | 0.087527 |
| v214 | 0.087777 |
| v983 | 0.088137 |
| v981 | 0.08824 |
| v501 | 0.088519 |
| v670 | 0.088539 |
| v19 | 0.088653 |
| v228 | 0.089332 |
| v1366 | 0.089667 |
| v691 | 0.089742 |
| v1118 | 0.090134 |
| v780 | 0.090269 |
| v1223 | 0.090427 |
| v1171 | 0.09148 |
| v425 | 0.091775 |
| v236 | 0.091981 |
| v560 | 0.092331 |
| v693 | 0.09344 |
| v920 | 0.093787 |
| v657 | 0.093833 |
| v1365 | 0.094098 |
| v96 | 0.094708 |
| v819 | 0.094715 |
| v685 | 0.095105 |
| v645 | 0.095755 |
| v593 | 0.096239 |
| v839 | 0.096283 |
| v823 | 0.0965 |
| v295 | 0.098204 |
| v595 | 0.098511 |
| v548 | 0.098811 |
| v137 | 0.099648 |
| v846 | 0.099662 |
| v402 | 0.099705 |
| v696 | 0.100414 |
| v1405 | 0.10044 |
| v602 | 0.10089 |
| v883 | 0.102212 |
| v1333 | 0.102252 |
| v1063 | 0.102919 |
| v46 | 0.103237 |
| v1475 | 0.103825 |
| v82 | 0.104166 |
| v583 | 0.104248 |
| v134 | 0.104826 |
| v494 | 0.107383 |
| v558 | 0.107756 |
| v855 | 0.107902 |
| v444 | 0.108043 |
| v781 | 0.10807 |
| v314 | 0.108615 |
| v796 | 0.109165 |
| v1424 | 0.110953 |
| v226 | 0.111138 |
| v1347 | 0.111844 |
| v1256 | 0.112451 |
| v257 | 0.113086 |
| v160 | 0.113212 |
| v451 | 0.11373 |
| v511 | 0.114802 |
| v1043 | 0.114958 |
| v36 | 0.115066 |
| v83 | 0.115299 |
| v350 | 0.116087 |
| v916 | 0.11639 |
| v1454 | 0.116706 |
| v1397 | 0.117903 |
| v752 | 0.118211 |
| v1110 | 0.118682 |
| v130 | 0.118787 |
| v1472 | 0.120608 |
| v276 | 0.121122 |
| v1087 | 0.12118 |
| v1440 | 0.121267 |
| v1372 | 0.121946 |
| v1284 | 0.122182 |
| v292 | 0.122358 |
| v1293 | 0.122692 |
| v1299 | 0.123021 |
| v455 | 0.123241 |
| v1470 | 0.123941 |
| v857 | 0.124639 |
| v15 | 0.124722 |
| v736 | 0.125554 |
| v1081 | 0.125568 |
| v332 | 0.125665 |
| v531 | 0.125792 |
| v80 | 0.126902 |
| v1217 | 0.128051 |
| v1298 | 0.128184 |
| v762 | 0.128739 |
| v759 | 0.132064 |
| v274 | 0.133041 |
| v573 | 0.13308 |
| v1261 | 0.13314 |
| v1039 | 0.133225 |
| v1164 | 0.133999 |
| v1024 | 0.135303 |
| v1415 | 0.135476 |
| v1139 | 0.135578 |
| v116 | 0.136846 |
| v138 | 0.136989 |
| v1179 | 0.137207 |
| v1235 | 0.1373 |
| v1113 | 0.137392 |
| v683 | 0.13864 |
| v955 | 0.138773 |
| v716 | 0.139005 |
| v316 | 0.13931 |
| v1057 | 0.140149 |
| v50 | 0.140528 |
| v1226 | 0.140707 |
| v1343 | 0.141563 |
| v1173 | 0.141952 |
| v475 | 0.142318 |
| v225 | 0.143097 |
| v1316 | 0.143277 |
| v977 | 0.144137 |
| v946 | 0.145238 |
| v336 | 0.145646 |
| v323 | 0.145687 |
| v339 | 0.145783 |
| v348 | 0.145803 |
| v448 | 0.146693 |
| v186 | 0.14738 |
| v692 | 0.148213 |
| v124 | 0.148788 |
| v438 | 0.14879 |
| v1117 | 0.149426 |
| v341 | 0.149897 |
| v34 | 0.150145 |
| v1320 | 0.150359 |
| v1339 | 0.15055 |
| v1356 | 0.150553 |
| v1127 | 0.150632 |
| v866 | 0.150844 |
| v1262 | 0.150915 |
| v136 | 0.151819 |
| v697 | 0.151908 |
| v63 | 0.152127 |
| v1086 | 0.152505 |
| v62 | 0.152683 |
| v1471 | 0.152716 |
| v711 | 0.152983 |
| v1264 | 0.152996 |
| v854 | 0.153883 |
| v1122 | 0.154676 |
| v33 | 0.154884 |
| v277 | 0.154897 |
| v1418 | 0.155436 |
| v1019 | 0.155792 |
| v1071 | 0.155828 |
| v185 | 0.155929 |
| v989 | 0.15773 |
| v1161 | 0.158566 |
| v561 | 0.159284 |
| v877 | 0.159863 |
| v987 | 0.160212 |
| v1121 | 0.160875 |
| v1237 | 0.160917 |
| v1224 | 0.162644 |
| v887 | 0.162732 |
| v925 | 0.163039 |
| v70 | 0.165722 |
| v721 | 0.166404 |
| v566 | 0.166756 |
| v125 | 0.167463 |
| v677 | 0.168454 |
| v340 | 0.168961 |
| v1151 | 0.169096 |
| v594 | 0.16911 |
| v863 | 0.169361 |
| v1269 | 0.17031 |
| v354 | 0.171252 |
| v1484 | 0.173518 |
| v1100 | 0.174449 |
| v766 | 0.174639 |
| v1290 | 0.174796 |
| v575 | 0.175306 |
| v189 | 0.176434 |
| v749 | 0.176482 |
| v1254 | 0.176648 |
| v785 | 0.178005 |
| v32 | 0.178696 |
| v503 | 0.178727 |
| v1355 | 0.178905 |
| v1052 | 0.179145 |
| v473 | 0.179786 |
| v660 | 0.182253 |
| v509 | 0.182504 |
| v588 | 0.183258 |
| v1045 | 0.185676 |
| v1318 | 0.186396 |
| v843 | 0.187798 |
| v611 | 0.188099 |
| v1379 | 0.188356 |
| v1236 | 0.188774 |
| v816 | 0.189005 |
| v422 | 0.189227 |
| v914 | 0.190875 |
| v456 | 0.190944 |
| v1453 | 0.191554 |
| v958 | 0.193333 |
| v774 | 0.193657 |
| v1061 | 0.194176 |
| v1285 | 0.195071 |
| v770 | 0.195763 |
| v1003 | 0.196195 |
| v1464 | 0.196566 |
| v778 | 0.19698 |
| v623 | 0.198188 |
| v1457 | 0.19998 |
| v498 | 0.200789 |
| v204 | 0.201015 |
| v719 | 0.2011 |
| v40 | 0.201739 |
| v1247 | 0.201944 |
| v254 | 0.202844 |
| v100 | 0.204641 |
| v997 | 0.204742 |
| v361 | 0.204858 |
| v1459 | 0.205548 |
| v190 | 0.206675 |
| v484 | 0.210402 |
| v822 | 0.21169 |
| v155 | 0.212123 |
| v1268 | 0.212641 |
| v787 | 0.213637 |
| v1058 | 0.214553 |
| v275 | 0.214816 |
| v1129 | 0.215054 |
| v1046 | 0.215209 |
| v173 | 0.215395 |
| v401 | 0.217015 |
| v856 | 0.217195 |
| v898 | 0.217558 |
| v53 | 0.217836 |
| v1483 | 0.218466 |
| v1381 | 0.221131 |
| v1426 | 0.221847 |
| v993 | 0.222196 |
| v61 | 0.222222 |
| v638 | 0.222751 |
| v1399 | 0.2229 |
| v217 | 0.22437 |
| v760 | 0.224415 |
| v2 | 0.225553 |
| v684 | 0.225644 |
| v1041 | 0.22627 |
| v598 | 0.228431 |
| v540 | 0.228885 |
| v831 | 0.229251 |
| v784 | 0.229643 |
| v322 | 0.229834 |
| v1025 | 0.23076 |
| v1245 | 0.230878 |
| v799 | 0.231179 |
| v1393 | 0.231503 |
| v712 | 0.232922 |
| v12 | 0.233535 |
| v1308 | 0.233565 |
| v140 | 0.234485 |
| v917 | 0.234723 |
| v1215 | 0.235137 |
| v1452 | 0.235591 |
| v1146 | 0.235603 |
| v1234 | 0.23584 |
| v735 | 0.236123 |
| v240 | 0.23647 |
| v813 | 0.236482 |
| v216 | 0.237224 |
| v1368 | 0.237399 |
| v1387 | 0.237816 |
| v9 | 0.238093 |
| v1069 | 0.238651 |
| v1363 | 0.23868 |
| v405 | 0.239275 |
| v150 | 0.239353 |
| v1409 | 0.239599 |
| v1433 | 0.240362 |
| v492 | 0.24044 |
| v223 | 0.2407 |
| v1473 | 0.241295 |
| v1205 | 0.24152 |
| v267 | 0.242972 |
| v844 | 0.244604 |
| v59 | 0.244845 |
| v325 | 0.24489 |
| v897 | 0.245 |
| v191 | 0.24571 |
| v901 | 0.247593 |
| v430 | 0.248272 |
| v1252 | 0.248484 |
| v77 | 0.248592 |
| v25 | 0.249206 |
| v928 | 0.249654 |
| v519 | 0.251826 |
| v98 | 0.252794 |
| v452 | 0.253455 |
| v224 | 0.253538 |
| v1287 | 0.253974 |
| v893 | 0.255082 |
| v324 | 0.255979 |
| v1062 | 0.256787 |
| v103 | 0.257538 |
| v1324 | 0.258375 |
| v121 | 0.259107 |
| v1340 | 0.259484 |
| v398 | 0.262474 |
| v243 | 0.26396 |
| v933 | 0.264828 |
| v1142 | 0.266025 |
| v1049 | 0.266825 |
| v1360 | 0.267403 |
| v196 | 0.268937 |
| v440 | 0.270731 |
| v360 | 0.271157 |
| v1092 | 0.271911 |
| v311 | 0.272006 |
| v1280 | 0.272257 |
| v590 | 0.272401 |
| v457 | 0.273829 |
| v181 | 0.27497 |
| v41 | 0.27684 |
| v482 | 0.276895 |
| v1300 | 0.277545 |
| v948 | 0.277618 |
| v1239 | 0.278505 |
| v910 | 0.279293 |
| v708 | 0.279295 |
| v681 | 0.279565 |
| v1404 | 0.27969 |
| v474 | 0.279782 |
| v1257 | 0.280009 |
| v929 | 0.280833 |
| v1431 | 0.28123 |
| v1461 | 0.283898 |
| v613 | 0.284072 |
| v1176 | 0.284512 |
| v990 | 0.284724 |
| v978 | 0.285621 |
| v152 | 0.288303 |
| v1259 | 0.290666 |
| v468 | 0.290669 |
| v188 | 0.291578 |
| v974 | 0.292302 |
| v651 | 0.292393 |
| v596 | 0.293229 |
| v1221 | 0.294834 |
| v1291 | 0.297488 |
| v530 | 0.297811 |
| v628 | 0.299612 |
| v416 | 0.299633 |
| v1283 | 0.299878 |
| v434 | 0.300039 |
| v1456 | 0.300485 |
| v769 | 0.301038 |
| v869 | 0.302211 |
| v1438 | 0.302944 |
| v26 | 0.303138 |
| v280 | 0.303296 |
| v545 | 0.304209 |
| v472 | 0.304434 |
| v1327 | 0.304645 |
| v1006 | 0.308191 |
| v303 | 0.308382 |
| v687 | 0.308823 |
| v259 | 0.309013 |
| v120 | 0.310064 |
| v395 | 0.310412 |
| v128 | 0.311045 |
| v1181 | 0.311891 |
| v90 | 0.313234 |
| v1214 | 0.316697 |
| v1402 | 0.317555 |
| v1230 | 0.318048 |
| v568 | 0.319656 |
| v183 | 0.319665 |
| v1002 | 0.321297 |
| v157 | 0.321641 |
| v247 | 0.322302 |
| v1095 | 0.322453 |
| v951 | 0.322583 |
| v1184 | 0.322813 |
| v56 | 0.323902 |
| v724 | 0.325323 |
| v520 | 0.325326 |
| v1149 | 0.325519 |
| v895 | 0.325654 |
| v483 | 0.326289 |
| v924 | 0.327038 |
| v264 | 0.327055 |
| v674 | 0.327346 |
| v1394 | 0.327758 |
| v972 | 0.328014 |
| v318 | 0.329094 |
| v477 | 0.329749 |
| v1175 | 0.330319 |
| v478 | 0.331314 |
| v161 | 0.331746 |
| v306 | 0.333321 |
| v824 | 0.333765 |
| v966 | 0.334072 |
| v206 | 0.334983 |
| v847 | 0.335281 |
| v1074 | 0.335506 |
| v741 | 0.336465 |
| v221 | 0.336495 |
| v1033 | 0.336862 |
| v139 | 0.337592 |
| v55 | 0.338859 |
| v1168 | 0.339238 |
| v1392 | 0.339727 |
| v258 | 0.340132 |
| v1260 | 0.340888 |
| v1005 | 0.341091 |
| v1015 | 0.342475 |
| v1085 | 0.343025 |
| v1016 | 0.345097 |
| v1314 | 0.34637 |
| v99 | 0.347196 |
| v461 | 0.348331 |
| v1229 | 0.349223 |
| v518 | 0.349619 |
| v761 | 0.349736 |
| v906 | 0.349875 |
| v882 | 0.350122 |
| v250 | 0.350645 |
| v365 | 0.350765 |
| v1325 | 0.351624 |
| v394 | 0.352563 |
| v1255 | 0.352596 |
| v429 | 0.355068 |
| v797 | 0.355084 |
| v672 | 0.357613 |
| v1182 | 0.358087 |
| v87 | 0.360091 |
| v392 | 0.361134 |
| v775 | 0.361719 |
| v860 | 0.361806 |
| v873 | 0.362117 |
| v1304 | 0.362927 |
| v754 | 0.364379 |
| v17 | 0.364752 |
| v298 | 0.36539 |
| v1411 | 0.366326 |
| v1447 | 0.367362 |
| v500 | 0.367705 |
| v876 | 0.367885 |
| v1296 | 0.368007 |
| v707 | 0.370526 |
| v1038 | 0.37053 |
| v383 | 0.370579 |
| v1065 | 0.371273 |
| v3 | 0.371353 |
| v1401 | 0.372817 |
| v481 | 0.37329 |
| v960 | 0.373442 |
| v790 | 0.373502 |
| v750 | 0.373538 |
| v115 | 0.375884 |
| v442 | 0.37654 |
| v343 | 0.377152 |
| v193 | 0.377304 |
| v1345 | 0.37768 |
| v1109 | 0.37816 |
| v689 | 0.37841 |
| v493 | 0.380693 |
| v832 | 0.38136 |
| v43 | 0.381408 |
| v615 | 0.382532 |
| v304 | 0.383904 |
| v710 | 0.383915 |
| v690 | 0.383977 |
| v78 | 0.384032 |
| v294 | 0.384599 |
| v686 | 0.384951 |
| v1027 | 0.385885 |
| thalamus_grey_vol | 0.387501 |
| v21 | 0.387997 |
| v172 | 0.388198 |
| v113 | 0.388248 |
| v182 | 0.388892 |
| v29 | 0.389023 |
| v309 | 0.390254 |
| v1391 | 0.392793 |
| v154 | 0.395335 |
| v1336 | 0.396077 |
| v1469 | 0.397312 |
| v238 | 0.398101 |
| v75 | 0.400312 |
| v76 | 0.401081 |
| v1211 | 0.401129 |
| v777 | 0.402226 |
| v1307 | 0.402707 |
| v1001 | 0.402967 |
| v635 | 0.405713 |
| v489 | 0.405987 |
| v640 | 0.406089 |
| v178 | 0.406109 |
| v980 | 0.408384 |
| v837 | 0.40984 |
| v551 | 0.41086 |
| v1106 | 0.411027 |
| v1359 | 0.411082 |
| v156 | 0.412291 |
| v1099 | 0.412559 |
| v54 | 0.414777 |
| v1425 | 0.415512 |
| v655 | 0.417942 |
| v1233 | 0.420514 |
| v1382 | 0.421149 |
| v159 | 0.421239 |
| v349 | 0.421469 |
| v627 | 0.421982 |
| v1437 | 0.422378 |
| v609 | 0.423324 |
| v60 | 0.424073 |
| v112 | 0.424399 |
| v702 | 0.42555 |
| v1243 | 0.426244 |
| v1225 | 0.426584 |
| v1428 | 0.428603 |
| v828 | 0.43025 |
| v1010 | 0.431937 |
| v16 | 0.434944 |
| v982 | 0.435221 |
| v872 | 0.435319 |
| v798 | 0.435392 |
| v1361 | 0.437909 |
| v1042 | 0.438497 |
| v194 | 0.438551 |
| v460 | 0.439978 |
| v305 | 0.441181 |
| v187 | 0.441865 |
| v1031 | 0.442981 |
| v1274 | 0.443095 |
| v1445 | 0.443875 |
| v834 | 0.446001 |
| v97 | 0.446317 |
| v829 | 0.447537 |
| v64 | 0.447832 |
| v527 | 0.448334 |
| v426 | 0.448704 |
| v1108 | 0.448704 |
| v374 | 0.449192 |
| v779 | 0.4497 |
| v105 | 0.449825 |
| v1244 | 0.451106 |
| v727 | 0.451282 |
| v806 | 0.451301 |
| v603 | 0.451752 |
| v1354 | 0.452851 |
| v1439 | 0.45394 |
| v768 | 0.455034 |
| v315 | 0.456485 |
| v1068 | 0.456921 |
| v1238 | 0.45774 |
| v418 | 0.457746 |
| v617 | 0.458042 |
| v1066 | 0.458157 |
| v1242 | 0.461007 |
| v5 | 0.46158 |
| v743 | 0.466205 |
| v1272 | 0.468455 |
| v1153 | 0.470102 |
| v1466 | 0.471253 |
| v237 | 0.472142 |
| v591 | 0.473717 |
| v1116 | 0.474304 |
| v1073 | 0.476054 |
| v1458 | 0.477713 |
| v1103 | 0.478622 |
| v563 | 0.478962 |
| v767 | 0.479002 |
| v261 | 0.480494 |
| v213 | 0.484544 |
| v1030 | 0.488061 |
| v1128 | 0.488224 |
| v1279 | 0.488357 |
| v995 | 0.48868 |
| v517 | 0.488795 |
| v676 | 0.490599 |
| v232 | 0.491626 |
| v865 | 0.492499 |
| v538 | 0.492865 |
| v921 | 0.492929 |
| v219 | 0.493394 |
| v889 | 0.493534 |
| v610 | 0.493884 |
| v491 | 0.494166 |
| v184 | 0.49532 |
| v202 | 0.495474 |
| v678 | 0.497424 |
| v935 | 0.498008 |
| v235 | 0.498097 |
| v870 | 0.498346 |
| v1443 | 0.499768 |
| v45 | 0.501323 |
| v321 | 0.501391 |
| v1044 | 0.502337 |
| v198 | 0.504075 |
| v886 | 0.504971 |
| v1386 | 0.504983 |
| v665 | 0.50538 |
| v375 | 0.508199 |
| v1131 | 0.510602 |
| v1263 | 0.510617 |
| v513 | 0.510712 |
| v840 | 0.511053 |
| v1311 | 0.511613 |
| v427 | 0.51192 |
| v853 | 0.511941 |
| v177 | 0.51236 |
| v74 | 0.513893 |
| v310 | 0.514182 |
| v1195 | 0.514881 |
| v709 | 0.515178 |
| v1241 | 0.515673 |
| v508 | 0.515727 |
| v580 | 0.516675 |
| v352 | 0.517564 |
| v1323 | 0.517916 |
| v663 | 0.519697 |
| v230 | 0.519723 |
| v299 | 0.519857 |
| v14 | 0.52012 |
| v600 | 0.520505 |
| v791 | 0.520683 |
| v133 | 0.520837 |
| v624 | 0.521394 |
| v535 | 0.522087 |
| v587 | 0.523569 |
| v871 | 0.524667 |
| v1429 | 0.525197 |
| v643 | 0.525601 |
| v502 | 0.525716 |
| v504 | 0.526418 |
| v1427 | 0.526772 |
| v1170 | 0.526925 |
| v1344 | 0.526983 |
| v1101 | 0.530669 |
| v1187 | 0.5324 |
| v1054 | 0.53454 |
| v208 | 0.536954 |
| v1105 | 0.537022 |
| v522 | 0.539084 |
| v1201 | 0.541959 |
| v841 | 0.541999 |
| v618 | 0.544064 |
| v386 | 0.546069 |
| v462 | 0.547545 |
| v957 | 0.548394 |
| v1374 | 0.549225 |
| v11 | 0.549918 |
| v1334 | 0.550365 |
| v878 | 0.550426 |
| v146 | 0.552129 |
| v359 | 0.552375 |
| v1056 | 0.552705 |
| v465 | 0.553727 |
| v447 | 0.554394 |
| v904 | 0.555015 |
| v1228 | 0.555761 |
| v1150 | 0.557628 |
| v1455 | 0.557662 |
| v308 | 0.558522 |
| v1419 | 0.558695 |
| v1328 | 0.558942 |
| v1097 | 0.559077 |
| v1479 | 0.559106 |
| v642 | 0.560571 |
| v905 | 0.561174 |
| v514 | 0.562392 |
| v817 | 0.565385 |
| v1332 | 0.566403 |
| v1417 | 0.566976 |
| v605 | 0.567576 |
| v586 | 0.568596 |
| v1094 | 0.56879 |
| v529 | 0.57051 |
| v1227 | 0.572374 |
| v999 | 0.57299 |
| v1123 | 0.576666 |
| v1210 | 0.576822 |
| v268 | 0.577311 |
| v1204 | 0.578266 |
| v597 | 0.578623 |
| v179 | 0.580254 |
| v8 | 0.5821 |
| v65 | 0.582271 |
| v1120 | 0.586035 |
| v1048 | 0.586211 |
| v68 | 0.587098 |
| v497 | 0.587802 |
| v1258 | 0.588688 |
| v814 | 0.590017 |
| v1389 | 0.590547 |
| v88 | 0.592791 |
| v826 | 0.597054 |
| v662 | 0.598043 |
| v363 | 0.598097 |
| v1104 | 0.598579 |
| v1157 | 0.598622 |
| v1282 | 0.600327 |
| v69 | 0.603159 |
| v811 | 0.604609 |
| v1301 | 0.605035 |
| v1276 | 0.605905 |
| v892 | 0.605956 |
| v915 | 0.60612 |
| v1053 | 0.606532 |
| v606 | 0.608751 |
| v521 | 0.60933 |
| v912 | 0.609432 |
| v1383 | 0.609575 |
| v1380 | 0.609591 |
| v1180 | 0.611188 |
| v862 | 0.612168 |
| v355 | 0.61284 |
| v127 | 0.61305 |
| v229 | 0.61604 |
| v815 | 0.617459 |
| v10 | 0.617898 |
| v641 | 0.620083 |
| v1480 | 0.620481 |
| v1186 | 0.620765 |
| v66 | 0.621357 |
| v938 | 0.623154 |
| v307 | 0.624776 |
| v415 | 0.625531 |
| v922 | 0.627217 |
| v351 | 0.627641 |
| v1416 | 0.627715 |
| v698 | 0.63086 |
| v328 | 0.631135 |
| v367 | 0.632599 |
| v67 | 0.634048 |
| v441 | 0.636051 |
| v1047 | 0.636295 |
| v169 | 0.636996 |
| v290 | 0.642337 |
| v864 | 0.643661 |
| v1064 | 0.643941 |
| v936 | 0.64653 |
| v810 | 0.646673 |
| v637 | 0.646796 |
| v1072 | 0.646854 |
| v812 | 0.646915 |
| v555 | 0.646949 |
| v49 | 0.647433 |
| v1420 | 0.647554 |
| v718 | 0.648165 |
| v1303 | 0.648552 |
| v1004 | 0.649025 |
| v200 | 0.649382 |
| v746 | 0.655022 |
| v1400 | 0.655449 |
| v699 | 0.657763 |
| v1358 | 0.658001 |
| v414 | 0.658734 |
| v73 | 0.659548 |
| v1177 | 0.660308 |
| v909 | 0.664662 |
| v1147 | 0.665749 |
| v1213 | 0.665752 |
| v1413 | 0.666723 |
| v84 | 0.666734 |
| v835 | 0.668887 |
| v900 | 0.66891 |
| v1011 | 0.670364 |
| v577 | 0.670509 |
| v1444 | 0.671532 |
| v992 | 0.6719 |
| v71 | 0.672442 |
| v510 | 0.672761 |
| v106 | 0.675436 |
| v433 | 0.678674 |
| v1029 | 0.678891 |
| v241 | 0.679652 |
| v1084 | 0.681443 |
| v1384 | 0.681663 |
| v729 | 0.684448 |
| v151 | 0.684523 |
| v490 | 0.686048 |
| v553 | 0.687116 |
| v269 | 0.6908 |
| v1289 | 0.692328 |
| v1188 | 0.692393 |
| v377 | 0.693382 |
| v644 | 0.693418 |
| v1481 | 0.695174 |
| v1265 | 0.695485 |
| v1485 | 0.69641 |
| v964 | 0.697814 |
| v6 | 0.699046 |
| v487 | 0.699641 |
| v1009 | 0.700842 |
| v271 | 0.700932 |
| v1032 | 0.701397 |
| v907 | 0.701738 |
| v1468 | 0.703479 |
| v792 | 0.704286 |
| v533 | 0.704635 |
| v631 | 0.705424 |
| v413 | 0.706222 |
| v122 | 0.707635 |
| v342 | 0.707809 |
| v1132 | 0.708799 |
| v1035 | 0.708866 |
| v569 | 0.70971 |
| v996 | 0.710066 |
| v1370 | 0.710808 |
| v1203 | 0.71095 |
| v833 | 0.711097 |
| v867 | 0.711267 |
| v7 | 0.712665 |
| v794 | 0.713438 |
| v998 | 0.715751 |
| v633 | 0.716376 |
| v899 | 0.71721 |
| v1310 | 0.717796 |
| v297 | 0.718063 |
| v688 | 0.719366 |
| v485 | 0.720291 |
| v1136 | 0.725274 |
| v480 | 0.726354 |
| v166 | 0.726369 |
| v968 | 0.726759 |
| v86 | 0.728424 |
| v28 | 0.728947 |
| v266 | 0.729817 |
| v755 | 0.729845 |
| v874 | 0.72998 |
| v421 | 0.731129 |
| v880 | 0.731179 |
| v1012 | 0.731222 |
| v94 | 0.732768 |
| v1200 | 0.733475 |
| v174 | 0.733583 |
| v947 | 0.734369 |
| v283 | 0.735297 |
| v220 | 0.737139 |
| v875 | 0.737608 |
| v673 | 0.737759 |
| v682 | 0.740994 |
| v1082 | 0.741039 |
| v793 | 0.742548 |
| v571 | 0.742691 |
| v110 | 0.742969 |
| v1337 | 0.744344 |
| v1160 | 0.74502 |
| v620 | 0.746473 |
| v81 | 0.7468 |
| v382 | 0.7468 |
| v963 | 0.747487 |
| v1477 | 0.747815 |
| v757 | 0.748472 |
| v565 | 0.748658 |
| v656 | 0.749133 |
| v453 | 0.749216 |
| v31 | 0.75175 |
| v1297 | 0.752136 |
| v737 | 0.753933 |
| v1367 | 0.754063 |
| v35 | 0.754725 |
| v486 | 0.755215 |
| v1351 | 0.756106 |
| v431 | 0.756642 |
| v653 | 0.75683 |
| v937 | 0.758048 |
| v713 | 0.759543 |
| v1407 | 0.760717 |
| v705 | 0.761327 |
| v636 | 0.761887 |
| v1051 | 0.761927 |
| v934 | 0.764559 |
| v1273 | 0.76741 |
| v1465 | 0.767494 |
| v726 | 0.76773 |
| v728 | 0.768068 |
| v879 | 0.769549 |
| v1145 | 0.770572 |
| v1270 | 0.770605 |
| v1450 | 0.770616 |
| v1185 | 0.771278 |
| v1432 | 0.773549 |
| v1422 | 0.773861 |
| v1075 | 0.773878 |
| v1023 | 0.774556 |
| v1249 | 0.775798 |
| v1240 | 0.775897 |
| v1467 | 0.776705 |
| v1353 | 0.776886 |
| v1369 | 0.781851 |
| v1267 | 0.782112 |
| putamen_grey_vol | 0.782599 |
| v42 | 0.782973 |
| v850 | 0.784113 |
| v1476 | 0.784467 |
| v123 | 0.784964 |
| v945 | 0.786957 |
| v286 | 0.788699 |
| v1462 | 0.790903 |
| v346 | 0.791528 |
| v969 | 0.79169 |
| v890 | 0.791985 |
| v357 | 0.793232 |
| v278 | 0.79586 |
| v970 | 0.797074 |
| v532 | 0.797074 |
| v1330 | 0.797343 |
| v1231 | 0.79893 |
| v1028 | 0.800563 |
| v466 | 0.801434 |
| v1008 | 0.80189 |
| v1098 | 0.8035 |
| v694 | 0.804621 |
| v805 | 0.80475 |
| v459 | 0.806984 |
| v411 | 0.808047 |
| v578 | 0.809031 |
| v163 | 0.80964 |
| v667 | 0.811047 |
| v437 | 0.811435 |
| v1209 | 0.8117 |
| v976 | 0.812204 |
| v147 | 0.812302 |
| v30 | 0.812321 |
| v1371 | 0.812478 |
| v1218 | 0.813348 |
| v703 | 0.814524 |
| v537 | 0.814796 |
| v436 | 0.816388 |
| v379 | 0.817111 |
| v1278 | 0.817247 |
| v58 | 0.820099 |
| v1060 | 0.821425 |
| v1197 | 0.821939 |
| v1309 | 0.822767 |
| v390 | 0.82403 |
| v1194 | 0.824408 |
| v1306 | 0.827809 |
| v704 | 0.828804 |
| v984 | 0.828809 |
| v463 | 0.829181 |
| v584 | 0.829905 |
| v884 | 0.830012 |
| v118 | 0.831105 |
| v439 | 0.83293 |
| v111 | 0.833859 |
| v499 | 0.833945 |
| v320 | 0.835415 |
| v505 | 0.836164 |
| v622 | 0.83757 |
| v625 | 0.83893 |
| v730 | 0.839136 |
| v601 | 0.840154 |
| v1126 | 0.841648 |
| v48 | 0.841961 |
| v406 | 0.843437 |
| v753 | 0.84544 |
| v417 | 0.845512 |
| v585 | 0.845942 |
| v471 | 0.84637 |
| v273 | 0.846938 |
| v450 | 0.847829 |
| v1322 | 0.848059 |
| v149 | 0.849224 |
| v564 | 0.850968 |
| v1442 | 0.851294 |
| v802 | 0.85323 |
| v751 | 0.853328 |
| v888 | 0.853376 |
| v180 | 0.853481 |
| v132 | 0.854902 |
| v975 | 0.855222 |
| v1112 | 0.855563 |
| v279 | 0.856717 |
| v1222 | 0.85741 |
| v911 | 0.85833 |
| v302 | 0.859646 |
| v1326 | 0.859969 |
| v85 | 0.860127 |
| v423 | 0.860642 |
| v821 | 0.861212 |
| v1377 | 0.862863 |
| v347 | 0.86292 |
| v1398 | 0.8636 |
| v428 | 0.863981 |
| v91 | 0.864076 |
| v773 | 0.867126 |
| v1250 | 0.868538 |
| v621 | 0.86921 |
| v1421 | 0.872364 |
| v410 | 0.872879 |
| v592 | 0.873393 |
| v1266 | 0.875124 |
| v738 | 0.875957 |
| v1154 | 0.87746 |
| v407 | 0.879404 |
| v988 | 0.879711 |
| v1474 | 0.88276 |
| v1478 | 0.885509 |
| v731 | 0.88597 |
| v1348 | 0.88623 |
| v1410 | 0.887366 |
| v51 | 0.887642 |
| v747 | 0.887877 |
| v495 | 0.888 |
| v370 | 0.88829 |
| v574 | 0.888309 |
| v1055 | 0.888485 |
| v669 | 0.88852 |
| v926 | 0.889095 |
| v24 | 0.889625 |
| v851 | 0.890719 |
| v432 | 0.891194 |
| v1040 | 0.891359 |
| v293 | 0.891754 |
| v786 | 0.892762 |
| v1329 | 0.894924 |
| v1199 | 0.89595 |
| v496 | 0.896288 |
| v272 | 0.898226 |
| v1232 | 0.898374 |
| v333 | 0.898741 |
| v765 | 0.900251 |
| v559 | 0.903622 |
| v1312 | 0.903834 |
| v107 | 0.904661 |
| v1430 | 0.906933 |
| v334 | 0.907058 |
| v1107 | 0.90768 |
| v449 | 0.909254 |
| v979 | 0.909499 |
| v153 | 0.911074 |
| v1115 | 0.914225 |
| v371 | 0.915023 |
| v397 | 0.916408 |
| v1196 | 0.917178 |
| v1135 | 0.918024 |
| v1178 | 0.919207 |
| v210 | 0.919785 |
| v364 | 0.920571 |
| v801 | 0.921019 |
| v1350 | 0.921847 |
| v195 | 0.92303 |
| v1362 | 0.925833 |
| v525 | 0.926791 |
| v207 | 0.927719 |
| v331 | 0.931614 |
| v652 | 0.931973 |
| v358 | 0.932221 |
| v528 | 0.932477 |
| v1292 | 0.932655 |
| v1482 | 0.932839 |
| v366 | 0.935568 |
| v526 | 0.937929 |
| v372 | 0.938315 |
| v256 | 0.93851 |
| v39 | 0.938664 |
| v1246 | 0.94408 |
| v1406 | 0.94439 |
| v675 | 0.949114 |
| v1352 | 0.949145 |
| v1114 | 0.950027 |
| v1313 | 0.95192 |
| v701 | 0.953497 |
| v1281 | 0.953651 |
| v1342 | 0.95421 |
| v539 | 0.954552 |
| v827 | 0.955437 |
| v941 | 0.956928 |
| v192 | 0.957063 |
| v852 | 0.958334 |
| v1451 | 0.959261 |
| v408 | 0.959397 |
| v733 | 0.960237 |
| v1220 | 0.961097 |
| v443 | 0.963922 |
| v679 | 0.966235 |
| v666 | 0.966727 |
| v939 | 0.969223 |
| v626 | 0.971395 |
| v1295 | 0.971602 |
| v1219 | 0.972721 |
| v4 | 0.97333 |
| v803 | 0.973742 |
| v589 | 0.973765 |
| v717 | 0.97401 |
| v1302 | 0.97575 |
| v985 | 0.978758 |
| v326 | 0.979791 |
| v1434 | 0.979922 |
| v776 | 0.981412 |
| v284 | 0.982146 |
| v435 | 0.983174 |
| v476 | 0.983319 |
| v1191 | 0.987478 |
| v544 | 0.987755 |
| v671 | 0.987782 |
| v1206 | 0.988319 |
| v723 | 0.989772 |
| v944 | 0.98989 |
| v725 | 0.99182 |
| v393 | 0.996008 |
| v1251 | 0.996102 |
| v949 | 0.996192 |
| v1036 | 0.996263 |
| v1070 | 0.997501 |

**Supplementary Table 3.** Pairwise correlations between variables to assess multicollinearity.

**| depress bmi lonely~l s1_gre~l insula~l amygda~l m1_gre~l**

-------------+---------------------------------------------------------------

depress | 1.0000

bmi | 0.1125 1.0000

lonely_soc~l | 0.2873 0.0966 1.0000

s1_grey_vol | 0.0233 -0.0011 -0.0139 1.0000

insula_gre~l | -0.0128 -0.0013 -0.0055 0.4570 1.0000

amygdala_g~l | 0.0070 -0.0512 -0.0065 0.4356 0.5875 1.0000

m1_grey_vol | 0.0031 0.0042 -0.0076 0.6582 0.5435 0.4834 1.0000

mfc_grey_vol | -0.0011 0.0228 -0.0088 0.3840 0.4264 0.3479 0.4286

v849 | 0.0261 0.0390 0.0196 -0.0947 0.0613 0.0283 0.0170

v380 | 0.0254 0.0513 0.0234 -0.1223 0.0019 -0.0275 0.0059

v135 | 0.0006 0.0582 0.0065 -0.0956 -0.0655 -0.1097 -0.0983

v1183 | -0.0080 0.0526 -0.0018 -0.0662 -0.0539 -0.0734 -0.0521

v20 | 0.0044 -0.0511 -0.0158 0.0599 -0.0850 -0.0241 -0.0241

v1143 | 0.0101 0.0925 0.0027 -0.0733 -0.0234 -0.0599 -0.0423

v145 | 0.0058 0.0424 0.0051 -0.0552 -0.0213 -0.0264 0.0116

hippo_grey~l | 0.0044 0.0240 -0.0011 0.4161 0.5669 0.6634 0.4554

v1357 | 0.0244 0.0178 0.0164 0.0293 -0.0472 -0.0195 -0.0029

s2_grey_vol | -0.0050 0.0402 -0.0047 0.4128 0.5860 0.4435 0.4609

v1148 | 0.0075 0.0242 0.0082 -0.0444 -0.0238 -0.0080 -0.0114

v222 | -0.0241 -0.0160 -0.0148 0.0281 0.0250 0.0485 0.0167

v930 | 0.0185 0.0390 0.0102 -0.0943 -0.0554 -0.1011 -0.0864

v142 | 0.0249 -0.0121 0.0189 -0.0263 -0.0374 -0.0121 -0.0072

v1140 | 0.0002 0.0883 0.0091 -0.0865 -0.0140 -0.0750 -0.0168

v262 | -0.0078 -0.0278 -0.0112 0.0617 0.0034 0.0224 0.0019

frontorbit~l | 0.0071 0.0496 -0.0061 0.4907 0.6732 0.5671 0.5687

v93 | 0.0052 0.0708 0.0024 0.0292 -0.0021 0.0293 0.0051

v838 | 0.0061 -0.0229 -0.0106 0.0816 0.0354 0.0359 0.0582

v44 | 0.0194 0.0027 -0.0066 0.0095 0.0199 0.0255 0.0299

acc_grey_vol | -0.0175 0.0414 0.0044 0.2328 0.3758 0.2662 0.3063

v789 | 0.0011 0.0311 -0.0089 -0.0287 -0.0477 -0.0150 -0.0215

v1253 | -0.0168 -0.0292 -0.0119 0.0079 0.0019 0.0129 0.0305

v1091 | 0.0179 0.0479 0.0128 0.0037 -0.0209 0.0029 0.0087

v102 | -0.0042 0.1068 0.0238 -0.0751 0.0524 -0.0258 0.0267

v458 | 0.0225 0.0144 0.0105 -0.0208 -0.0461 -0.0391 -0.0374

v404 | 0.0097 0.0406 0.0120 -0.1111 0.0189 -0.0378 0.0202

v464 | 0.0012 0.0082 0.0064 0.0740 -0.0128 0.0007 0.0299

v1165 | -0.0073 -0.0325 -0.0162 0.1023 -0.0209 0.0041 -0.0390

v312 | 0.0104 -0.0353 -0.0191 0.1306 0.0664 0.1039 0.1059

v732 | 0.0220 0.0095 0.0092 -0.1724 -0.0424 -0.0444 -0.0612

v581 | 0.0333 0.0006 0.0118 -0.0327 -0.0448 -0.0579 -0.0389

v201 | -0.0015 -0.0279 -0.0080 0.0434 0.1183 0.0767 0.0655

v319 | -0.0048 -0.0181 -0.0155 0.0636 0.0137 0.0130 0.0061

v164 | -0.0209 0.0475 0.0055 -0.1510 -0.0711 -0.0817 -0.0780

v1286 | -0.0002 0.0183 0.0041 -0.0123 -0.0287 -0.0173 -0.0188

v967 | -0.0106 0.0485 0.0025 -0.0562 0.0208 0.0002 -0.0092

v740 | 0.0009 0.0456 -0.0011 -0.1133 -0.0971 -0.0690 -0.0715

v1137 | 0.0211 0.0247 -0.0051 -0.1184 -0.0503 -0.0395 -0.0460

v369 | 0.0129 0.0094 -0.0143 -0.0676 -0.0460 -0.0725 -0.0561

v1207 | -0.0011 0.0136 -0.0089 0.0449 0.0947 0.0730 0.0418

v373 | -0.0238 0.0024 -0.0118 -0.0161 -0.0066 -0.0394 -0.0423

v745 | -0.0196 -0.0081 -0.0308 -0.0476 0.0487 0.0385 -0.0268

v547 | 0.0119 0.0513 0.0099 -0.0115 0.0198 0.0157 -0.0118

v171 | 0.0009 -0.0244 -0.0018 0.0768 0.0438 0.0426 0.0398

v144 | -0.0009 -0.0182 -0.0044 0.0777 0.0194 0.0265 0.0378

v344 | -0.0098 -0.0173 0.0111 -0.0064 -0.0173 -0.0254 -0.0235

v629 | -0.0102 0.0626 -0.0083 -0.0633 0.0517 -0.0151 0.0170

v524 | -0.0076 -0.0089 -0.0126 0.0095 -0.0168 -0.0045 -0.0140

v337 | 0.0003 0.0315 0.0074 -0.0707 -0.0243 -0.0265 -0.0264

v141 | -0.0109 0.0465 0.0023 -0.0833 -0.0595 -0.0534 -0.0249

v1408 | 0.0037 -0.0090 0.0035 0.0022 0.0078 0.0090 -0.0011

v614 | 0.0048 -0.0166 -0.0068 0.0856 -0.0172 -0.0088 -0.0062

v1172 | 0.0045 -0.0369 -0.0014 0.0779 -0.0108 -0.0110 -0.0030

v47 | -0.0006 -0.0537 -0.0242 0.0421 -0.0200 0.0182 -0.0155

v788 | 0.0206 -0.0105 0.0115 -0.0319 -0.0522 -0.0545 -0.0549

v329 | -0.0042 -0.0290 -0.0199 0.0054 0.0084 0.0321 0.0097

v961 | 0.0085 -0.0120 -0.0038 0.0583 0.1644 0.0993 0.0958

v608 | 0.0078 0.0127 -0.0131 -0.0620 -0.0707 -0.0443 -0.0734

v820 | -0.0052 -0.0211 0.0018 0.0279 0.0713 0.0743 0.0533

sma_grey_vol | -0.0053 0.0367 -0.0039 0.3182 0.4154 0.3401 0.4402

v1076 | -0.0119 -0.0385 -0.0000 0.0133 -0.0251 -0.0051 -0.0117

v953 | 0.0186 -0.0496 0.0037 0.0520 -0.0751 0.0207 -0.0128

v1321 | 0.0077 0.0311 0.0169 -0.0429 -0.0282 -0.0360 -0.0327

v1133 | -0.0107 0.0931 0.0145 -0.0736 0.0538 -0.0007 0.0468

v804 | 0.0084 0.0561 0.0182 -0.0250 0.0198 -0.0019 -0.0204

v861 | 0.0114 0.0559 0.0005 0.0055 -0.0015 0.0094 -0.0092

v420 | -0.0049 -0.0087 -0.0056 0.0271 0.0245 0.0436 0.0312

v162 | -0.0121 -0.0554 0.0021 -0.0002 -0.0157 0.0144 0.0008

v639 | 0.0056 0.0904 0.0238 0.0100 0.0315 -0.0080 0.0223

v630 | -0.0018 0.0147 -0.0020 -0.0315 -0.0390 -0.0452 -0.0294

v940 | -0.0153 -0.0178 -0.0129 0.0621 0.0815 0.0935 0.0372

v1141 | 0.0082 0.0347 -0.0005 -0.0574 0.0054 -0.0021 0.0050

v1096 | 0.0049 0.0212 0.0033 -0.0466 -0.0533 -0.0590 -0.0635

v1174 | 0.0091 0.0293 0.0065 -0.0119 0.0089 -0.0219 0.0283

v291 | -0.0064 0.0504 0.0049 -0.0526 -0.0588 -0.0436 -0.0109

v1083 | -0.0112 -0.0176 -0.0012 0.0638 0.0077 0.0164 0.0319

v554 | -0.0111 0.0256 0.0078 -0.0698 -0.0875 -0.0718 -0.0716

v1102 | 0.0151 0.0195 0.0020 -0.0942 -0.0570 -0.0507 -0.0749

v758 | 0.0162 0.0290 0.0045 -0.0239 0.0152 -0.0210 0.0062

v23 | -0.0175 0.0364 -0.0072 0.0151 0.0122 -0.0095 0.0124

v1294 | -0.0111 -0.0159 -0.0032 0.0099 0.0138 0.0189 0.0107

v1014 | 0.0012 0.0422 0.0179 -0.0042 -0.0110 -0.0360 0.0158

v1288 | 0.0011 0.0097 -0.0156 0.0522 0.0188 0.0178 0.0341

v1193 | -0.0116 -0.0206 -0.0131 0.0030 -0.0249 -0.0222 -0.0240

v143 | 0.0341 0.0131 0.0211 -0.0873 -0.0230 -0.0165 -0.0023

v648 | -0.0074 -0.0080 0.0002 0.0982 0.0413 0.0427 0.0483

v57 | -0.0043 -0.0148 0.0048 -0.0121 -0.0161 -0.0155 0.0085

v567 | -0.0408 0.0211 -0.0087 0.0199 0.0533 0.0385 0.0615

v783 | 0.0238 0.0480 -0.0031 -0.0042 -0.0578 -0.0076 -0.0121

grip_str_h~h | -0.0604 0.0911 -0.0096 0.1957 0.3664 0.3537 0.2977

v546 | -0.0263 -0.0264 -0.0077 -0.0097 -0.0279 -0.0366 -0.0117

**| mfc_gr~l v849 v380 v135 v1183 v20 v1143**

-------------+---------------------------------------------------------------

mfc_grey_vol | 1.0000

v849 | 0.0227 1.0000

v380 | 0.0018 0.2579 1.0000

v135 | -0.0805 -0.0089 -0.0058 1.0000

v1183 | -0.0297 0.0054 0.0555 0.0491 1.0000

v20 | -0.0226 -0.0503 -0.0701 -0.0624 -0.1059 1.0000

v1143 | -0.0307 0.0794 0.1073 0.0953 0.0474 -0.0535 1.0000

v145 | -0.0096 0.1058 0.1652 0.0074 0.0444 -0.0582 0.0357

hippo_grey~l | 0.3293 0.0062 -0.0487 -0.0661 -0.0452 -0.0581 -0.0343

v1357 | 0.0087 -0.0157 -0.0250 -0.0297 0.0322 0.0561 -0.0320

s2_grey_vol | 0.3392 -0.0336 -0.1118 -0.0548 -0.0463 -0.0734 -0.0735

v1148 | 0.0062 0.0208 0.0557 0.0124 0.0163 -0.0367 0.0106

v222 | 0.0210 -0.0110 -0.0531 -0.0248 -0.0409 0.0623 -0.0081

v930 | -0.0665 0.0460 0.0821 0.0574 0.0697 -0.1062 0.0448

v142 | -0.0078 0.0567 0.0686 -0.0224 0.0010 0.0140 0.0030

v1140 | -0.0233 0.0863 0.1596 0.0467 0.0438 -0.0923 0.1363

v262 | 0.0364 -0.0523 -0.0658 -0.0408 -0.0393 0.0567 -0.0388

frontorbit~l | 0.3999 0.0325 -0.0187 -0.0794 -0.0888 -0.0860 -0.0396

v93 | 0.0067 -0.0264 0.0480 -0.0298 0.0768 -0.0741 0.0486

v838 | 0.0298 -0.0620 -0.0455 -0.0429 -0.0640 0.0596 -0.0713

v44 | 0.0201 0.0900 0.0868 -0.0145 0.0098 -0.0303 -0.0084

acc_grey_vol | 0.1325 0.0445 0.0191 0.0158 -0.0236 -0.0405 0.0473

v789 | -0.0010 0.0758 0.0865 0.0102 0.0087 0.0021 0.0343

v1253 | 0.0125 -0.0127 -0.0412 -0.0272 -0.0283 0.0184 -0.0011

v1091 | 0.0000 -0.0523 -0.0632 0.0078 0.0161 -0.0278 0.0153

v102 | 0.0117 0.1315 0.2084 0.0286 0.0737 -0.1277 0.1805

v458 | -0.0267 0.0151 0.0167 -0.0086 0.0273 -0.0037 0.0099

v404 | -0.0080 0.1697 0.1959 0.0020 0.0575 -0.1085 0.0900

v464 | -0.0004 -0.2061 -0.1980 -0.0040 0.0082 -0.0239 -0.0286

v1165 | 0.0004 -0.1152 -0.1535 -0.0439 -0.0486 0.1195 -0.0191

v312 | 0.0462 -0.0449 -0.1105 -0.0377 -0.0679 0.0699 -0.0563

v732 | -0.0441 0.2018 0.2302 0.0037 0.0187 -0.0572 0.0591

v581 | -0.0235 0.0518 0.0859 0.0147 0.0317 -0.0203 -0.0020

v201 | 0.0671 0.0760 0.0622 -0.0545 -0.0578 0.0534 0.0152

v319 | 0.0022 -0.0261 -0.0764 -0.0303 -0.0336 0.0384 -0.0101

v164 | -0.0484 0.0808 0.1535 0.0300 0.0739 -0.0422 0.0826

v1286 | -0.0159 0.0103 0.0258 0.0171 -0.0156 0.0265 0.0253

v967 | -0.0376 0.0081 0.0186 0.0468 0.0304 -0.0951 0.0946

v740 | -0.0639 0.0577 0.0416 0.0035 0.0258 -0.0393 0.0495

v1137 | -0.0447 0.1066 0.1558 0.0219 0.0611 -0.1023 -0.0061

v369 | -0.0481 0.0109 0.0524 0.0301 0.0477 -0.0564 0.0264

v1207 | 0.0440 -0.0244 -0.0338 -0.0334 -0.1505 0.0501 -0.0374

v373 | -0.0052 -0.0375 -0.0082 0.0208 0.0312 -0.0637 0.0423

v745 | 0.0082 0.0236 0.0080 -0.0047 0.0051 -0.0516 0.0355

v547 | 0.0116 -0.0144 -0.0424 0.0115 -0.0123 -0.0057 0.0171

v171 | 0.0387 -0.0158 -0.0495 -0.0389 -0.0143 0.0448 -0.0312

v144 | 0.0231 -0.0608 -0.0644 -0.0218 -0.0284 0.0212 -0.0400

v344 | 0.0044 -0.0339 -0.0508 0.0208 -0.0029 -0.0347 0.0161

v629 | 0.0073 0.0364 0.0613 0.0324 0.0327 -0.0676 0.0583

v524 | 0.0002 -0.0366 0.0402 -0.0102 0.0088 0.0185 0.0164

v337 | -0.0114 0.0466 0.1093 0.0474 0.0527 -0.0891 0.0674

v141 | -0.0252 0.0462 0.1243 0.0394 0.0465 -0.0540 0.0398

v1408 | 0.0153 -0.0064 0.0295 -0.0040 -0.0002 0.0039 -0.0105

v614 | 0.0094 -0.0616 -0.0466 -0.0124 -0.0110 0.0892 -0.0408

v1172 | 0.0252 -0.0697 -0.0655 -0.0278 0.0048 0.1323 -0.1089

v47 | -0.0021 -0.0119 -0.0380 -0.0448 -0.0081 0.0420 -0.0173

v788 | -0.0374 -0.0173 -0.1029 -0.0112 -0.0337 0.0231 0.0143

v329 | 0.0159 -0.0189 -0.0107 -0.0394 -0.0255 -0.0066 -0.0274

v961 | 0.0784 0.0936 0.0431 -0.0626 -0.1056 0.1278 -0.0416

v608 | -0.0399 0.0402 0.0752 0.0256 0.0738 -0.0437 0.0323

v820 | 0.0495 0.0382 0.0100 -0.0224 -0.0238 -0.0031 -0.0212

sma_grey_vol | 0.3087 0.0387 -0.0001 -0.0485 -0.0283 -0.0394 -0.0323

v1076 | 0.0155 -0.0473 -0.0493 -0.0219 -0.0247 0.0452 -0.0020

v953 | 0.0158 -0.0668 -0.0353 -0.0688 -0.1345 0.0798 -0.0608

v1321 | -0.0490 -0.0010 0.0102 0.0013 0.0406 -0.0285 0.0210

v1133 | 0.0187 0.0870 0.0577 0.0483 0.0570 -0.2211 -0.0127

v804 | 0.0093 0.0563 -0.0118 -0.0007 -0.0142 0.0055 0.0613

v861 | -0.0093 -0.0345 0.0631 0.0117 0.0333 -0.0334 -0.0079

v420 | 0.0249 -0.0056 0.0117 -0.0454 -0.0134 0.0111 -0.0114

v162 | -0.0133 -0.0463 -0.1521 -0.0389 -0.0660 0.0684 -0.0334

v639 | 0.0195 0.0415 0.0588 0.0322 0.0247 -0.1485 0.0739

v630 | -0.0360 0.0205 0.0376 0.0266 0.0112 -0.0102 0.0341

v940 | 0.0723 -0.0288 -0.0535 -0.0294 -0.0733 0.0648 -0.0413

v1141 | -0.0127 0.1057 0.0542 0.0181 -0.0034 -0.0943 -0.0466

v1096 | 0.0010 0.0195 0.0408 0.0598 0.0390 -0.0244 0.1509

v1174 | 0.0066 0.1014 0.0859 0.0125 0.0363 -0.0680 0.0605

v291 | -0.0274 0.0113 0.0484 -0.0249 0.0206 -0.0477 0.1118

v1083 | 0.0205 -0.1307 -0.1218 0.0163 -0.0290 -0.0182 -0.0369

v554 | -0.0426 0.0211 0.0332 0.0261 0.0335 0.0240 0.0213

v1102 | -0.0381 0.1453 0.1692 0.0170 0.0017 -0.0165 0.0603

v758 | -0.0079 0.0176 0.0447 0.1413 0.0245 -0.0122 0.0062

v23 | 0.0060 -0.0495 -0.0439 0.0252 0.0149 -0.0159 0.0328

v1294 | 0.0047 -0.0115 -0.0557 0.0212 -0.0047 -0.0025 -0.0042

v1014 | -0.0357 -0.0569 -0.0820 0.0297 0.0112 -0.0148 -0.0227

v1288 | 0.0135 -0.0479 -0.0368 -0.0171 0.0021 0.0206 -0.0376

v1193 | -0.0061 -0.0317 -0.0307 -0.0253 0.0135 0.0214 -0.0219

v143 | -0.0143 0.0613 0.1172 0.0090 0.0114 -0.0629 0.0196

v648 | 0.0375 -0.0620 -0.0536 -0.0306 -0.0211 0.0950 -0.0426

v57 | 0.0056 -0.0193 -0.0952 0.0278 0.0037 -0.0155 0.0270

v567 | 0.0327 -0.0220 -0.0134 0.0095 0.0065 -0.0632 0.0548

v783 | -0.0095 0.0355 0.0296 -0.0411 0.0031 0.0307 -0.0005

grip_str_h~h | 0.2502 0.0113 -0.0069 -0.0473 -0.0187 -0.1395 0.0446

v546 | -0.0305 -0.0072 0.0143 0.0364 0.0162 -0.0361 0.0254

**| v145 hippo_~l v1357 s2_gre~l v1148 v222 v930**

-------------+---------------------------------------------------------------

v145 | 1.0000

hippo_grey~l | -0.0313 1.0000

v1357 | -0.0301 -0.0307 1.0000

s2_grey_vol | -0.0255 0.4167 -0.0358 1.0000

v1148 | 0.0274 -0.0085 0.0088 -0.0088 1.0000

v222 | -0.0804 0.0469 0.0319 -0.0281 0.0058 1.0000

v930 | 0.0849 -0.0765 -0.0397 -0.0730 0.0073 -0.0723 1.0000

v142 | 0.0757 -0.0425 0.0250 -0.0223 0.0142 -0.0217 0.0191

v1140 | 0.1036 -0.0659 -0.0308 -0.0325 0.0662 -0.1105 0.0837

v262 | -0.0344 0.0143 0.0291 0.0319 -0.0270 0.0204 -0.0498

frontorbit~l | -0.0073 0.5239 -0.0385 0.5399 -0.0052 0.0311 -0.0858

v93 | 0.1238 0.0173 -0.0285 0.0827 0.0014 -0.0760 0.0619

v838 | -0.1002 0.0423 0.0238 0.0271 0.0358 0.0627 -0.0684

v44 | 0.0263 0.0119 -0.0126 0.0138 0.0013 -0.0271 0.0227

acc_grey_vol | -0.0379 0.2628 -0.0349 0.2580 -0.0232 0.0117 -0.0261

v789 | 0.0670 -0.0312 -0.0149 -0.0576 0.0199 -0.0323 0.0087

v1253 | -0.0244 0.0024 0.0072 0.0210 0.0005 0.0144 -0.0367

v1091 | 0.0036 0.0177 0.0257 0.0214 0.0296 -0.0066 -0.0025

v102 | 0.0749 0.0018 -0.0948 0.0154 0.0487 -0.0200 0.0686

v458 | 0.0294 -0.0416 0.0593 -0.0321 0.0066 0.0066 0.0040

v404 | 0.0997 -0.0163 -0.0275 -0.0428 0.0497 -0.0150 0.0820

v464 | -0.0579 0.0225 -0.0295 0.0972 0.0002 -0.0015 -0.0344

v1165 | -0.0649 -0.0104 0.0487 0.0098 -0.1817 -0.0019 -0.0226

v312 | -0.1228 0.0868 0.0397 0.0738 -0.0159 0.0168 -0.1103

v732 | 0.0799 -0.0733 -0.0071 -0.0979 0.0766 0.0434 0.0405

v581 | 0.0840 -0.0636 0.0033 -0.0470 0.0119 -0.0790 0.1309

v201 | 0.0039 0.0539 -0.0171 0.0190 -0.0072 0.0244 -0.0003

v319 | -0.0437 0.0123 0.0244 0.0017 -0.0249 0.0247 -0.0242

v164 | 0.0804 -0.0847 -0.0349 -0.1021 0.0349 -0.0102 0.0455

v1286 | -0.0103 -0.0193 0.0052 -0.0323 -0.0042 -0.0004 0.0096

v967 | 0.0382 0.0244 -0.0575 0.0252 0.0114 -0.0297 0.0305

v740 | 0.0800 -0.0695 0.0029 -0.0793 0.0044 -0.0116 0.0340

v1137 | 0.0742 -0.0191 -0.0183 -0.0758 0.1351 -0.0120 0.0372

v369 | 0.1937 -0.0608 -0.0418 -0.0344 0.0077 -0.0594 0.0561

v1207 | -0.0472 0.0812 -0.0178 0.0670 -0.0142 0.0209 -0.0592

v373 | 0.0317 -0.0143 -0.0356 0.0262 0.0155 -0.0124 0.0307

v745 | -0.0109 0.0295 -0.0060 0.0260 0.0121 0.0138 -0.0079

v547 | -0.0183 0.0186 0.0162 0.0383 0.0073 0.0143 -0.0144

v171 | 0.0420 0.0355 0.0316 0.0430 -0.0128 0.0189 -0.0060

v144 | -0.0669 0.0383 -0.0096 0.0335 -0.0109 0.0267 -0.0557

v344 | -0.0263 -0.0071 0.0008 0.0082 0.0209 0.0175 0.0284

v629 | 0.0522 0.0096 -0.0289 0.0195 0.0062 -0.0494 0.0512

v524 | 0.0060 -0.0142 0.0182 0.0180 -0.0066 -0.0067 0.0185

v337 | 0.0919 -0.0250 -0.0268 -0.0347 0.0201 -0.0947 0.1079

v141 | 0.1026 -0.0545 -0.0248 -0.0514 0.0347 -0.0250 0.0413

v1408 | 0.0134 -0.0021 -0.0031 0.0159 0.0075 -0.0149 0.0112

v614 | -0.0379 -0.0009 0.0036 0.0138 -0.0226 0.0178 -0.0353

v1172 | -0.0439 -0.0369 0.0076 -0.0415 -0.0911 0.0149 0.0250

v47 | 0.0378 -0.0059 0.0173 -0.0016 -0.0326 -0.0627 0.0057

v788 | -0.0965 -0.0413 0.0564 -0.0331 0.0313 0.0592 -0.0197

v329 | 0.0032 0.0262 -0.0139 0.0077 0.0058 -0.0123 -0.0405

v961 | -0.0249 0.0765 -0.0340 0.0394 -0.0130 0.0105 -0.0410

v608 | 0.0603 -0.0464 -0.0175 -0.0707 0.0228 -0.0255 0.0638

v820 | 0.0203 0.0560 0.0058 0.0686 -0.0078 0.0036 -0.0447

sma_grey_vol | 0.0015 0.3201 -0.0207 0.3264 -0.0205 0.0051 -0.0283

v1076 | -0.0344 -0.0122 0.0124 -0.0098 0.0041 0.0422 -0.0257

v953 | -0.0402 -0.0180 0.0932 -0.0179 0.0004 0.0372 -0.0709

v1321 | 0.0215 -0.0263 0.0080 -0.0155 0.0110 -0.0274 0.0651

v1133 | 0.0576 0.0289 -0.0671 0.0947 0.0880 -0.0417 0.0195

v804 | -0.0274 0.0059 0.0102 0.0289 0.0241 0.0443 -0.0329

v861 | 0.0678 -0.0031 -0.0088 0.0144 -0.0262 -0.0506 0.0479

v420 | -0.0125 0.0262 -0.0023 0.0230 -0.0117 0.0111 -0.0199

v162 | -0.1434 0.0081 0.0490 -0.0159 0.0169 0.0976 -0.0772

v639 | 0.0797 0.0094 -0.0348 0.0328 0.0317 -0.0443 0.0409

v630 | 0.0197 -0.0478 -0.0103 -0.0566 -0.0031 0.0071 0.0324

v940 | -0.0536 0.0820 -0.0134 0.0983 -0.0027 0.0327 -0.1382

v1141 | 0.0362 0.0052 -0.0344 0.0048 0.0596 -0.0179 -0.0085

v1096 | 0.0444 -0.0803 -0.0092 -0.0626 -0.0015 -0.0593 0.0484

v1174 | 0.0538 -0.0246 -0.0008 -0.0165 0.0631 -0.0194 0.0146

v291 | 0.0554 -0.0247 0.0226 -0.0111 0.0274 -0.0365 0.0119

v1083 | 0.0001 0.0198 0.0191 0.0837 -0.0225 -0.0195 -0.0279

v554 | 0.0356 -0.0758 -0.0037 -0.0732 0.0289 0.0299 0.0261

v1102 | 0.0799 -0.0672 -0.0318 -0.1066 0.0259 -0.0312 0.0344

v758 | 0.0295 -0.0006 -0.0353 -0.0031 0.0002 -0.0218 0.0172

v23 | 0.0035 -0.0021 -0.0042 0.0294 0.0010 0.0079 -0.0038

v1294 | -0.0243 0.0240 -0.0049 0.0353 0.0161 0.0069 -0.0104

v1014 | -0.0061 0.0048 0.0095 -0.0082 -0.0130 0.0063 0.0166

v1288 | 0.0206 0.0190 0.0272 0.0506 -0.0123 -0.0117 -0.0106

v1193 | -0.0171 -0.0136 0.0022 -0.0148 -0.0043 0.0111 -0.0085

v143 | 0.0094 -0.0227 -0.0388 -0.0334 0.0488 -0.0157 0.0351

v648 | -0.1599 0.0236 0.0426 0.0283 -0.0202 0.0720 -0.0234

v57 | -0.0408 -0.0209 -0.0159 0.0187 0.0020 0.0864 -0.0106

v567 | -0.0072 0.0559 -0.0390 0.0490 0.0099 -0.0006 -0.0218

v783 | 0.0570 -0.0132 0.0296 -0.0527 -0.0067 -0.0106 0.0154

grip_str_h~h | -0.0335 0.3569 -0.0735 0.3498 0.0236 0.0335 -0.0687

v546 | -0.0221 -0.0232 -0.0280 -0.0338 0.0214 -0.0007 0.0137

**| v142 v1140 v262 fronto~l v93 v838 v44**

-------------+---------------------------------------------------------------

v142 | 1.0000

v1140 | 0.0225 1.0000

v262 | 0.0089 -0.0574 1.0000

frontorbit~l | -0.0193 -0.0215 0.0058 1.0000

v93 | 0.0688 0.1222 0.0360 0.0294 1.0000

v838 | -0.0369 -0.0756 0.0010 0.0511 -0.0924 1.0000

v44 | 0.0352 0.0124 -0.0025 0.0183 0.0665 0.0249 1.0000

acc_grey_vol | -0.0280 0.0287 0.0017 0.3111 -0.0755 -0.0157 0.0192

v789 | 0.0293 0.0779 -0.0090 -0.0229 0.0962 -0.0462 0.0761

v1253 | -0.0136 -0.0196 0.0173 0.0007 -0.0588 0.0032 -0.0251

v1091 | 0.0099 0.0154 0.0420 0.0222 0.0409 -0.0111 -0.0266

v102 | -0.0031 0.1427 -0.0318 0.0098 0.1432 -0.0764 0.0612

v458 | 0.0241 0.0004 0.0013 -0.0105 0.0254 0.0130 -0.0108

v404 | 0.0509 0.0704 -0.0610 -0.0082 -0.0002 -0.0587 0.0578

v464 | -0.0446 -0.0611 0.0263 0.0262 0.0296 0.0257 -0.0384

v1165 | 0.0145 -0.0545 0.0818 -0.0279 0.0611 -0.0642 -0.0242

v312 | -0.0362 -0.1780 0.0369 0.0872 -0.1422 0.1581 0.0272

v732 | 0.0308 0.0603 -0.0665 -0.0433 -0.0724 -0.0124 0.0342

v581 | 0.1351 0.0600 0.0210 -0.0386 0.1370 -0.0008 0.0787

v201 | 0.0022 0.0064 0.0495 0.0563 -0.0813 0.0481 0.0462

v319 | 0.0015 -0.0223 0.0122 0.0038 -0.0096 0.0293 0.0087

v164 | 0.0154 0.1069 -0.0167 -0.0959 0.0908 -0.0775 0.0135

v1286 | -0.0138 0.0225 0.0200 -0.0397 -0.0124 -0.0136 -0.0356

v967 | -0.0090 0.0963 -0.0449 0.0257 0.0830 -0.0769 -0.0234

v740 | 0.0226 0.0794 -0.0238 -0.0529 0.0457 -0.0645 -0.0238

v1137 | 0.0326 0.0342 -0.1313 -0.0248 0.0284 0.0807 0.0413

v369 | 0.0564 0.0848 0.0062 -0.0287 0.0839 -0.1477 0.0163

v1207 | -0.0297 -0.0339 0.0313 0.0904 -0.0601 0.0395 -0.0091

v373 | -0.0175 0.0709 0.0164 -0.0098 0.1065 -0.0584 -0.0203

v745 | -0.0097 0.0070 0.0239 0.0316 -0.0031 0.0214 0.0610

v547 | 0.0118 0.0041 0.0327 0.0139 0.0169 -0.0056 -0.0204

v171 | -0.0479 -0.0292 0.0146 0.0411 -0.0086 -0.0051 -0.0108

v144 | -0.0431 -0.0580 0.0016 0.0389 -0.0225 0.0768 -0.0288

v344 | -0.0249 -0.0035 -0.0106 -0.0207 -0.0405 0.0108 -0.0425

v629 | 0.0051 0.1200 0.0063 0.0280 0.0789 -0.0636 0.0199

v524 | -0.0125 0.0442 0.0122 -0.0017 0.0502 -0.0096 0.0077

v337 | 0.0289 0.0983 -0.0524 -0.0098 0.0946 -0.0463 0.0290

v141 | 0.0323 0.0750 -0.0022 -0.0545 0.0915 -0.0501 0.0218

v1408 | 0.0009 -0.0022 0.0072 0.0047 0.0093 -0.0055 0.0127

v614 | -0.0187 -0.0861 -0.0121 -0.0131 -0.0256 0.0042 0.0010

v1172 | 0.0003 -0.0783 0.0839 -0.0814 0.0116 -0.0094 0.0044

v47 | 0.0240 0.0115 0.0147 -0.0050 0.1289 -0.0249 0.0394

v788 | -0.0086 -0.0394 -0.0079 -0.0421 -0.2032 0.0505 -0.1653

v329 | 0.0039 -0.0108 0.0192 0.0292 0.0282 0.0895 0.0015

v961 | 0.0067 0.0042 0.0028 0.0663 -0.1972 0.0855 0.0318

v608 | 0.0071 0.0601 -0.0154 -0.0677 0.0404 -0.0350 0.0050

v820 | 0.0324 -0.0277 -0.0018 0.0758 0.0374 0.0057 0.0661

sma_grey_vol | 0.0008 0.0083 0.0232 0.4027 0.0022 0.0311 0.0172

v1076 | -0.0142 -0.0107 0.0066 -0.0160 -0.0488 -0.0116 -0.0376

v953 | 0.0285 -0.0897 0.0503 -0.0125 -0.0246 0.0325 -0.0053

v1321 | 0.0126 0.0574 -0.0216 -0.0322 0.0352 -0.0097 0.0059

v1133 | -0.0147 0.0279 -0.0770 0.0731 0.0003 0.0112 0.0617

v804 | -0.0049 0.0159 -0.0302 0.0048 -0.0404 -0.0398 -0.0523

v861 | 0.0253 0.0540 -0.0078 -0.0029 0.1827 -0.0682 0.0539

v420 | 0.0039 -0.0156 0.0097 0.0312 0.0364 -0.0014 0.0598

v162 | -0.0413 -0.1141 -0.0040 -0.0123 -0.2855 0.1209 -0.0771

v639 | -0.0066 0.1113 0.0321 0.0474 0.0951 -0.0922 0.0443

v630 | 0.0149 0.0346 -0.0176 -0.0383 0.0088 -0.0111 0.0247

v940 | -0.0093 -0.0503 0.0351 0.0761 -0.0169 0.0569 -0.0147

v1141 | 0.0152 0.0072 -0.0258 0.0278 -0.0251 0.0133 0.0793

v1096 | 0.0171 0.0869 0.0151 -0.0651 0.0958 -0.1103 -0.0086

v1174 | 0.0254 0.0698 -0.0267 0.0196 -0.0012 -0.0126 0.0752

v291 | 0.0221 0.0536 -0.0326 -0.0042 0.0540 -0.0043 0.0163

v1083 | 0.0033 -0.0434 0.0807 0.0437 0.0155 0.0455 0.0052

v554 | 0.0199 -0.0082 -0.0364 -0.0925 -0.0154 -0.0688 -0.0114

v1102 | 0.0232 0.0901 -0.0813 -0.0388 0.0071 -0.0341 0.0367

v758 | 0.0057 0.0000 -0.0239 -0.0138 -0.0205 0.0203 0.0196

v23 | -0.0323 0.0310 0.0053 -0.0008 0.0102 -0.0354 -0.0340

v1294 | -0.0279 -0.0242 -0.0064 0.0137 -0.0565 0.0080 -0.0387

v1014 | -0.0070 -0.0340 -0.0614 -0.0126 -0.0720 0.0441 0.0043

v1288 | 0.0092 -0.0280 -0.0012 0.0337 0.0145 0.0075 -0.0116

v1193 | -0.0177 -0.0585 0.0061 -0.0158 -0.0504 0.0066 -0.0168

v143 | 0.0053 0.0318 -0.0303 -0.0057 -0.0083 0.0094 0.0232

v648 | -0.0296 -0.0428 0.0132 0.0133 -0.0526 0.1585 -0.0170

v57 | -0.0207 0.0145 0.0126 -0.0157 -0.0543 0.0380 -0.0103

v567 | -0.3126 0.0414 -0.0084 0.0547 0.0570 -0.0118 -0.0297

v783 | 0.0542 0.0191 0.0207 -0.0322 0.1141 -0.0003 0.0851

grip_str_h~h | -0.0715 0.0305 -0.0204 0.3834 0.0750 0.0170 -0.0067

v546 | -0.1750 0.0111 -0.0198 -0.0485 -0.0240 -0.0236 -0.0026

**| acc_gr~l v789 v1253 v1091 v102 v458 v404**

-------------+---------------------------------------------------------------

acc_grey_vol | 1.0000

v789 | -0.0168 1.0000

v1253 | 0.0000 0.0033 1.0000

v1091 | 0.0247 0.0266 0.0259 1.0000

v102 | 0.0975 0.0601 -0.0105 -0.0170 1.0000

v458 | -0.0358 -0.0215 -0.0135 0.0236 -0.0149 1.0000

v404 | 0.0368 0.0248 -0.0166 -0.0195 0.1696 -0.0030 1.0000

v464 | -0.0101 -0.0085 0.0050 0.0587 -0.0580 0.0213 -0.1086

v1165 | -0.0214 -0.0358 -0.0025 -0.0310 -0.1574 0.0358 -0.1554

v312 | 0.0365 -0.0180 0.0269 -0.0211 -0.1127 -0.0112 -0.1234

v732 | -0.0645 0.0246 0.0055 -0.0069 0.1199 0.0268 0.1707

v581 | -0.0126 0.0380 -0.0289 0.0203 0.0194 0.0155 0.0347

v201 | 0.0886 -0.0232 0.0100 0.0203 0.0464 -0.0353 0.0439

v319 | 0.0264 -0.0154 0.0120 -0.0018 -0.0507 0.0007 -0.0009

v164 | -0.0136 0.0621 -0.0016 -0.0110 0.2500 -0.0091 0.0844

v1286 | -0.0278 0.0260 0.0180 -0.0117 0.0202 0.0014 -0.0027

v967 | -0.0276 0.0077 -0.0021 0.0003 0.0704 0.0005 0.0324

v740 | -0.0712 0.0278 -0.0082 0.0278 0.0400 0.0244 0.0361

v1137 | -0.0519 0.0902 -0.0361 0.0615 0.1095 0.0365 0.1189

v369 | -0.0830 0.0444 -0.0134 0.0156 0.0540 -0.0014 0.0635

v1207 | 0.0343 -0.0169 0.0110 -0.0072 -0.0477 -0.0507 -0.0404

v373 | -0.0344 -0.0073 -0.0215 0.0438 0.0400 0.0212 0.0004

v745 | 0.0438 -0.0226 -0.0172 0.0021 0.0362 -0.0059 0.0277

v547 | 0.0391 0.0153 0.0005 0.0194 0.0130 -0.0009 0.0070

v171 | 0.0429 -0.0374 -0.0100 0.0047 -0.0317 0.0161 -0.0382

v144 | -0.0122 -0.0369 0.0181 -0.0112 -0.0353 0.0017 -0.0606

v344 | 0.0228 -0.0511 0.0130 0.0052 0.0183 0.0064 -0.0216

v629 | 0.0644 0.0309 -0.0097 0.0198 0.1211 -0.0247 0.0803

v524 | -0.0448 0.0149 -0.0180 0.0267 -0.0559 0.0241 -0.0247

v337 | -0.0223 0.0457 -0.0344 0.0244 0.0726 0.0263 0.0711

v141 | -0.0289 0.0435 -0.0182 -0.0146 0.1004 0.0075 0.0399

v1408 | 0.0052 0.0113 0.0095 0.0068 0.0157 0.0006 -0.0025

v614 | -0.0086 0.0306 0.0042 -0.0036 -0.0469 -0.0124 -0.0472

v1172 | -0.0337 -0.0252 -0.0258 -0.0763 -0.0853 -0.0253 -0.0943

v47 | -0.0760 0.0572 -0.0244 -0.0300 -0.1527 0.0177 -0.0542

v788 | -0.0083 -0.1601 0.0377 0.0289 -0.0958 0.0399 0.0020

v329 | 0.0007 -0.0448 -0.0063 -0.0169 -0.0252 0.0232 -0.0280

v961 | 0.1436 -0.0048 -0.0003 -0.0684 -0.0315 -0.0509 0.0330

v608 | -0.1106 0.0348 -0.0226 -0.0061 0.0392 0.0034 0.0308

v820 | 0.0464 -0.0123 0.0049 0.0051 -0.0011 0.0079 0.0301

sma_grey_vol | 0.1746 -0.0126 0.0209 0.0276 0.0575 -0.0400 0.0235

v1076 | -0.0241 0.0511 0.0130 0.0180 -0.0191 -0.0122 -0.0043

v953 | -0.0304 -0.0168 0.0045 0.0377 -0.1995 0.0391 -0.0813

v1321 | 0.0062 0.0090 0.0003 0.0382 0.0177 0.0094 0.0325

v1133 | 0.0660 0.0791 0.0237 0.0905 0.1570 -0.0036 0.1278

v804 | 0.0165 0.0552 0.0232 0.0290 0.0083 0.0023 0.0343

v861 | -0.0176 0.0042 -0.0372 0.0063 0.0460 0.0097 -0.0141

v420 | 0.0178 0.0357 0.0003 0.0051 -0.0005 0.0043 0.0125

v162 | -0.0027 -0.1337 0.0583 -0.0134 -0.1289 0.0074 -0.0399

v639 | 0.0409 0.0658 0.0041 0.0555 0.0978 0.0133 0.0482

v630 | 0.0060 0.0342 -0.0199 -0.0036 0.0372 -0.0010 0.0470

v940 | 0.0125 -0.0032 0.0110 -0.0021 -0.0493 -0.0369 -0.0621

v1141 | 0.0065 0.0814 -0.0058 0.0719 0.1088 0.0022 0.0998

v1096 | -0.0298 0.0311 -0.0179 0.0072 -0.0073 -0.0260 -0.0055

v1174 | 0.0441 0.0569 -0.0062 0.0313 0.1059 0.0198 0.0952

v291 | 0.0189 0.0258 -0.0026 0.0600 0.0143 0.0257 0.0318

v1083 | -0.0042 0.0239 0.0030 0.1688 -0.0975 0.0241 -0.0594

v554 | -0.0799 0.0279 0.0030 -0.0003 0.0212 0.0032 0.0311

v1102 | -0.0109 0.0504 -0.0328 -0.0559 0.0746 0.0191 0.0746

v758 | 0.0589 0.0013 -0.0240 -0.0022 0.0088 -0.0034 0.0241

v23 | 0.0275 -0.0055 0.0186 0.0112 0.0298 -0.0075 -0.0018

v1294 | 0.0057 -0.0295 0.0115 -0.0006 -0.0211 -0.0088 -0.0247

v1014 | 0.0217 -0.0182 -0.0038 0.0737 -0.0149 0.0258 -0.0018

v1288 | -0.0015 -0.0000 -0.0021 0.0304 -0.0503 -0.0017 -0.0221

v1193 | -0.0218 -0.0213 0.0065 0.0014 -0.0356 0.0105 -0.0295

v143 | -0.0075 0.0255 -0.0191 0.0145 0.0395 0.0132 0.0794

v648 | 0.0268 -0.0191 -0.0026 -0.0388 -0.0534 -0.0122 -0.0595

v57 | 0.0237 -0.0726 0.0075 -0.0090 -0.0231 -0.0067 -0.0216

v567 | 0.0355 0.0151 0.0025 0.0100 0.0428 -0.0397 0.0030

v783 | -0.0251 0.0872 -0.0145 0.0270 -0.0019 -0.0103 0.0229

grip_str_h~h | 0.1836 -0.0102 0.0206 -0.0186 0.1103 -0.0471 0.0248

v546 | 0.0163 0.0016 -0.0065 -0.0158 0.0152 -0.0090 0.0078

**| v464 v1165 v312 v732 v581 v201 v319**

-------------+---------------------------------------------------------------

v464 | 1.0000

v1165 | 0.0284 1.0000

v312 | 0.0140 0.0178 1.0000

v732 | -0.0764 -0.1986 -0.0657 1.0000

v581 | -0.0442 0.0273 -0.0387 -0.0106 1.0000

v201 | -0.0899 0.0099 0.0410 0.0405 0.0172 1.0000

v319 | -0.0098 0.0573 0.0095 -0.0837 -0.0053 0.0371 1.0000

v164 | -0.0807 -0.0728 -0.1022 0.0940 0.0196 -0.0374 -0.1356

v1286 | -0.0172 -0.0030 -0.0269 0.0069 -0.0139 -0.0229 -0.0160

v967 | 0.0295 -0.0338 -0.0866 0.0385 0.0125 -0.0602 -0.0390

v740 | -0.0356 0.0127 -0.0640 0.1501 -0.0136 -0.0374 -0.0109

v1137 | -0.0165 -0.3417 -0.0529 0.1953 0.0329 0.0018 -0.0406

v369 | -0.0115 -0.0248 -0.1238 0.0503 0.0709 0.0125 -0.0174

v1207 | 0.0276 0.0242 0.0453 -0.0169 -0.0299 0.0554 0.0152

v373 | 0.0291 0.0281 -0.0956 -0.0214 0.0156 -0.0375 -0.0213

v745 | -0.0165 -0.0179 0.0443 0.0438 -0.0215 0.0491 0.0204

v547 | 0.0092 0.0155 0.0121 -0.0552 -0.0217 -0.0058 0.0224

v171 | 0.0102 0.0531 0.0225 -0.0773 0.0087 0.0048 0.0288

v144 | 0.0391 0.0262 0.0490 -0.0440 -0.0343 -0.0106 0.0137

v344 | 0.0298 -0.0215 -0.0044 -0.0205 -0.0325 -0.0233 -0.0157

v629 | -0.0243 -0.0273 -0.0737 -0.0141 0.0377 0.0145 0.0095

v524 | 0.0679 0.0763 -0.0455 0.0040 0.0161 0.0195 -0.0445

v337 | -0.0195 -0.0410 -0.1593 0.0584 0.0819 -0.0263 -0.0691

v141 | -0.0172 -0.0732 -0.0795 0.0852 0.0458 -0.0672 -0.0717

v1408 | -0.0013 -0.0022 -0.0125 -0.0063 0.0215 0.0168 0.0027

v614 | 0.0516 0.0447 0.0361 -0.0424 0.0128 -0.0334 -0.0344

v1172 | -0.0186 0.2158 0.0220 -0.0897 0.0161 0.0576 0.0375

v47 | 0.0221 0.1281 -0.0320 -0.0592 0.0804 -0.0092 0.0315

v788 | 0.0045 0.0171 0.1039 0.0478 -0.0671 0.0017 0.0274

v329 | 0.0351 -0.0191 0.0512 -0.0443 0.0176 0.0039 -0.0027

v961 | -0.0687 -0.0328 0.1103 -0.0146 0.0108 0.1947 0.0699

v608 | -0.0215 -0.0225 -0.0380 0.0952 0.0318 -0.0219 -0.0921

v820 | 0.0025 -0.0529 0.0266 -0.0038 0.0001 0.0174 0.0102

sma_grey_vol | 0.0124 -0.0310 0.0396 -0.0507 -0.0128 0.0867 0.0231

v1076 | 0.0455 0.0221 0.0086 -0.0226 -0.0455 0.0035 0.0009

v953 | 0.0301 0.1318 0.0542 -0.0249 0.0077 -0.0724 0.0056

v1321 | -0.0039 -0.0078 -0.0523 0.0077 0.0410 -0.0109 -0.0099

v1133 | 0.0616 -0.2908 -0.0233 0.1055 -0.0148 -0.0666 -0.0471

v804 | -0.0217 -0.0071 -0.0183 0.0352 -0.0672 0.0064 0.0070

v861 | 0.0028 0.0427 -0.1250 -0.0320 0.0766 -0.0133 -0.0270

v420 | 0.0038 0.0098 0.0200 -0.0041 0.0466 0.0134 -0.0038

v162 | 0.0401 -0.0010 0.1400 0.0673 -0.1343 0.0216 0.0499

v639 | 0.0006 -0.0180 -0.0612 0.0065 0.0311 -0.0214 -0.0488

v630 | -0.0393 -0.0033 -0.0229 0.0335 -0.0205 0.0037 -0.0113

v940 | 0.0037 0.0348 0.0810 -0.0250 -0.0578 0.0298 0.0213

v1141 | -0.0051 -0.2125 0.0286 0.1202 -0.0074 -0.0071 -0.0480

v1096 | -0.0602 0.0720 -0.1235 -0.0039 0.0420 0.0118 -0.0045

v1174 | -0.0314 -0.1599 0.0007 0.0973 0.0031 0.0266 -0.0223

v291 | 0.0326 0.0079 -0.0244 0.0109 0.0292 -0.0528 -0.0279

v1083 | 0.1380 0.0298 0.0475 -0.1444 0.0355 -0.0085 0.0226

v554 | -0.0164 -0.0331 -0.1100 0.0886 -0.0270 -0.0432 -0.0454

v1102 | -0.1044 -0.0563 -0.0476 0.1184 0.0138 -0.0996 -0.0156

v758 | 0.0017 -0.0334 -0.0086 -0.0037 0.0317 0.0127 -0.0158

v23 | 0.0434 -0.0221 -0.0306 -0.0059 -0.0254 -0.0354 -0.0222

v1294 | 0.0200 0.0014 0.0310 0.0046 -0.0331 -0.0165 -0.0754

v1014 | 0.0807 0.0106 0.0442 -0.0124 0.0094 -0.0215 -0.0158

v1288 | 0.0356 0.0133 0.0020 -0.0687 0.0029 -0.0169 -0.0001

v1193 | 0.0210 0.0101 0.0058 -0.0044 -0.0216 -0.0154 -0.0042

v143 | -0.0075 -0.1067 -0.0469 0.1180 0.0514 0.0033 -0.0560

v648 | 0.0270 0.0651 0.1048 -0.0633 0.0264 0.0078 0.0602

v57 | 0.0665 0.0085 -0.0042 0.0263 -0.0369 0.0037 0.0148

v567 | 0.0110 -0.0182 0.0022 -0.0283 -0.1491 0.0040 0.0059

v783 | -0.0190 0.0309 -0.0077 -0.0128 0.0678 0.0200 0.0160

grip_str_h~h | 0.0380 -0.1080 0.0486 -0.0567 -0.1126 0.0322 -0.0114

v546 | 0.0160 -0.0375 0.0048 0.0220 -0.0341 0.0309 -0.0331

**| v164 v1286 v967 v740 v1137 v369 v1207**

-------------+---------------------------------------------------------------

v164 | 1.0000

v1286 | 0.0277 1.0000

v967 | 0.0332 0.0143 1.0000

v740 | 0.0621 0.0175 0.0959 1.0000

v1137 | 0.0661 -0.0563 0.0190 0.0557 1.0000

v369 | 0.0389 -0.0142 0.0500 0.0503 0.0451 1.0000

v1207 | -0.0441 0.0242 -0.0488 -0.0560 -0.0316 -0.0281 1.0000

v373 | 0.0339 -0.0279 0.0440 0.0061 0.0077 0.0280 -0.0529

v745 | 0.0161 -0.0352 -0.0050 -0.0048 0.0502 0.0100 -0.0011

v547 | 0.1102 0.0101 -0.0082 -0.0037 -0.0235 -0.0046 0.0164

v171 | 0.0482 -0.0209 -0.0491 -0.0553 -0.0372 -0.0639 0.0088

v144 | -0.0531 -0.0122 -0.0164 -0.0201 -0.0250 -0.0368 0.0055

v344 | 0.0201 0.0105 0.0097 -0.0166 -0.0157 -0.0340 -0.0105

v629 | 0.1060 -0.0106 0.0752 -0.0065 0.0008 0.0495 -0.0218

v524 | -0.0255 0.0069 0.0070 0.0096 -0.0596 -0.0608 -0.0090

v337 | 0.0513 -0.0157 0.0644 0.0379 0.0821 0.0626 -0.0400

v141 | 0.0719 0.0253 0.0433 0.0645 0.0712 0.0026 -0.0327

v1408 | 0.0042 -0.0016 -0.0033 -0.0146 0.0061 -0.0001 -0.0126

v614 | -0.1911 0.0087 -0.0151 -0.0552 -0.0510 -0.0289 -0.0011

v1172 | -0.0205 0.0148 -0.0999 -0.0920 -0.2023 -0.0045 0.0484

v47 | -0.0174 -0.0045 -0.0078 0.0280 -0.0550 0.0215 -0.0018

v788 | -0.0386 0.0213 -0.0395 0.0085 -0.0368 -0.0585 0.0188

v329 | -0.0298 -0.0113 -0.0146 -0.0292 0.0381 -0.0247 0.0098

v961 | 0.0026 0.0049 -0.1375 -0.0929 -0.0327 -0.0549 0.0907

v608 | 0.0325 0.0320 -0.0181 0.0504 0.0103 0.0739 -0.0071

v820 | 0.0057 -0.0111 -0.0138 -0.0343 0.0164 -0.0129 0.0288

sma_grey_vol | -0.0354 -0.0045 -0.0278 -0.0440 -0.0287 -0.0029 0.0411

v1076 | -0.0023 -0.0126 -0.0102 0.0016 -0.0193 -0.0033 -0.0135

v953 | -0.0602 0.0209 -0.0232 0.0615 -0.0499 -0.0524 0.0164

v1321 | 0.0370 0.0363 0.0125 0.0252 0.0267 0.0229 -0.0337

v1133 | 0.0586 -0.0418 0.0210 -0.0173 0.2231 0.0331 -0.0329

v804 | 0.0235 0.0301 0.0314 0.0136 -0.0350 -0.0063 0.0154

v861 | 0.0353 -0.0196 0.0320 0.0109 -0.0071 0.0645 -0.0213

v420 | 0.0099 -0.0047 -0.0072 0.0099 -0.0006 -0.0078 0.0028

v162 | -0.1119 -0.0016 -0.0440 -0.0307 -0.0535 -0.0733 0.0338

v639 | 0.0398 0.0007 0.0356 0.0626 0.0277 0.0493 -0.0455

v630 | 0.0461 -0.0154 0.0080 0.0152 0.0132 0.0194 -0.0208

v940 | -0.0384 0.0270 -0.0601 -0.0815 -0.0451 -0.0873 0.0963

v1141 | 0.0464 0.0015 0.0004 -0.0063 0.1629 0.0236 -0.0010

v1096 | 0.0416 0.0325 0.0470 0.0446 -0.0826 0.0581 -0.0446

v1174 | 0.0356 0.0147 0.0034 0.0102 0.0523 -0.0052 -0.0291

v291 | 0.0379 -0.0051 0.0400 0.0595 0.0451 0.0017 -0.0325

v1083 | -0.0830 -0.0138 -0.0043 -0.0060 -0.0539 -0.0108 0.0061

v554 | 0.0233 0.0197 0.0099 0.0155 0.0405 0.0231 -0.0318

v1102 | 0.0855 0.0109 0.0080 0.0548 0.0445 0.0465 -0.0424

v758 | 0.0016 -0.0045 -0.0108 -0.0152 0.0188 -0.0193 0.0022

v23 | 0.0345 0.0008 0.0318 0.0017 -0.0147 0.0246 -0.0028

v1294 | -0.0648 -0.0786 0.0059 -0.0170 -0.0128 -0.0205 0.0098

v1014 | -0.0159 -0.0469 -0.0321 -0.0045 0.0743 -0.0153 -0.0257

v1288 | -0.0228 -0.0285 -0.0247 -0.0237 -0.0238 -0.0002 -0.0050

v1193 | -0.0152 -0.0076 -0.0208 -0.0268 -0.0182 0.0082 0.0528

v143 | 0.0236 -0.0278 -0.0084 0.0253 0.1099 0.0215 -0.0197

v648 | -0.0274 0.0272 -0.0448 -0.1551 -0.0785 -0.0890 0.0381

v57 | -0.0545 -0.0036 -0.0093 -0.0287 -0.0018 -0.0425 0.0037

v567 | 0.0103 0.0018 0.0352 0.0119 0.0015 0.0089 0.0100

v783 | 0.0297 0.0136 -0.0054 0.0551 0.0195 0.0163 -0.0120

grip_str_h~h | -0.0206 0.0116 0.0811 -0.0570 -0.0261 -0.0643 0.0649

v546 | -0.0144 -0.0071 -0.0014 0.0008 0.0330 -0.0795 -0.0120

**| v373 v745 v547 v171 v144 v344 v629**

-------------+---------------------------------------------------------------

v373 | 1.0000

v745 | 0.0385 1.0000

v547 | 0.0059 0.0303 1.0000

v171 | 0.0054 -0.0032 0.0255 1.0000

v144 | 0.0047 -0.0189 -0.0143 -0.0137 1.0000

v344 | 0.0349 0.0007 -0.0637 0.0259 0.0141 1.0000

v629 | 0.0276 0.0423 0.1578 -0.0017 -0.0396 -0.0339 1.0000

v524 | 0.0886 -0.0209 0.0188 0.0420 -0.0105 -0.0260 -0.0055

v337 | 0.0432 0.0137 -0.0287 -0.0224 -0.0367 -0.0224 0.0260

v141 | 0.0395 0.0022 -0.0238 -0.0395 -0.0332 -0.0166 0.0052

v1408 | 0.0053 -0.0014 -0.0098 -0.0017 -0.0006 0.0060 0.0066

v614 | -0.0125 -0.0934 -0.1064 0.0884 0.0150 0.0628 -0.1459

v1172 | -0.0122 -0.0166 -0.0012 0.0553 0.0206 0.0068 -0.0208

v47 | 0.0372 -0.0039 0.0024 0.0168 -0.0194 -0.0493 -0.0237

v788 | -0.0182 0.0046 0.0298 0.0393 -0.0380 0.0789 -0.0440

v329 | 0.0092 0.1227 0.0128 0.0026 0.0179 -0.0268 -0.0210

v961 | -0.0811 0.0069 0.0232 0.0322 0.0056 -0.0262 0.0276

v608 | 0.0290 -0.0062 -0.0418 -0.0384 -0.0409 -0.0129 -0.0032

v820 | -0.0281 0.0493 -0.0010 -0.0005 -0.0061 -0.0047 -0.0065

sma_grey_vol | -0.0341 -0.0014 0.0021 0.0234 0.0221 -0.0226 0.0444

v1076 | 0.0075 -0.0202 0.0016 0.0093 0.0308 0.0118 -0.0160

v953 | -0.0230 -0.0221 0.0101 0.0111 0.0115 0.0073 -0.0878

v1321 | 0.0074 -0.0095 -0.0004 0.0077 -0.0242 0.0034 0.0249

v1133 | 0.0040 0.0422 0.0326 -0.0383 -0.0203 0.0137 0.0950

v804 | -0.0045 -0.0438 0.0349 0.0086 -0.0296 0.0223 0.0069

v861 | 0.0337 -0.0289 0.0089 -0.0062 0.0247 -0.0474 0.0359

v420 | -0.0173 -0.0143 0.0141 0.0066 0.0042 -0.0314 -0.0061

v162 | -0.0408 0.0364 -0.0008 0.0080 0.0565 0.0724 -0.0835

v639 | 0.0568 0.0153 -0.0175 0.0109 -0.0357 -0.0084 0.0454

v630 | 0.0054 0.0069 -0.0071 0.0086 -0.0206 -0.0064 0.0085

v940 | -0.0116 -0.0116 0.0224 0.0167 0.0148 -0.0198 -0.0347

v1141 | -0.0185 0.0492 0.0028 -0.0488 -0.0131 -0.0061 0.0289

v1096 | 0.0565 0.0033 -0.0119 -0.0066 -0.0390 -0.0108 0.0390

v1174 | -0.0134 0.0428 0.0047 -0.0252 -0.0415 0.0031 0.0400

v291 | 0.0104 0.0165 0.0262 -0.0251 -0.0065 -0.0279 0.0156

v1083 | -0.0089 -0.0057 0.0065 -0.0005 0.0255 -0.0201 -0.0022

v554 | -0.0074 -0.0122 -0.0467 -0.0415 -0.0025 -0.0052 -0.0633

v1102 | 0.0054 0.0070 -0.0004 -0.0293 -0.0275 -0.0120 0.0272

v758 | -0.0217 -0.0025 -0.0080 -0.0060 0.0018 -0.0165 0.0059

v23 | 0.0392 0.0031 0.0127 0.0068 -0.0465 0.0146 0.0252

v1294 | 0.0135 -0.0018 -0.0368 -0.0135 0.0106 0.0200 -0.0234

v1014 | -0.0031 0.0043 0.0081 0.0281 0.0219 0.0035 -0.0168

v1288 | -0.0007 -0.0307 -0.0034 0.0125 0.0229 0.0110 -0.0072

v1193 | 0.0099 -0.0156 -0.0093 0.0016 0.0194 0.0188 -0.0385

v143 | -0.0113 -0.0066 -0.0243 -0.0477 0.0035 -0.0156 -0.0197

v648 | -0.0483 0.0044 0.0116 0.0126 0.0228 0.0063 0.0046

v57 | 0.0121 0.0281 -0.0068 0.0076 0.0232 0.0590 -0.0142

v567 | 0.0108 0.0321 0.0237 -0.0062 0.0109 0.0075 0.0394

v783 | -0.0237 -0.0065 0.0167 -0.0041 -0.0557 -0.0407 -0.0153

grip_str_h~h | 0.0181 0.0435 0.0372 -0.0094 0.0169 -0.0039 0.0649

v546 | 0.0132 0.0062 -0.0279 -0.0350 0.0158 0.0398 -0.0274

**| v524 v337 v141 v1408 v614 v1172 v47**

-------------+---------------------------------------------------------------

v524 | 1.0000

v337 | 0.0305 1.0000

v141 | 0.0152 0.0516 1.0000

v1408 | 0.0097 0.0160 -0.0020 1.0000

v614 | 0.0255 -0.0306 -0.0040 -0.0165 1.0000

v1172 | -0.0029 -0.0814 -0.0456 -0.0024 0.0601 1.0000

v47 | 0.0713 0.0473 0.0204 0.0210 -0.0088 0.0506 1.0000

v788 | -0.0081 -0.0874 -0.0852 -0.0297 -0.0399 0.0161 -0.0984

v329 | 0.0007 0.0143 0.0152 0.0182 -0.0062 -0.0104 0.0170

v961 | -0.0405 -0.0768 -0.0619 0.0146 -0.0293 0.1224 0.0171

v608 | -0.0046 0.0589 0.0541 0.0074 0.0019 0.0406 0.0208

v820 | -0.0164 -0.0001 0.0033 0.0275 -0.0228 -0.0548 0.0141

sma_grey_vol | -0.0213 -0.0052 -0.0130 0.0127 -0.0234 -0.0159 -0.0302

v1076 | 0.0264 -0.0229 -0.0362 0.0040 0.0196 0.0074 -0.0109

v953 | 0.0355 -0.0460 -0.0169 -0.0101 0.0486 -0.0081 0.0432

v1321 | -0.0006 0.0371 -0.0004 -0.0038 -0.0092 -0.0146 0.0179

v1133 | -0.0430 0.0537 0.0183 0.0094 -0.0751 -0.2250 -0.1028

v804 | 0.0119 -0.0174 -0.0396 -0.0183 0.0259 -0.0197 -0.0656

v861 | 0.0150 0.0704 0.0684 0.0280 -0.0021 0.0045 0.0744

v420 | 0.0123 0.0114 -0.0085 0.0065 0.0163 -0.0122 0.0279

v162 | -0.0082 -0.1232 -0.0840 -0.0329 -0.0322 0.0155 -0.1059

v639 | 0.0221 0.0531 0.0490 -0.0101 -0.0294 -0.1355 -0.0044

v630 | 0.0047 0.0188 0.0025 -0.0061 -0.0386 -0.0165 -0.0192

v940 | 0.0061 -0.0199 -0.0277 0.0033 -0.0050 0.0174 0.0237

v1141 | -0.0633 0.0406 0.0103 0.0023 -0.0535 -0.0796 -0.0724

v1096 | 0.0081 0.0562 0.0402 0.0079 -0.0037 0.0638 0.0586

v1174 | -0.0120 0.0234 0.0358 0.0172 -0.0260 -0.1106 -0.0597

v291 | 0.0384 0.0462 0.0186 0.0080 -0.0205 -0.1299 0.0116

v1083 | 0.0698 0.0126 -0.0102 0.0178 -0.0004 -0.0862 0.0003

v554 | -0.0198 0.0140 0.0473 -0.0161 0.0318 0.0022 -0.0519

v1102 | -0.0165 0.0397 0.0487 0.0027 -0.0332 -0.0636 0.0189

v758 | -0.0176 0.0447 0.0238 0.0082 0.0471 -0.0090 -0.0110

v23 | -0.0197 -0.0002 0.0308 -0.0071 -0.0178 -0.0251 -0.0204

v1294 | 0.0153 -0.0147 -0.0194 -0.0033 0.0342 0.0078 -0.0116

v1014 | -0.0141 -0.0001 -0.0179 -0.0073 0.0182 -0.0066 -0.0253

v1288 | 0.0006 -0.0066 -0.0171 0.0034 0.0125 -0.0019 0.0135

v1193 | 0.0050 -0.0105 -0.0180 -0.0081 0.0108 0.0133 -0.0046

v143 | -0.0150 0.0504 0.0937 0.0028 -0.0234 -0.0473 -0.0067

v648 | -0.0004 -0.0547 -0.0652 -0.0041 0.0106 0.1257 0.0172

v57 | 0.0381 0.0234 -0.0173 0.0116 0.0018 0.0540 -0.0007

v567 | 0.0014 0.0035 0.0003 -0.0150 -0.0285 -0.0249 0.0051

v783 | 0.0264 0.0118 0.0098 0.0162 0.0175 0.0332 0.0688

grip_str_h~h | -0.0213 0.0144 -0.0153 0.0022 -0.0389 -0.0981 -0.0333

v546 | 0.0200 -0.0053 0.0478 0.0204 0.0239 -0.0007 -0.0084

**| v788 v329 v961 v608 v820 sma_gr~l v1076**

-------------+---------------------------------------------------------------

v788 | 1.0000

v329 | -0.0239 1.0000

v961 | 0.0305 0.0084 1.0000

v608 | 0.0014 -0.0263 -0.0584 1.0000

v820 | 0.0019 0.0303 0.0482 -0.0290 1.0000

sma_grey_vol | -0.0456 0.0279 0.1344 -0.0443 0.0483 1.0000

v1076 | 0.0275 -0.0258 0.0014 0.0046 -0.0426 -0.0072 1.0000

v953 | 0.0587 0.0264 -0.0895 -0.0512 0.0010 -0.0533 -0.0075

v1321 | 0.0273 -0.0469 -0.0347 0.0221 -0.0008 -0.0056 -0.0131

v1133 | 0.0282 -0.0124 -0.0136 -0.0047 0.1063 0.0467 0.0037

v804 | 0.1298 -0.0634 -0.0096 0.0041 -0.0419 -0.0073 0.0321

v861 | -0.2546 0.0102 -0.0392 0.0108 0.0225 0.0105 -0.0172

v420 | -0.0212 -0.0051 0.0000 -0.0046 0.0326 0.0239 0.0009

v162 | 0.2684 0.0007 0.0955 -0.0418 -0.0029 -0.0105 0.0576

v639 | -0.0593 -0.0206 -0.1082 0.0005 -0.0054 0.0162 -0.0067

v630 | 0.0003 -0.0312 -0.0007 0.0551 -0.0049 -0.0168 0.0008

v940 | 0.0300 0.0183 0.0741 -0.0109 0.0210 0.0310 0.0081

v1141 | 0.0175 -0.0074 0.0475 0.0756 0.0921 0.0151 -0.0071

v1096 | -0.0468 -0.0553 -0.0848 0.0474 -0.0379 -0.0411 -0.0165

v1174 | 0.0058 -0.0279 0.0075 0.0286 0.0579 0.0057 0.0017

v291 | 0.0002 0.0170 -0.0986 -0.0042 0.0194 -0.0126 -0.0025

v1083 | -0.0134 0.0206 -0.0483 -0.0389 0.0431 0.0393 0.0021

v554 | 0.0291 -0.0389 -0.0384 0.0437 -0.0122 -0.0545 0.0207

v1102 | -0.0323 -0.0090 0.0016 0.0537 0.0057 -0.0572 -0.0157

v758 | -0.0676 0.0019 0.0383 0.0286 0.0103 0.0362 -0.0221

v23 | -0.0186 -0.0073 -0.0343 -0.0108 -0.0193 -0.0076 0.0088

v1294 | 0.0573 0.0043 -0.0087 0.0146 -0.0235 0.0024 0.0240

v1014 | 0.0317 0.0101 -0.0107 -0.0490 -0.0112 0.0140 0.0113

v1288 | -0.0203 0.0090 0.0043 -0.0580 0.0186 0.0358 0.0130

v1193 | 0.0194 -0.0050 -0.0072 -0.0333 0.0049 -0.0258 0.0091

v143 | 0.0008 0.0067 -0.0078 0.0258 0.0403 -0.0050 -0.0018

v648 | 0.0527 0.0035 0.1107 -0.0260 0.0003 0.0099 0.0240

v57 | 0.0716 0.0085 0.0292 -0.0418 -0.0090 0.0039 0.0145

v567 | -0.0069 0.0111 -0.0087 -0.0037 -0.0159 0.0488 0.0340

v783 | -0.0058 -0.0009 -0.0054 0.0257 0.0990 -0.0081 -0.0350

grip_str_h~h | -0.0871 0.0343 0.0143 -0.0346 0.0554 0.2066 -0.0260

v546 | -0.0026 0.0126 0.0109 0.0170 -0.0135 -0.0361 -0.0100

**| v953 v1321 v1133 v804 v861 v420 v162**

-------------+---------------------------------------------------------------

v953 | 1.0000

v1321 | -0.0143 1.0000

v1133 | -0.1279 0.0308 1.0000

v804 | 0.0321 0.0296 0.0761 1.0000

v861 | -0.0204 0.0052 -0.0432 -0.0822 1.0000

v420 | 0.0181 0.0152 0.0203 0.0106 -0.0127 1.0000

v162 | 0.0411 -0.0125 0.0091 0.0534 -0.1550 -0.0249 1.0000

v639 | -0.0302 0.0047 0.0978 0.0264 0.0329 0.0026 -0.1488

v630 | -0.0220 0.0215 0.0164 0.0052 0.0044 0.0097 -0.0181

v940 | 0.0169 -0.0296 0.0009 0.0422 -0.0416 0.0017 0.0256

v1141 | -0.0638 0.0079 0.3273 0.0441 -0.0126 0.0323 0.0331

v1096 | -0.0436 0.0173 -0.0394 0.0289 0.0323 -0.0130 -0.1023

v1174 | -0.0379 -0.0026 0.2232 0.0472 -0.0211 0.0087 -0.0096

v291 | 0.0478 0.0083 0.0634 -0.0084 0.0297 0.0038 -0.0269

v1083 | 0.0753 0.0153 0.0471 -0.0114 0.0214 0.0284 -0.0397

v554 | -0.0105 0.0172 0.0055 0.0325 -0.0042 -0.0002 0.0222

v1102 | -0.0170 -0.0034 0.0480 0.0221 0.0059 0.0028 -0.0273

v758 | -0.0689 0.0079 0.0126 -0.0321 0.0462 -0.0177 -0.0386

v23 | -0.0336 -0.0103 0.0025 -0.0060 0.0291 0.0004 -0.0179

v1294 | 0.0057 -0.0640 0.0187 0.0080 -0.0433 -0.0345 0.0463

v1014 | -0.0139 0.1207 0.0366 -0.0890 0.0243 -0.0283 0.0576

v1288 | 0.0077 0.0137 0.0224 -0.0106 0.0368 0.0088 -0.0124

v1193 | 0.0013 -0.0174 -0.0224 -0.0099 0.0035 -0.0128 0.0390

v143 | -0.0207 -0.0084 0.0923 -0.0077 0.0056 0.0063 -0.0044

v648 | 0.0081 -0.0145 -0.0656 0.0205 -0.0508 -0.0054 0.0818

v57 | -0.0225 -0.0059 0.0061 -0.0317 -0.0503 -0.0167 0.0931

v567 | -0.0181 0.0004 0.0384 0.0391 -0.0136 0.0185 -0.0102

v783 | 0.0475 0.0390 -0.0309 -0.0346 0.0225 0.0512 -0.0720

grip_str_h~h | -0.0935 -0.0483 0.1249 0.0463 -0.0100 0.0350 -0.0370

v546 | -0.0398 0.0139 0.0327 -0.0160 -0.0042 0.0145 0.0136

**| v639 v630 v940 v1141 v1096 v1174 v291**

-------------+---------------------------------------------------------------

v639 | 1.0000

v630 | 0.0321 1.0000

v940 | -0.1082 -0.0285 1.0000

v1141 | 0.0231 0.0063 0.0308 1.0000

v1096 | 0.0728 0.0335 -0.0148 -0.0481 1.0000

v1174 | 0.0620 0.0180 -0.0188 0.2481 0.0113 1.0000

v291 | 0.0756 -0.0002 -0.0353 0.0106 -0.0232 0.0157 1.0000

v1083 | 0.0786 -0.0189 -0.0018 0.0002 -0.0384 0.0563 0.0709

v554 | 0.0018 0.0215 -0.0254 0.0127 0.0302 0.0268 0.0058

v1102 | -0.0001 0.0385 -0.0329 0.0699 0.0504 0.0455 -0.0144

v758 | -0.0041 0.0107 -0.0187 0.0000 0.0191 -0.0038 0.0101

v23 | 0.0386 -0.0063 -0.0152 -0.0064 0.0221 -0.0045 0.0101

v1294 | -0.0076 -0.0249 0.0046 0.0026 -0.0037 -0.0103 0.0004

v1014 | -0.0329 -0.0063 -0.0234 -0.0042 -0.0600 -0.0914 0.0574

v1288 | 0.0013 0.0145 -0.0035 0.0106 -0.0175 -0.0143 0.0207

v1193 | -0.0184 -0.0032 0.0033 -0.0199 -0.0237 -0.0110 -0.0135

v143 | 0.0255 0.0138 -0.0047 0.0921 0.0016 0.0755 0.0503

v648 | -0.1430 -0.0135 0.0510 -0.0231 -0.0236 -0.0334 -0.0768

v57 | -0.0228 -0.0134 -0.0044 -0.0027 -0.0151 0.0003 -0.0081

v567 | 0.0348 -0.0086 0.0291 0.0067 0.0100 -0.0083 0.0176

v783 | 0.0075 0.0375 0.0038 -0.0376 0.0304 -0.0056 0.0137

grip_str_h~h | 0.1048 -0.0411 0.1082 0.0509 -0.0423 0.0114 0.0160

v546 | -0.0089 0.0413 -0.0031 0.0194 0.0137 0.0305 0.0124

**| v1083 v554 v1102 v758 v23 v1294 v1014**

-------------+---------------------------------------------------------------

v1083 | 1.0000

v554 | -0.0398 1.0000

v1102 | -0.1685 0.0275 1.0000

v758 | 0.0210 0.0157 -0.0270 1.0000

v23 | -0.0181 -0.0079 -0.0030 -0.0139 1.0000

v1294 | 0.0064 -0.0008 -0.0249 -0.0006 -0.0029 1.0000

v1014 | 0.0439 0.0054 -0.0713 0.0425 0.0123 0.0073 1.0000

v1288 | 0.0411 -0.0135 -0.0332 0.0066 0.0166 -0.0058 0.0377

v1193 | 0.0031 0.0076 -0.0115 -0.0147 0.0025 0.0100 -0.0017

v143 | 0.0054 0.0354 0.0243 0.0101 -0.0162 -0.0045 0.0001

v648 | -0.0083 -0.0261 -0.0333 -0.0175 -0.0182 0.0097 -0.0042

v57 | 0.0043 0.0084 -0.0376 0.0027 0.0196 0.0159 0.0572

v567 | -0.0003 -0.0075 0.0121 -0.0366 0.0334 0.0023 -0.0411

v783 | 0.0617 0.0116 -0.0061 -0.0096 -0.0702 -0.0460 0.0033

grip_str_h~h | 0.0201 -0.0868 -0.0593 -0.0067 0.0228 0.0165 -0.0872

v546 | -0.0216 0.1035 0.0052 0.0027 0.0170 -0.0005 0.0036

**| v1288 v1193 v143 v648 v57 v567 v783**

-------------+---------------------------------------------------------------

v1288 | 1.0000

v1193 | 0.0111 1.0000

v143 | -0.0055 -0.0173 1.0000

v648 | -0.0004 -0.0070 -0.0015 1.0000

v57 | -0.0026 0.0069 0.0215 0.0630 1.0000

v567 | -0.0012 -0.0159 -0.0057 -0.0063 -0.0212 1.0000

v783 | 0.0007 -0.0338 0.0064 -0.0006 -0.0536 -0.0080 1.0000

grip_str_h~h | -0.0178 -0.0304 -0.0146 0.0012 0.0015 0.1256 -0.0445

v546 | -0.0150 0.0001 0.0029 -0.0122 0.0524 0.0706 -0.0252

| grip_s~h v546

-------------+------------------

grip_str_h~h | 1.0000

v546 | -0.0107 1.0000

| **Supplementary Table 4.** Cluster validity methods to determine appropriate cluster number for all variables. | | | | |
| --- | --- | --- | --- | --- |
| **Cluster Number** | **Calinski-Harabasz*** | **Davies-Bouldin**** | **Sillhouette*** | **Fuzzy Entropy**** |
| *2-Dimensions* | | | | |
| K=2 | 3467 | 0.97 | 0.65 | **0.6113** |
| K=3 | 3355 | 0.88 | 0.66 | 1.0484 |
| K=4 | 3337 | 0.89 | 0.60 | 1.2509 |
| K=5 | **3831** | **0.77** | 0.65 | 1.2481 |
| K=6 | 3795 | 0.80 | **0.68** | 1.6973 |
| *3-Dimensions* | | | | |
| K=2 | **2400** | 1.18 | 0.57 | **0.6093** |
| K=3 | 2089 | 1.21 | **0.59** | 1.0472 |
| K=4 | 1986 | 1.26 | 0.41 | 1.2968 |
| K=5 | 1899 | 1.14 | 0.43 | 1.5437 |
| K=6 | 1805 | **1.02** | 0.44 | 1.7216 |
| *4-Dimensions* | | | | |
| K=2 | **1886** | 1.35 | 0.52 | **0.6040** |
| K=3 | 1553 | **1.23** | **0.53** | 1.0448 |
| K=4 | 1445 | 1.48 | 0.34 | 1.2962 |
| K=5 | 1313 | 1.42 | 0.35 | 1.5494 |
| K=6 | 1215 | 1.33 | 0.33 | 1.7589 |
| *5-Dimensions* | | | | |
| K=2 | **1557** | 1.48 | **0.49** | **0.6040** |
| K=3 | 1234 | **1.40** | 0.31 | 1.0290 |
| K=4 | 1111 | 1.69 | 0.29 | 1.2927 |
| K=5 | 989 | 1.55 | 0.29 | 1.5400 |
| K=6 | 916 | 1.51 | 0.27 | 1.7415 |
| *6-Dimensions* | | | | |
| K=2 | **1313** | 1.61 | **0.45** | **0.6030** |
| K=3 | 1015 | **1.54** | 0.43 | 1.0297 |
| K=4 | 894 | 1.89 | 0.25 | 1.2937 |
| K=5 | 791 | 1.83 | 0.23 | 1.5397 |
| K=6 | 724 | 1.69 | 0.24 | 1.7217 |
| *7-Dimensions* | | | | |
| K=2 | **1192** | 1.69 | **0.43** | **0.6034** |
| K=3 | 910 | **1.63** | 0.41 | 1.0292 |
| K=4 | 801 | 1.99 | 0.23 | 1.2949 |
| K=5 | 706 | 1.93 | 0.22 | 1.5425 |
| K=6 | 639 | 1.84 | 0.21 | 1.7243 |
| *Higher values for Calinski-Harabasz and Silhouette values indicate the optimal cluster number. **Lower values for Davies-Bouldin and Fuzzy Entropy values indicate the optimal class number. | | | | |

**Supplementary Table 5.** Average within- and between-cluster distances and discrimination values on a normalised 0-1 scale for psychosocial derived subgroups.

|  | Cluster 1 | Cluster 2 | Cluster 3 | Cluster 4 | Cluster 5 |
| --- | --- | --- | --- | --- | --- |
| *Within-cluster* distances | | | | | |
| Distance | 0.122 | 0.109 | 0.177 | 0.206 | 0.119 |
| *Between-cluster distances (discrimination value)* | | | | | |
| Cluster 1 | 0 | 0.226 (-0.22) | 0.564 (-0.82) | 0.395 (-0.46) | 0.329 (-0.34) |
| Cluster 2 | 0.226 (-0.22) | 0 | 0.681 (-1.08) | 0.616 (-0.92) | 0.366 (-0.42) |
| Cluster 3 | 0.564 (-0.83) | 0.681 (-1.08) | 0 | 0.459 (-0.54) | 0.332 (-0.29) |
| Cluster 4 | 0.395 (-0.46) | 0.616 (-0.92) | 0.459 (-0.54) | 0 | 0.493 (-0.58) |
| Cluster 5 | 0.329 (-0.34) | 0.366 (-0.42) | 0.332 (-0.29) | 0.493 (-0.58) | 0 |
| Smaller within-cluster distances indicate less variation in the data, while higher between-cluster values indicate greater separability between clusters. | | | | | |

| **Supplementary Table 6.** Binary one vs rest calibration metrics of Brier scores, Log Loss and Expected Calibration Error (ECE) across classifiers. | | | | | | | | | | | | |
| --- | --- | --- | --- | --- | --- | --- | --- | --- | --- | --- | --- | --- |
|  | **Support Vector Machine** | | | **Naïve Bayes** | | | **k-Nearest Neighbour** | | | **Random Forest** | | |
|  | **Brier Score** | **Log Loss** | **ECE** | **Brier Score** | **Log Loss** | **ECE** | **Brier Score** | **Log Loss** | **ECE** | **Brier Score** | **Log Loss** | **ECE** |
| Main analysis – CBP Only  *CBP Class 1*  *CBP Class 2*  *CBP Class 3*  *CBP Class 4*  *CBP Class 5* | <0.001  <0.001  <0.001  <0.001  <0.001 | 0.002  0.002  0.002  0.001  0.001 | <0.001  <0.001  <0.001  <0.001  <0.001 | 0.022  0.017  0.012  0.014  0.007 | 0.076  0.060  0.044  0.051  0.029 | 0.002  0.002  0.001  <0.001  0.001 | 0.055  0.047  0.011  0.019  0.020 | 0.351  0.307  0.058  0.134  0.142 | 0.007  0.009  <0.001  0.001  <0.001 | <0.001  <0.001  <0.001  <0.001  <0.001 | 0.001  0.001  0.003  0.002  <0.001 | <0.001  <0.001  <0.001  <0.001  <0.001 |
| Main analysis – CBP vs Pain-free  *Pain-free*  *CBP Class 1*  *CBP Class 2*  *CBP Class 3*  *CBP Class 4*  *CBP Class 5* | 0.079  0.021  0.052  0.004  0.007  0.017 | 0.265  0.076  0.181  0.015  0.022  0.059 | <0.001  <0.001  <0.001  <0.001  <0.001  <0.001 | 0.113  0.021  0.073  0.005  0.008  0.016 | 0.369  0.066  0.244  0.018  0.028  0.053 | 0.010  0.003 0.014  <0.001  <0.001  <0.001 | 0.180  0.035  0.110  0.009  0.012  0.032 | 1.257  0.260  0.765  0.064  0.087  0.230 | 0.019  0.005  0.010  <0.001  <0.001  0.002 | 0.125  0.024  0.080  0.006  0.009  0.023 | 0.656  0.102  0.420  0.025  0.036  0.116 | <0.001  <0.001  <0.001  <0.001  <0.001  <0.001 |
| Physical analysis – CBP Only  *CBP Class 1*  *CBP Class 2*  *CBP Class 3* | 0.004  0.005  0.005 | 0.015  0.016  0.018 | <0.001  <0.001  <0.001 | 0.021  0.026  0.031 | 0.074  0.089  0.101 | 0.001  <0.001  <0.001 | 0.095  0.125  0.098 | 0.868  0.954  0.819 | 0.007  0.006  <0.001 | 0.005  0.005  0.007 | 0.027  0.027  0.034 | <0.001  <0.001  <0.001 |
| Physical analysis – CBP vs Pain-free  *Pain-free*  *CBP Class 1*  *CBP Class 2*  *CBP Class 3* | 0.077  0.019  0.041  0.027 | 0.255  0.068  0.141  0.095 | <0.001  <0.001  <0.001  <0.001 | 0.110  0.025  0.058  0.037 | 0.360  0.083  0.189  0.125 | 0.005  <0.001  0.004  0.002 | 0.181  0.048  0.097  0.068 | 1.432  0.444  0.772  0.696 | 0.019  0.004  0.008  0.008 | 0.121  0.032  0.066  0.040 | 0.661  0.161  0.346  0.203 | <0.001  <0.001  <0.001  <0.001 |
| Brain Function analysis – CBP Only  *CBP Class 1*  *CBP Class 2*  *CBP Class 3* | 0.005  0.005  0.005 | 0.015  0.016  0.015 | <0.001  <0.001  <0.001 | 0.039  0.027  0.027 | 0.135  0.106  0.099 | <0.001  <0.001  <0.001 | 0.129  0.137  0.139 | 0.136  1.158  1.133 | 0.001  <0.001  0.002 | 0.017  0.012  0.012 | 0.092  0.072  0.072 | <0.001  <0.001  <0.001 |
| Brain Function analysis – CBP vs Pain-free  *Pain-free*  *CBP Class 1*  *CBP Class 2*  *CBP Class 3* | 0.078  0.024  0.033  0.034 | 0.259  0.083  0.119  0.118 | <0.001  <0.001  <0.001  <0.001 | 0.105  0.031  0.042  0.043 | 0.341  0.101  0.146  0.142 | <0.001  <0.001  <0.001  <0.001 | 0.179  0.062  0.082  0.079 | 1.466  0.557  0.687  0.874 | 0.018  0.005  0.006  0.008 | 0.131  0.041  0.052  0.055 | 0.773  0.187  0.281  0.362 | <0.001  <0.001  <0.001  <0.001 |
| Brain Structure analysis – CBP Only  *CBP Class 1*  *CBP Class 2* | 0.003  0.003 | 0.011  0.011 | <0.001  <0.001 | 0.040  0.040 | 0.139  0.139 | 0.001  0.001 | 0.162  0.162 | 5.505  5.505 | 0.006  0.006 | 0.017  0.017 | 0.407  0.407 | <0.001  <0.001 |
| Brain Structure analysis – CBP vs Pain-free  *Pain-free*  *CBP Class 1*  *CBP Class 2* | 0.077  0.037  0.047 | 0.257  0.131  0.163 | <0.001  <0.001  <0.001 | 0.120  0.056  0.073 | 0.389  0.184  0.283 | 0.013  0.004  0.009 | 0.181  0.098  0.114 | 1.467  1.053  1.147 | 0.018  0.011  0.007 | 0.132  0.062  0.079 | 0.728  0.321  0.437 | <0.001  <0.001  <0.001 |
| Test analysis – CBP Only  *CBP Class 1*  *CBP Class 2*  *CBP Class 3*  *CBP Class 4*  *CBP Class 5* | 0.014  <0.001  0.019  0.006  <0.001 | 0.067  0.002  0.094  0.029  0.002 | 0.003  <0.001  0.003  0.002  <0.001 | 0.079  0.189  0.017  0.017  0.143 | 0.270  0.533  0.052  0.074  0.429 | 0.010  0.022  <0.001  <0.001  0.008 | 0.037  0.037  0.037  0.060  0.030 | 0.206  0.230  0.197  0.395  0.202 | 0.005  0.002  0.004  0.010  0.006 | 0.010  <0.001  0.016  0.006  <0.001 | 0.049  0.001  0.080  0.032  <0.001 | 0.005  <0.001  <0.001  0.003  <0.001 |

| **Supplementary Table 7.** Cluster validity methods to determine appropriate cluster number for physical variables (body mass index and grip strength) | | | | |
| --- | --- | --- | --- | --- |
| **Cluster Number** | **Calinski-Harabasz*** | **Davies-Bouldin**** | **Sillhouette*** | **Fuzzy Entropy**** |
| *2-Dimensions* | | | | |
| K=2 | 2162 | 1.149 | 0.5032 | **0.6684** |
| K=3 | **3173** | **0.8248** | **0.5814** | 1.0487 |
| K=4 | 2945 | 0.8926 | 0.5357 | 1.3631 |
| K=5 | 2857 | 0.9543 | 0.4955 | 1.5319 |
| K=6 | 2792 | 0.9457 | 0.4807 | 1.7249 |
| *Higher values for Calinski-Harabasz and Silhouette values indicate the optimal cluster number. **Lower values for Davies-Bouldin and Fuzzy Entropy values indicate the optimal class number. | | | | |

**Supplementary Table 8.** Average within- and between-cluster distances and discrimination values on a normalised 0-1 scale for physical derived subgroups (body mass index and grip strength).

|  | **Cluster 1** | **Cluster 2** | **Cluster 3** |
| --- | --- | --- | --- |
| *Within-cluster* distances | | | |
| Distance | 0.122 | 0.088 | 0.098 |
| *Between-cluster distances (discrimination value)* | | | |
| Cluster 1 | 0 | 0.254 (-0.30) | 0.274 (-0.33) |
| Cluster 2 | 0.254 (-0.30) | 0 | 0.228 (-0.27) |
| Cluster 3 | 0.274 (-0.33) | 0.228 (-0.27) | 0 |
| *Smaller within-cluster distances indicate less variation in the data, while higher between-cluster values indicate greater separability between clusters. | | | |

| **Supplementary Table 9.** Cluster validity methods to determine appropriate cluster number for brain function variables. | | | | |
| --- | --- | --- | --- | --- |
| **Cluster Number** | **Calinski-Harabasz*** | **Davies-Bouldin**** | **Sillhouette*** | **Fuzzy Entropy**** |
| *2-Dimensions* | | | | |
| K=2 | 2111 | 1.216 | 0.4637 | **0.6904** |
| K=3 | **2537** | 0.9369 | **0.4974** | 1.0976 |
| K=4 | 2409 | 0.9759 | 0.4704 | 1.3750 |
| K=5 | 2356 | 0.9567 | 0.4544 | 1.5701 |
| K=6 | 2393 | **0.8779** | 0.4607 | 1.7347 |
| *3-Dimensions* | | | | |
| K=2 | 1353 | 1.582 | 0.3652 | **0.6904** |
| K=3 | **1398** | 1.308 | 0.3718 | 1.0981 |
| K=4 | 1365 | 1.191 | **0.3799** | 1.3820 |
| K=5 | 1280 | 1.218 | 0.3617 | 1.6017 |
| K=6 | 1216 | **1.183** | 0.3493 | 1.7891 |
| *Higher values for Calinski-Harabasz and Silhouette values indicate the optimal cluster number. **Lower values for Davies-Bouldin and Fuzzy Entropy values indicate the optimal class number. | | | | |

**Supplementary Table 10.** Average within- and between-cluster distances and discrimination values on a normalised 0-1 scale for brain function derived subgroups.

|  | **Cluster 1** | **Cluster 2** | **Cluster 3** |
| --- | --- | --- | --- |
| *Within-cluster* distances | | | |
| Distance | 0.116 | 0.112 | 0.105 |
| *Between-cluster distances (discrimination values)* | | | |
| Cluster 1 | 0 | 0.251 (-0.27) | 0.247 (-0.27) |
| Cluster 2 | 0.251 (-0.27) | 0 | 0.228 (-0.24) |
| Cluster 3 | 0.245 (-0.27) | 0.228 (-0.24) | 0 |
| *Smaller within-cluster distances indicate less variation in the data, while higher between-cluster values indicate greater separability between clusters. | | | |

| **Supplementary Table 11.** Cluster validity methods to determine appropriate cluster number for brain structure variables. | | | | |
| --- | --- | --- | --- | --- |
| **Cluster Number** | **Calinski-Harabasz*** | **Davies-Bouldin**** | **Sillhouette*** | **Fuzzy Entropy**** |
| *2-Dimensions* | | | | |
| K=2 | **3897** | **0.8898** | **0.5894** | **0.6901** |
| K=3 | 3398 | 0.9821 | 0.5014 | 1.0524 |
| K=4 | 3134 | 0.9328 | 0.4806 | 1.3690 |
| K=5 | 3030 | 0.9335 | 0.4664 | 1.5691 |
| K=6 | 2966 | 0.9035 | 0.4698 | 1.7386 |
| *3-Dimensions* | | | | |
| K=2 | **3250** | **1.01** | **0.5477** | **0.6916** |
| K=3 | 2638 | 1.159 | 0.4368 | 1.0544 |
| K=4 | 2275 | 1.171 | 0.4016 | 1.3779 |
| K=5 | 2082 | 1.183 | 0.3728 | 1.5804 |
| K=6 | 1944 | 1.192 | 0.3758 | 1.7586 |
| *Higher values for Calinski-Harabasz and Silhouette values indicate the optimal cluster number. **Lower values for Davies-Bouldin and Fuzzy Entropy values indicate the optimal class number. | | | | |

**Supplementary Table 12.** Average within- and between-cluster distances and discrimination values on a normalised 0-1 scale for brain structure derived subgroups.

|  | **Cluster 1** | **Cluster 2** |  |
| --- | --- | --- | --- |
| *Within-cluster* distances | | | |
| Distance | 0.086 | 0.076 |  |
| *Between-cluster distances (discrimination values)* | | | |
| Cluster 1 | 0 | 0.182 (-0.20) |  |
| Cluster 2 | 0.182 (-0.20) | 0 |  |
| *Smaller within-cluster distances indicate less variation in the data, while higher between-cluster values indicate greater separability between clusters. | | | |

| **Supplementary Table 13.** Cluster validity methods to determine appropriate cluster number for all variable only using the training data. | | | | |
| --- | --- | --- | --- | --- |
| **Cluster Number** | **Calinski-Harabasz*** | **Davies-Bouldin**** | **Sillhouette*** | **Fuzzy Entropy**** |
| *2-Dimensions* | | | | |
| K=2 | 1714 | 0.98 | 0.65 | **0.61** |
| K=3 | 1633 | 0.91 | **0.68** | 0.82 |
| K=4 | 1743 | 0.84 | 0.60 | 1.30 |
| K=5 | **1958** | **0.75** | 0.64 | 1.49 |
| K=6 | 1916 | 0.79 | 0.64 | 1.53 |
| *3-Dimensions* | | | | |
| K=2 | **1193** | 1.19 | 0.57 | **0.61** |
| K=3 | 1019 | 1.12 | **0.58** | 0.84 |
| K=4 | 943 | 1.26 | 0.44 | 1.30 |
| K=5 | 923 | 1.11 | 0.45 | 1.50 |
| K=6 | 936 | **0.98** | 0.47 | 1.66 |
| *4-Dimensions* | | | | |
| K=2 | **935** | 1.35 | **0.52** | **0.61** |
| K=3 | 756 | 1.28 | 0.51 | 0.84 |
| K=4 | 687 | 1.51 | 0.35 | 1.30 |
| K=5 | 624 | 1.34 | 0.36 | 1.51 |
| K=6 | 624 | **1.25** | 0.35 | 1.73 |
| *5-Dimensions* | | | | |
| K=2 | **741** | 1.51 | **0.46** | **0.61** |
| K=3 | 576 | **1.44** | 0.45 | 0.84 |
| K=4 | 517 | 1.74 | 0.28 | 1.30 |
| K=5 | 474 | 1.59 | 0.27 | 1.55 |
| K=6 | 445 | 1.50 | 0.28 | 1.73 |
| *6-Dimensions* | | | | |
| K=2 | **620** | 1.66 | **0.43** | **0.61** |
| K=3 | 471 | **1.60** | 0.26 | 0.84 |
| K=4 | 411 | 1.94 | 0.24 | 1.29 |
| K=5 | 374 | 1.83 | 0.24 | 1.54 |
| K=6 | 347 | 1.70 | 0.23 | 1.73 |
| *7-Dimensions* | | | | |
| K=2 | **548** | 1.78 | **0.39** | **0.61** |
| K=3 | 411 | **1.70** | 0.38 | 0.84 |
| K=4 | 356 | 2.11 | 0.22 | 1.33 |
| K=5 | 319 | 1.98 | 0.21 | 1.55 |
| K=6 | 293 | 1.87 | 0.20 | 1.73 |
| *Higher values for Calinski-Harabasz and Silhouette values indicate the optimal cluster number. **Lower values for Davies-Bouldin and Fuzzy Entropy values indicate the optimal class number. | | | | |

**Supplementary Table 14.** Average within- and between-cluster distances and discrimination values on a normalised 0-1 scale for psychosocial derived subgroups derived in the training data only.

|  | Cluster 1 | Cluster 2 | Cluster 3 | Cluster 4 | Cluster 5 |
| --- | --- | --- | --- | --- | --- |
| *Within-cluster* distances | | | | | |
| Distance | 0.159 | 0.056 | 0.217 | 0.124 | 0.054 |
| *Between-cluster distances (discrimination values)* | | | | | |
| Cluster 1 | 0 | 0.293 (-0.36) | 0.471 (-0.53) | 0.396 (-0.56) | 0.487 (-0.75) |
| Cluster 2 | 0.293 (-0.36) | 0 | 0.599 (-0.92) | 0.271 (-0.41) | 0.200 (-0.29) |
| Cluster 3 | 0.471 (-0.53) | 0.599 (-0.92) | 0 | 0.423 (-0.54) | 0.699 (-1.16) |
| Cluster 4 | 0.396 (-0.56) | 0.271 (-0.41) | 0.423 (-0.54) | 0 | 0.287 (-0.44) |
| Cluster 5 | 0.487 (-0.75) | 0.200 (-0.29) | 0.699 (-1.16) | 0.287 (-0.44) | 0 |
| Smaller within-cluster distances indicate less variation in the data, while higher between-cluster values indicate greater separability between clusters. | | | | | |

**Supplementary Table 15.** Average within- and between-cluster distances and discrimination values on a normalised 0-1 scale for psychosocial derived subgroups derived in the testing data only.

|  | Cluster 1 | Cluster 2 | Cluster 3 | Cluster 4 | Cluster 5 |
| --- | --- | --- | --- | --- | --- |
| *Within-cluster* distances | | | | | |
| Distance | 0.155 | 0.054 | 0.243 | 0.140 | 0.054 |
| *Between-cluster distances (discrimination values)* | | | | | |
| Cluster 1 | 0 | 0.286 (-0.36) | 0.454 (-0.51) | 0.420 (-0.55) | 0.483 (-0.76) |
| Cluster 2 | 0.286 (-0.36) | 0 | 0.596 (-0.90) | 0.296 (-0.40) | 0.200 (-0.29) |
| Cluster 3 | 0.454 (-0.51) | 0.596 (-0.90) | 0 | 0.440 (-0.50) | 0.716 (-1.14) |
| Cluster 4 | 0.420 (-0.55) | 0.296 (-0.40) | 0.440 (-0.50) | 0 | 0.309 (-0.42) |
| Cluster 5 | 0.483 (-0.76) | 0.200 (-0.29) | 0.716 (-1.14) | 0.309 (-0.42) | 0 |
| Smaller within-cluster distances indicate less variation in the data, while higher between-cluster values indicate greater separability between clusters. | | | | | |

| **Supplementary Table 16.** Classification accuracy and area under the curve for validation and test sets in the secondary analyses. | | | | |
| --- | --- | --- | --- | --- |
|  | **Support Vector Machine** | **Naïve Bayes** | **k-Nearest Neighbour** | **Random Forest** |
| Validation Accuracy | >99% | 63.6% | 92.3% | >99% |
| Validation AUC (range) | 1.00-1.00 | 0.583-0.999 | 0.949-0.989 | 1.00-1.00 |
| Test Accuracy | 97.9% | 62.9% | 89.9% | 98.4% |
| Test AUC (range) | 0.99-1.00 | 0.566-0.997 | 0.940-0.979 | 0.992-1.00 |

| **Supplementary Table 17.** Classification accuracy (and error; as a percentage) of original and prior probability adjusted classification models. | | | | | | | | |
| --- | --- | --- | --- | --- | --- | --- | --- | --- |
|  | **Support Vector Machine (Original)** | **Support Vector Machine (Probability Adjusted)** | **Naïve Bayes (Original)** | **Naïve Bayes (Probability Adjusted)** | **k-Nearest Neighbour (Original)** | **k-Nearest Neighbour (Probability Adjusted)*** | **Random Forest (Original)** | **Random Forest (Probability Adjusted)** |
| Main analysis – CBP Only  *Overall*  *CBP Class 1*  *CBP Class 2*  *CBP Class 3*  *CBP Class 4*  *CBP Class 5* | >99 (<0.1)  18.7 (0)  55.2 (0)  4.4 (<0.1)  7.1 (0)  14.5 (0) | <99 (<0.1)  18.7 (0)  55.2 (0)  4.4 (<0.1)  7.1 (<0.1)  14.5 (0) | 94.7 (5.3)  16.0 (2.6)  55.1 (0.1)  2.5 (1.9)  6.5 (0.7)  14.5 (0) | 94.3 (5.6)  18.2 (0.5)  52.7 (2.6)  3.8 (0.7)  6.9 (0.2)  14.3 (0.2) | 92.3 (7.7)  15.4 (3.3)  53.7 (1.5)  3.8 (0.6)  6.0 (1.2)  13.5 (1.0) | 88.8 (11.2)  -  -  -  -  - | >99 (<0.1)  18.7 (0)  55.2 (0)  4.5 (0)  7.1 (<0.1)  14.5 (0) | >99 (<0.1)  18.7 0)  55.2 (0)  4.3 (<0.1)  7.1 (<0.1)  14.5 (0) |
| Main analysis – CBP vs Pain-free  *Overall*  *Pain-free*  *CBP Class 1*  *CBP Class 2*  *CBP Class 3*  *CBP Class 4*  *CBP Class 5* | 88.7 (11.2)  75.4 (2.8)  2.4 (1.7)  7.2 (4.9)  0.8 (0.2)  1.3 (0.3)  1.8 (1.4) | 91.7 (8.3)  57.6 (20.7)  3.9 (0.2)  10.5 (1.5)  1.0 (0.0)  1.5 (0.0)  3.0 (0.1) | 83.5 (16.5)  71.6 (6.6)  1.3 (2.8)  7.9 (4.1)  0.4 (0.6)  0.7 (0.8)  1.6 (1.6) | 85.0 (15.0)  37.0 (41.3)  3.8 (0.3)  10.9 (1.1)  0.8 (0.1)  1.5 (0.1)  3.1 (0.1) | 81.1 (18.9)  71.0 (7.2)  1.9 (2.2)  5.5 (6.5)  0.5 (0.5)  0.9 (0.7)  1.3 (1.8) | 54.6 (45.4)  -  -  -  -  -  - | 85.7 (14.3)  71.3 (6.9)  2.9 (1.1)  7.1 (4.9)  0.8 (0.2)  1.2 (0.3)  2.0 (1.2) | 70.8 (20.2)  67.6 (10.5)  2.7 (1.3)  4.9 (4.1)  0.7 (0.3)  1.1 (0.4)  2.0 (1.2) |
| Physical analysis – CBP Only  *Overall*  *CBP Class 1*  *CBP Class 2*  *CBP Class 3* | 98.9 (1.1)  20.6 (0.4)  46.3 (0.2)  32.0 (0.5) | 98.7 (1.3)  20.9 (0.1)  45.6 (0.9)  32.0 (0.6) | 94.5 (5.5)  18.9 (2.0)  45.0 (1.5)  30.6 (2.0) | 94.6 (5.4)  19.9 (1.1)  43.9 (2.6)  30.8 (1.8) | 84.8 (15.6)  15.5 (5.4)  41.1 (5.4)  27.8 (4.8) | 82.9 (17.1)  -  -  - | 98.7 (1.3)  20.4 (0.6)  46.2 (0.3)  32.2 (0.4) | 98.6 (1.4)  20.5 (0.5)  46.2 (0.3)  32.2 (0.4) |
| Physical analysis – CBP vs Pain-free  *Overall*  *Pain-free*  *CBP Class 1*  *CBP Class 2*  *CBP Class 3* | 84.9 (15.1)  71.1 (7.1)  2.8 (1.8)  6.2 (3.9)  4.8 (2.3) | 86.9 (13.1)  58.5 (19.8)  4.3 (0.3)  9.0 (1.1)  6.4 (0.7) | 84.4 (15.6)  73.1 (5.1)  2.7 (2.3)  5.4 (4.7)  3.6 (3.5) | 79.1 (20.9)  39.9 (38.3)  4.1 (0.5)  8.7 (1.5)  6.4 (0.7) | 80.2 (19.8)  71.0 (7.2)  1.7 (2.9)  4.6 (5.6)  3.0 (4.1) | 54.2 (45.8)  -  -  -  - | 84.9 (15.1)  71.1 (7.1)  2.8 (1.8)  6.2 (3.9)  4.8 (2.3) | 71.0 (29.0)  67.3 (10.9)  2.8 (1.7)  6.8 (3.3)  4.8 (2.2) |
| Brain Function analysis – CBP Only  *Overall*  *CBP Class 1*  *CBP Class 2*  *CBP Class 3* | 99.2 (0.8)  31.8 (0.6)  32.1 (0.1)  35.3 (0.1) | 98.9 (1.1)  31.9 (0.6)  31.9 (0.2)  35.2 (0.3) | 95.2 (4.8)  30.5 (1.9)  30.8 (1.3)  33.8 (1.6) | 94.7 (5.3)  30.4 (2.0)  30.8 (1.4)  33.6 (1.9) | 88.9 (11.1)  29.8 (3.6)  28.2 (3.9)  31.9 (3.6) | 79.7 (20.3)  -  -  - | 98.0 (2.0)  31.5 (0.9)  31.6 (0.5)  34.8 (0.6) | 97.8 (2.2)  31.6 (0.8)  31.5 (0.6)  34.7 (0.8) |
| Brain Function analysis – CBP vs Pain-free  *Overall*  *Pain-free*  *CBP Class 1*  *CBP Class 2*  *CBP Class 3* | 88.8 (11.2)  75.3 (2.9)  5.1 (1.9)  3.7 (3.3)  4.7 (3.1) | 86.9 (13.1)  58.5 (19.7)  4.3 (0.3)  9.0 (1.1)  6.4 (0.7) | 84.6 (15.4)  75.0 (3.3)  4.1 (3.0)  2.3 (4.7)  3.4 (4.4) | 79.3 (20.7)  41.5 (36.8)  4.1 (0.5)  8.6 (1.5)  6.4 (0.7) | 79.7 (20.3)  71.0 (7.3)  3.5 (3.6)  2.4 (4.6)  2.9 (4.8) | 53.5 (46.5)  -  -  -  - | 84.5 (15.5)  70.9 (7.3)  4.9 (2.1)  4.0 (3.0)  4.6 (3.1) | 71.6 (28.4)  67.5 (10.7)  2.9 (1.6)  6.8 (3.3)  4.8 (2.3) |
| Brain Structure analysis – CBP Only  *Overall*  *CBP Class 1*  *CBP Class 2* | 99.5 (0.5)  45.7 (0.4)  53.8 (0.1) | 99.7 (0.3)  46.9 (0.2)  53.8 (0.1) | 94.3 (5.7)  43.6 (2.6)  50.8 (3.1) | 94.5 (5.5)  44.0 (2.1)  50.4 (3.5) | 83.6 (16.4)  37.5 (8.7)  46.2 (7.7) | 84.1 (15.9)  -  - | 98.3 (1.7)  45.3 (0.8)  53.0 (0.9) | 98.8 (1.2)  45.4 (0.6)  53.3 (0.6) |
| Brain Structure analysis – CBP vs Pain-free  *Overall*  *Pain-free*  *CBP Class 1*  *CBP Class 2* | 84.2 (15.8)  70.5 (7.8)  6.5 (3.6)  7.4 (4.4) | 85.9 (14.1)  61.7 (16.5)  9.1 (1.0)  10.4 (1.3) | 82.7 (17.3)  69.9 (8.3)  5.1 (4.9)  7.7 (4.1) | 76.6 (23.4)  44.5 (33.7)  8.7 (1.3)  10.1 (1.6) | 80.2 (19.8)  71.0 (7.2)  4.1 (6.0)  5.2 (6.6) | 58.6 (41.4)  -  -  - | 84.2 (15.8)  70.5 (7.8)  6.5 (3.6)  7.4 (4.4) | 73.1 (26.9)  67.3 (11.0)  6.8 (3.2)  7.7 (4.0) |
| Validation analysis – CBP Only  *Overall*  *CBP Class 1*  *CBP Class 2*  *CBP Class 3*  *CBP Class 4*  *CBP Class 5* | >99 (<0.1)  18.2 (0)  26.1 (0)  6.5 (0)  15.2 (<0.1)  33.9 (0) | 100 (0)  18.2 (0)  26.1 (0)  6.5 (0)  15.3 (0)  33.9 (0) | 63.6 (36.4)  13.9 (4.3)  3.7 (22.4)  5.5 (1.0)  15.0 (0.2)  25.5 (8.5) | 60.8 (39.2)  16.5 (1.8)  4.5 (21.6)  6.3 (0.2)  14.7 (0.6)  19.0 (15.0) | 92.3 (7.7)  17.2 (1.0)  25.1 (1.0)  5.1 (1.4)  11.7 (3.5)  33.1 (0.8) | 88.8 (11.2)  -  -  -  -  - | >99 (<0.1)  18.1 (<0.1)  26.1 (0)  6.4 (<0.1)  15.3 (0)  33.9 (0) | >99 (<0.1)  18.2 (0)  26.1 (0)  6.3 (0.2)  15.0 (0.2)  33.9 (0) |
| Test analysis – CBP Only  *Overall*  *CBP Class 1*  *CBP Class 2*  *CBP Class 3*  *CBP Class 4*  *CBP Class 5* | 97.9 (2.1)  16.9 (0)  25.5 (0)  6.0 (1.4)  14.1 (0.7)  35.5 (0) | 97.9 (2.1)  16.9 (0)  25.5 (0)  6.0 (1.4)  14.1 (0.7)  35.5 (0) | 62.9 (37.1)  12.3 (4.6)  5.0 (20.5)  5.8 (1.8)  13.6 (1.2)  26.3 (9.2) | 57.9 (42.1)  15.1 (1.8)  5.0 (20.5)  6.8 (0.6)  13.2 (1.6)  17.9 (17.6) | 89.9 (10.1)  15.5 (1.3)  23.9 (1.6)  5.1 (2.3)  10.8 (4.0)  34.6 (0.9) | 89.9 (10.1)  -  -  -  -  - | 98.4 (1.6)  16.9 (0)  25.5 (0)  6.4 (1.0)  14.2 (0.6)  35.5 (0) | 98.4 (1.6)  16.9 (0)  25.5 (0)  6.4 (1.0)  13.8 (1.1)  35.5 (0) |
| *No class results available | | | | | | | | |


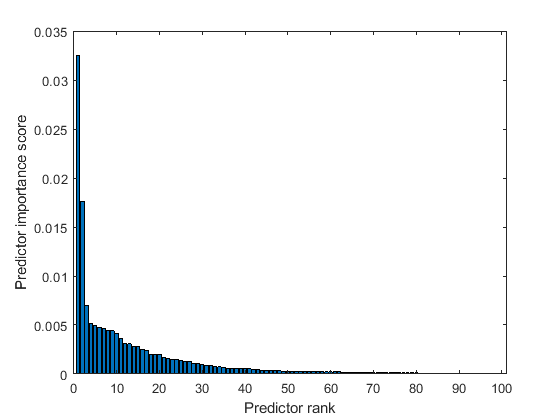


**Supplementary Figure 1.** Minimum redundancy maximum relevance (MRMR) of 100 variables between chronic back pain and pain-free individuals. This ranks the features based on their MRMR and sorts them in order from largest to smallest based on their relevancy value. This is used to assess the difference between two groups. Here we see that that top six features are depressive symptoms, body mass index, inferior parietal love to superior temporal gyrus connectivity (node 222), inferior parietal lobe to middle temporal gyrus connectivity (node 1014), calcarine cortex to precuneus connectivity (node 44).


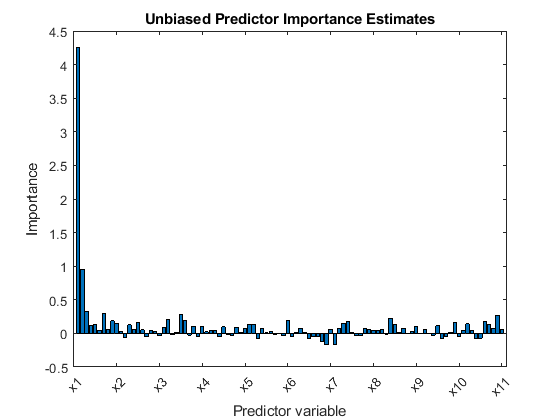


**Supplementary Figure 2.** Regression tree predictor of 100 variables between chronic back pain and pain‑free individuals. Higher values indicate important features and represent which features explain differences between two groups. The six most important variables are depressive symptoms (v1), body mass index (v2), loneliness and social isolation symptoms (v3), grip strength (v99), primary motor cortex grey matter volume (v7) and fronto-orbital grey matter volume (v25).


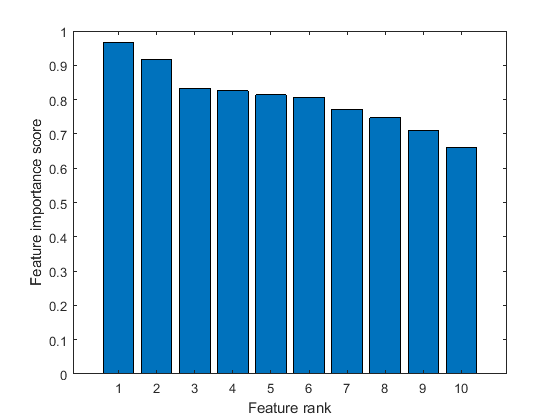


**Supplementary Figure 3.** Laplacian scores to rank features in the order of importance based on how they explain differences in datapoints in a single cohort (e.g. CBP only). For this data, the order of importance of features in a CBP-only space was symptoms of loneliness/social isolation, depressive symptoms, inferior parietal lobe to middle temporal gyrus connectivity (node 1014), grip strength, body mass index, inferior parietal love to superior temporal gyrus connectivity (node 222), calcarine cortex to precuneus connectivity (node 44), fronto‑orbital cortex, primary motor and somatosensory grey matter volumes.


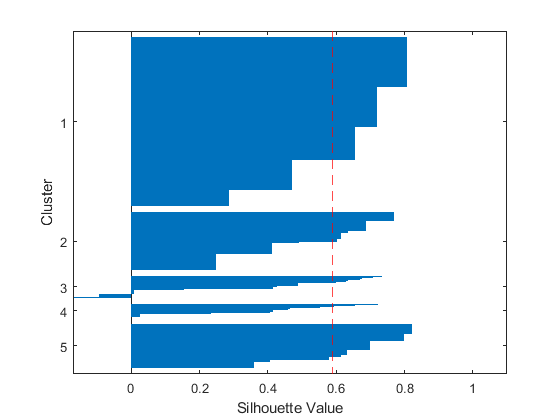


**Supplementary Figure 4.** Post clustering evaluation for clustering consisting of psychosocial variables. The red line indicates the average of all the similarity measures. Values of 1 indicate good similarity of a datapoint to its cluster. The average Silhouette value is 0.589.


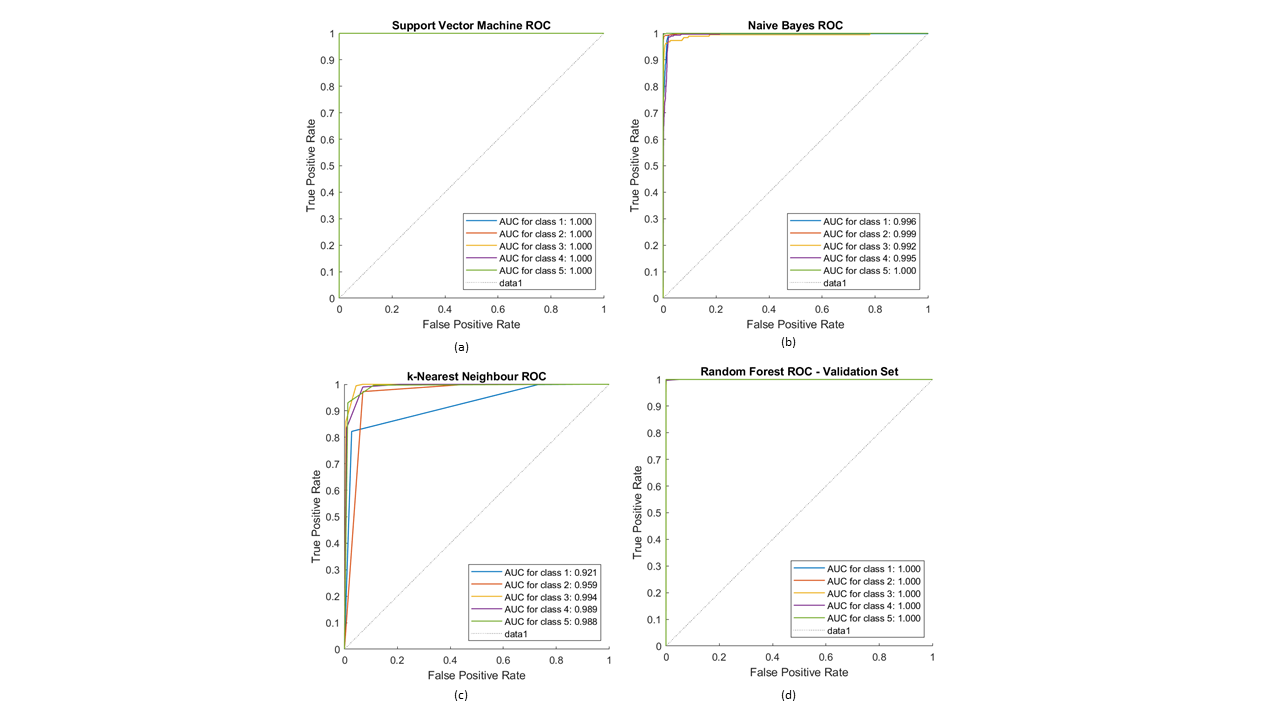


**Supplementary Figure 5.** Class specific area under the curve metrics data for classifiers in the main analysis on CBP individuals only.


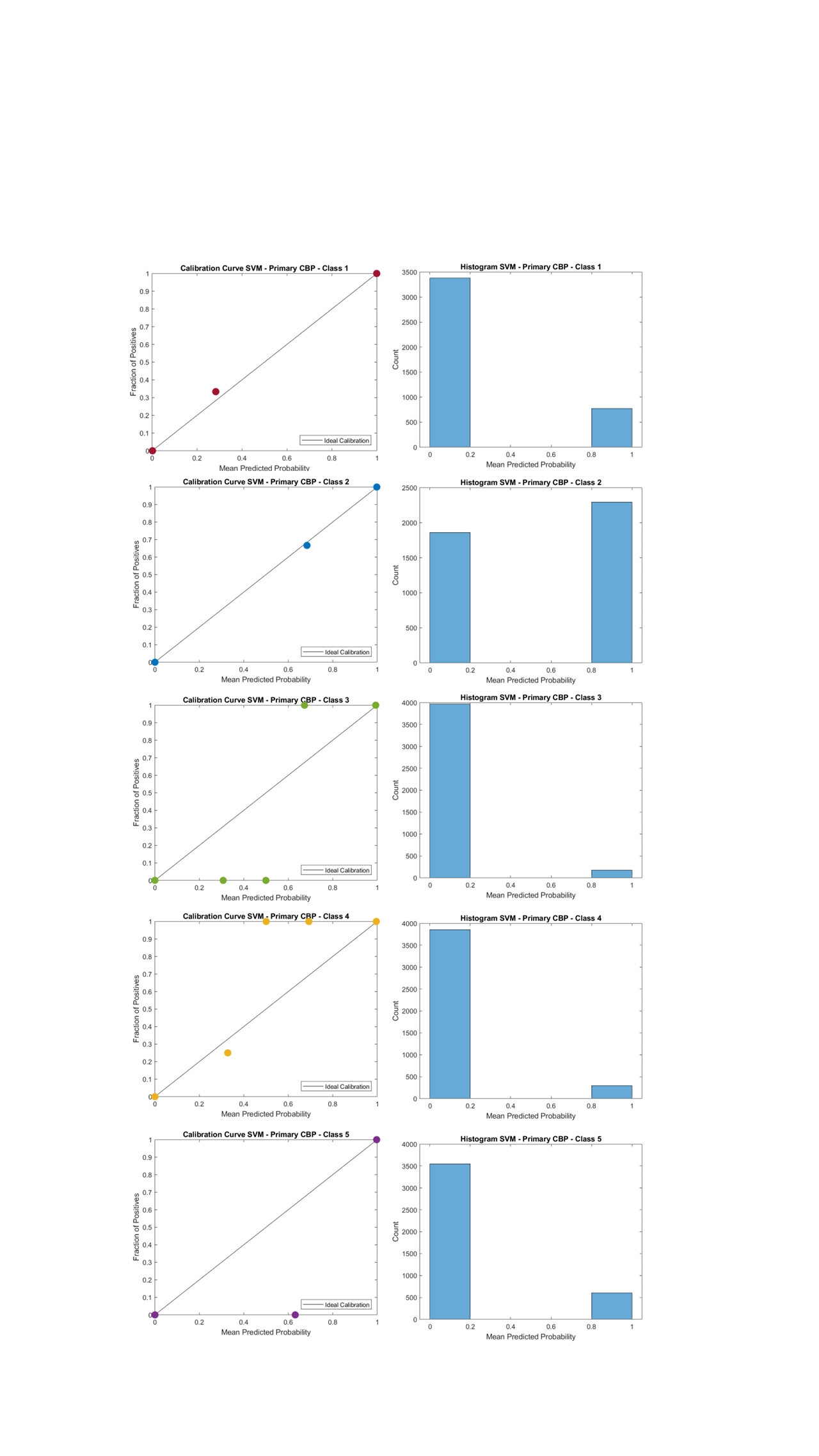


**Supplementary Figure 6.** Support vector machine calibration curves for each class for classifiers in the main analysis on CBP individuals only.


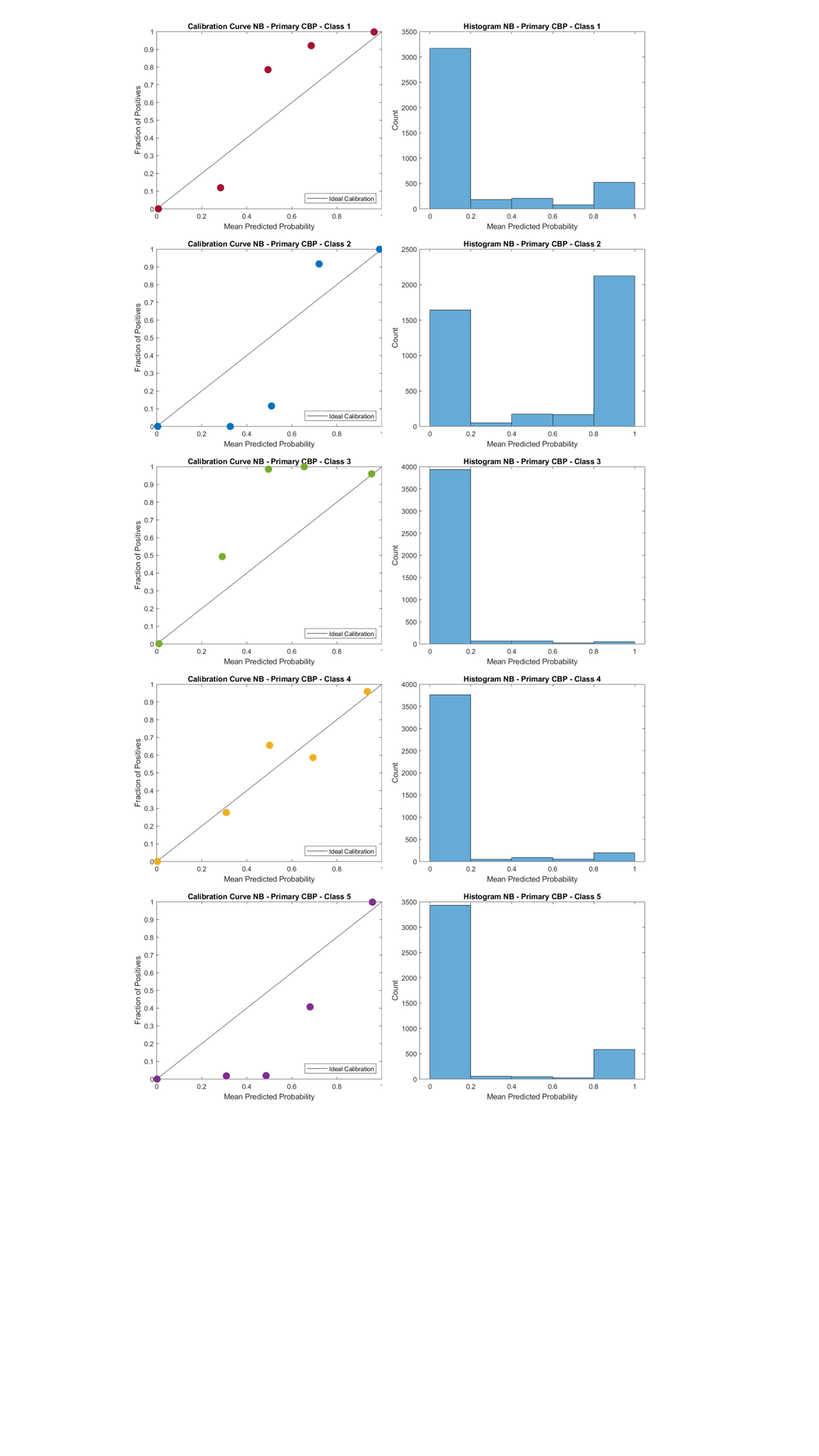


**Supplementary Figure 7.** Naïve Bayes calibration curves for each class for classifiers in the main analysis on CBP individuals only.


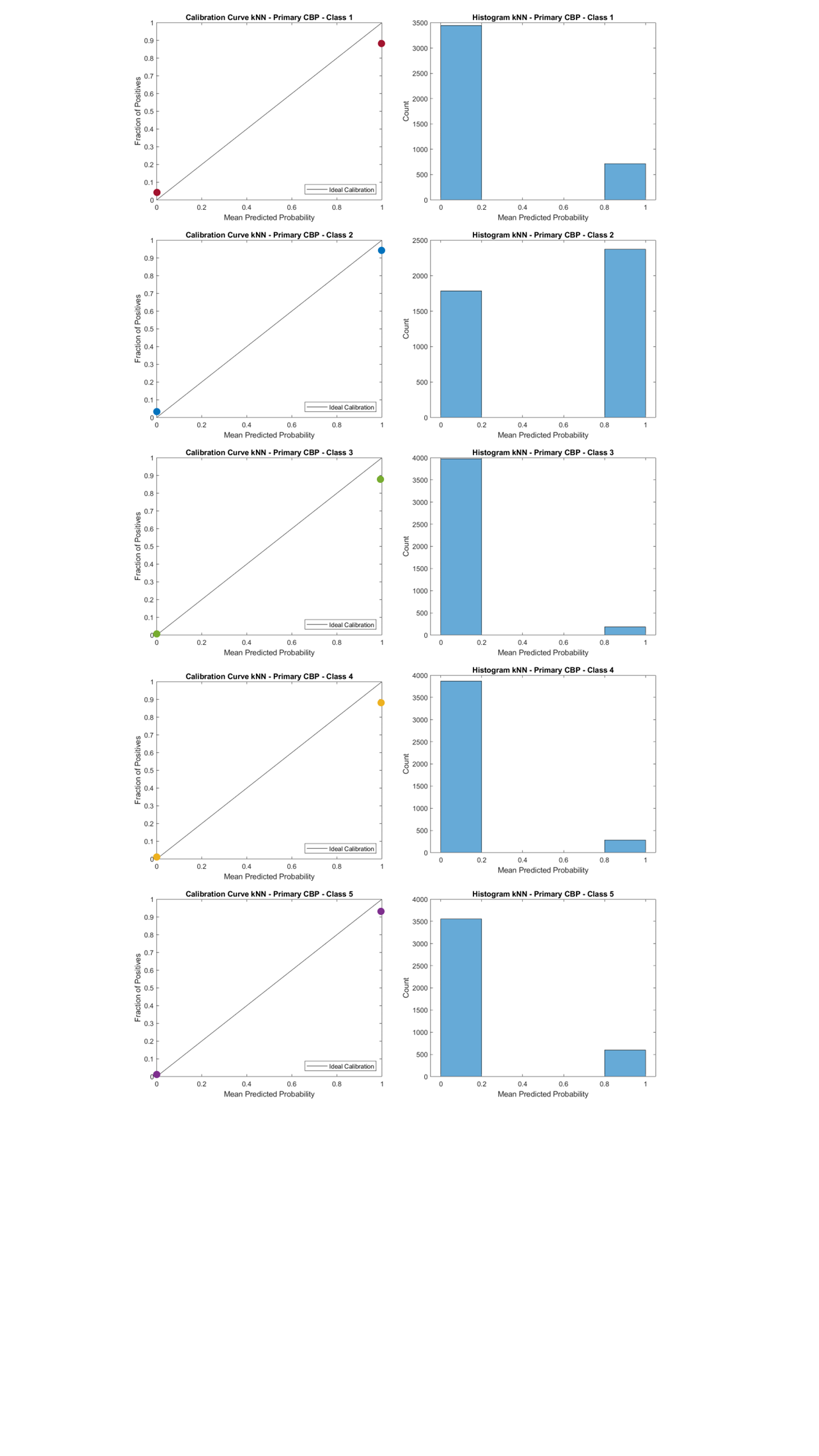


**Supplementary Figure 8.** K-Nearest Neighbour calibration curves for each class for classifiers in the main analysis on CBP individuals only.


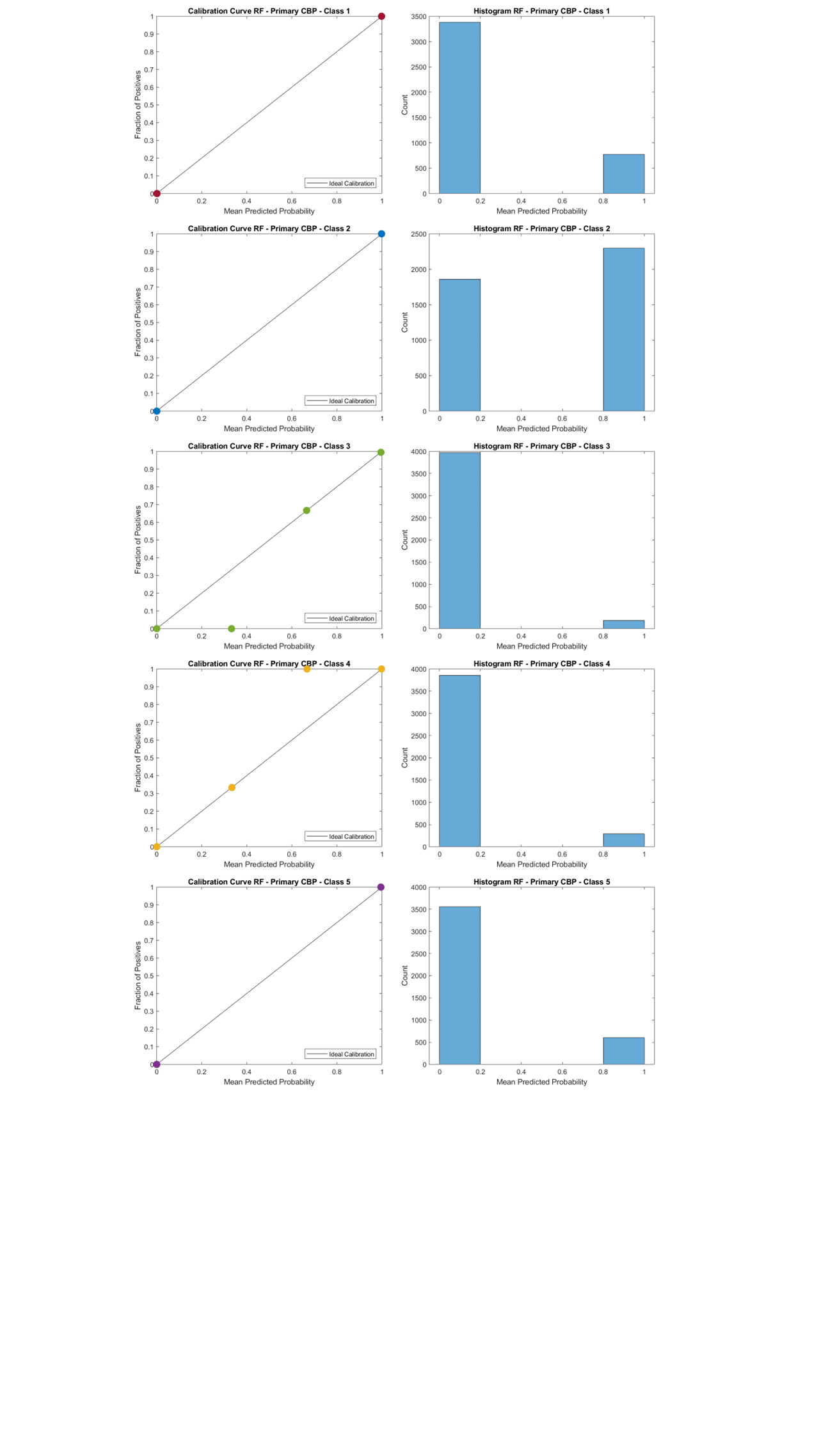


**Supplementary Figure 9.** Random Forest calibration curves for each class for classifiers in the main analysis on CBP individuals only.


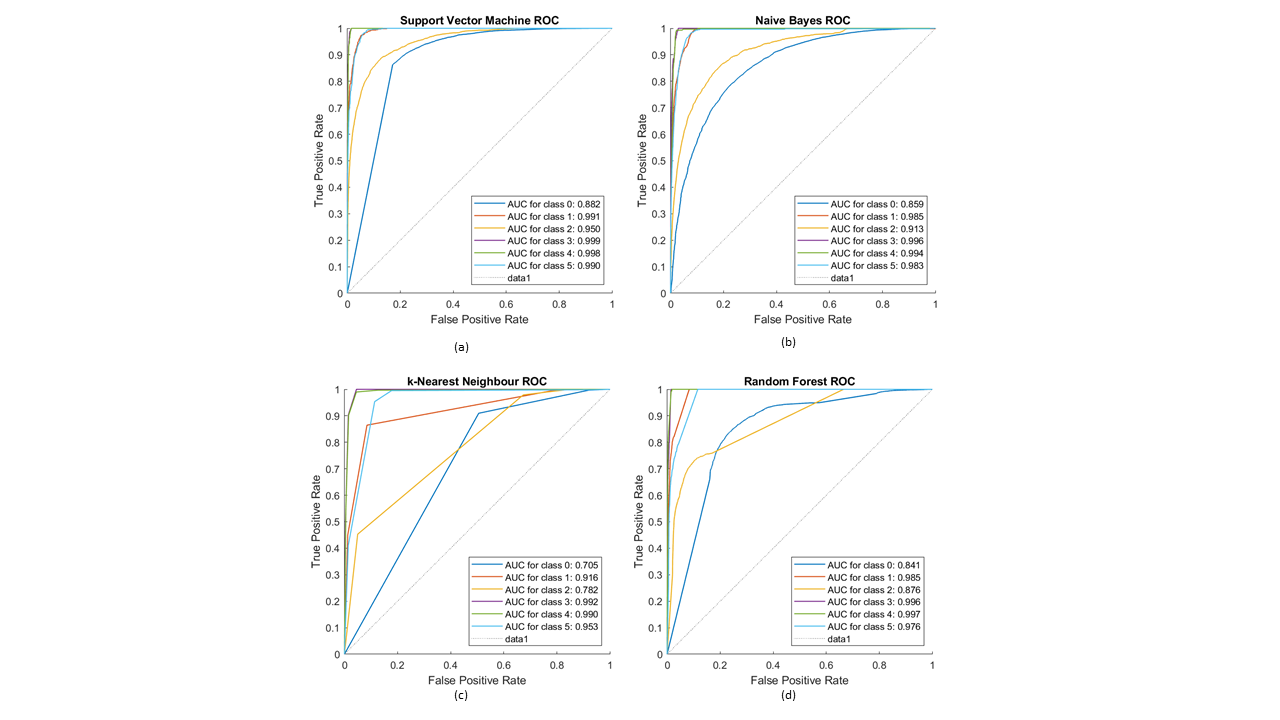


**Supplementary Figure 10.** Class specific area under the curve metrics data for classifiers in the main analysis on CBP and pain-free individuals.


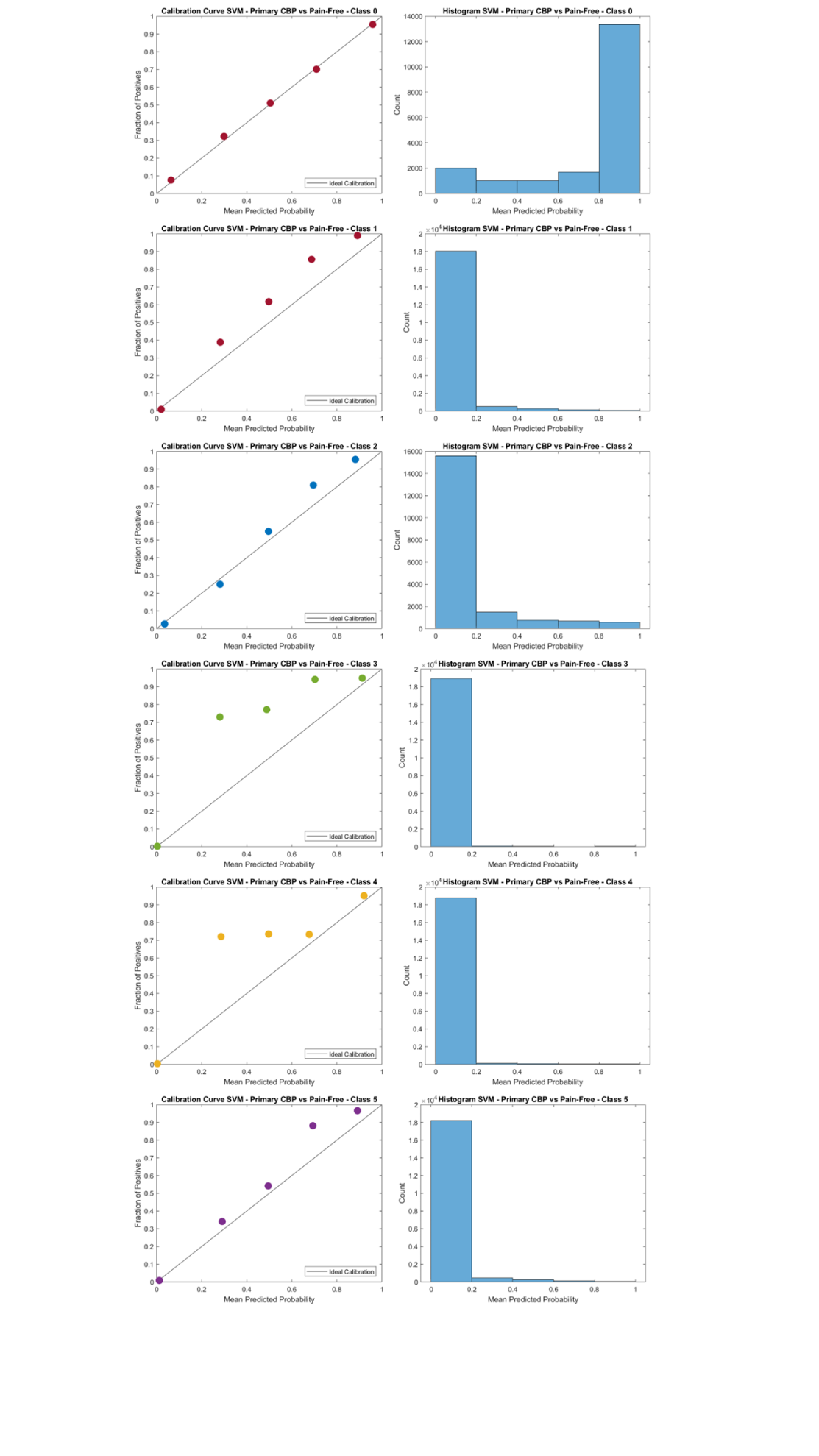


**Supplementary Figure 11.** Support vector machine calibration curves for each class for classifiers in the main analysis on CBP individuals and pain-free individuals.


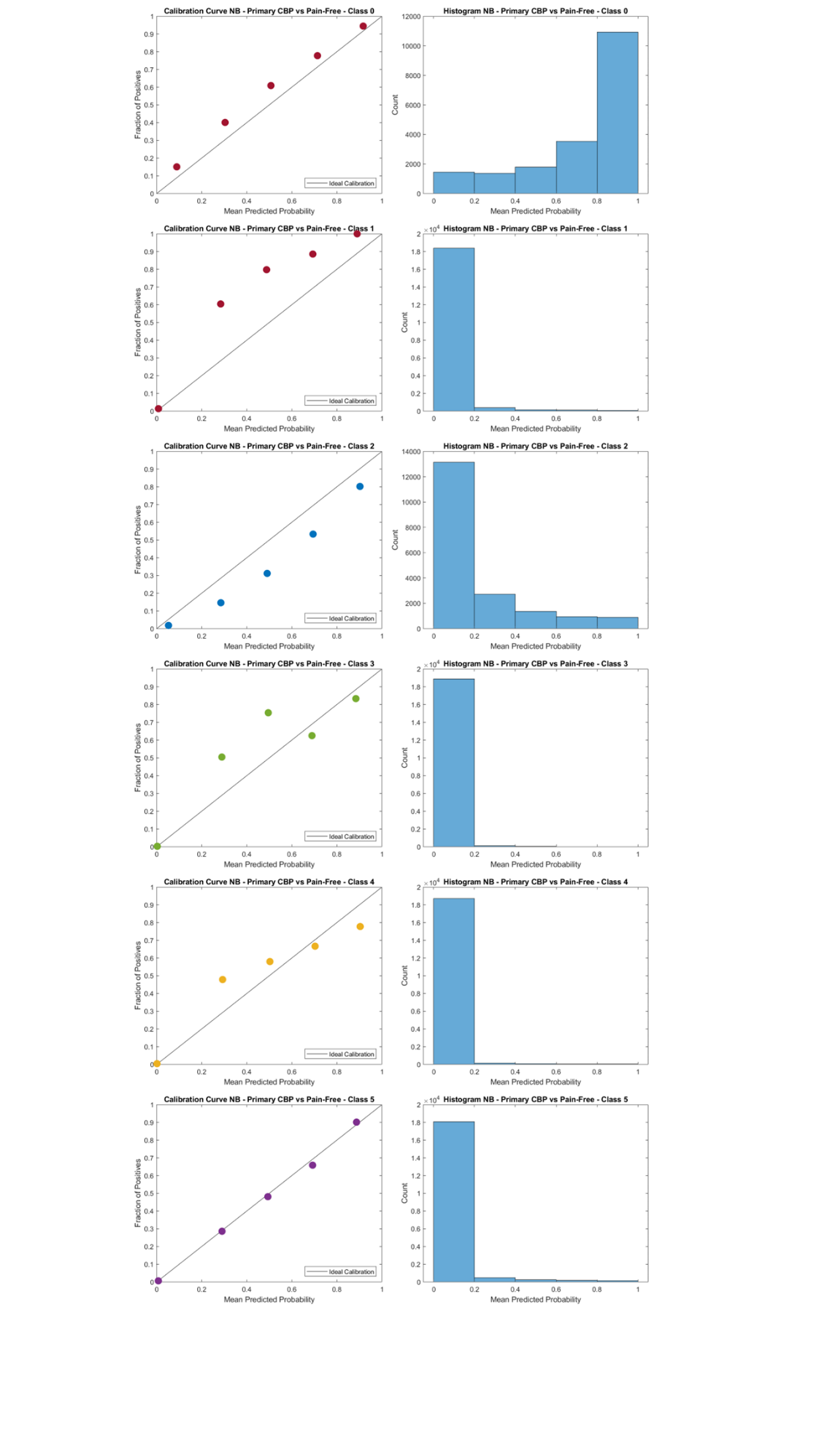


**Supplementary Figure 12.** Naïve Bayes calibration curves for each class for classifiers in the main analysis on CBP individuals and pain-free individuals.


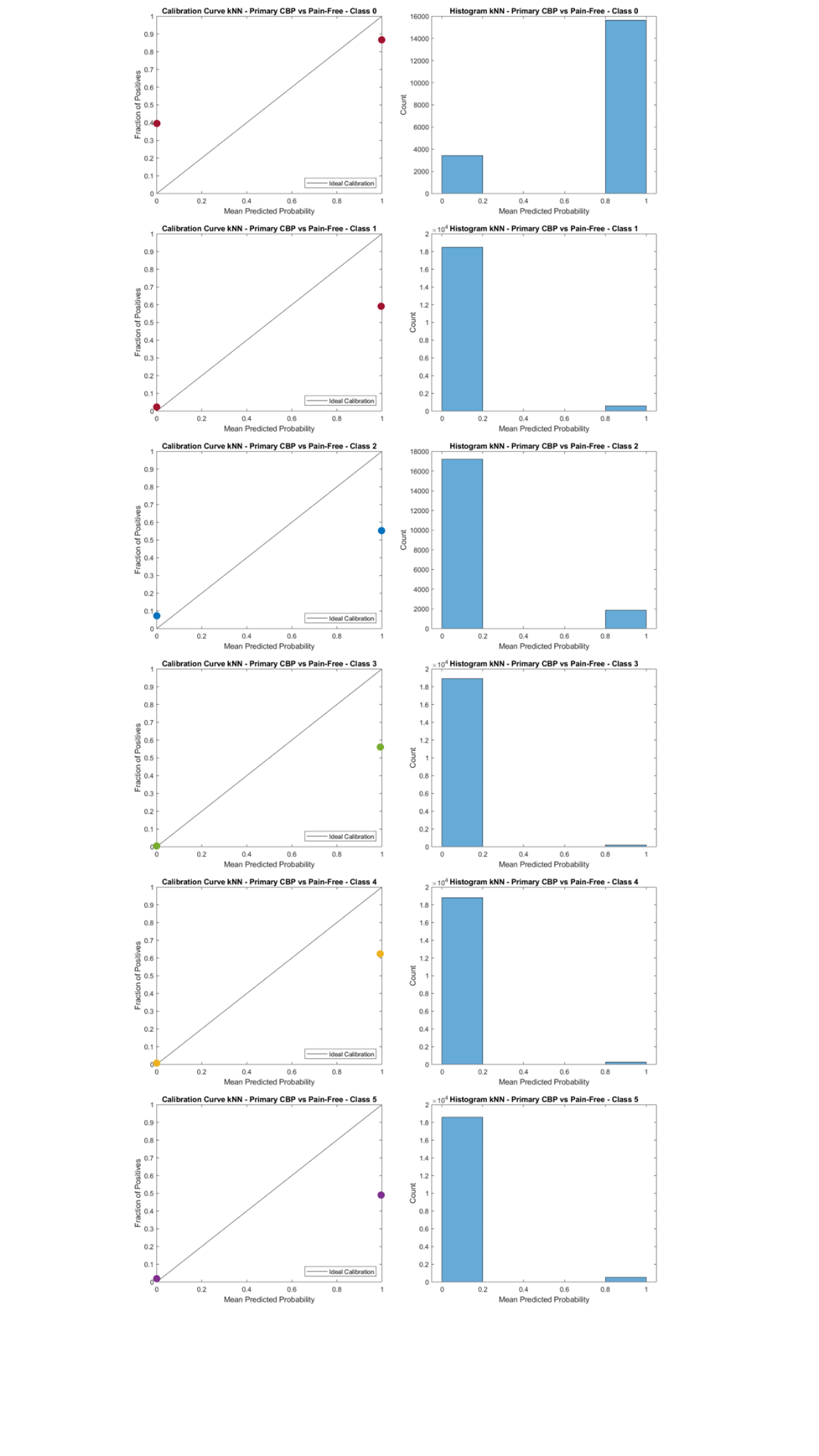


**Supplementary Figure 13.** K-Nearest Neighbour calibration curves for each class for classifiers in the main analysis on CBP individuals and pain-free individuals.


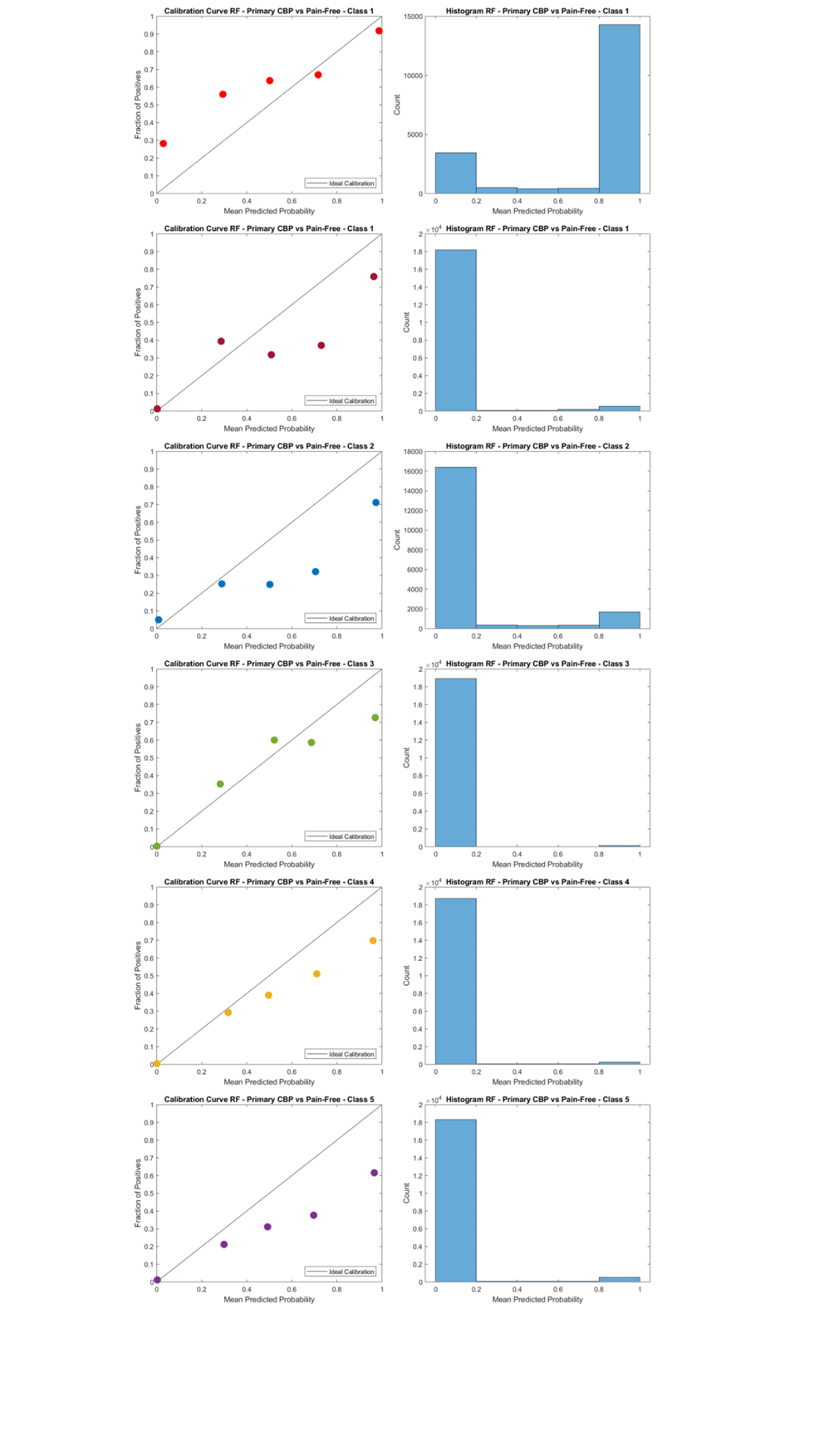


**Supplementary Figure 14.** Random Forest calibration curves for each class for classifiers in the main analysis on CBP individuals and pain-free individuals.


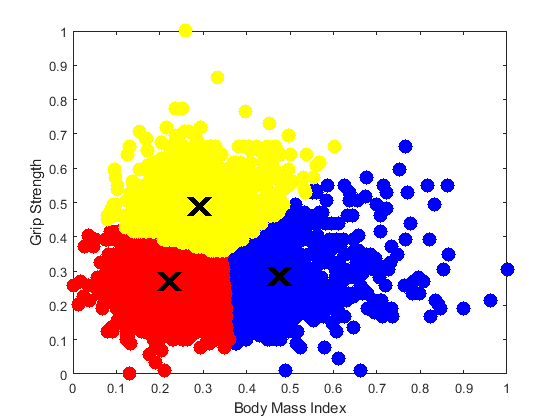


**Supplementary Figure 15.** Scatter plot of the distribution of sub-groups of individuals with CBP based on body mass index and grip strength. Data is presented on (a) normal item range and (b) normalised scale of 0-1. Based on the centroids of fuzzy c-means clustering, classes and colours are (1; blue) higher body mass index and low grip strength (n=870; 20.9%); (2; red) lower body mass index and grip strength (n=1,933; 46.5%); and (3; yellow) lower body mass index and higher grip strength (n=1,353; 32.6%) (Supplementary Figure 5). Black squares on the heat map indicate no class was available at those values. The X value on the scatter plot indicates the centroid of that cluster.


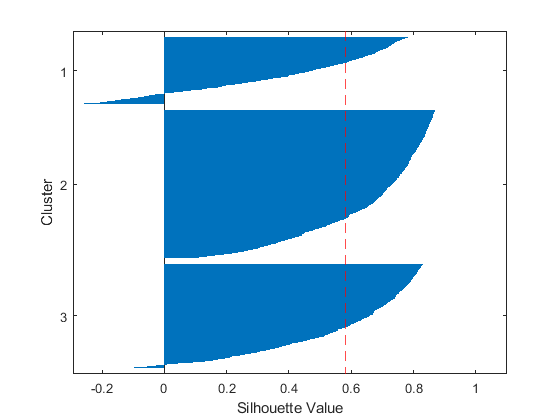


**Supplementary Figure 16.** Post clustering evaluation for clustering consisting of physical variables. The red line indicates the average of all the similarity measures. Values of 1 indicate good similarity of a datapoint to its cluster. The average Silhouette value is 0.581.


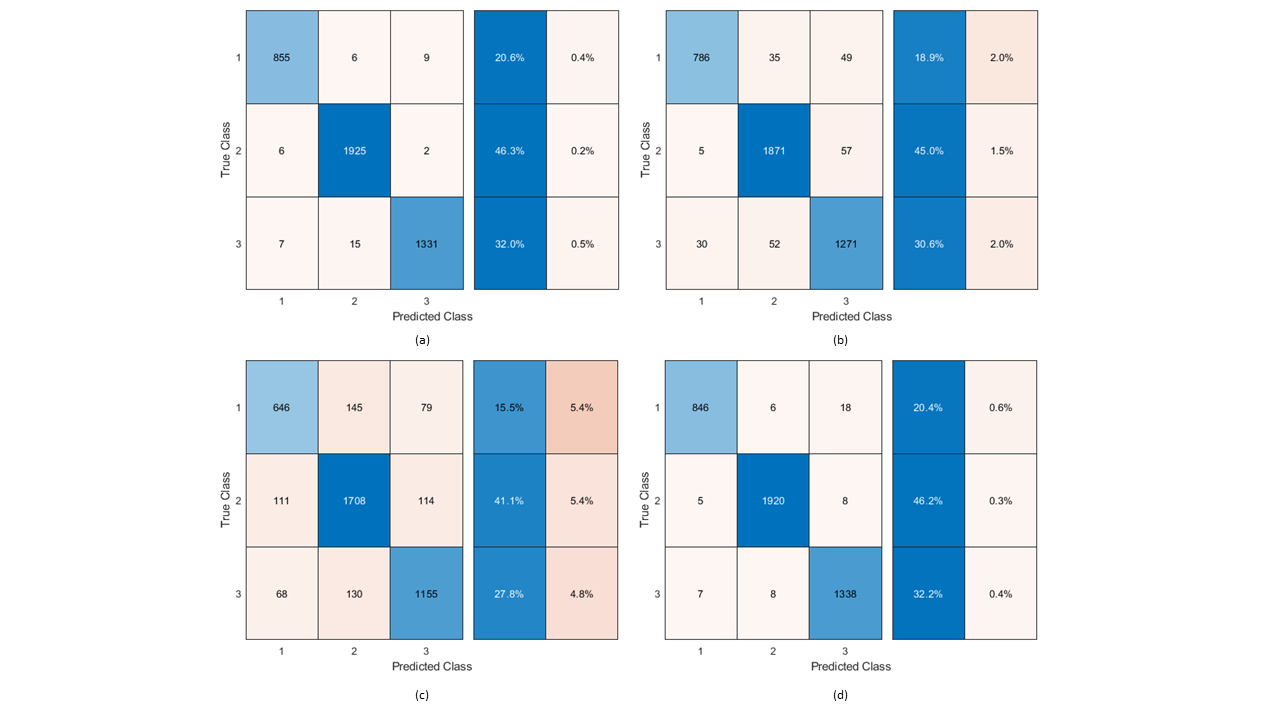


**Supplementary Figure 17.** Confusion matrix of classifiers on chronic back pain (no pain-free controls included) sub-groups based on body mass index and grip strength with (a) Support Vector Machine, (b) Naïve Bayes, (c) k-Nearest Neighbour and (d) Random Forest classifiers. The x-axis is the predicted class while the y-axis is the true class. Blue squares indicate the number in the class that was accurately classified, while the oranges squares show the number of misclassifications. The boxes on the right of the matrix show the percentage of classification (blue) and misclassification (orange) for the class. Classes are (1) higher body mass index and low grip strength (n=870; 20.9%); (2) lower body mass index and grip strength (n=1,933; 46.5%); and (3) lower body mass index and higher grip strength (n=1,353; 32.6%)


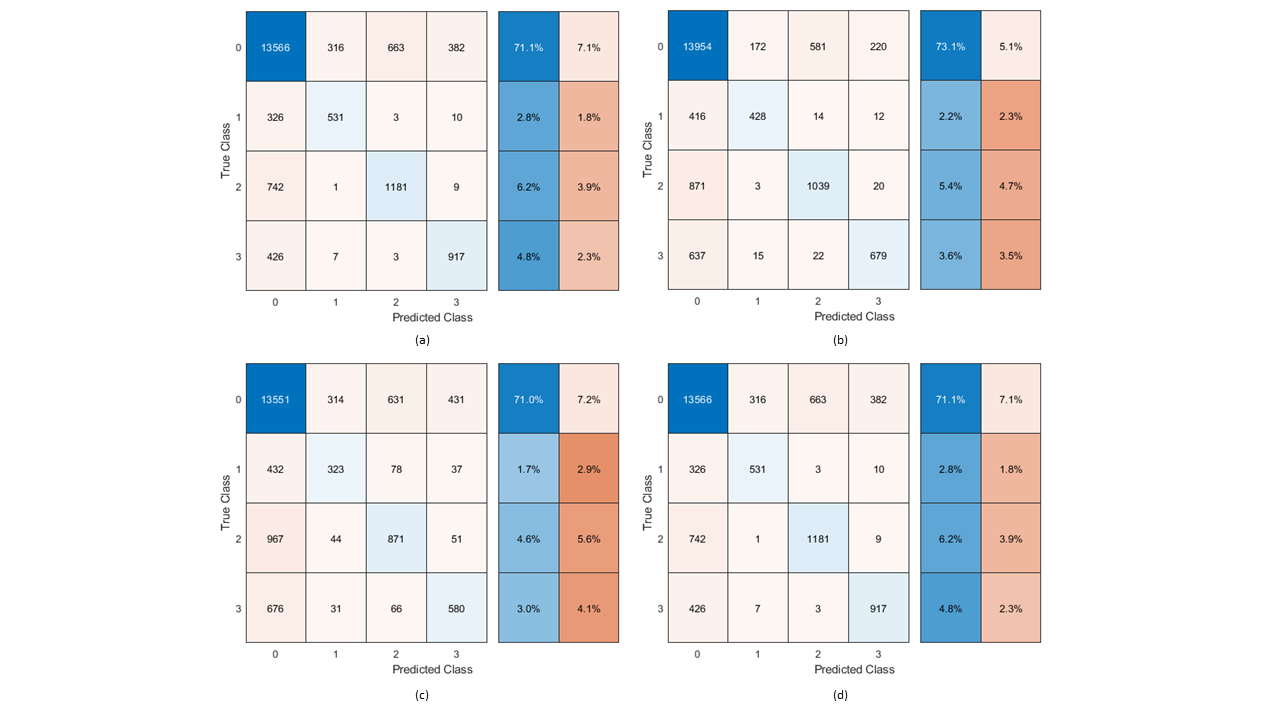


**Supplementary Figure 18.** Confusion matrix of classifiers on chronic back pain and pain-free sub-groups based on body mass index and grip strength with (a) Support Vector Machine, (b) Naïve Bayes, (c) k-Nearest Neighbour and (d) Random Forest classifiers. The x-axis is the predicted class while the y-axis is the true class. Blue squares indicate the number in the class that was accurately classified, while the oranges squares show the number of misclassifications. The boxes on the right of the matrix show the percentage of classification (blue) and misclassification (orange) for the class. Classes are (0) pain-free individuals (n=14,927; 78.2%); (1) higher body mass index and low grip strength (n=870; 4.6%); (2) lower body mass index and grip strength (n=1,933; 10.1%); and (3) lower body mass index and higher grip strength (n=1,353; 7.1%).


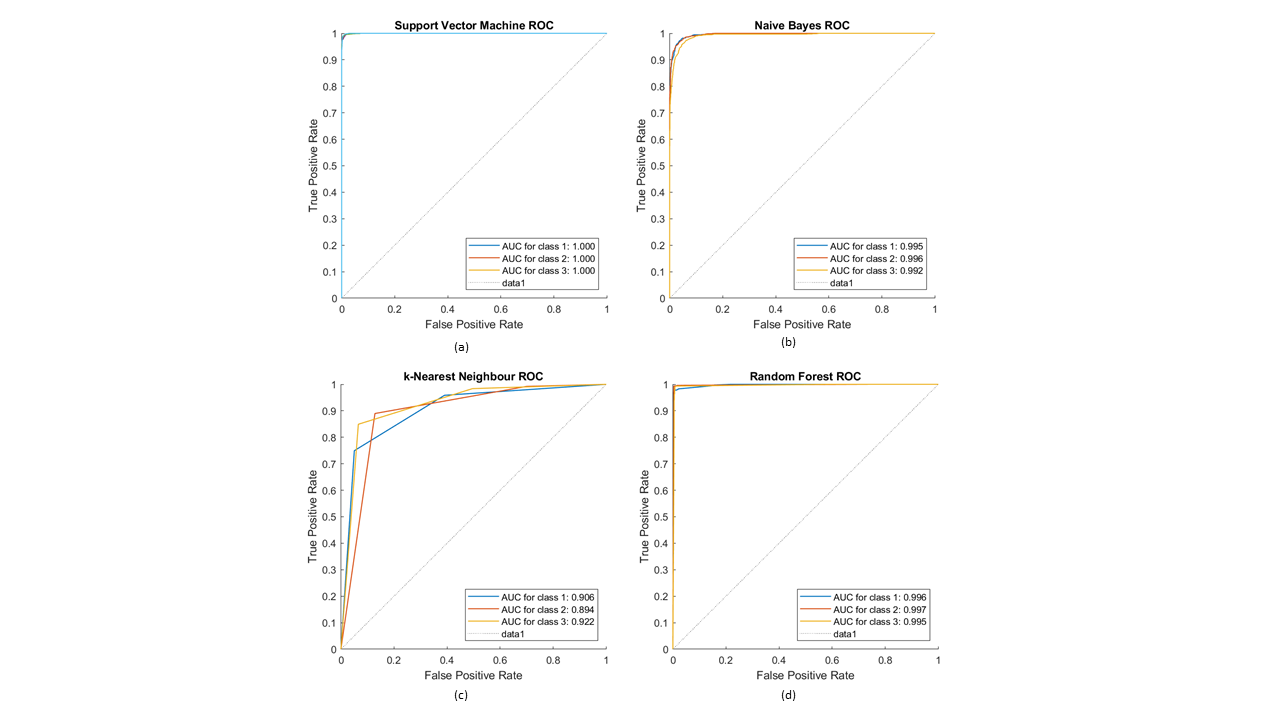


**Supplementary Figure 19.** Class specific area under the curve metrics data for classifiers in the physical sub-domain analysis on CBP individuals only.


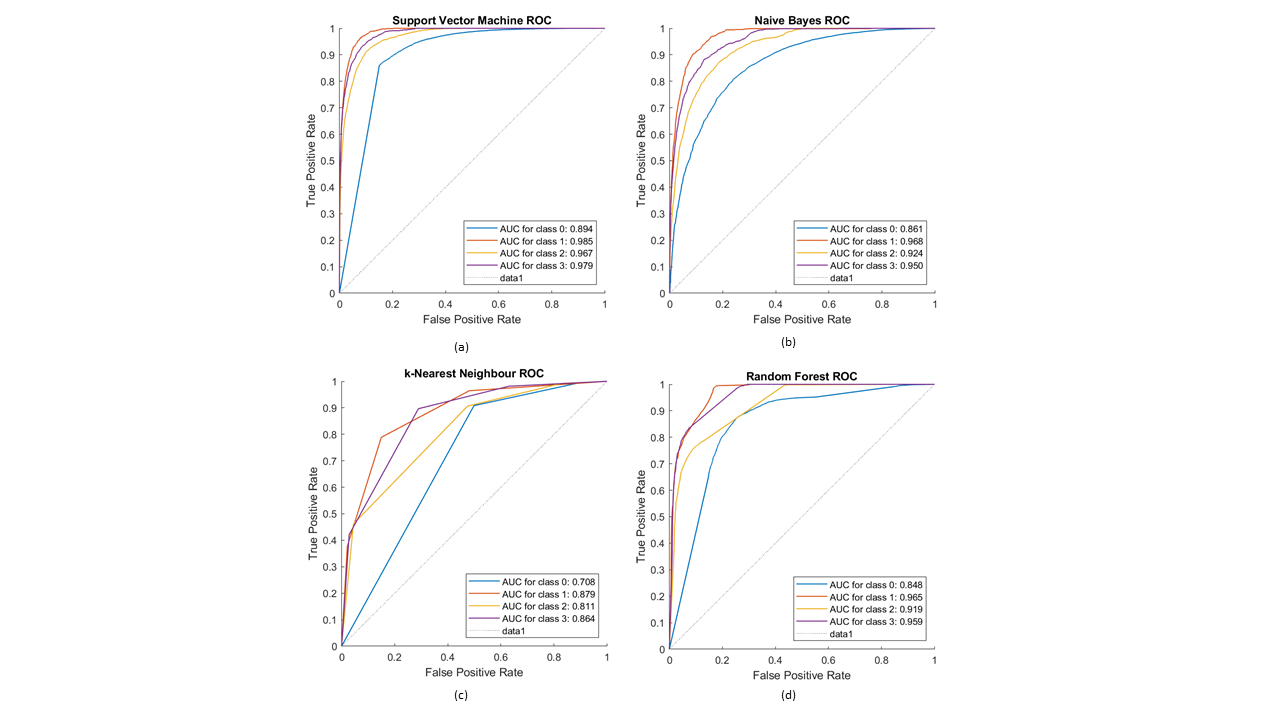


**Supplementary Figure 20.** Class specific area under the curve metrics data for classifiers in the physical sub-domain analysis on CBP and pain-free individuals.


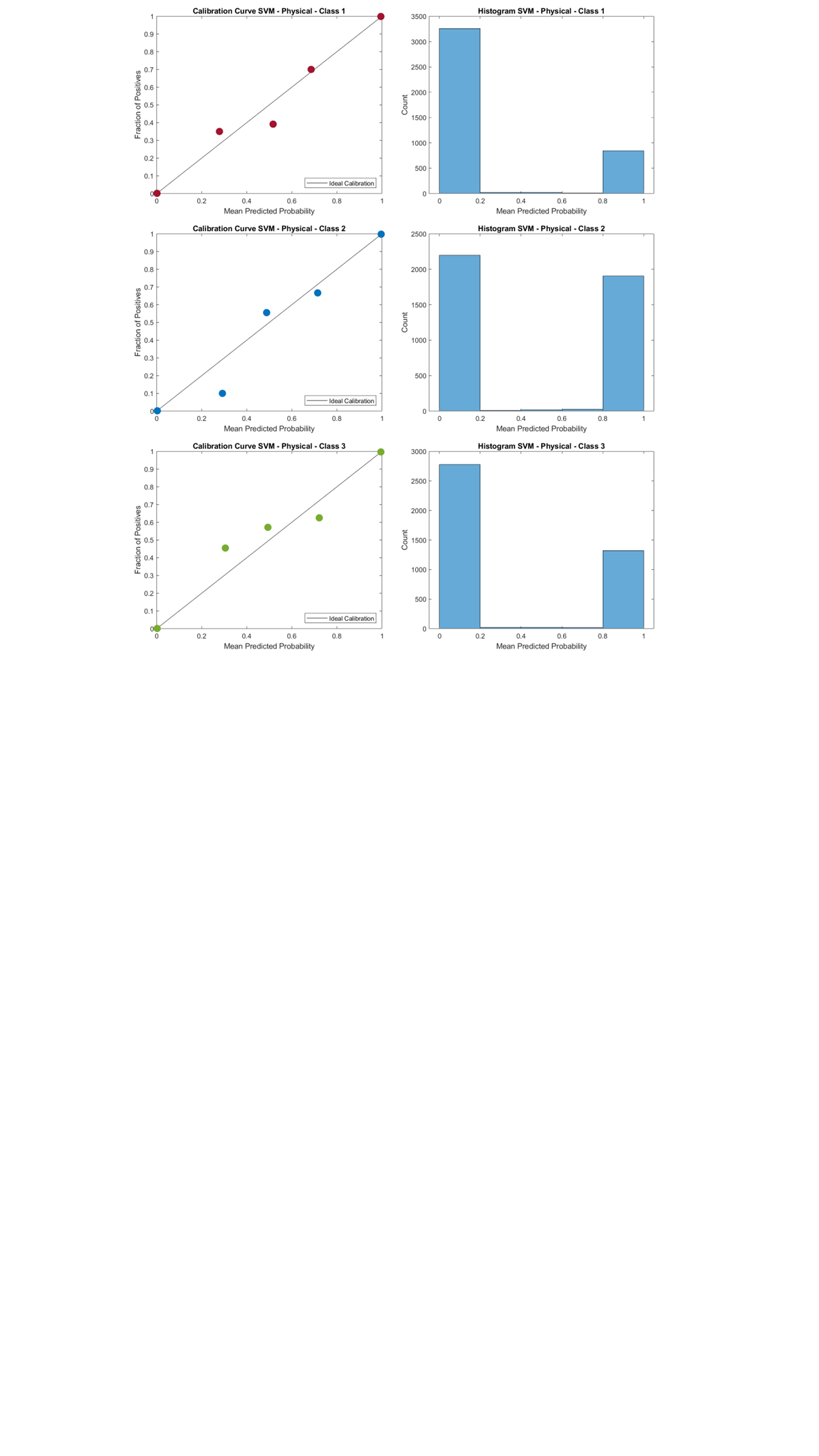


**Supplementary Figure 21.** Support vector machine calibration curves for each class for classifiers in the physical sub-domain analysis on CBP individuals only.


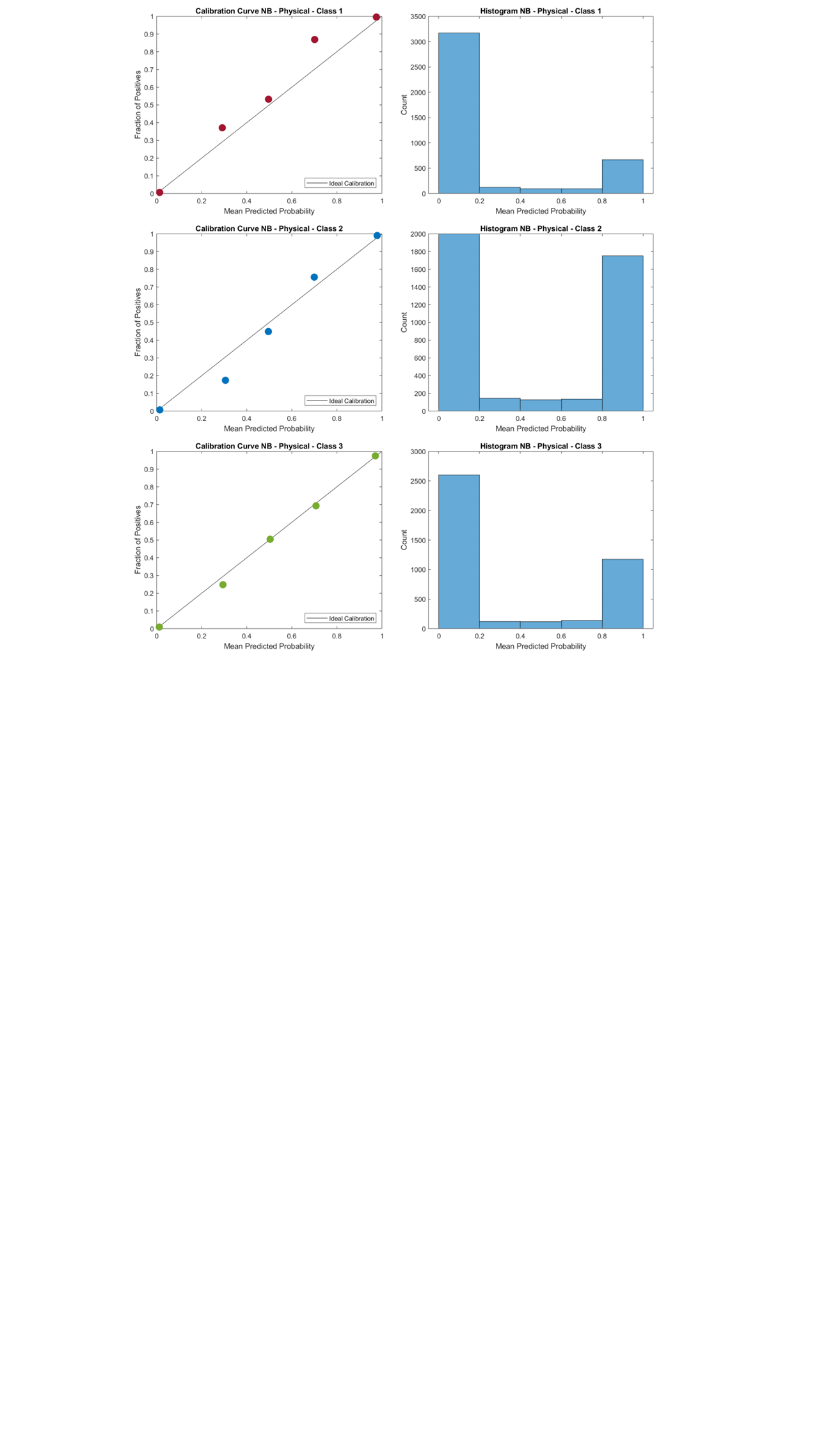


**Supplementary Figure 22.** Naïve Bayes calibration curves for each class for classifiers in the physical sub-domain analysis on CBP individuals only.


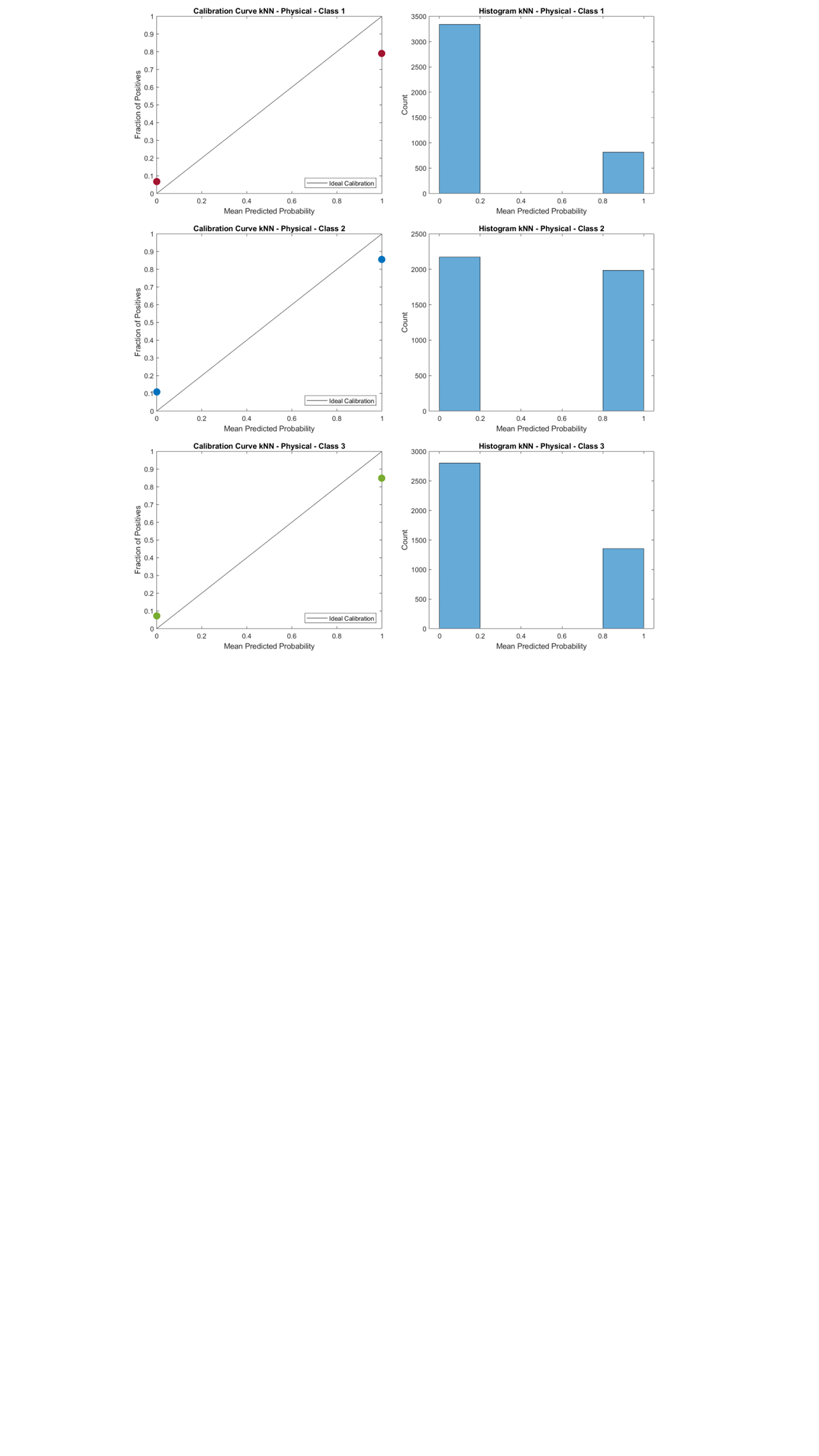


**Supplementary Figure 23.** K-Nearest Neighbour calibration curves for each class for classifiers in the physical sub-domain analysis on CBP individuals only.


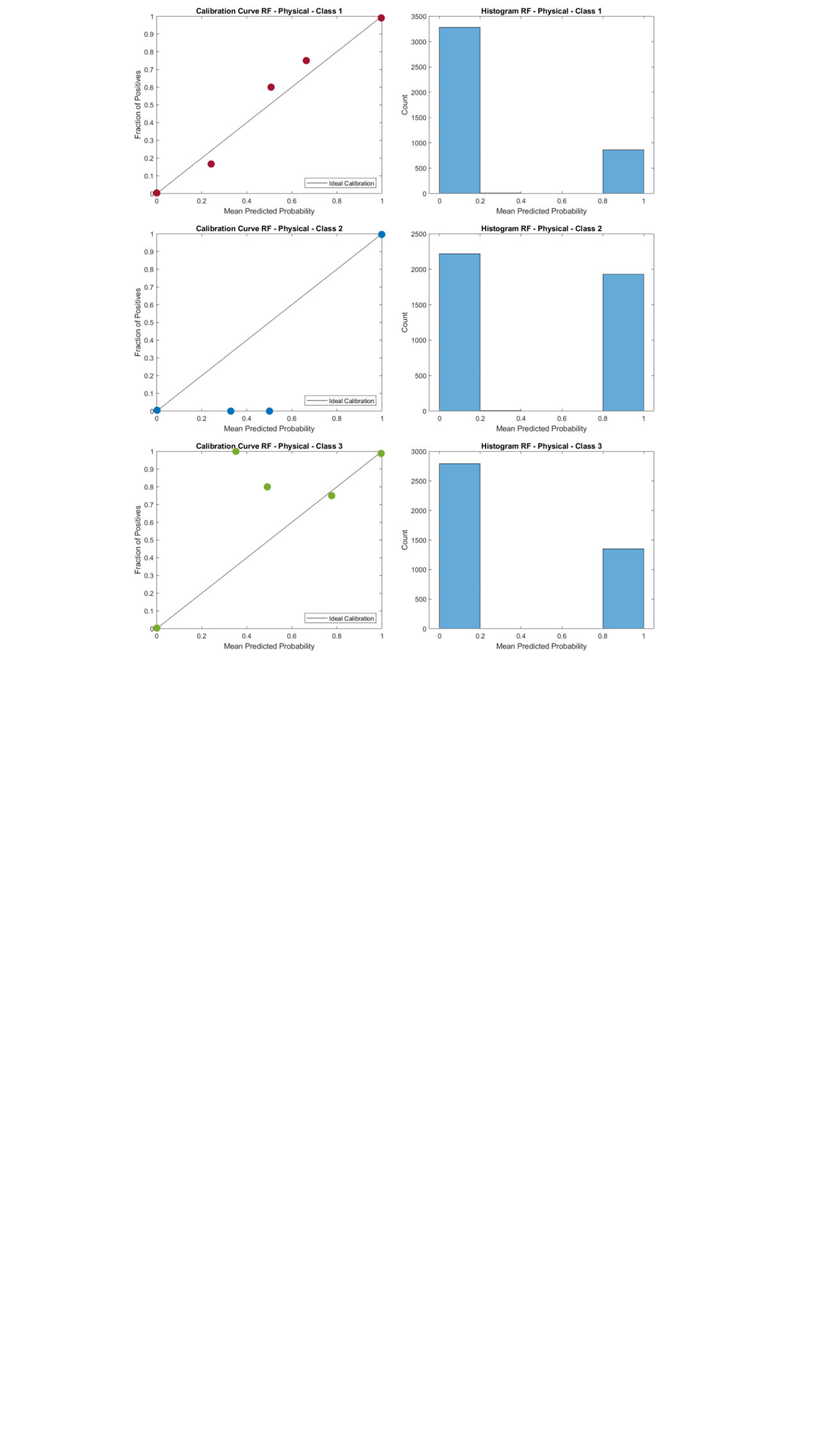


**Supplementary Figure 24.** Random Forest calibration curves for each class for classifiers in the physical sub-domain analysis on CBP individuals only.


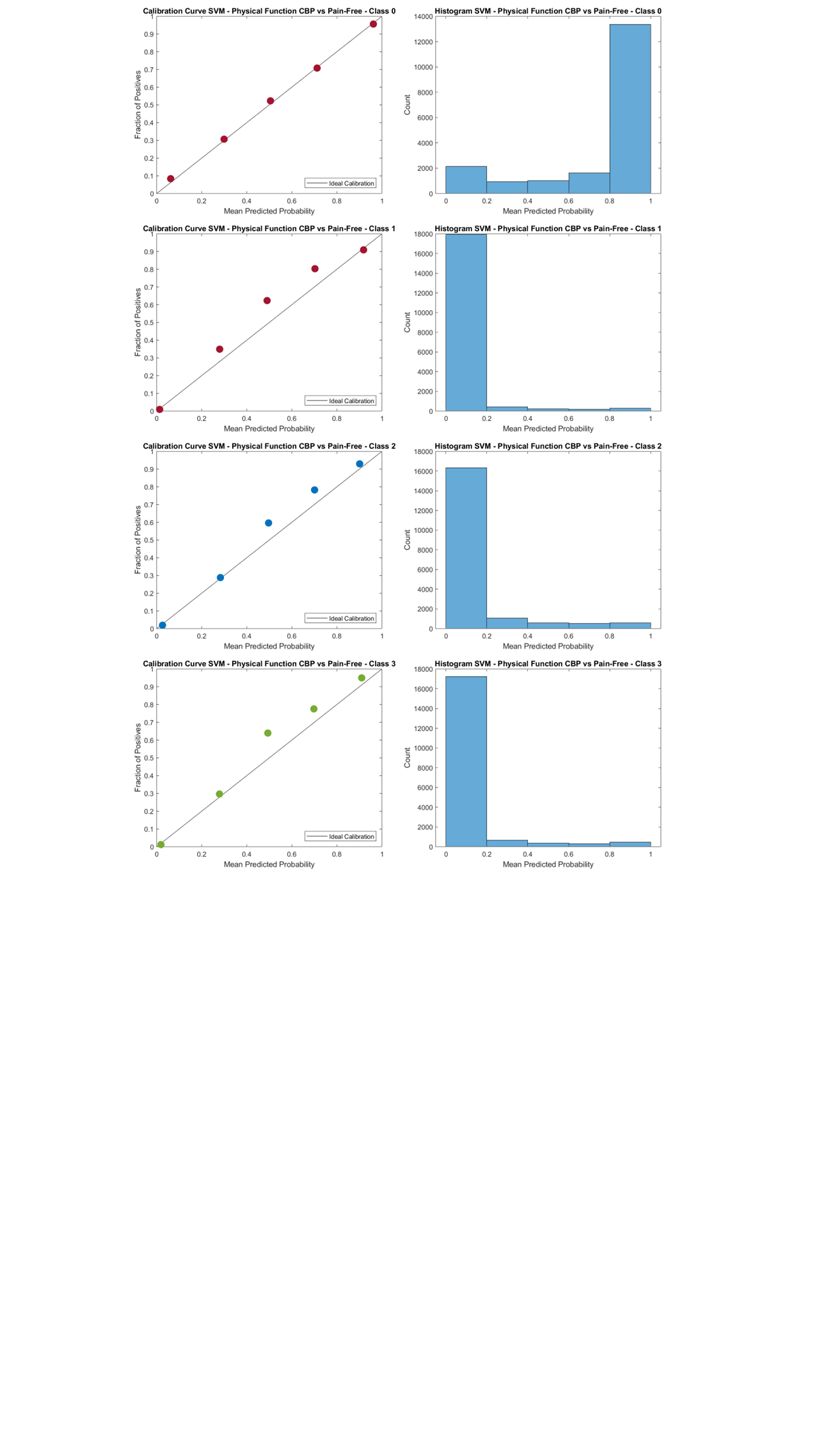


**Supplementary Figure 25.** Support vector machine calibration curves for each class for classifiers in the physical sub-domain analysis on CBP and pain-free individuals.


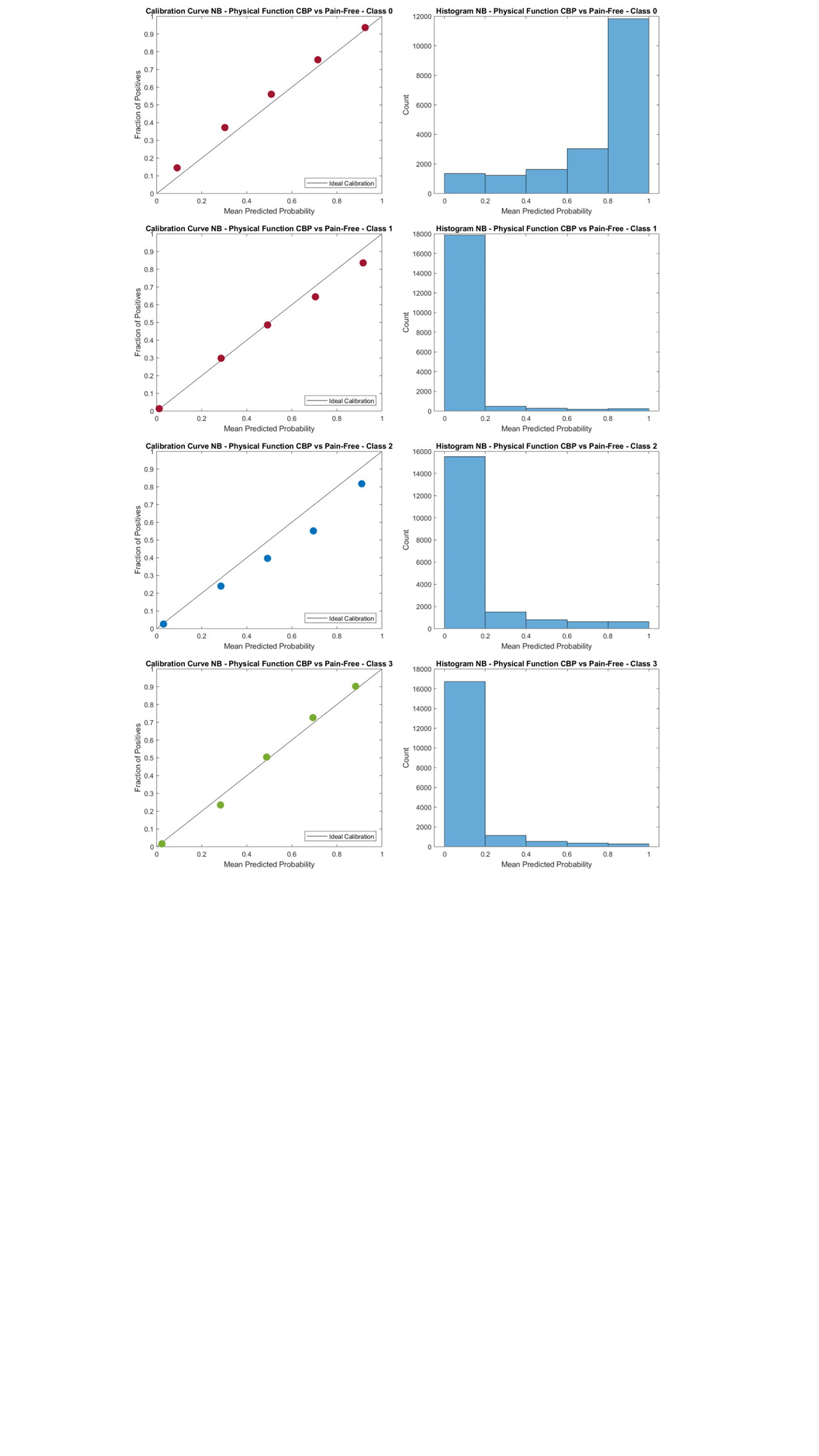


**Supplementary Figure 26.** Naïve Bayes calibration curves for each class for classifiers in the physical sub-domain analysis on CBP and pain-free individuals.


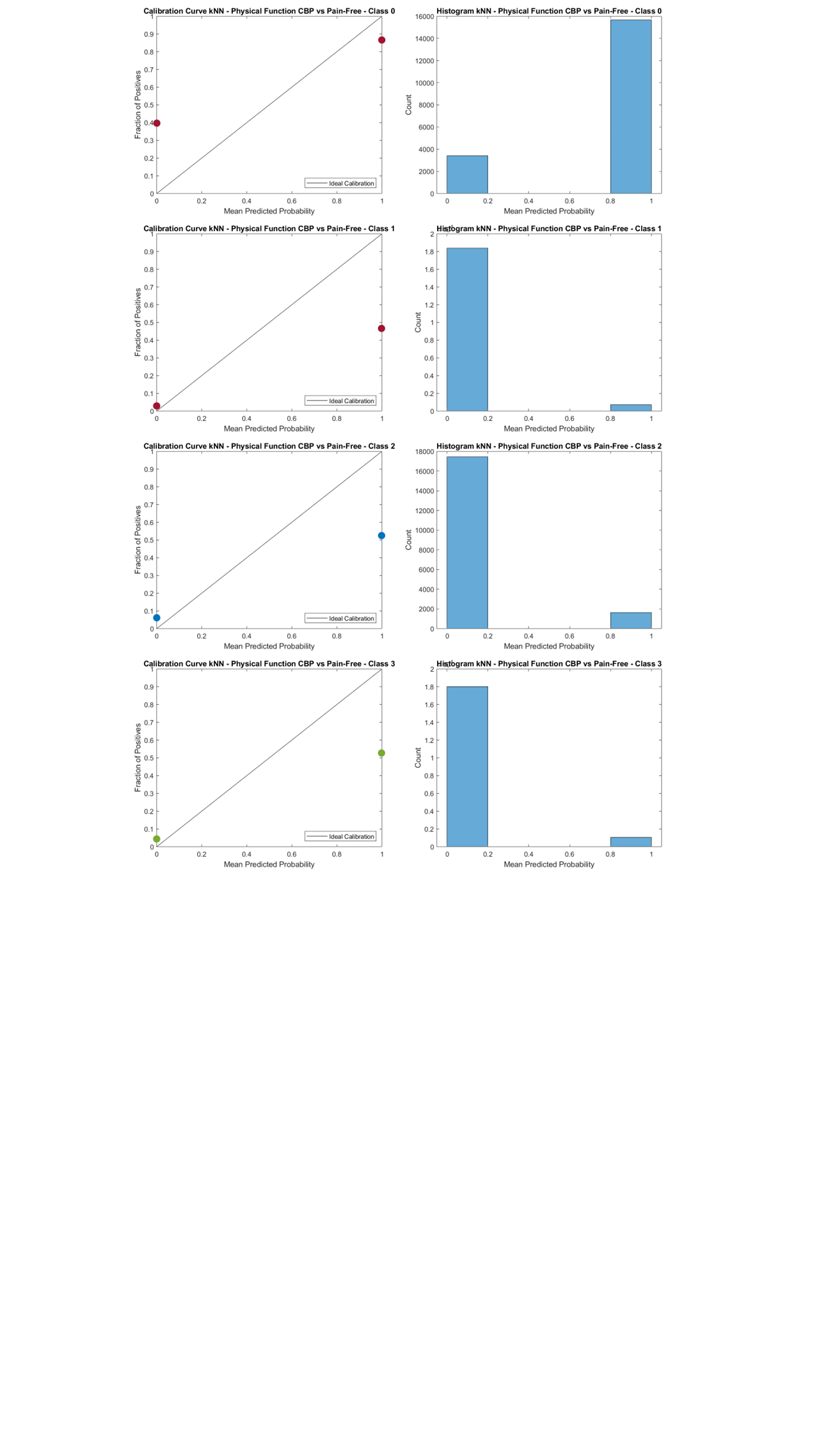


**Supplementary Figure 27.** K-Nearest Neighbour calibration curves for each class for classifiers in the physical sub-domain analysis on CBP and pain-free individuals.


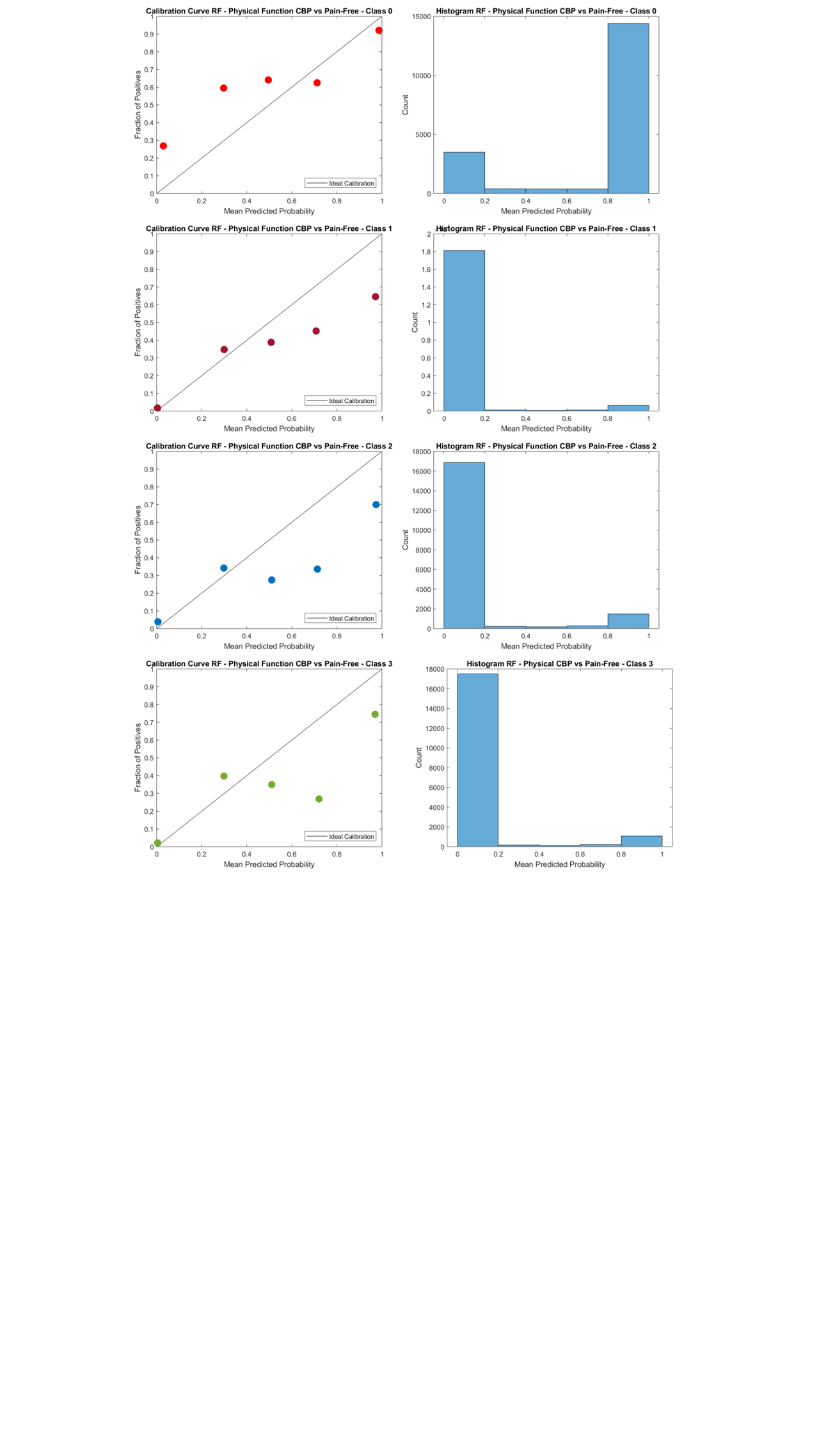


**Supplementary Figure 28.** Random Forest calibration curves for each class for classifiers in the physical sub-domain analysis on CBP and pain-free individuals.


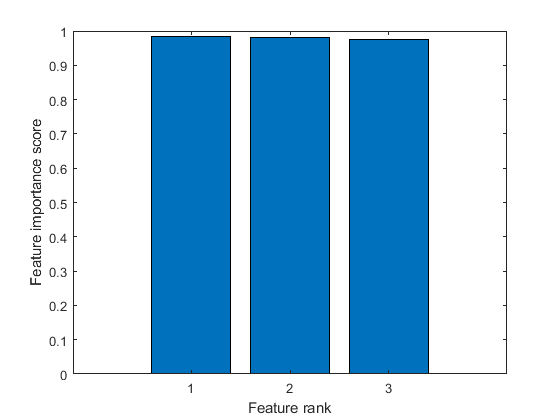


**Supplementary Figure 29.** Laplacian scores to rank brain function features in the order of importance based on how they explain differences in datapoints in a single cohort (e.g. CBP only). For this data, the order of importance of features in a CBP-only space was inferior parietal lobe to middle temporal gyrus connectivity (node 1014), inferior parietal love to superior temporal gyrus connectivity (node 222) and calcarine cortex to precuneus connectivity (node 44).


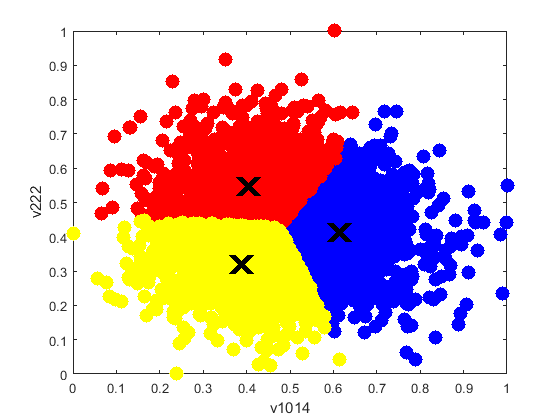


**Supplementary Figure 30.** Scatter plot of the distribution of sub-groups of individuals with CBP based on inferior parietal lobe to middle temporal gyrus connectivity (node 1014) and inferior parietal love to superior temporal gyrus connectivity (node 222. Data is presented on (a) normal item range and (b) normalised scale of 0-1. Based on the centroids of fuzzy c-means clustering, classes and colours are (1; blue) High FPN-DMN connectivity (n=1,347; 32.4%); (2; red) High FPN-SMN connectivity (n=1,335; 32.1%); and (3; yellow) Low FPN-DMN and FPN-SMN network connectivity (n=1,474; 35.5%). Black squares on the heat map indicate no class was available at those values. The X value on the scatter plot indicates the centroid of that cluster.


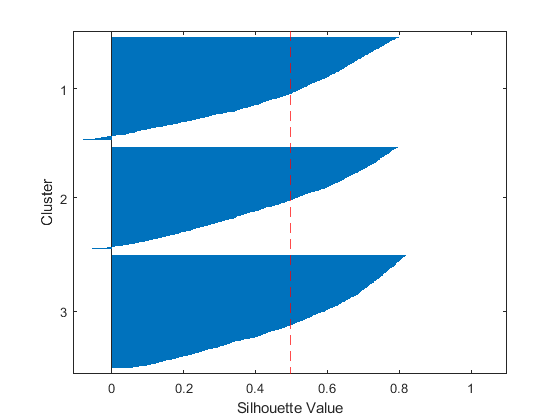


**Supplementary Figure 31.** Post clustering evaluation for clustering consisting of brain function variables. The red line indicates the average of all the similarity measures. Values of 1 indicate good similarity of a datapoint to its cluster. The average Silhouette value is 0.49


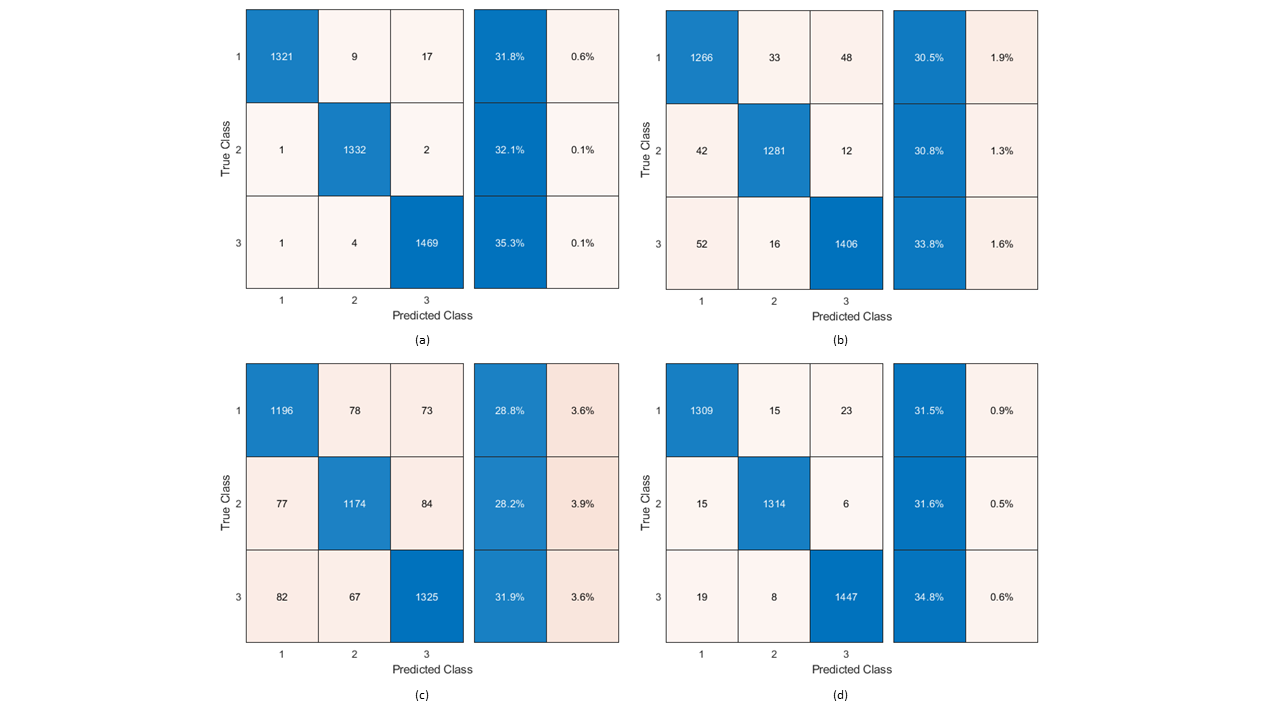


**Supplementary Figure 32.** Confusion matrix of classifiers on chronic back pain sub-groups (no pain-free controls included) based on brain function with (a) Support Vector Machine, (b) Naïve Bayes, (c) k-Nearest Neighbour and (d) Random Forest classifiers. The x-axis is the predicted class while the y-axis is the true class. Blue squares indicate the number in the class that was accurately classified, while the oranges squares show the number of misclassifications. The boxes on the right of the matrix show the percentage of classification (blue) and misclassification (orange) for the class. Classes are (1) high FPN-DMN connectivity (n=1,347; 32.4%); (2) high FPN-SMN connectivity (n=1,335; 32.1%); and (3) low FPN-DMN and FPN-SMN network connectivity (n=1,474; 35.5%).


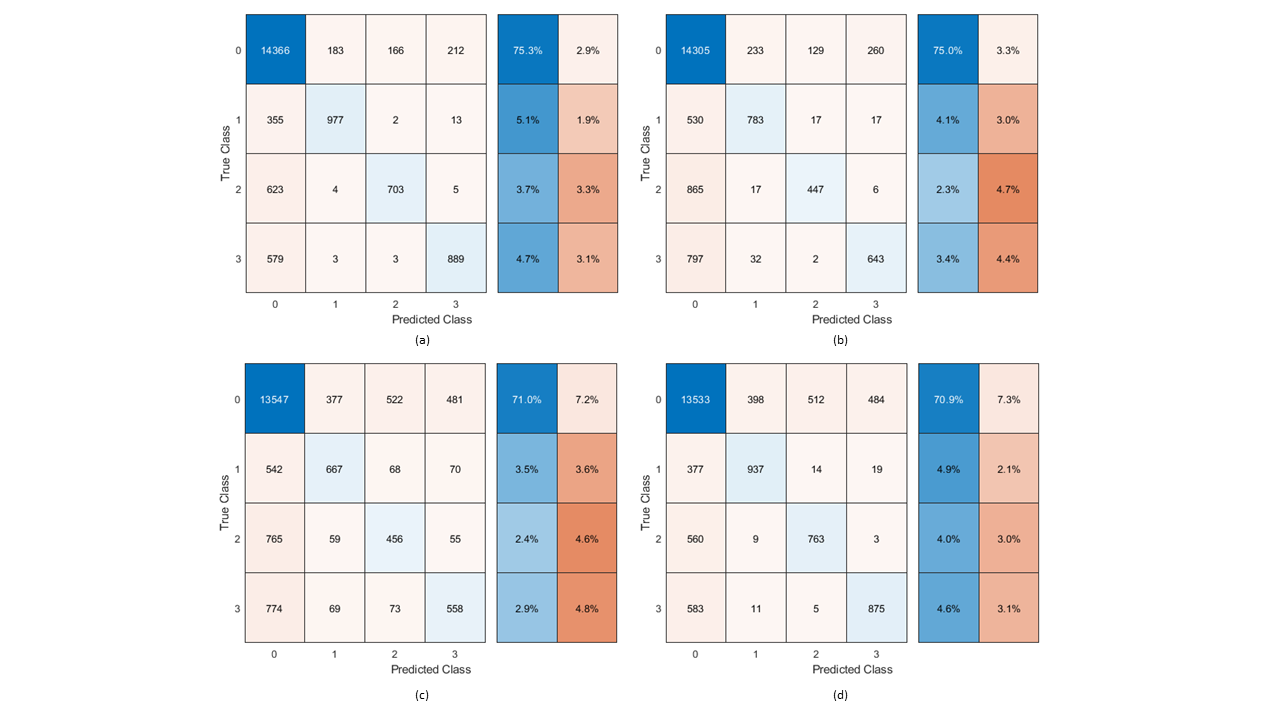


**Supplementary Figure 33.** Confusion matrix of classifiers on chronic back pain and pain-free sub-groups based on brain function with (a) Support Vector Machine, (b) Naïve Bayes, (c) k-Nearest Neighbour and (d) Random Forest classifiers. The x-axis is the predicted class while the y-axis is the true class. Blue squares indicate the number in the class that was accurately classified, while the oranges squares show the number of misclassifications. The boxes on the right of the matrix show the percentage of classification (blue) and misclassification (orange) for the class. Classes are (0) pain-free individuals (n=14,927; 78.2%); (1) high FPN-DMN connectivity (n=1,347; 7.1%); (2) high FPN-SMN connectivity (n=1,335; 7.0%); and (3) low FPN-DMN and FPN-SMN network connectivity (n=1,474; 7.7%).


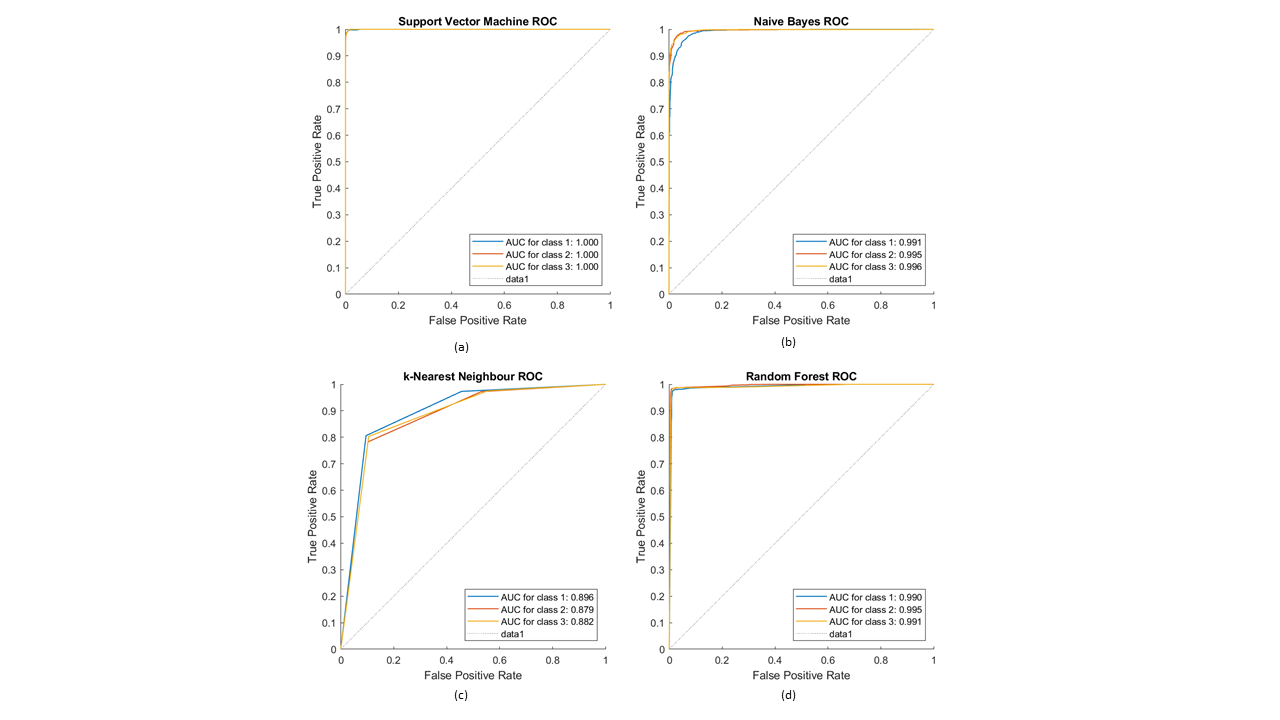


**Supplementary Figure 34.** Class specific area under the curve metrics data for classifiers in the brain function sub-domain analysis on CBP individuals only.


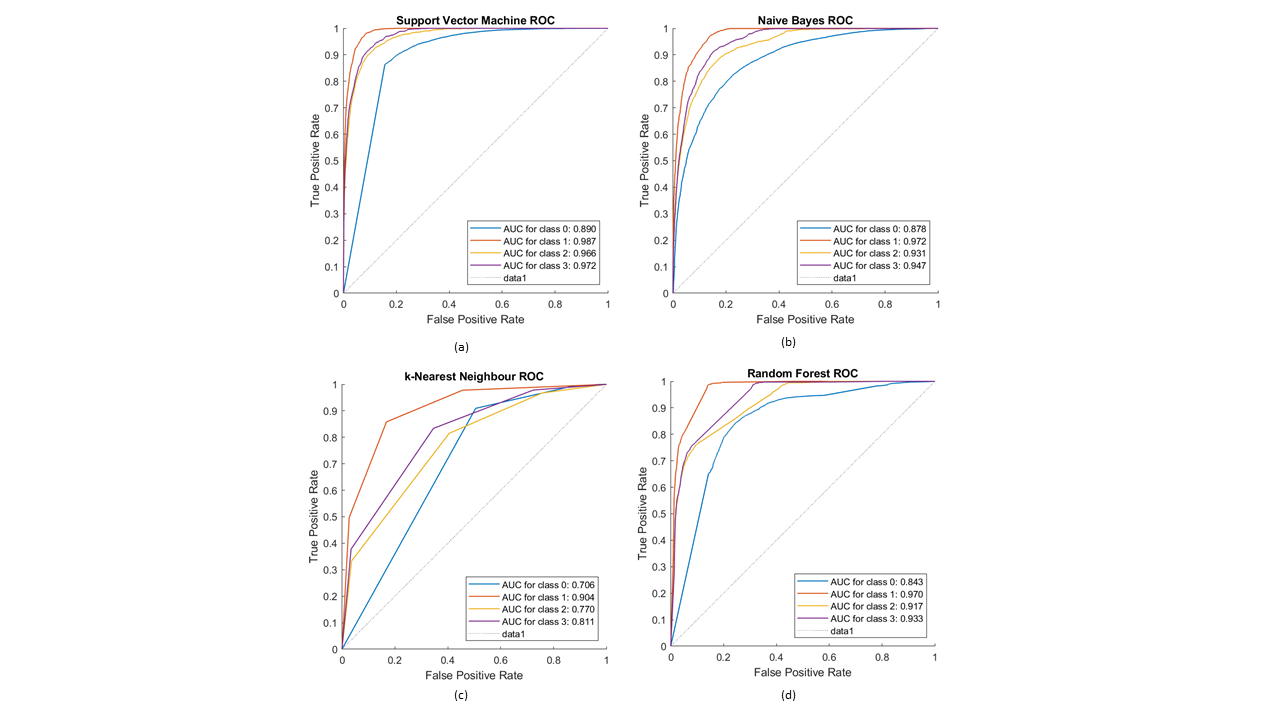


**Supplementary Figure 35.** Class specific area under the curve metrics data for classifiers in the brain function sub-domain analysis on CBP and pain-free individuals.


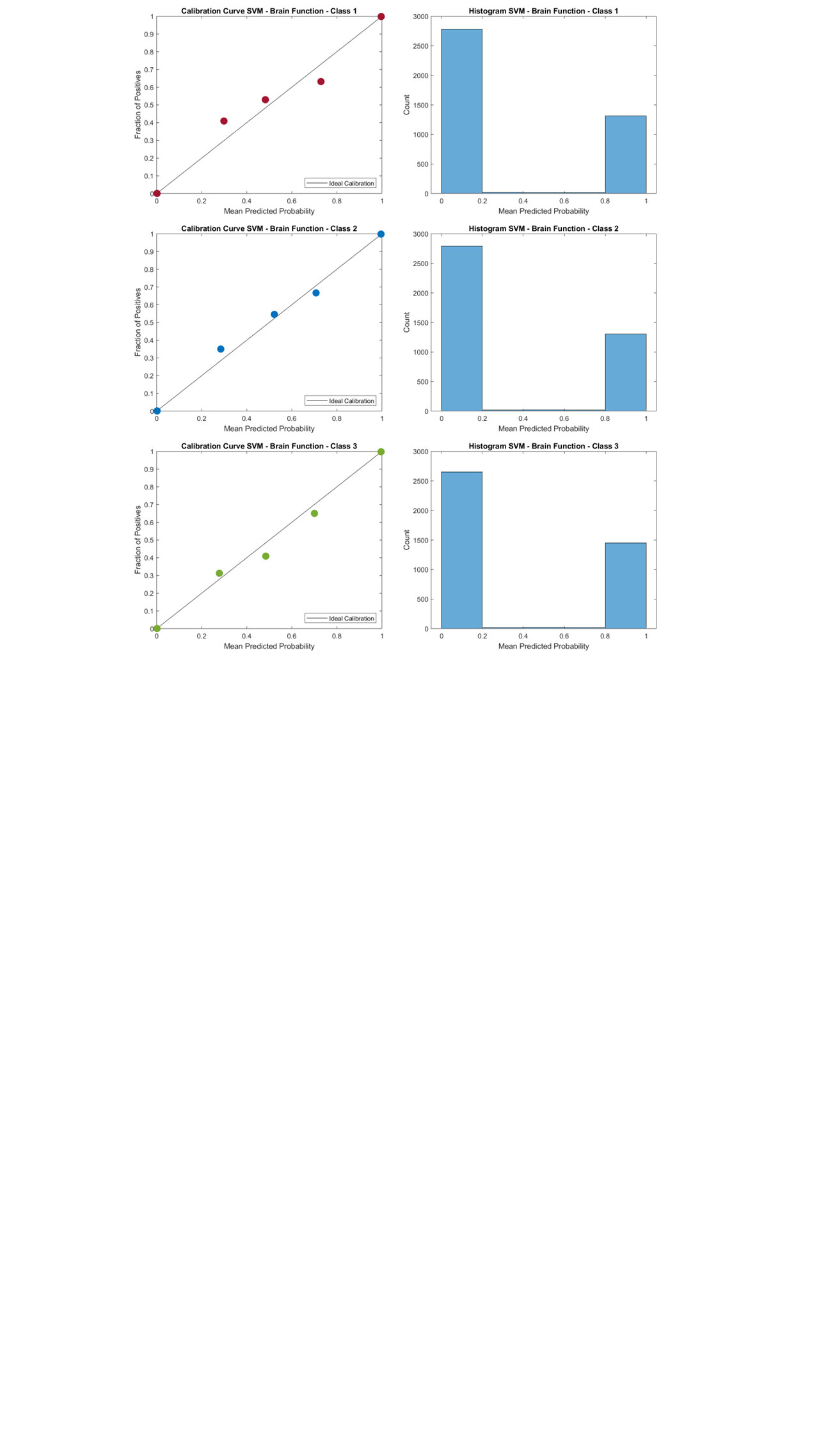


**Supplementary Figure 36.** Support vector machine calibration curves for each class for classifiers in the brain function sub-domain analysis on CBP individuals only.


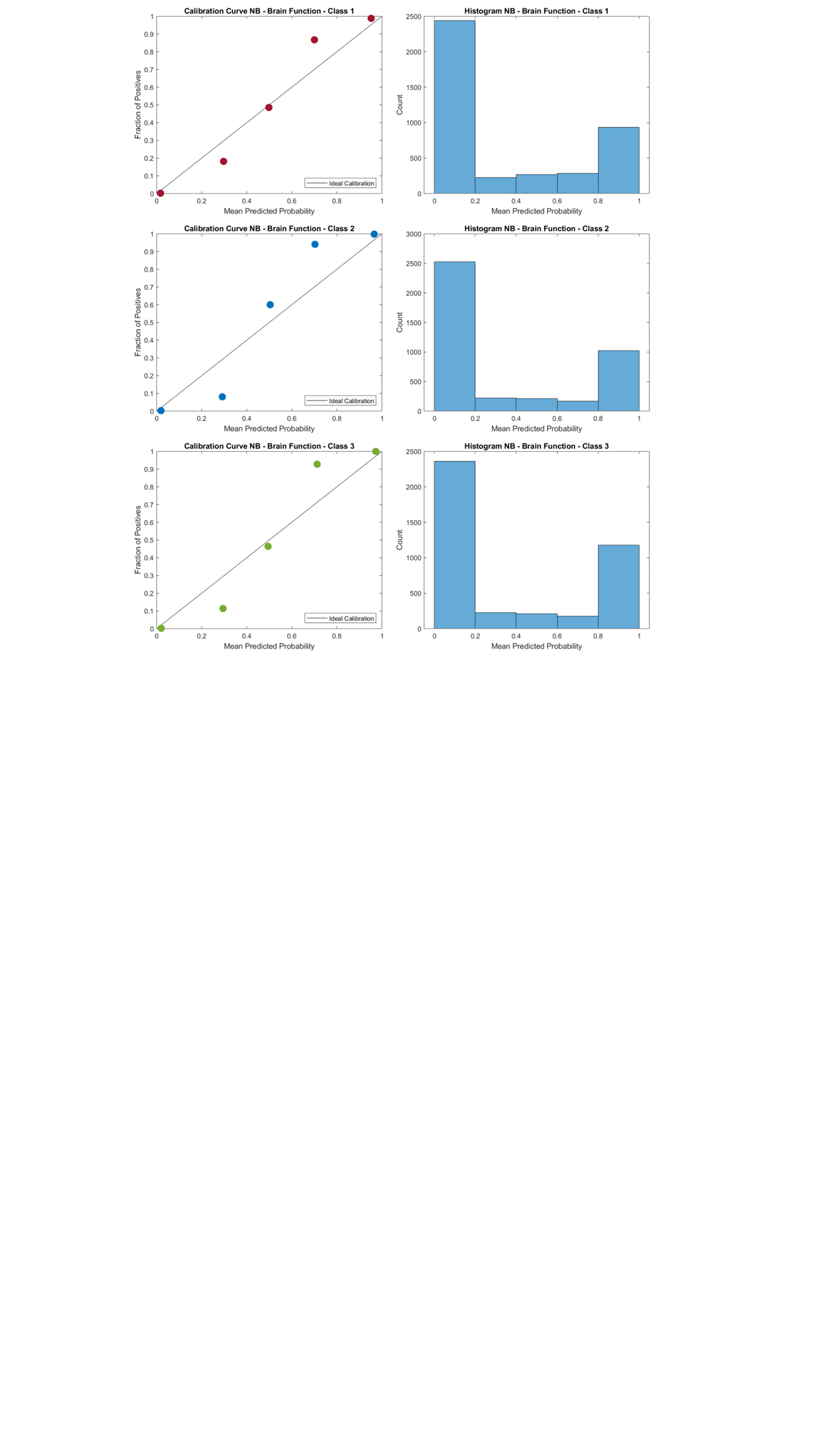


**Supplementary Figure 37.** Naïve Bayes calibration curves for each class for classifiers in the brain function sub-domain analysis on CBP individuals only.


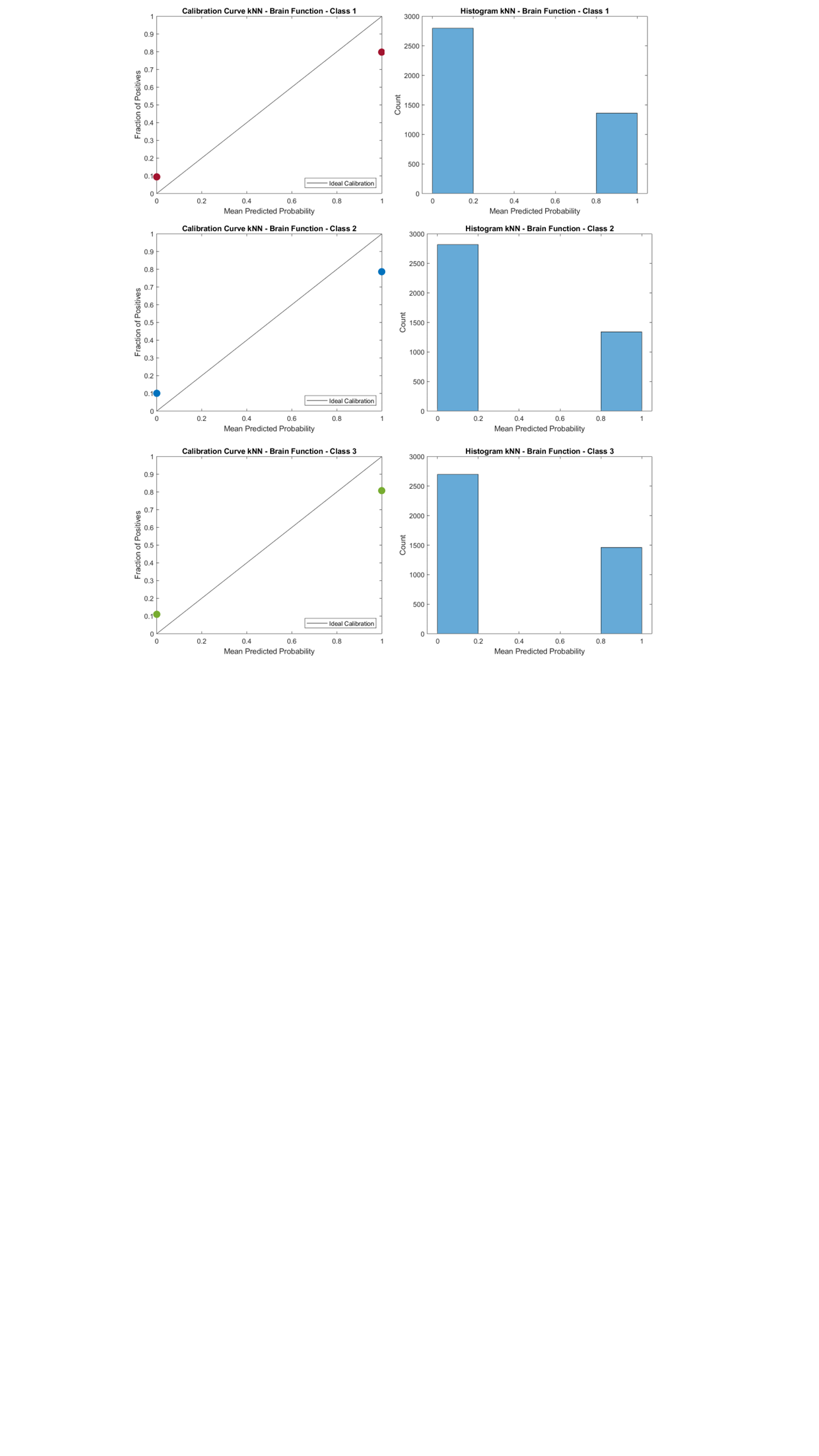


**Supplementary Figure 38.** K-Nearest Neighbour calibration curves for each class for classifiers in the brain function sub-domain analysis on CBP individuals only.


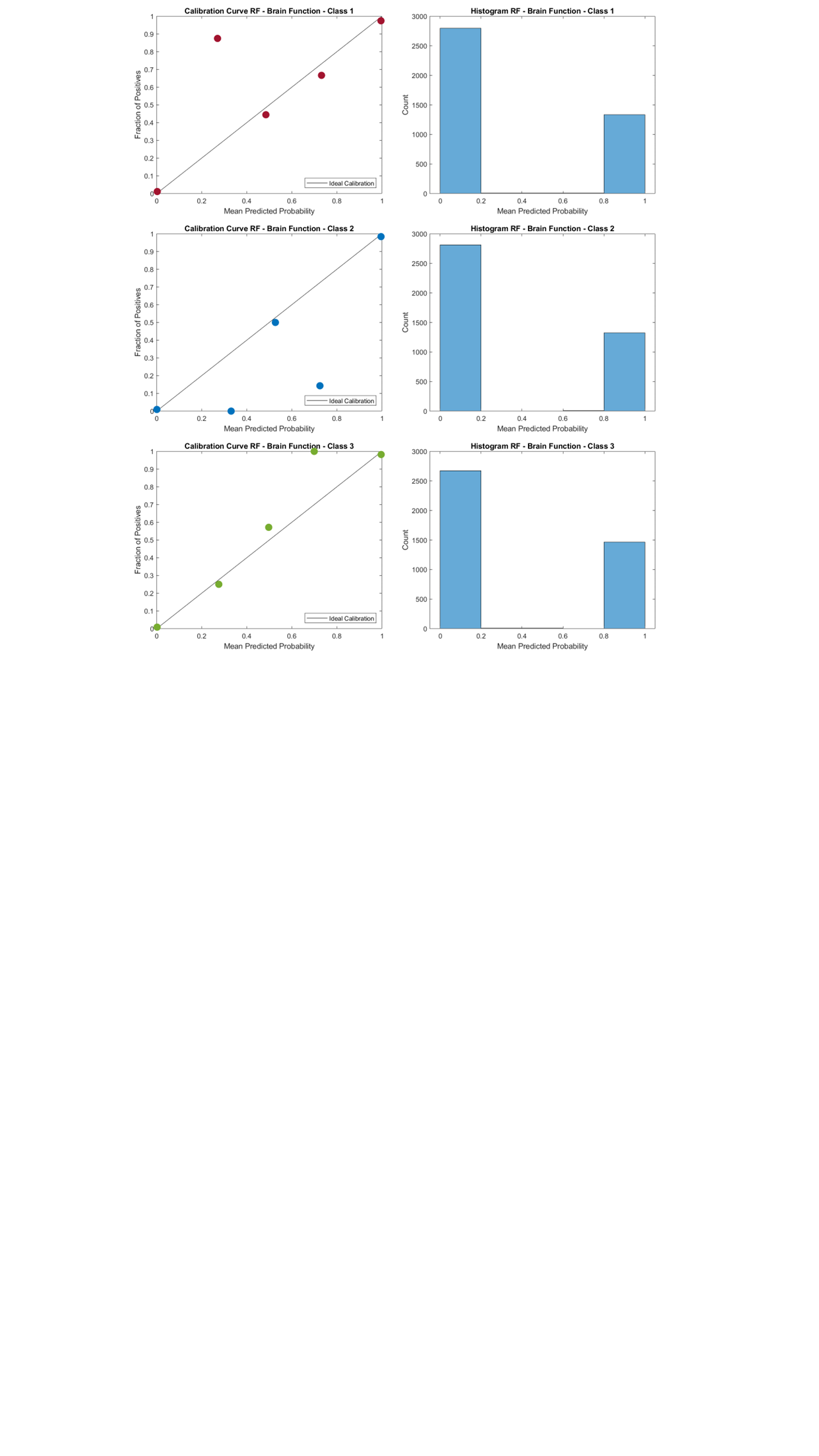


**Supplementary Figure 39.** Random Forest calibration curves for each class for classifiers in the brain function sub-domain analysis on CBP individuals only.


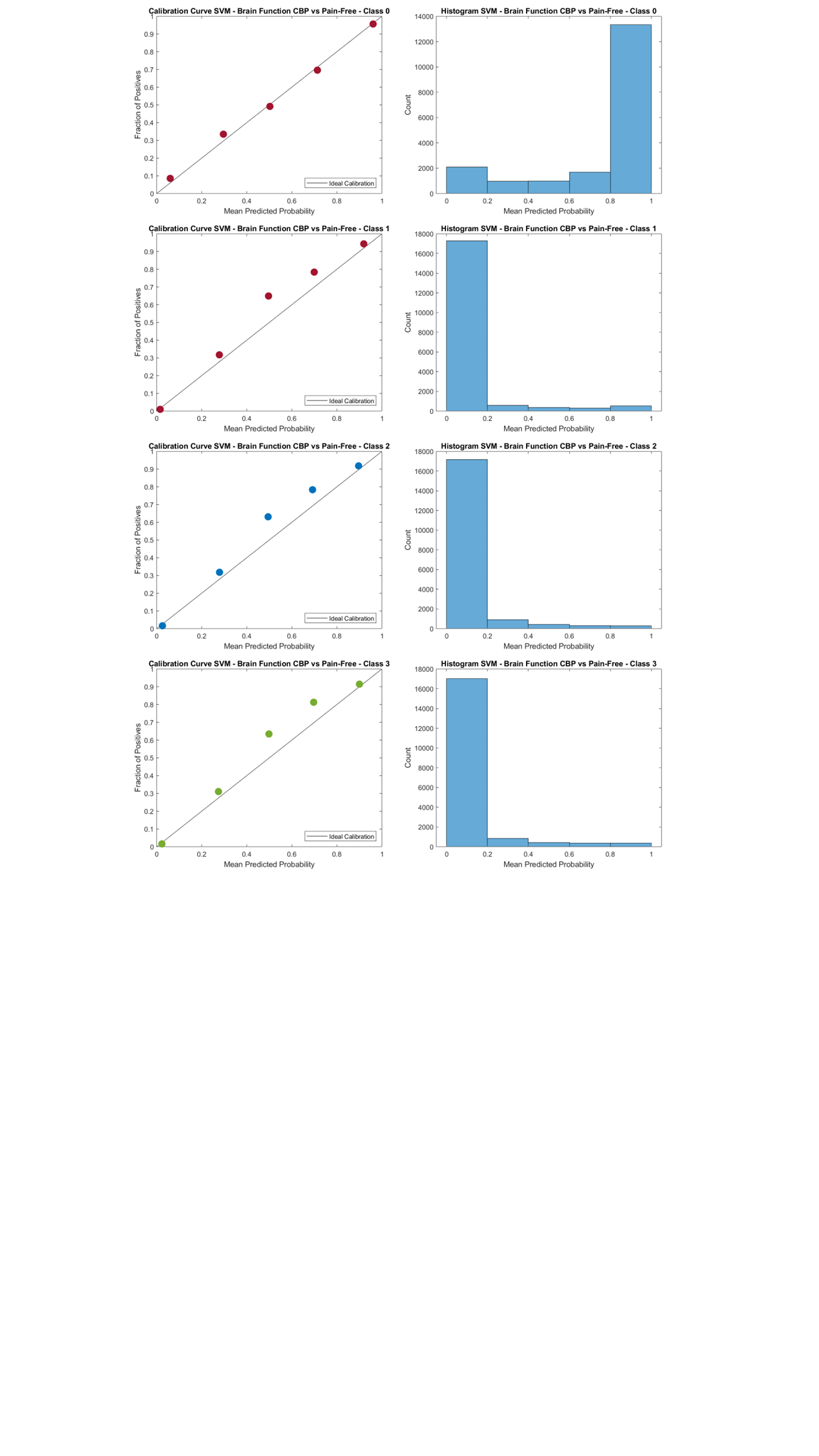


**Supplementary Figure 40.** Support vector machine calibration curves for each class for classifiers in the brain function sub-domain analysis on CBP and pain-free individuals.


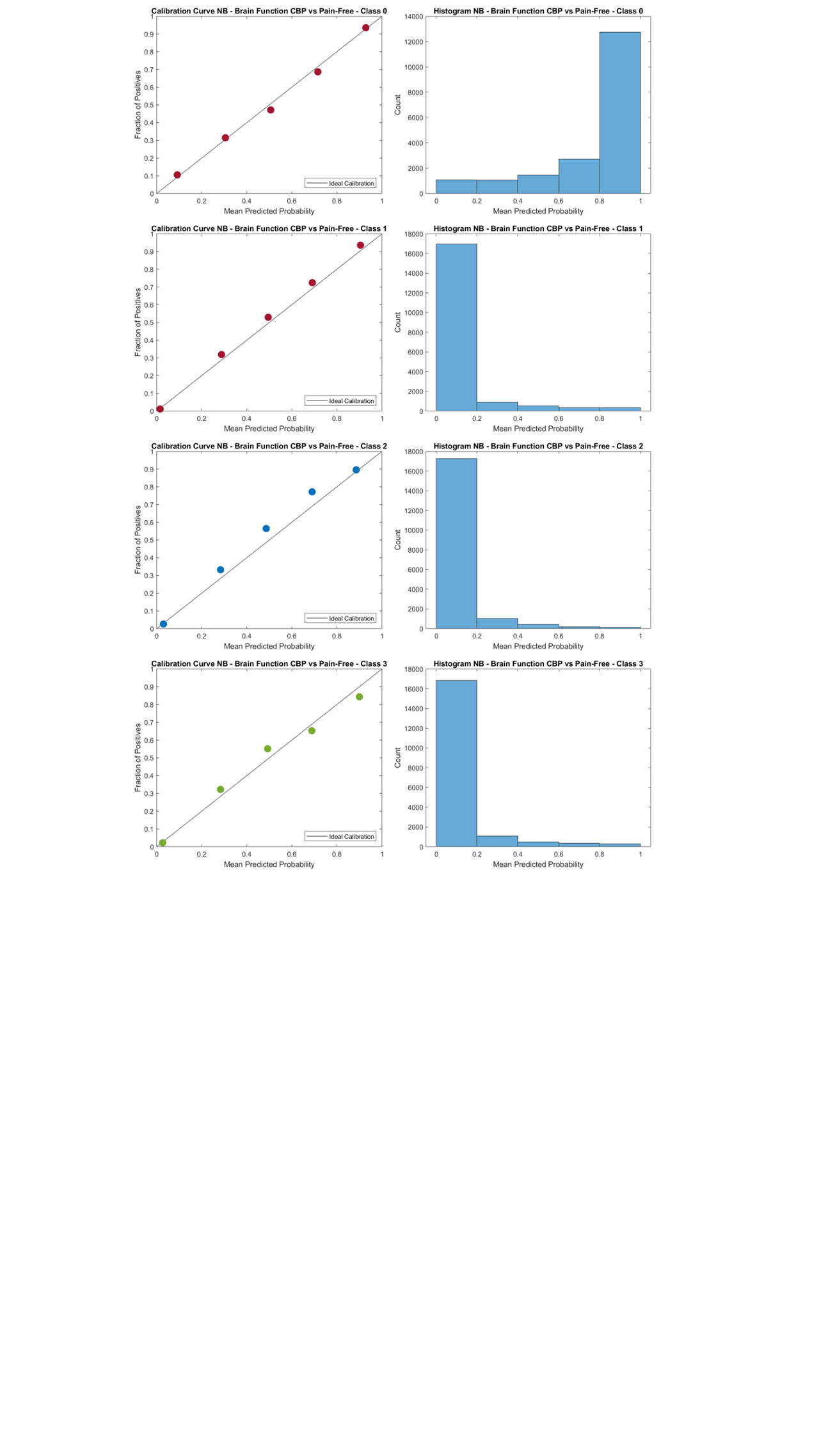


**Supplementary Figure 41.** Naïve Bayes calibration curves for each class for classifiers in the brain function sub-domain analysis on CBP and pain-free individuals.


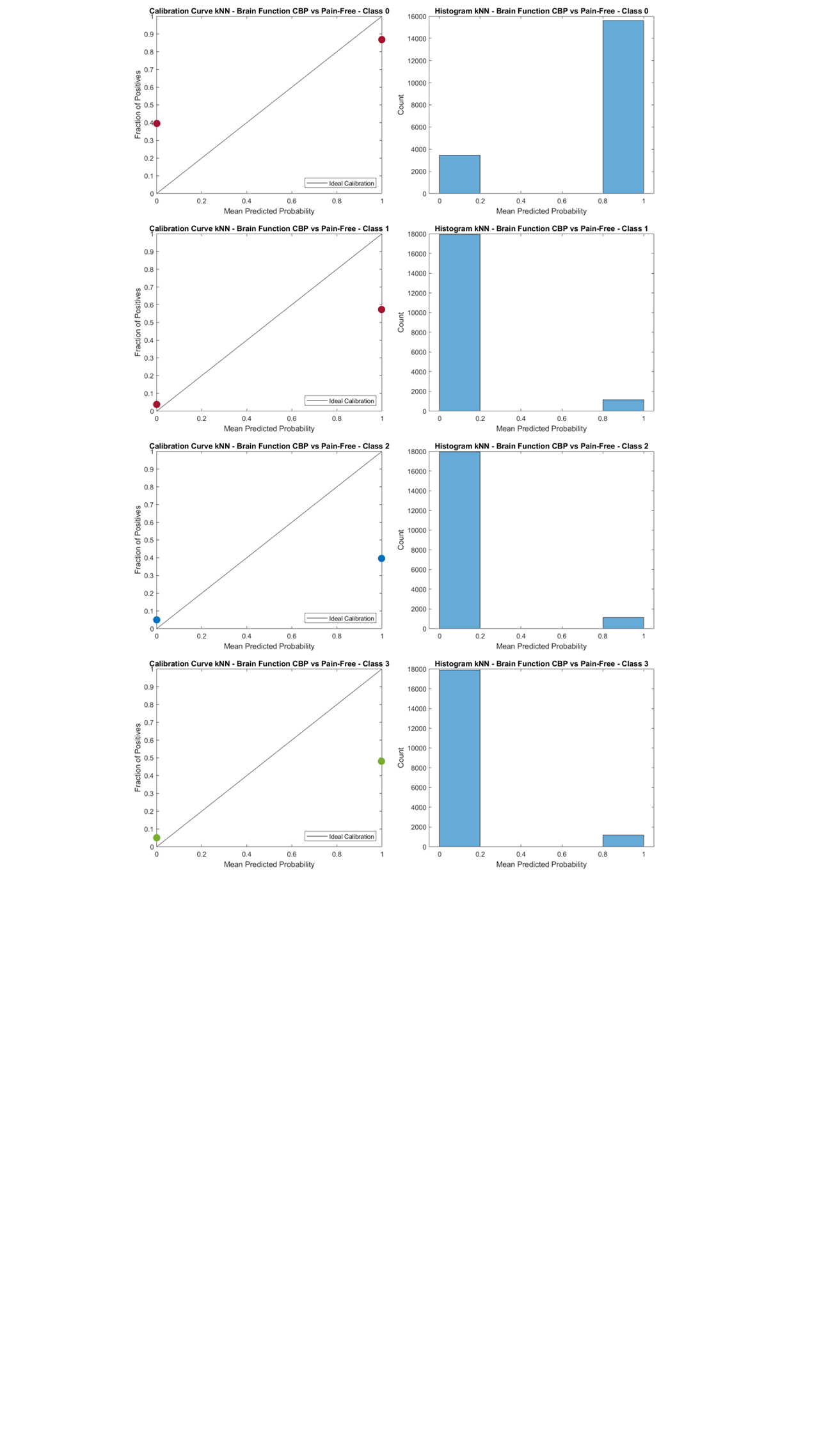


**Supplementary Figure 42.** K-Nearest Neighbour calibration curves for each class for classifiers in the brain function sub-domain analysis on CBP and pain-free individuals.


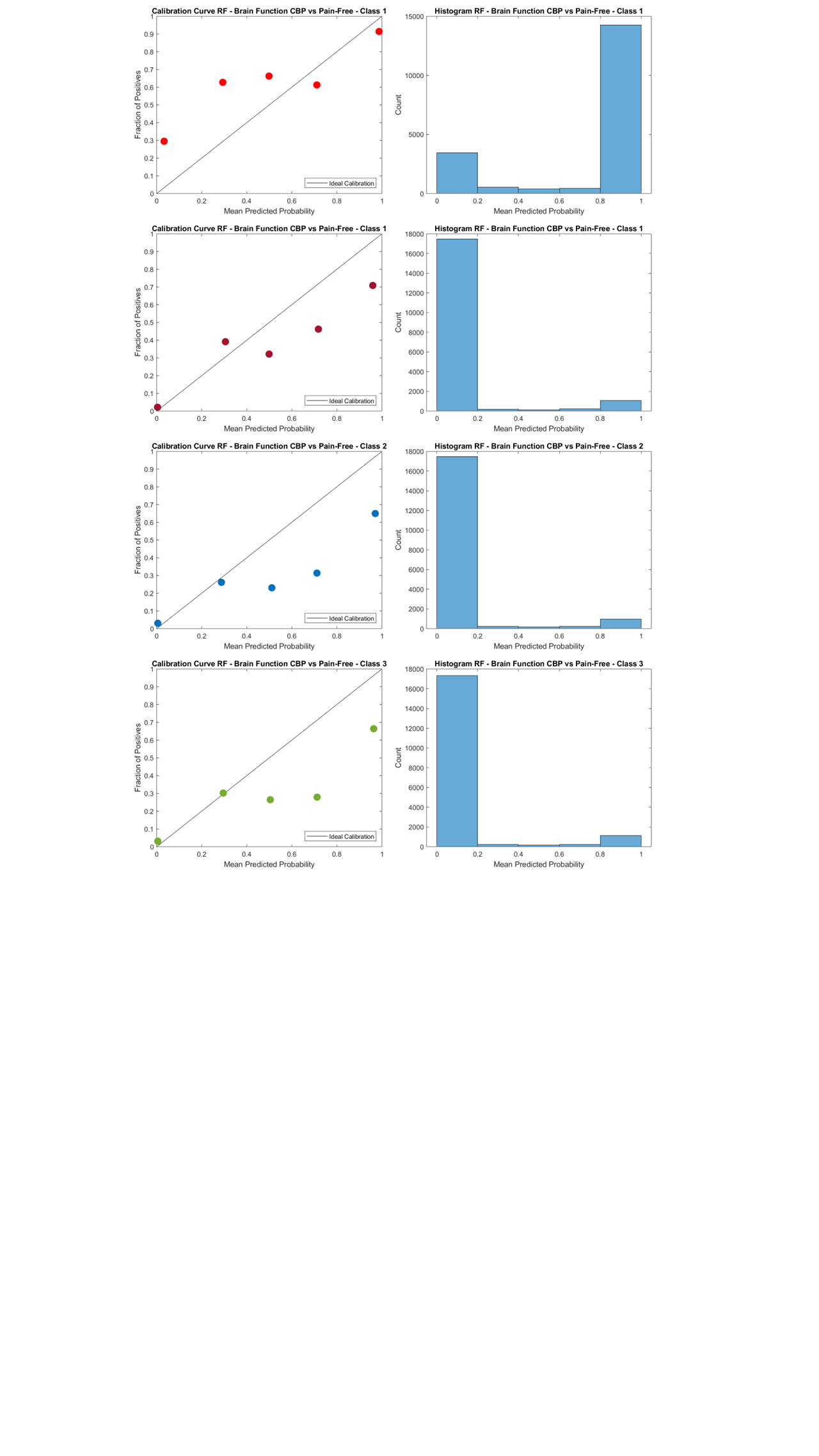


**Supplementary Figure 43.** Random Forest calibration curves for each class for classifiers in the brain function sub-domain analysis on CBP and pain-free individuals.


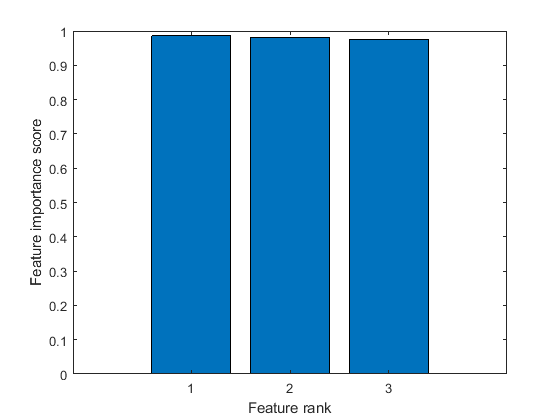


**Supplementary Figure 44.** Laplacian scores to rank brain structure features in the order of importance based on how they explain differences in datapoints in a single cohort (e.g. CBP only). For this data, the order of importance of features in a CBP-only space was fronto-orbital cortex, primary motor cortex and primary somatosensory cortex.


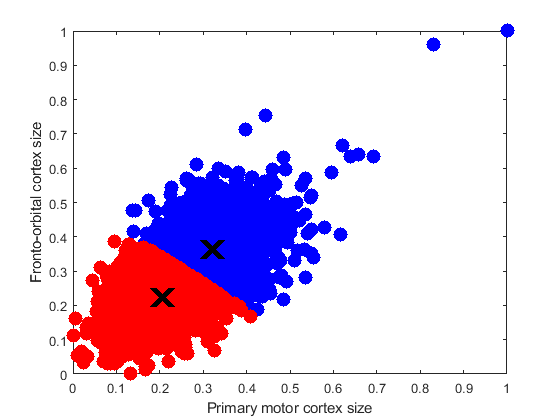


**Supplementary Figure 45.** Scatter plot of the distribution of sub-groups of individuals with CBP based on fronto-orbital and primary motor cortex sizes. Data is presented on (a) normal item range and (b) normalised scale of 0-1. Based on the centroids of fuzzy c-means clustering, classes and colours are (1; blue) higher fronto-orbital and primary motor cortex sizes (n=1,917; 46.1%); and (2; red) smaller fronto-orbital and primary motor cortex sizes (n=2,239; 53.9%). Black squares on the heat map indicate no class was available at those values. The X value on the scatter plot indicates the centroid of that cluster.


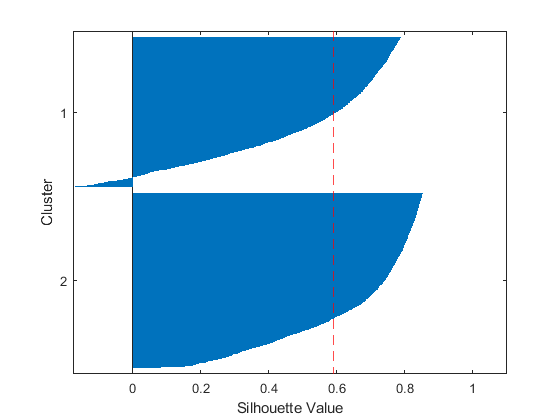


**Supplementary Figure 46.** Post clustering evaluation for clustering consisting of brain structure variables. The red line indicates the average of all the similarity measures. Values of 1 indicate good similarity of a datapoint to its cluster. The average Silhouette value is 0.589.


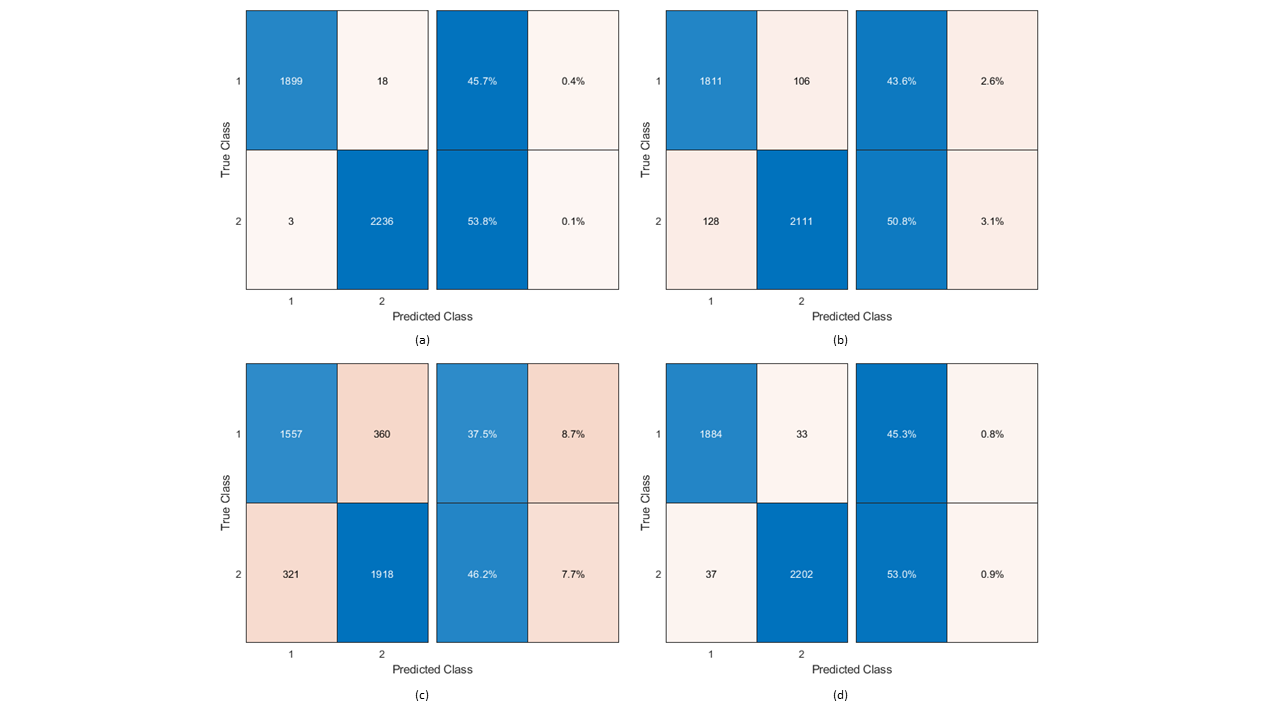


**Supplementary Figure 47.** Confusion matrix of classifiers on chronic back pain sub-groups (no pain-free controls included) based on brain structure with (a) Support Vector Machine, (b) Naïve Bayes, (c) k-Nearest Neighbour and (d) Random Forest classifiers. The x-axis is the predicted class while the y-axis is the true class. Blue squares indicate the number in the class that was accurately classified, while the oranges squares show the number of misclassifications. The boxes on the right of the matrix show the percentage of classification (blue) and misclassification (orange) for the class. Classes are (1) higher fronto-orbital and primary motor cortex sizes (n=1,917; 46.1%); and (2) smaller fronto‑orbital and primary motor cortex sizes (n=2,239; 53.9%).


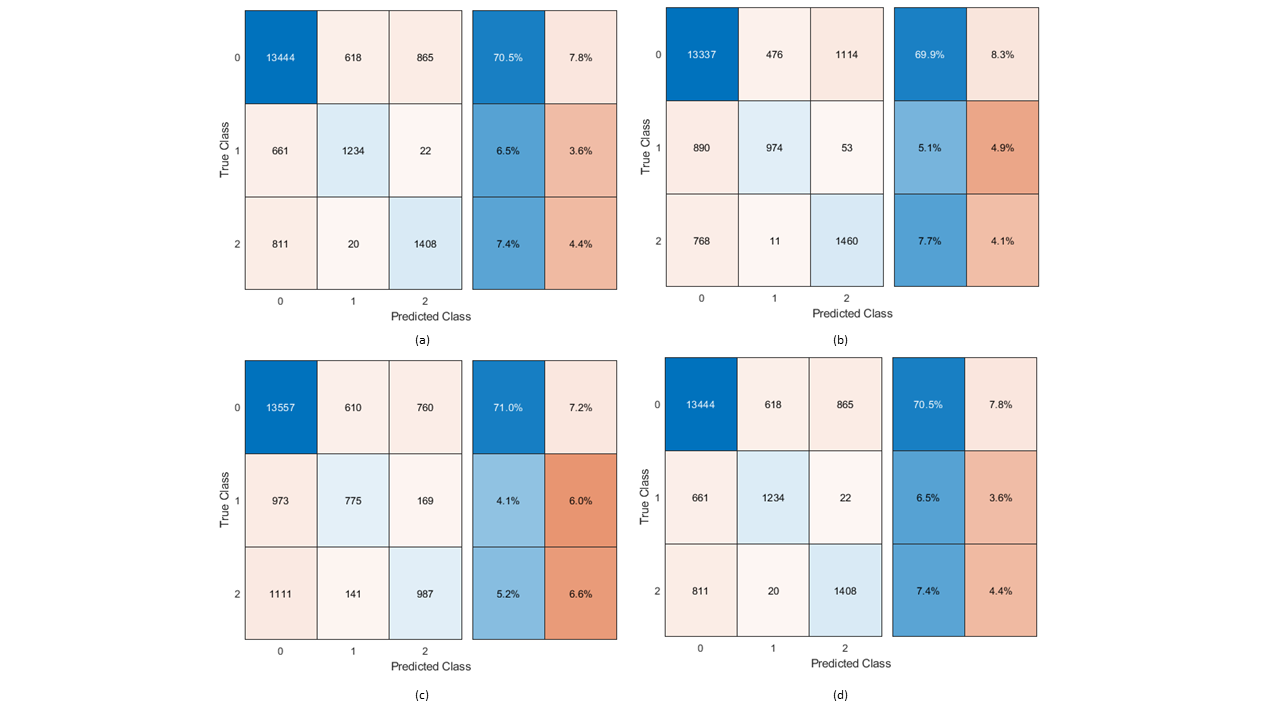


**Supplementary Figure 48.** Confusion matrix of classifiers on chronic back pain and pain-free sub-groups based on brain structure with (a) Support Vector Machine, (b) Naïve Bayes, (c) k-Nearest Neighbour and (d) Random Forest classifiers. The x-axis is the predicted class while the y-axis is the true class. Blue squares indicate the number in the class that was accurately classified, while the oranges squares show the number of misclassifications. The boxes on the right of the matrix show the percentage of classification (blue) and misclassification (orange) for the class. Classes are (0) pain-free individuals (n=14,927; 78.2%); (1) higher fronto-orbital and primary motor cortex sizes (n=1,917; 10.1%); and (2) smaller fronto‑orbital and primary motor cortex sizes (n=2,239; 11.7%).


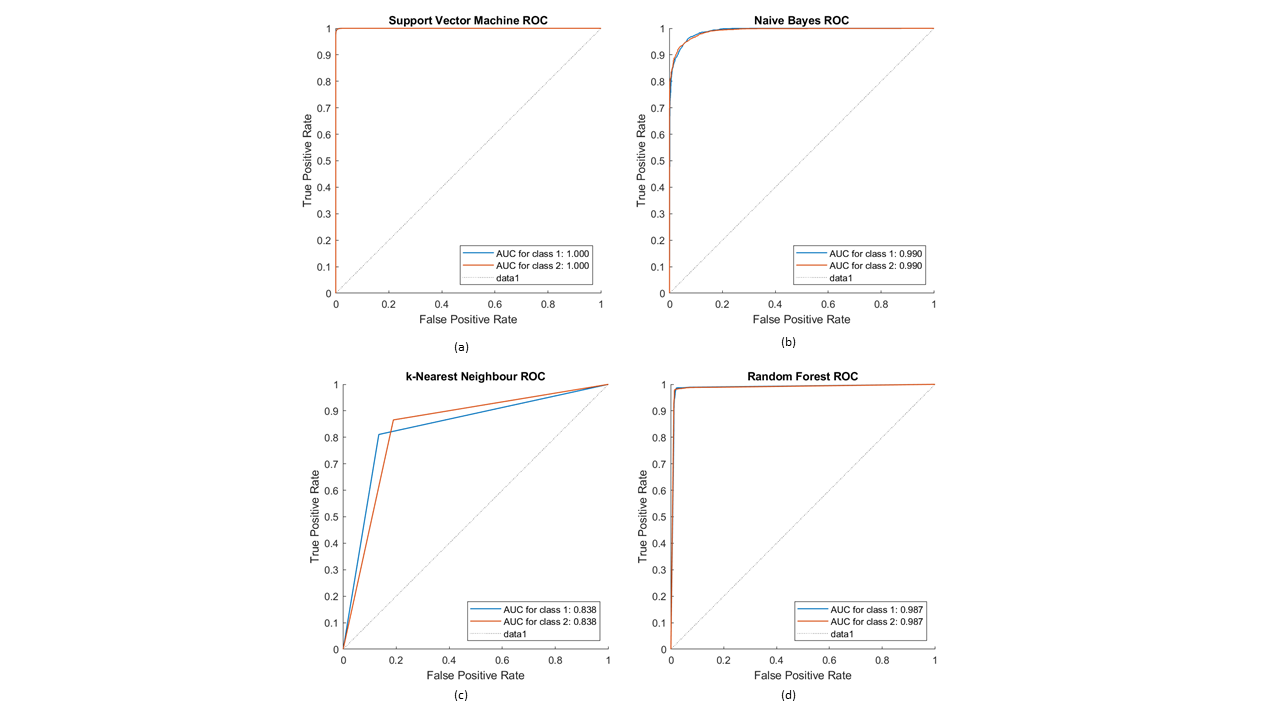


**Supplementary Figure 49.** Class specific area under the curve metrics data for classifiers in the brain structure sub-domain analysis on CBP individuals only.


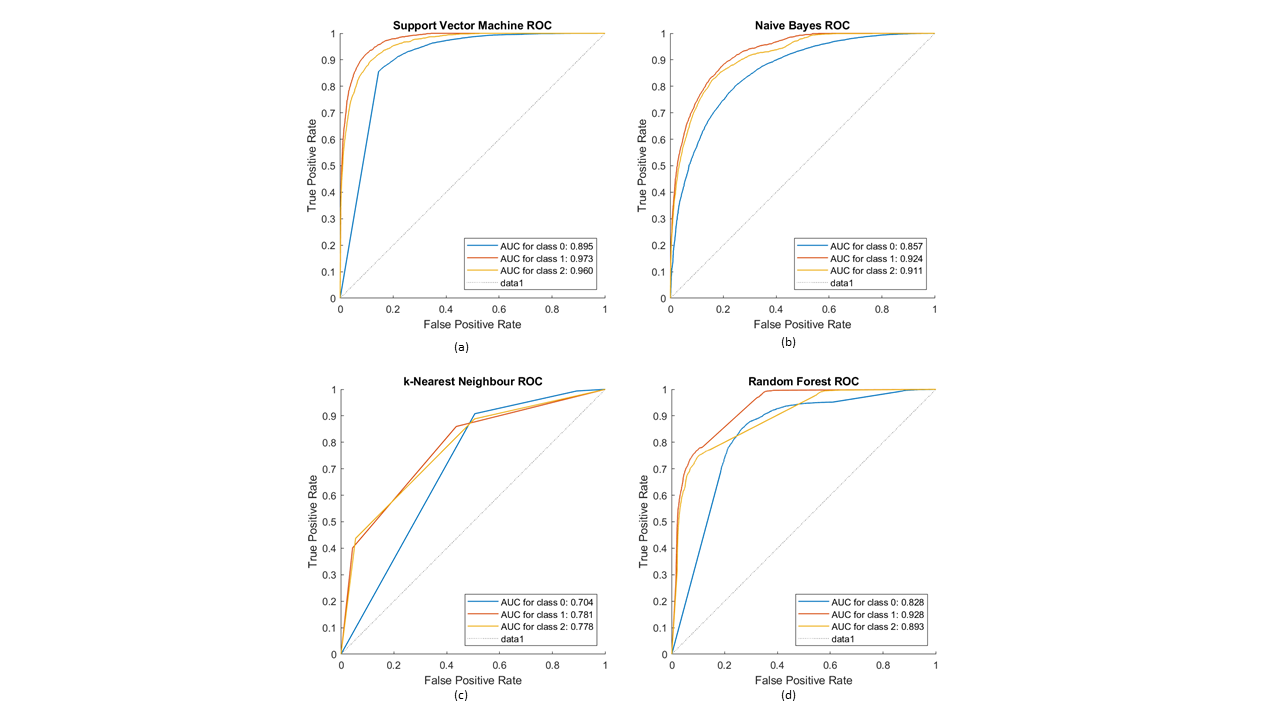


**Supplementary Figure 50.** Class specific area under the curve metrics data for classifiers in the brain structure sub-domain analysis on CBP and pain-free individuals.


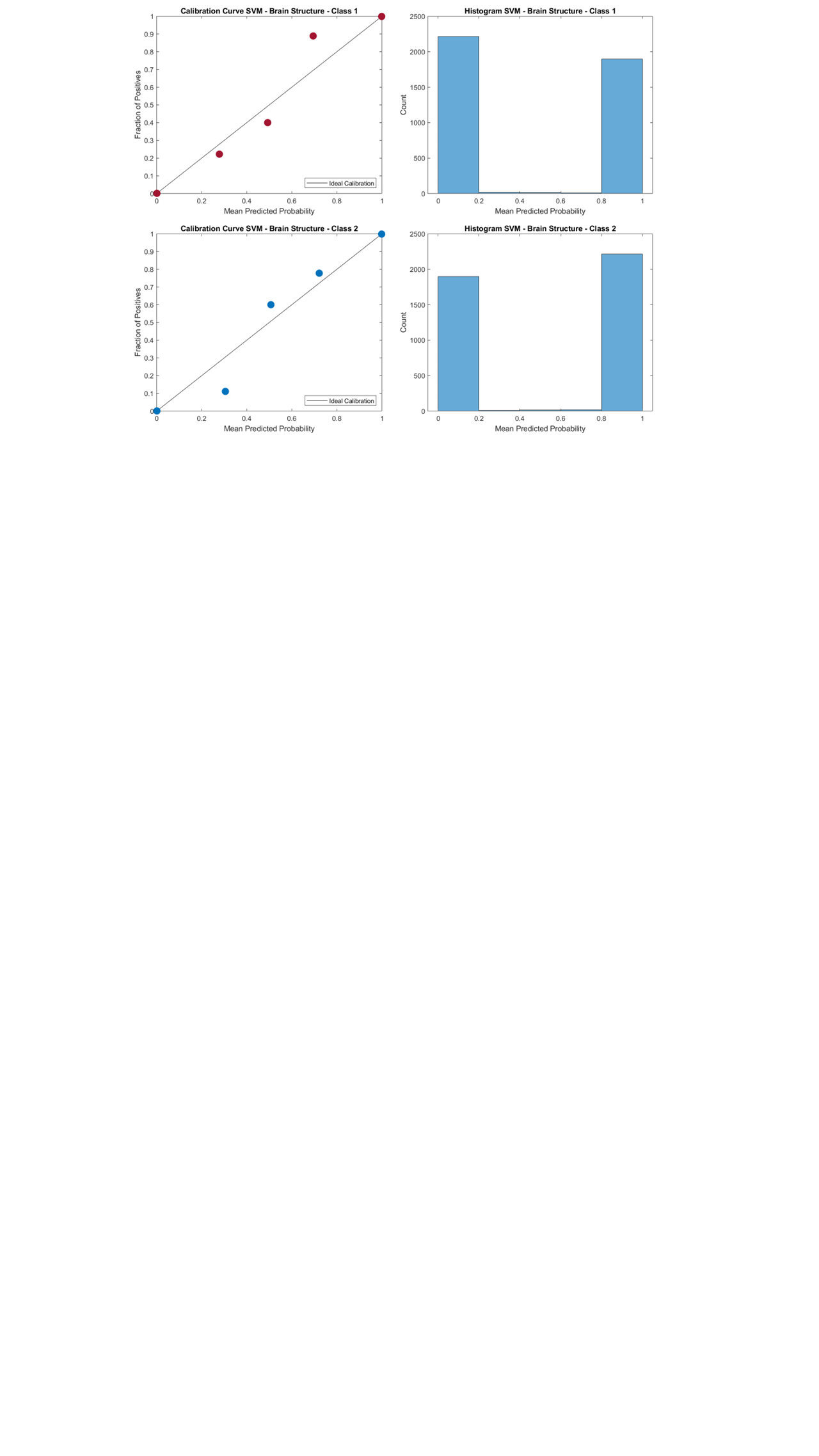


**Supplementary Figure 51.** Support vector machine calibration curves for each class for classifiers in the brain structure sub-domain analysis on CBP individuals only.


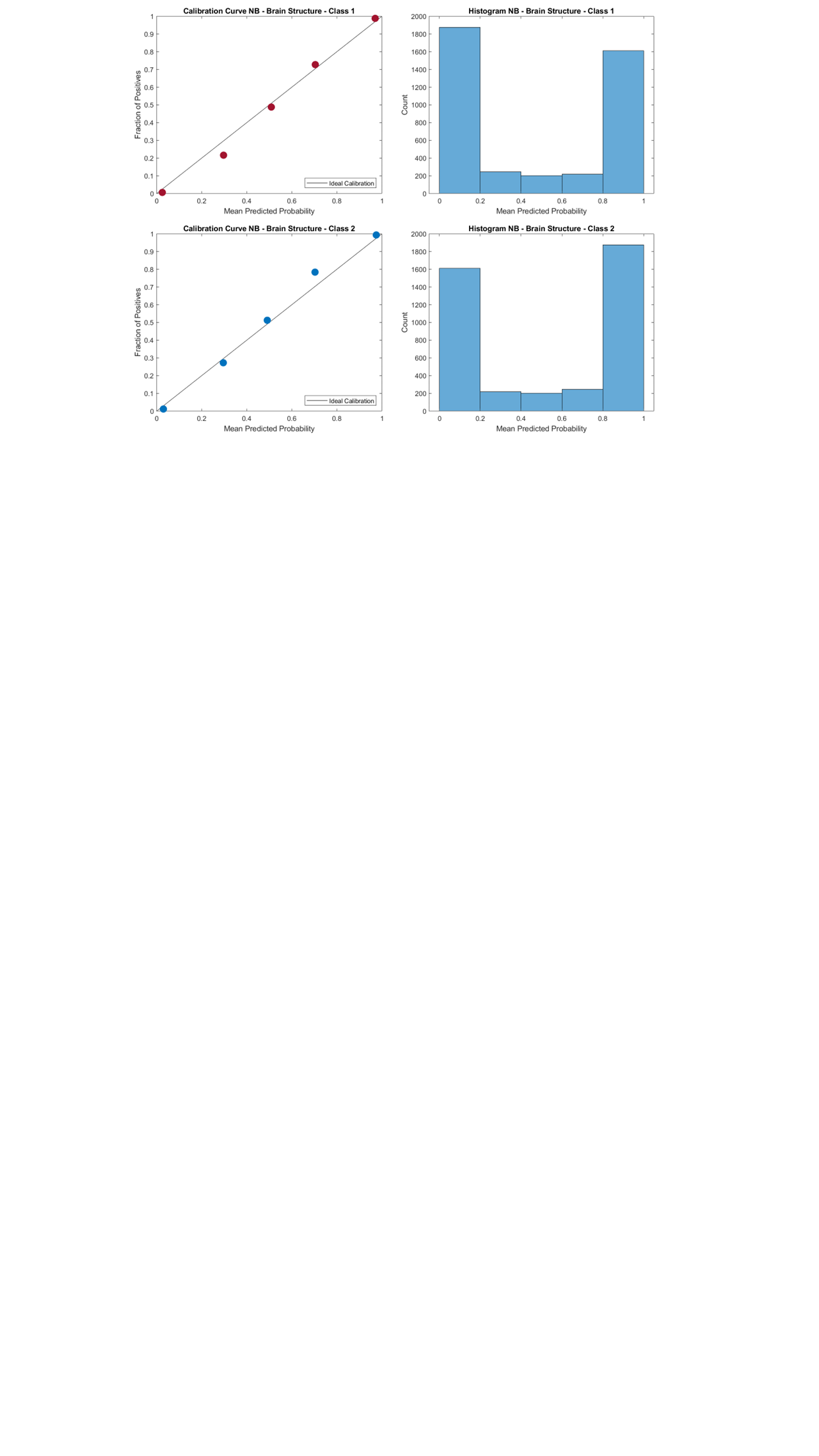


**Supplementary Figure 52.** Naïve Bayes calibration curves for each class for classifiers in the brain structure sub-domain analysis on CBP individuals only.


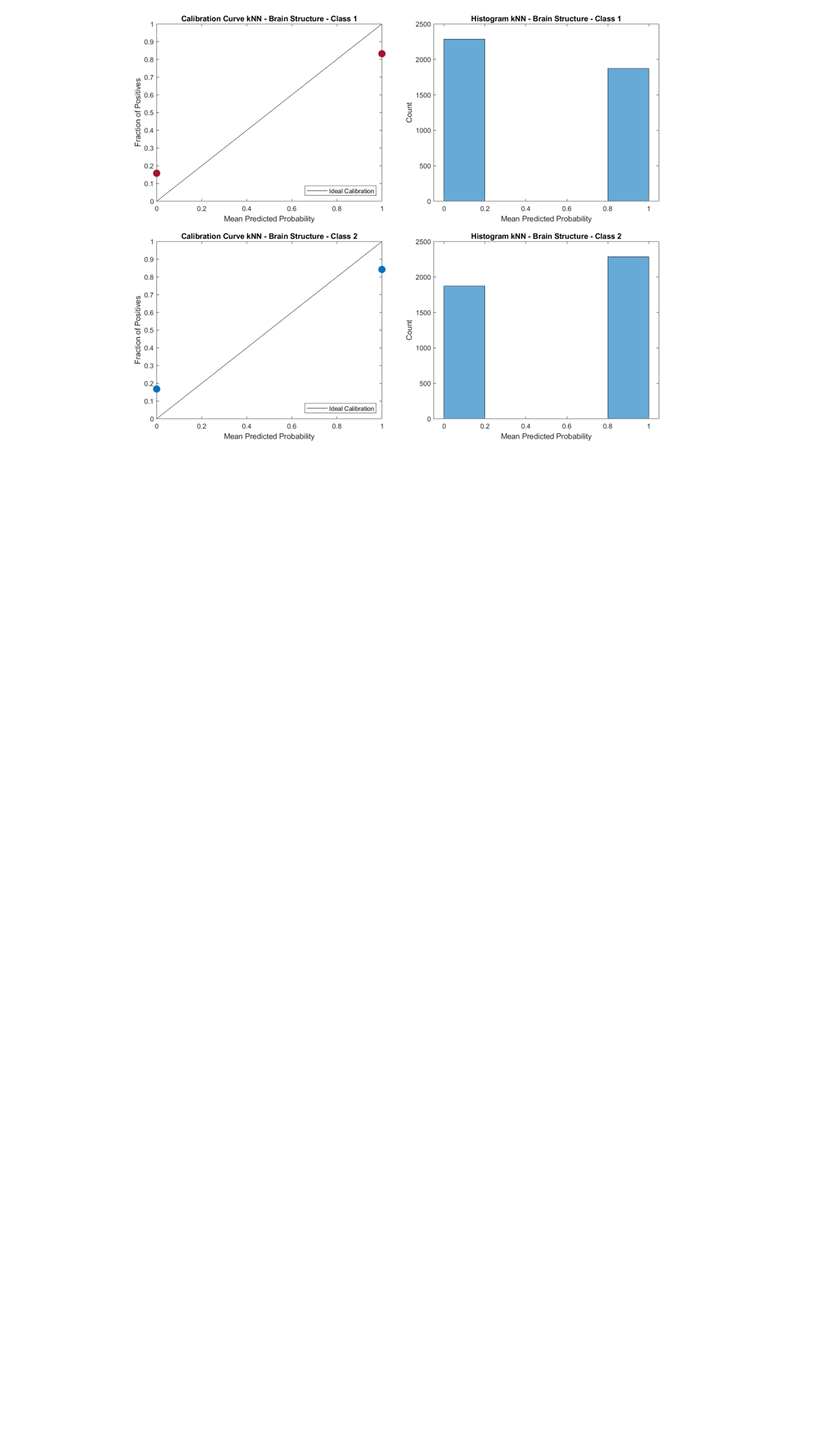


**Supplementary Figure 53.** K-Nearest Neighbour calibration curves for each class for classifiers in the brain structure sub-domain analysis on CBP individuals only.


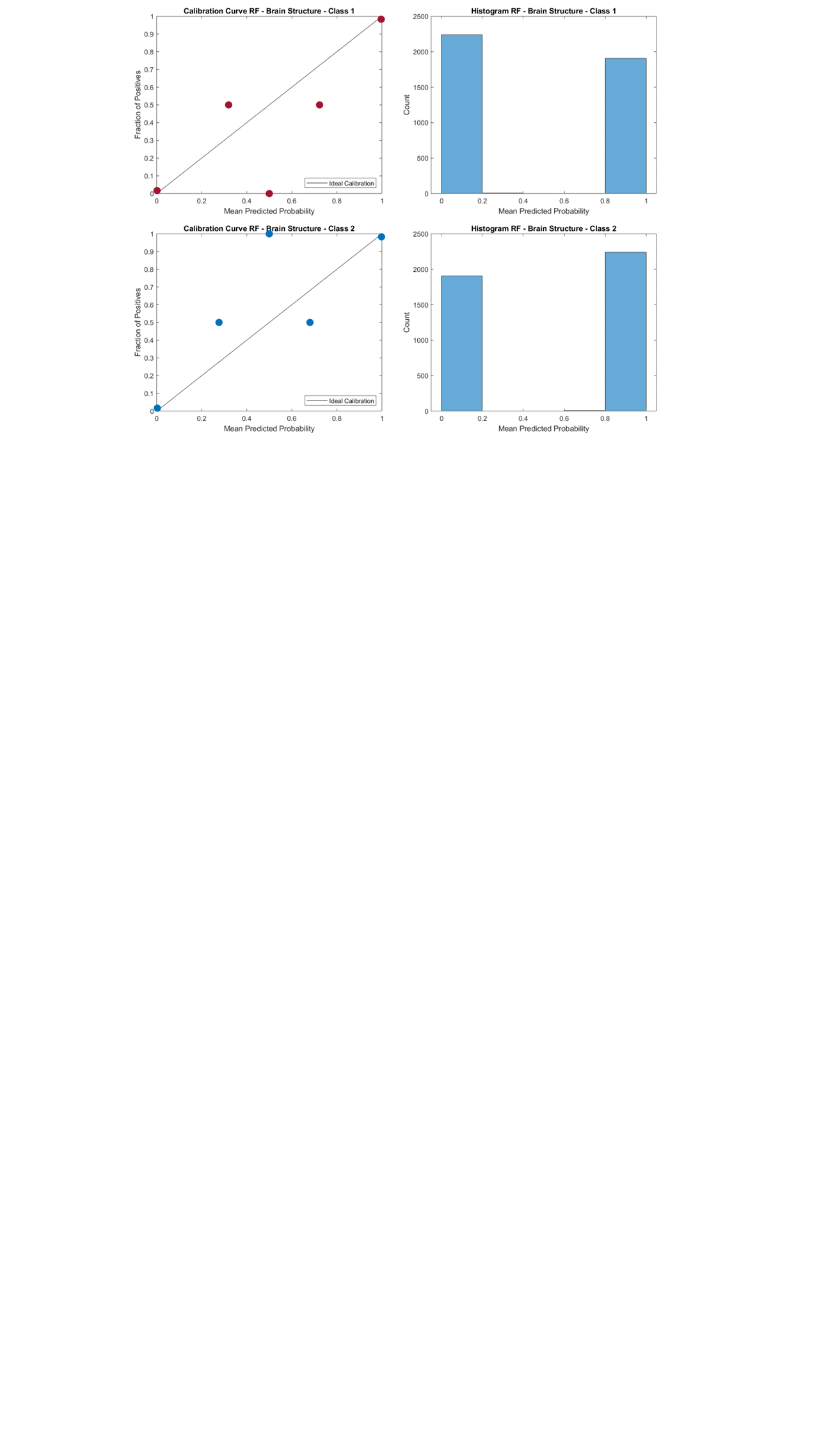


**Supplementary Figure 54.** Random Forest calibration curves for each class for classifiers in the brain structure sub-domain analysis on CBP individuals only.


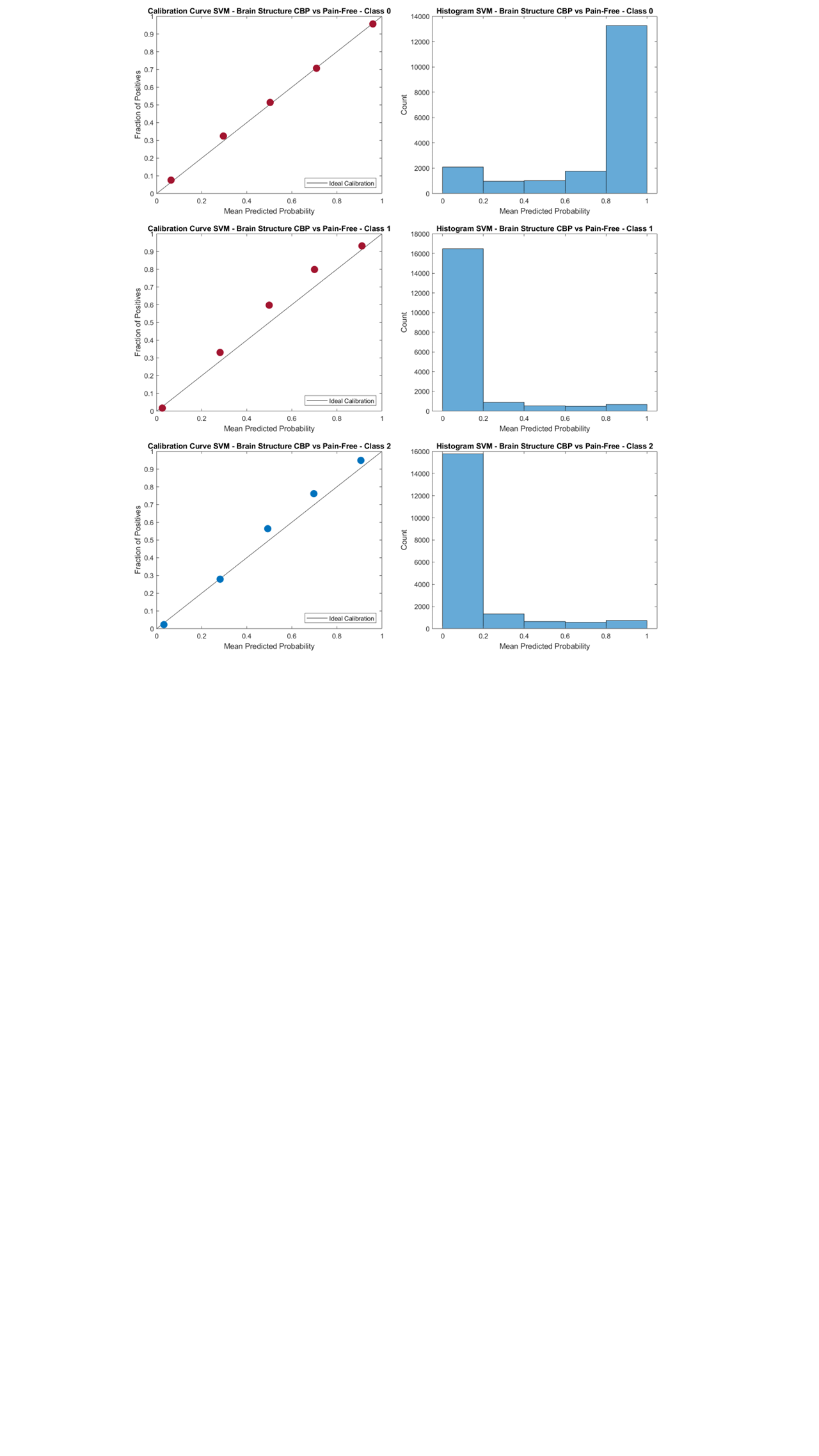


**Supplementary Figure 55.** Support vector machine calibration curves for each class for classifiers in the brain structure sub-domain analysis on CBP and pain-free individuals.


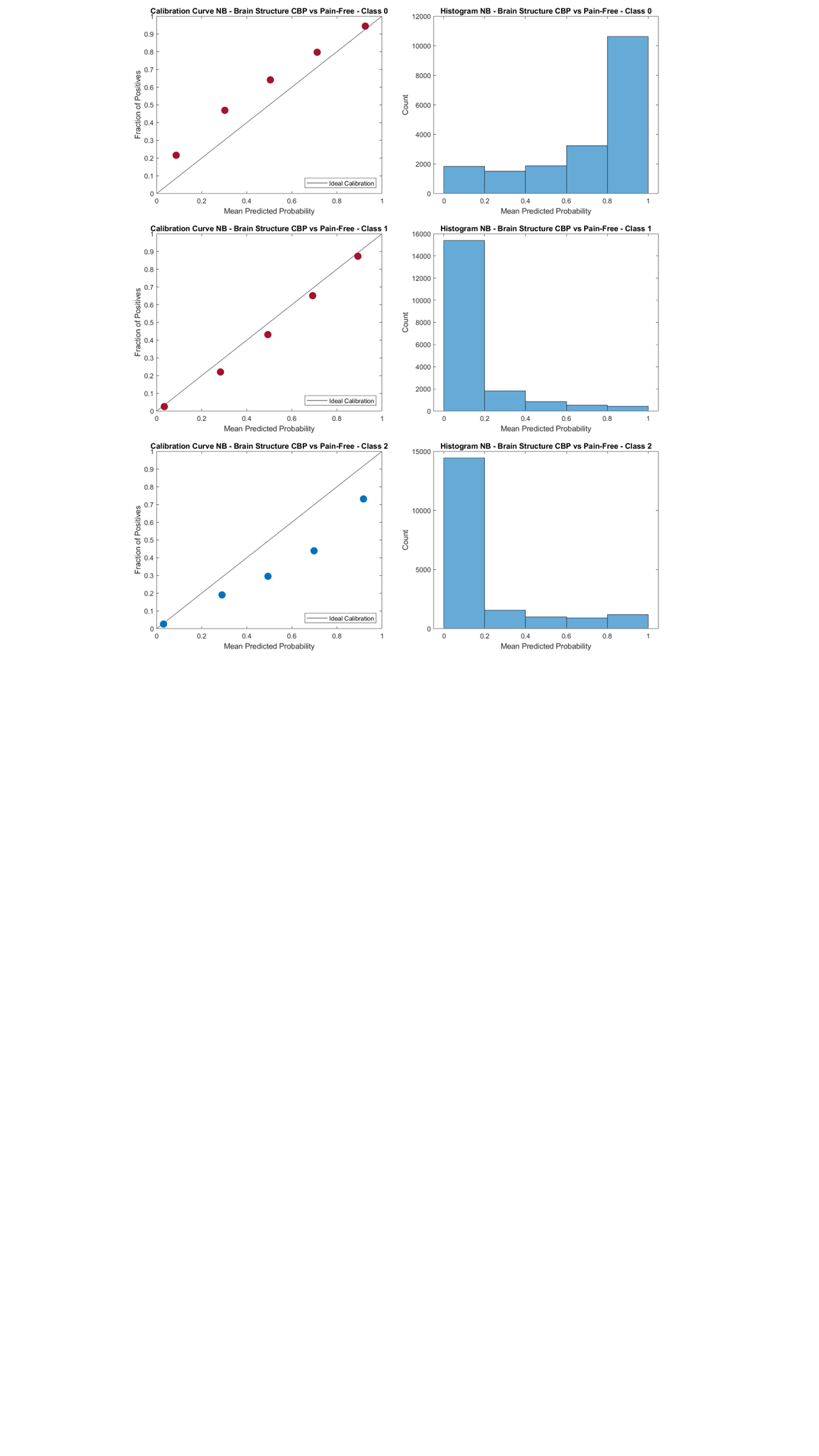


**Supplementary Figure 56.** Naïve Bayes calibration curves for each class for classifiers in the brain structure sub-domain analysis on CBP and pain-free individuals.


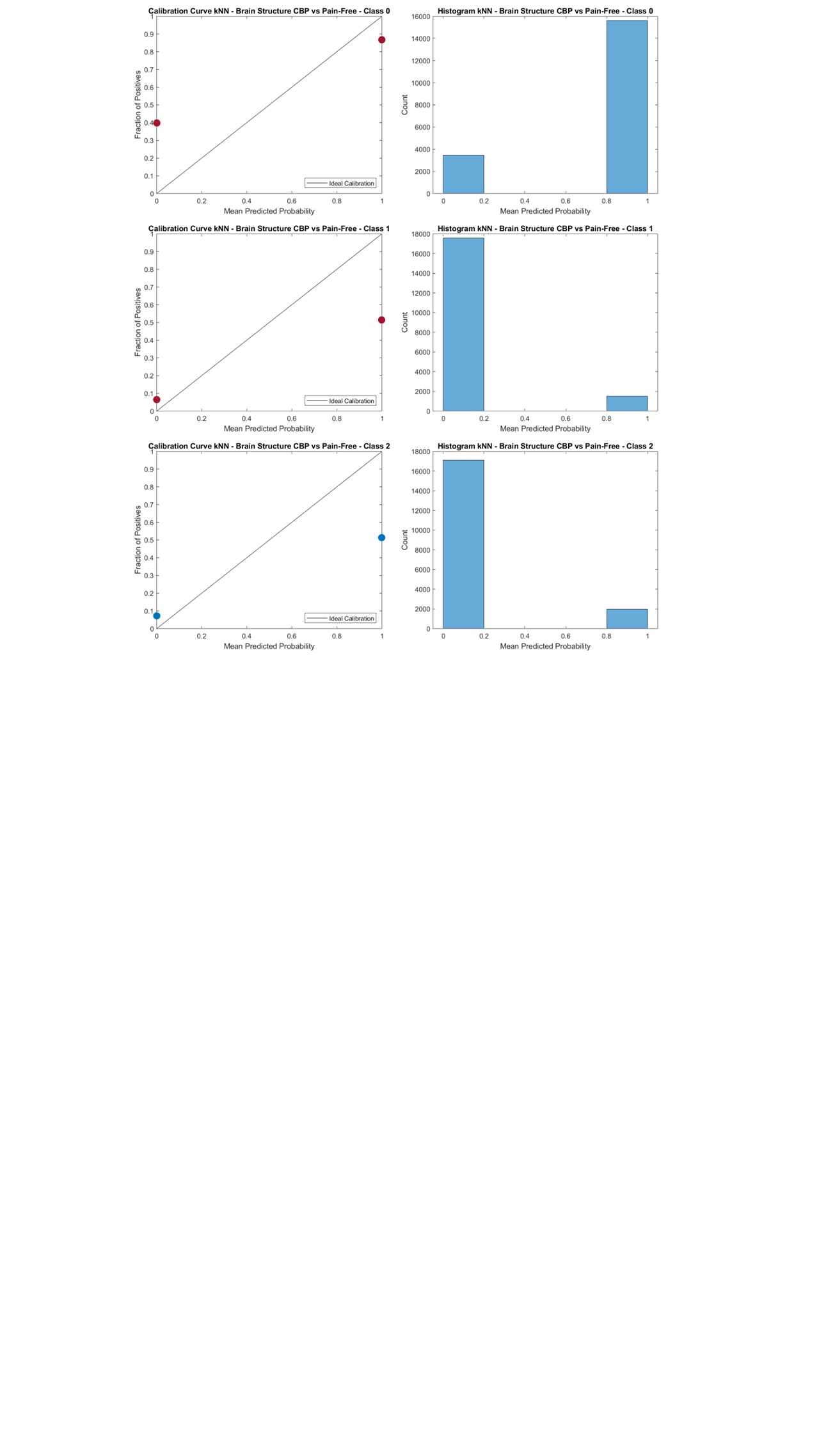


**Supplementary Figure 57.** K-Nearest Neighbour calibration curves for each class for classifiers in the brain structure sub-domain analysis on CBP and pain-free individuals.


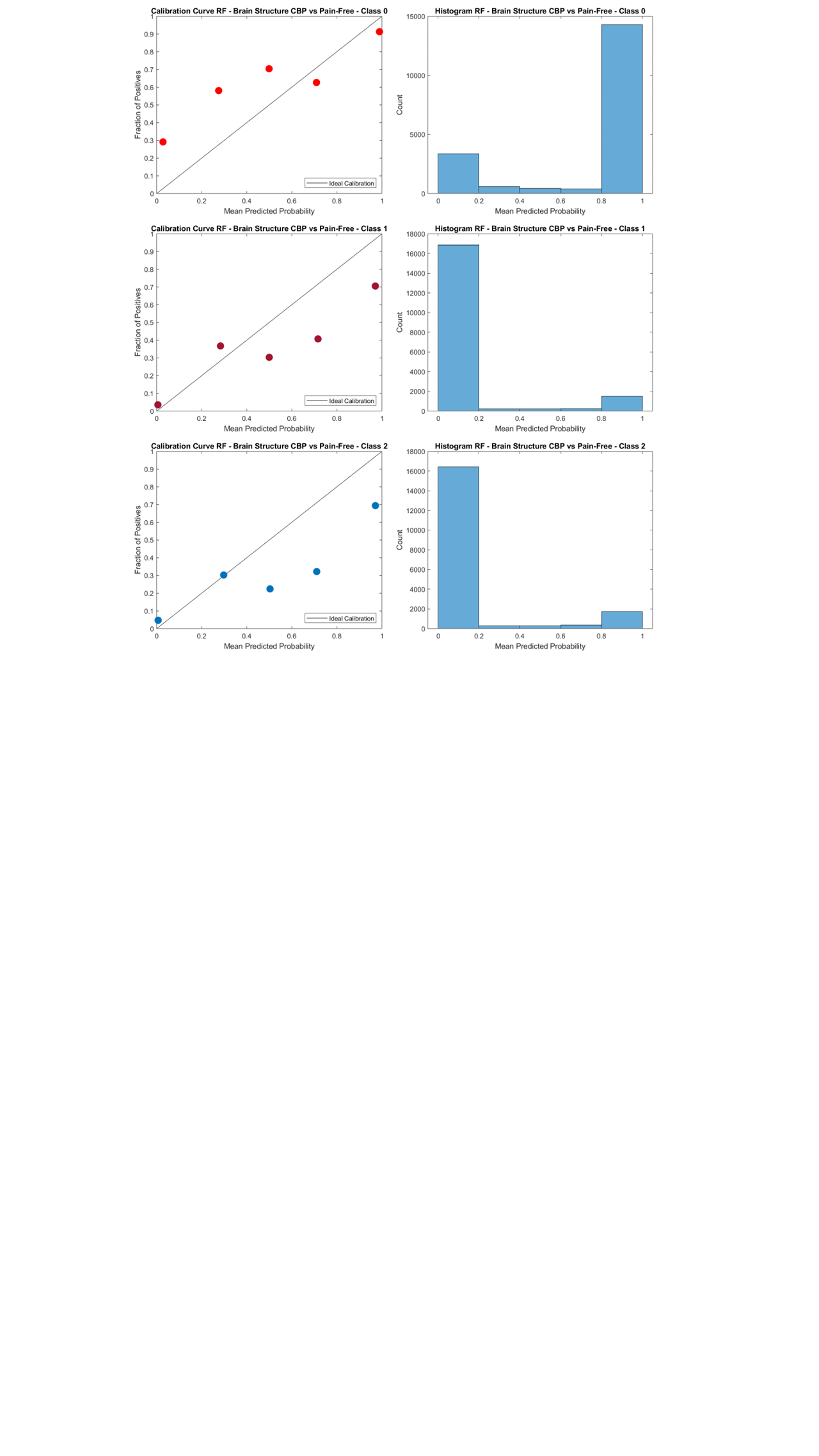


**Supplementary Figure 58.** Random Forest calibration curves for each class for classifiers in the brain structure sub-domain analysis on CBP and pain-free individuals.


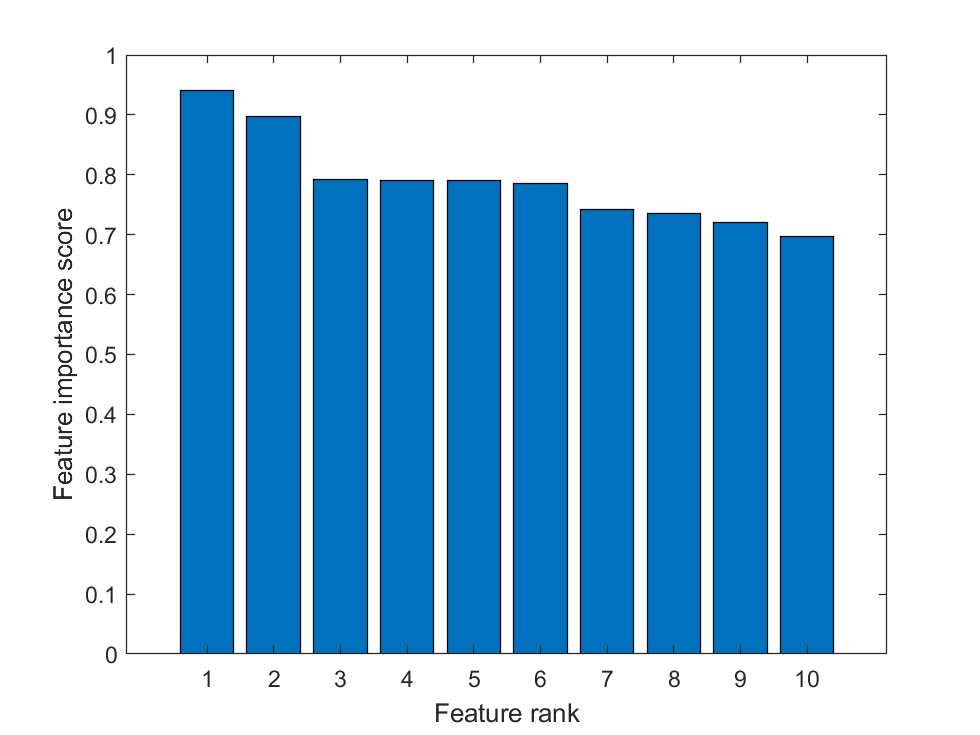


**Supplementary Figure 59.** Laplacian scores to rank features in the order of importance based on how they explain differences in datapoints in a single cohort (e.g. CBP only). This was conducted in the training data only. For this data, the order of importance of features in a CBP-only space was symptoms of loneliness/social isolation, depressive symptoms, body mass index, grip strength, inferior parietal lobe to middle temporal gyrus connectivity (node 1014), inferior parietal love to superior temporal gyrus connectivity (node 222), calcarine cortex to precuneus connectivity (node 44), primary motor cortex grey matter volume, fronto-orbital cortex grey matter volume and primary somatosensory grey matter volume.


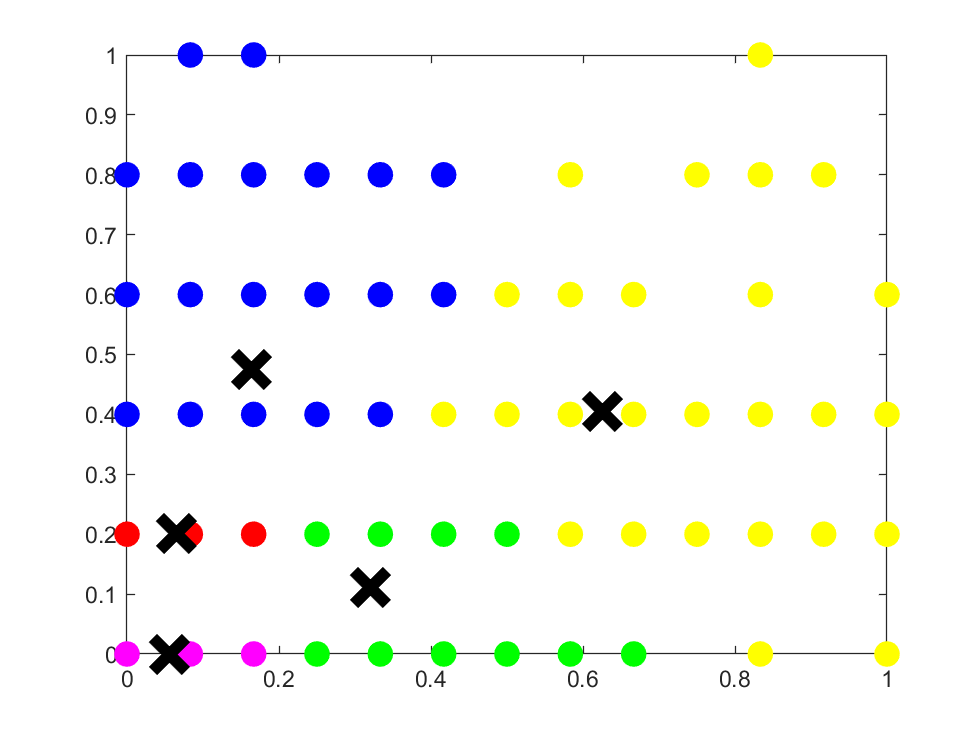


**Supplementary Figure 60.** Results of fuzzy c-means clustering in using only the training data. Clusters are (1; blue) higher social isolation (n=379; 18.2%), (2; red) normal values of both (n=562; 26.1%), (3; yellow) higher depressive symptoms and social isolation (n=135; 6.5%), (4; green) moderate depressive symptoms (n=317; 15.3%) and (5; pink) no symptoms of depression or social isolation (n=705; 33.9%).


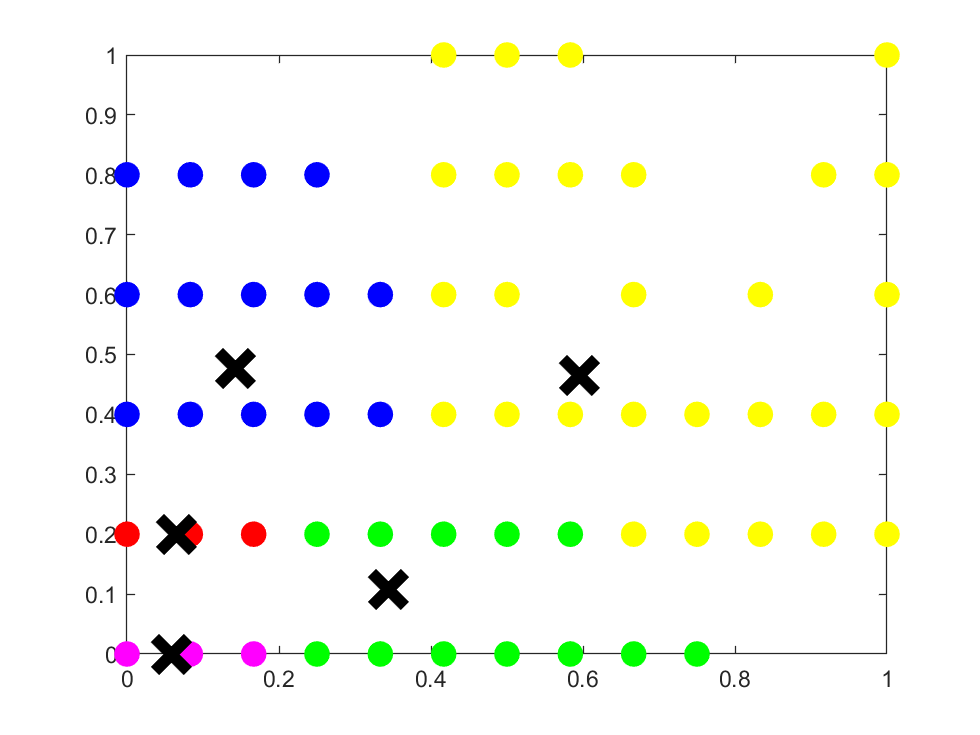


**Supplementary Figure 61.** Results of fuzzy c-means clustering in using only the testing data. Clusters are (1; blue) higher social isolation (n=351; 16.9%), (2; red) normal values of both (n=529; 25.4%), (3; yellow) higher depressive symptoms and social isolation (n=153; 7.4%), (4; green) moderate depressive symptoms (n=308; 14.8%) and (5; pink) no symptoms of depression or social isolation (n=737; 35.5%).


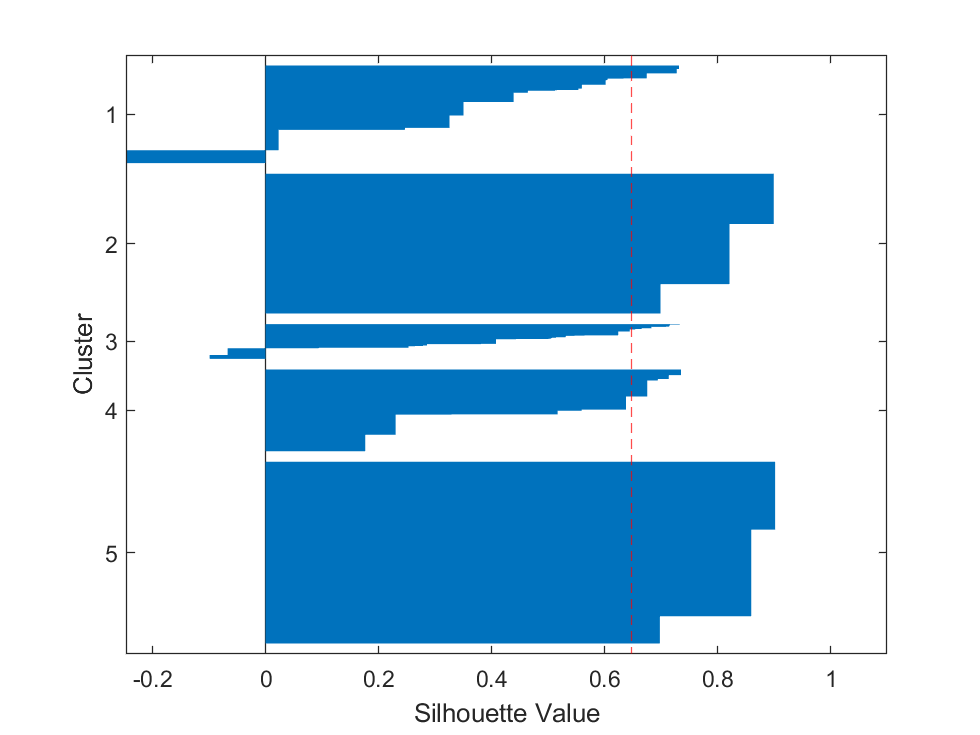


**Supplementary Figure 62.** Post clustering evaluation for clustering consisting of psychosocial variables in the training data only. The red line indicates the average of all the similarity measures. Values of 1 indicate good similarity of a datapoint to its cluster. The average Silhouette value is 0.65.


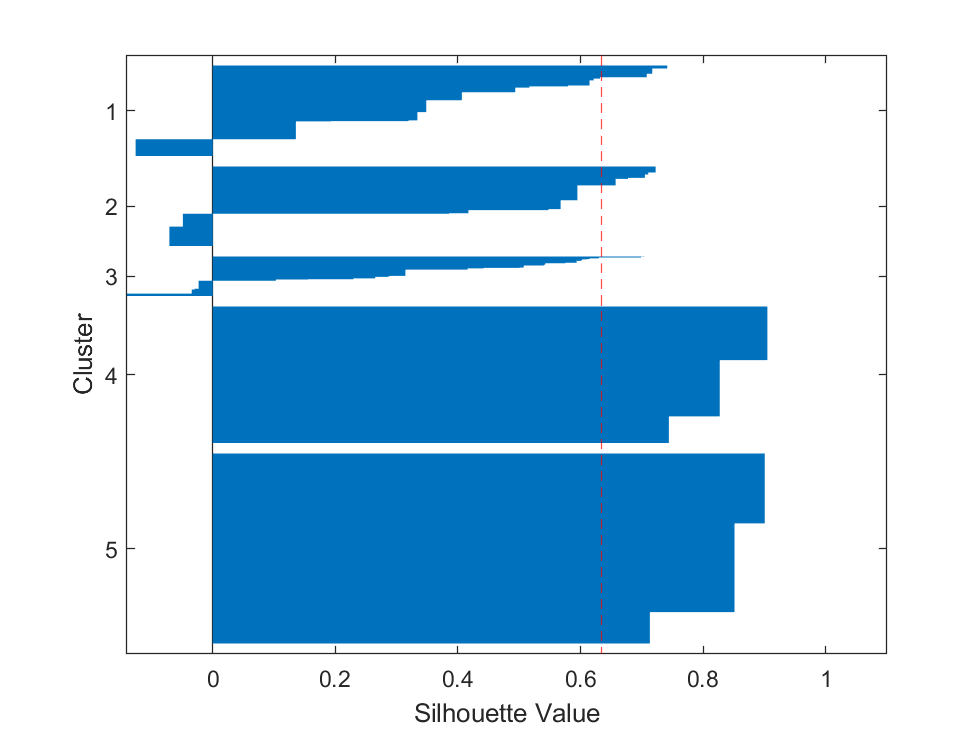


**Supplementary Figure 63.** Post clustering evaluation for clustering consisting of psychosocial variables in the testing data only. The red line indicates the average of all the similarity measures. Values of 1 indicate good similarity of a datapoint to its cluster. The average Silhouette value is 0.63.


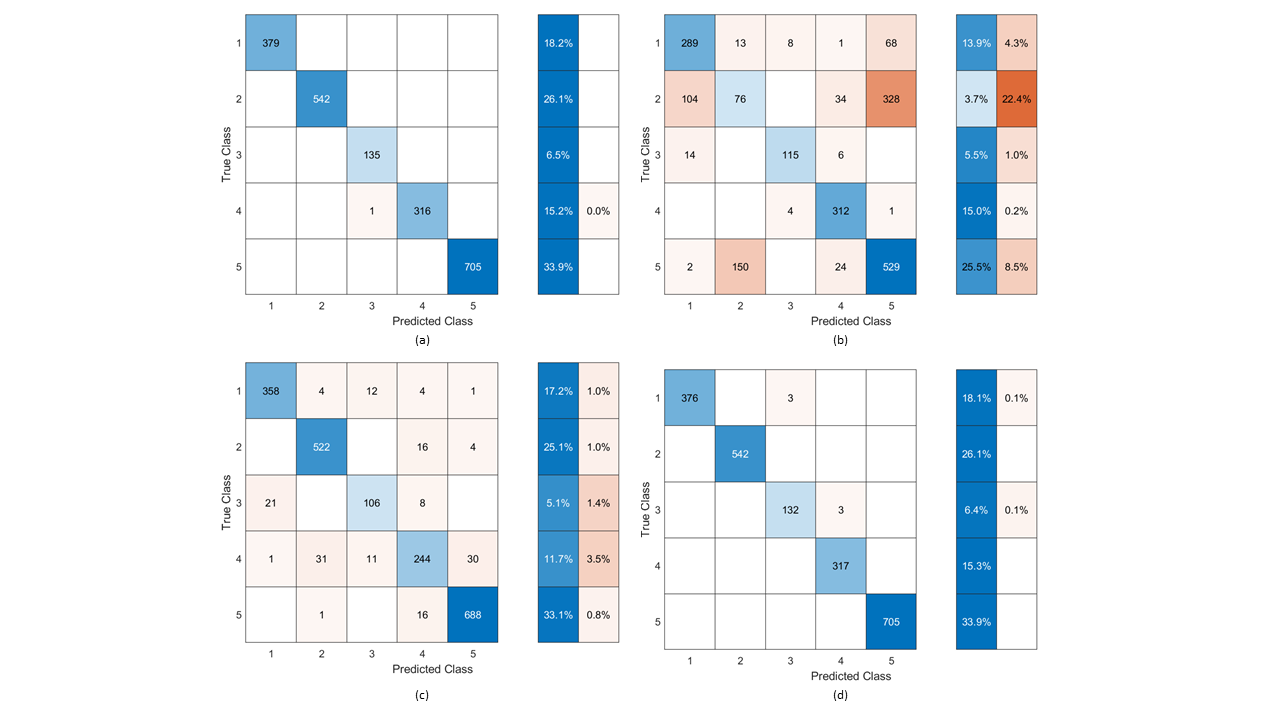


**Supplementary Figure 64.** Confusion matrix of classifiers on chronic back pain classes (no pain-free controls included) in the validation data only with (a) Support Vector Machine, (b) Naïve Bayes, (c) k-Nearest Neighbour and (d) Random Forest classifiers. The x-axis is the predicted class while the y-axis is the true class. Blue squares indicate the number in the class that was accurately classified, while the oranges squares show the number of misclassifications. The boxes on the right of the matrix show the percentage of classification (blue) and misclassification (orange) for the class.


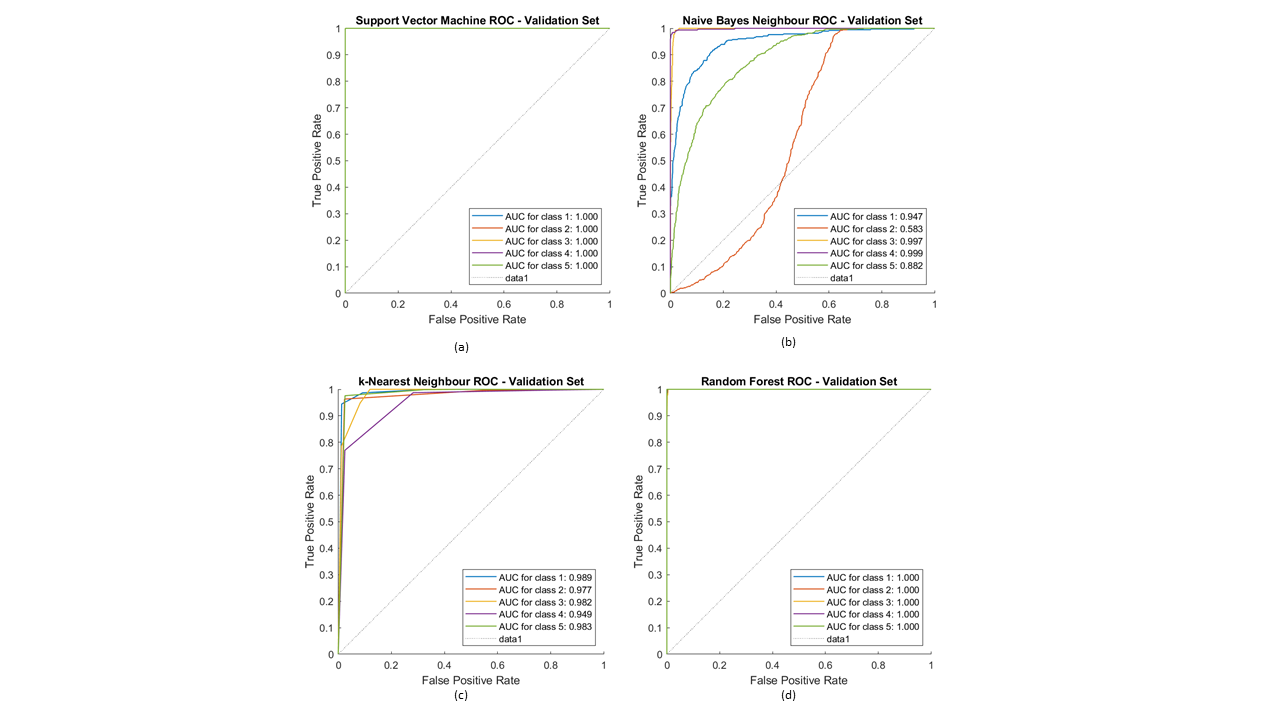


**Supplementary Figure 65.** Class specific area under the curve metrics data for classifiers in the main analysis on CBP individuals only using a training/validation set of the data.


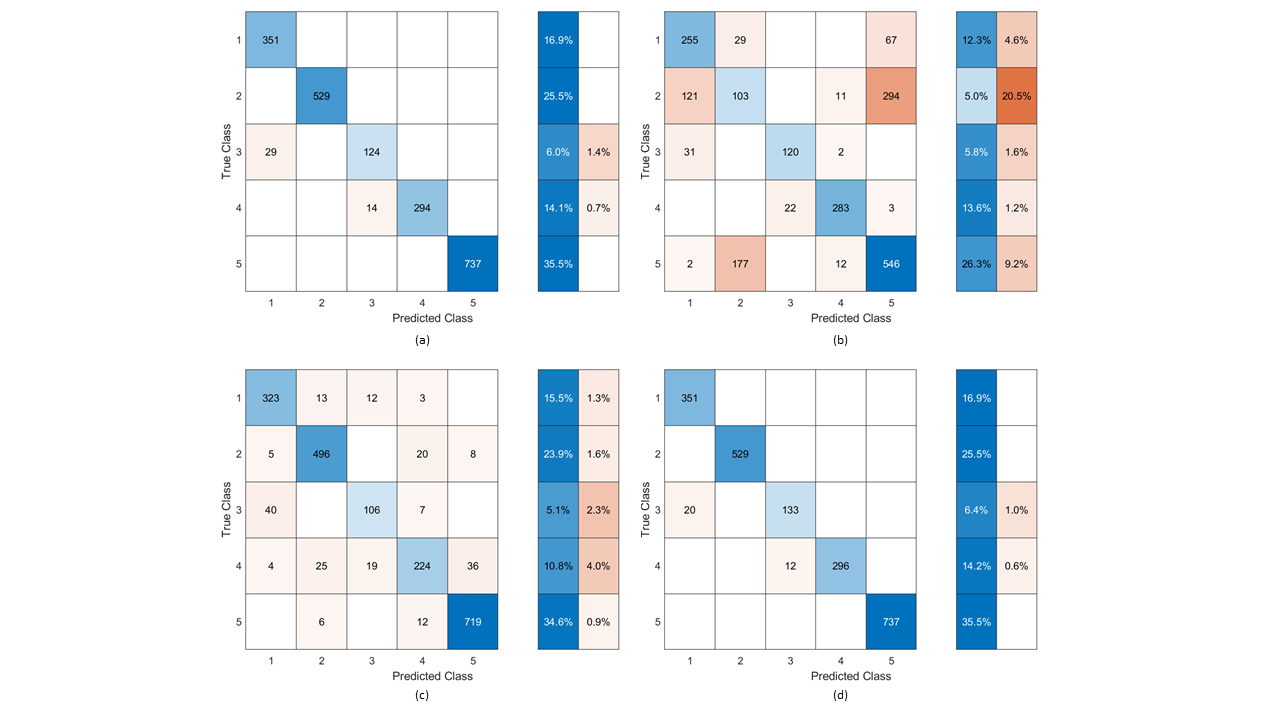


**Supplementary Figure 66.** Confusion matrix of classifiers on chronic back pain classes (no pain-free controls included) in the test data set only with (a) Support Vector Machine, (b) Naïve Bayes, (c) k-Nearest Neighbour and (d) Random Forest classifiers. The x-axis is the predicted class while the y-axis is the true class. Blue squares indicate the number in the class that was accurately classified, while the oranges squares show the number of misclassifications. The boxes on the right of the matrix show the percentage of classification (blue) and misclassification (orange) for the class.


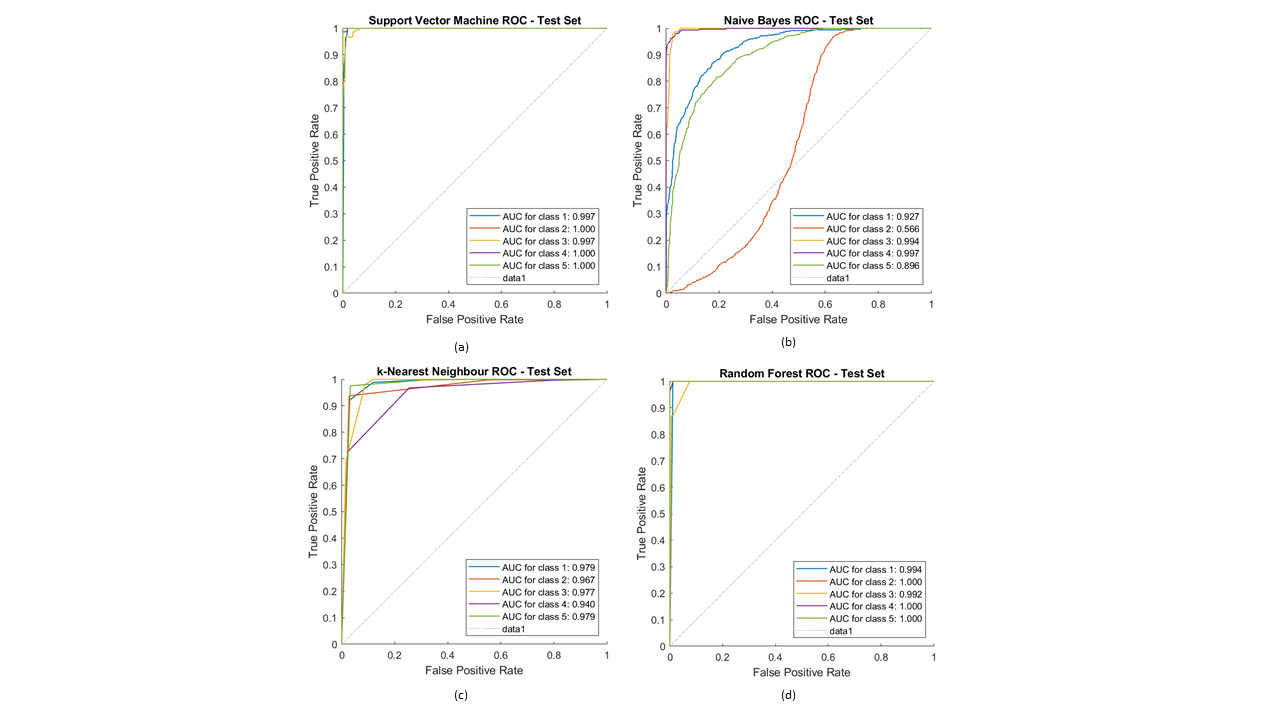


**Supplementary Figure 67.** Class specific area under the curve metrics data for classifiers in the main analysis on CBP individuals only using the testing set of the data.


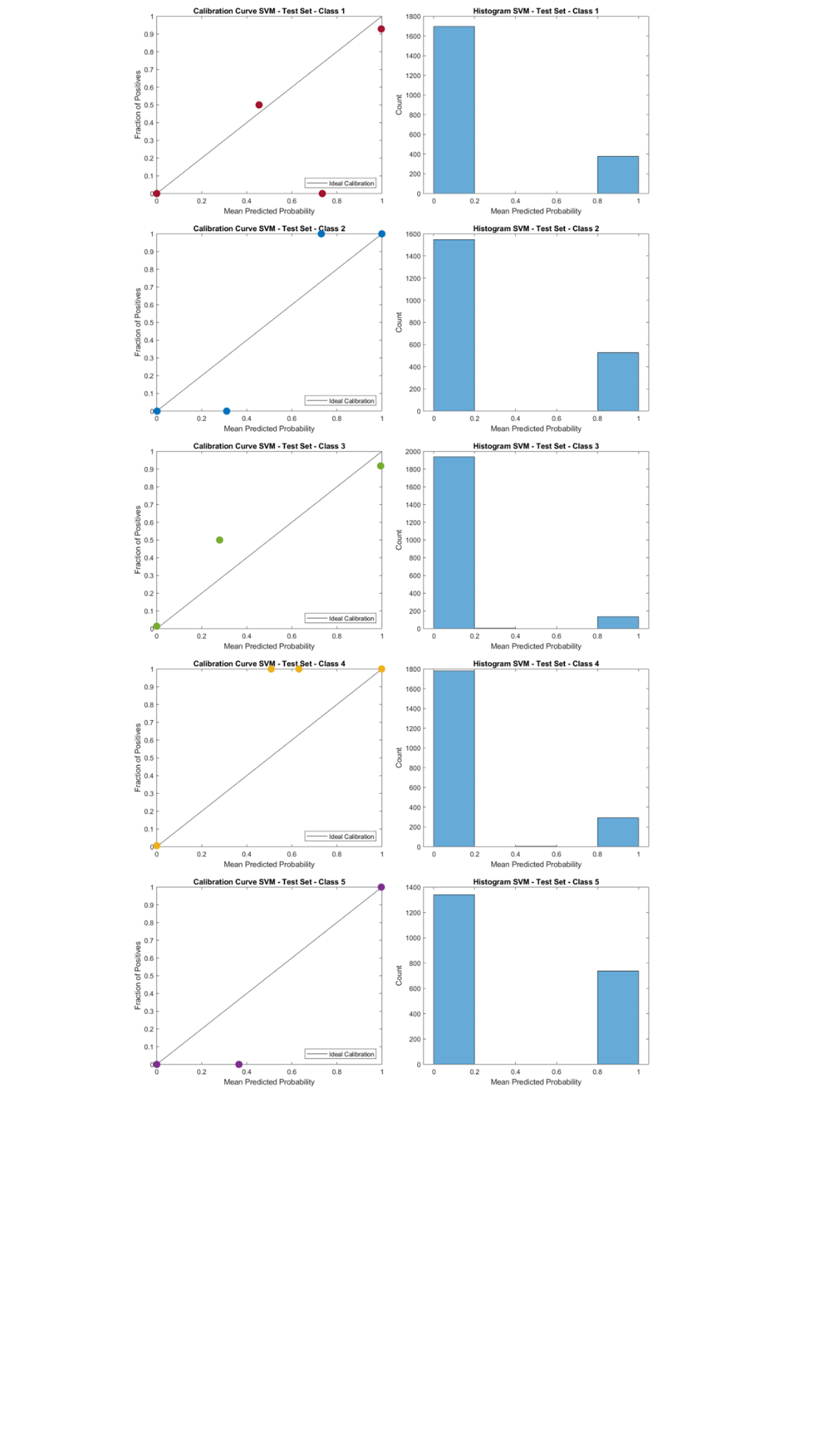


**Supplementary Figure 68.** Support vector machine calibration curves for each class for classifiers in the test data on CBP individuals only.


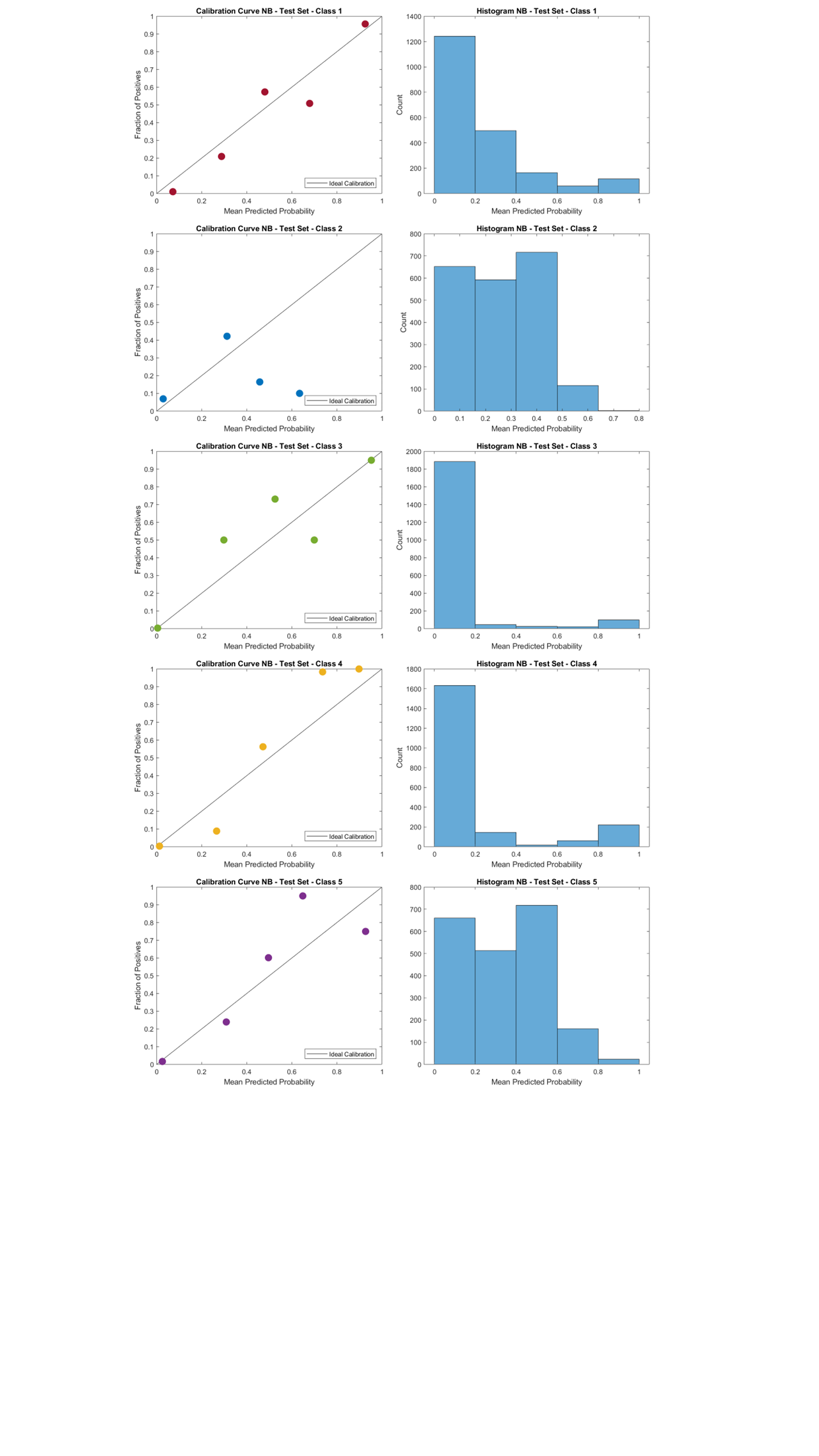


**Supplementary Figure 69.** Naïve Bayes calibration curves for each class for classifiers in the test data on CBP individuals only.


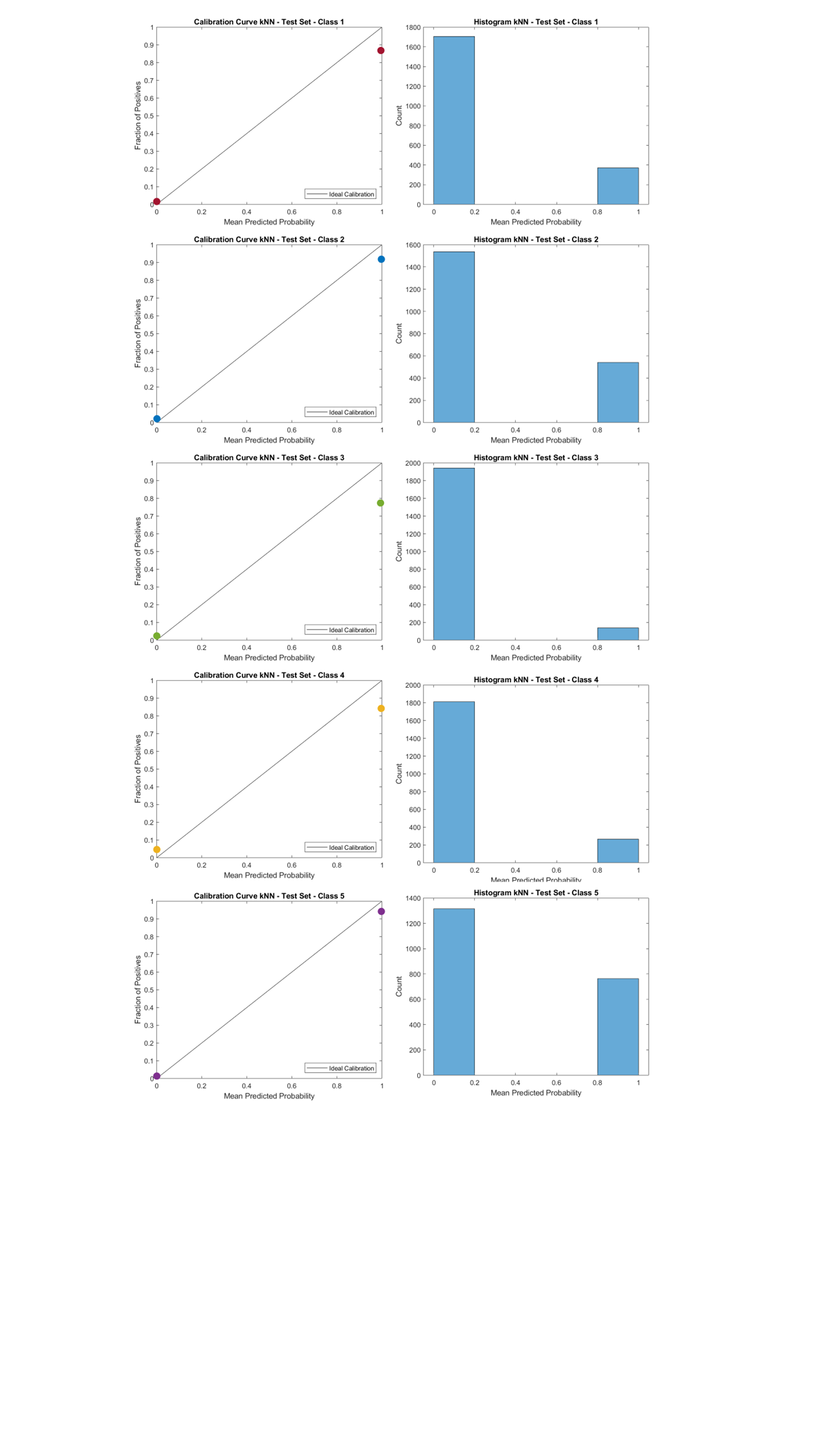


**Supplementary Figure 70.** K-Nearest Neighbour calibration curves for each class for classifiers in the test data on CBP individuals only.


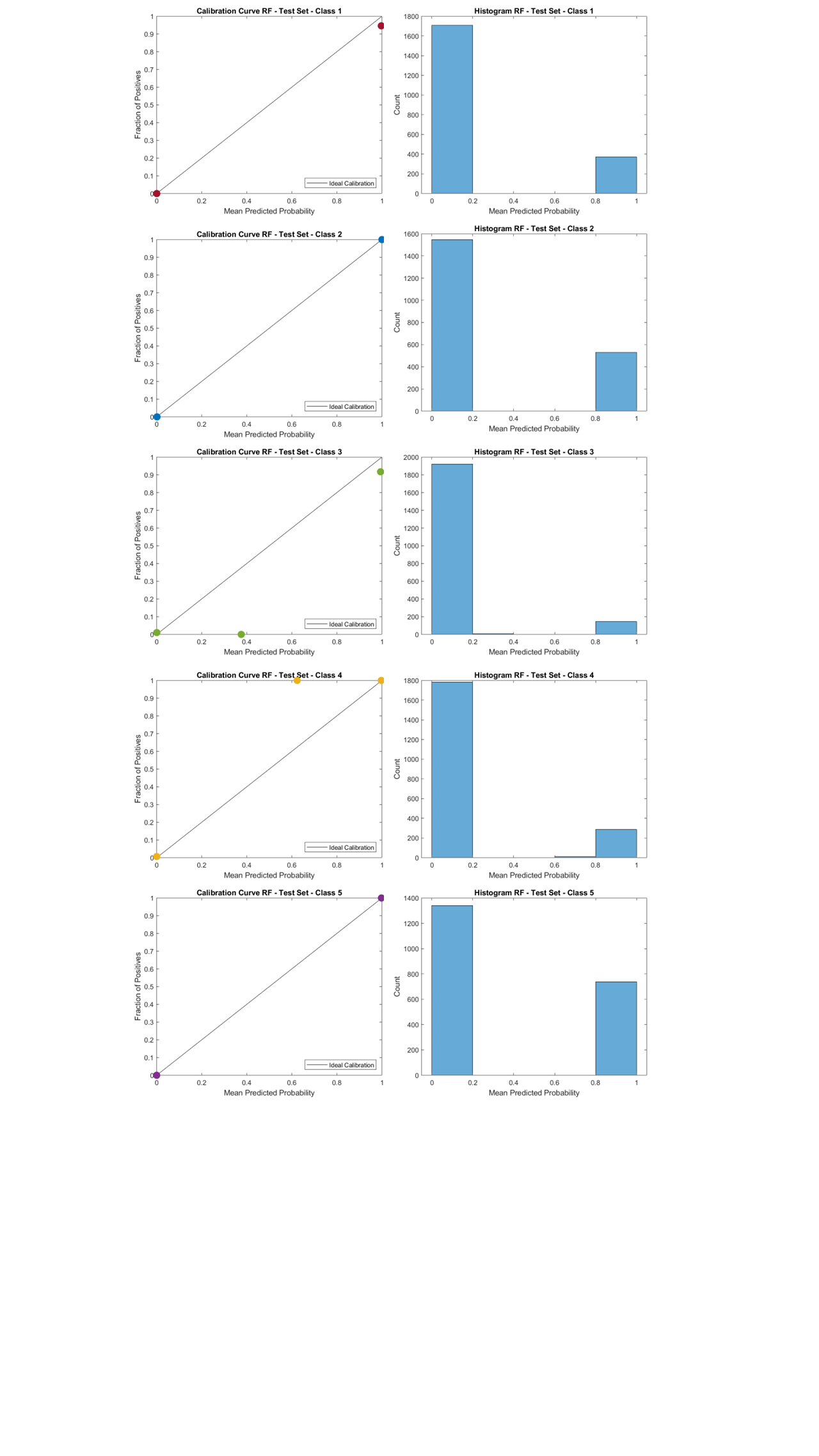


**Supplementary Figure 71.** Random Forest calibration curves for each class for classifiers in the test data on CBP individuals only.

**REFERENCES**

1. He, X., Cai, D. & Niyogi, P. Laplacian score for feature selection. *Adv. Neural Inf. Process. Syst.* 507–514 (2006).

2. Caliński, T. & Harabasz, J. A dendrite method for cluster analysis. *Commun. Stat.-Theory Methods* **3**, 1–27 (1974).

3. Davies, D. L. & Bouldin, D. W. A cluster separation measure. *IEEE Trans. Pattern Anal. Mach. Intell.* 224–227 (1979).

4. Kaufman, L. & Rousseeuw, P. J. *Finding groups in data: an introduction to cluster analysis*. vol. 344 (John Wiley & Sons, 2009).

5. Krauss, P. *et al.* A statistical method for analyzing and comparing spatiotemporal cortical activation patterns. *Sci. Rep.* **8**, 5433 (2018).

6. Bezdek, J. C. *Pattern recognition with fuzzy objective function algorithms*. (Springer Science & Business Media, 2013).

7. Khushaba, R. N., Al-Jumaily, A. & Al-Ani, A. Novel feature extraction method based on fuzzy entropy and wavelet packet transform for myoelectric control. *2007 Int. Symp. Commun. Inf. Technol.* 352–357 (2007).

8. Fradkin, D. & Muchnik, I. Support vector machines for classification. *DIMACS Ser. Discrete Math. Theor. Comput. Sci.* **70**, 13–20 (2006).

9. Rish, I. An empirical study of the naive Bayes classifier. *IJCAI 2001 Workshop Empir. Methods Artif. Intell.* **3**, 41–46 (2001).

10. Weinberger, K. Q., Blitzer, J. & Saul, L. K. Distance metric learning for large margin nearest neighbor classification. *Adv. Neural Inf. Process. Syst.* 1473–1480 (2006).

11. Breiman, L. Random forests. *Mach. Learn.* **45**, 5–32 (2001).

12. Huang, Y., Li, W., Macheret, F., Gabriel, R. A. & Ohno-Machado, L. A tutorial on calibration measurements and calibration models for clinical prediction models. *J. Am. Med. Inform. Assoc.* **27**, 621–633 (2020).

13. Painsky, A. & Wornell, G. On the Universality of the Logistic Loss Function. in *2018 IEEE International Symposium on Information Theory (ISIT)* 936–940 (2018). doi:10.1109/ISIT.2018.8437786.
